# Supplementary material for: Overexpression and Biochemical Characterization of an Endo-α-1,4-polygalacturonase from Aspergillus nidulans in Pichia pastoris
Source: Int J Mol Sci. 2020 Mar 19;21(6):2100. doi: 10.3390/ijms21062100 (PMC7139513; doi:10.3390/ijms21062100)
Supplement: Supplementary file 1 [file ijms-21-02100-s001.pdf]

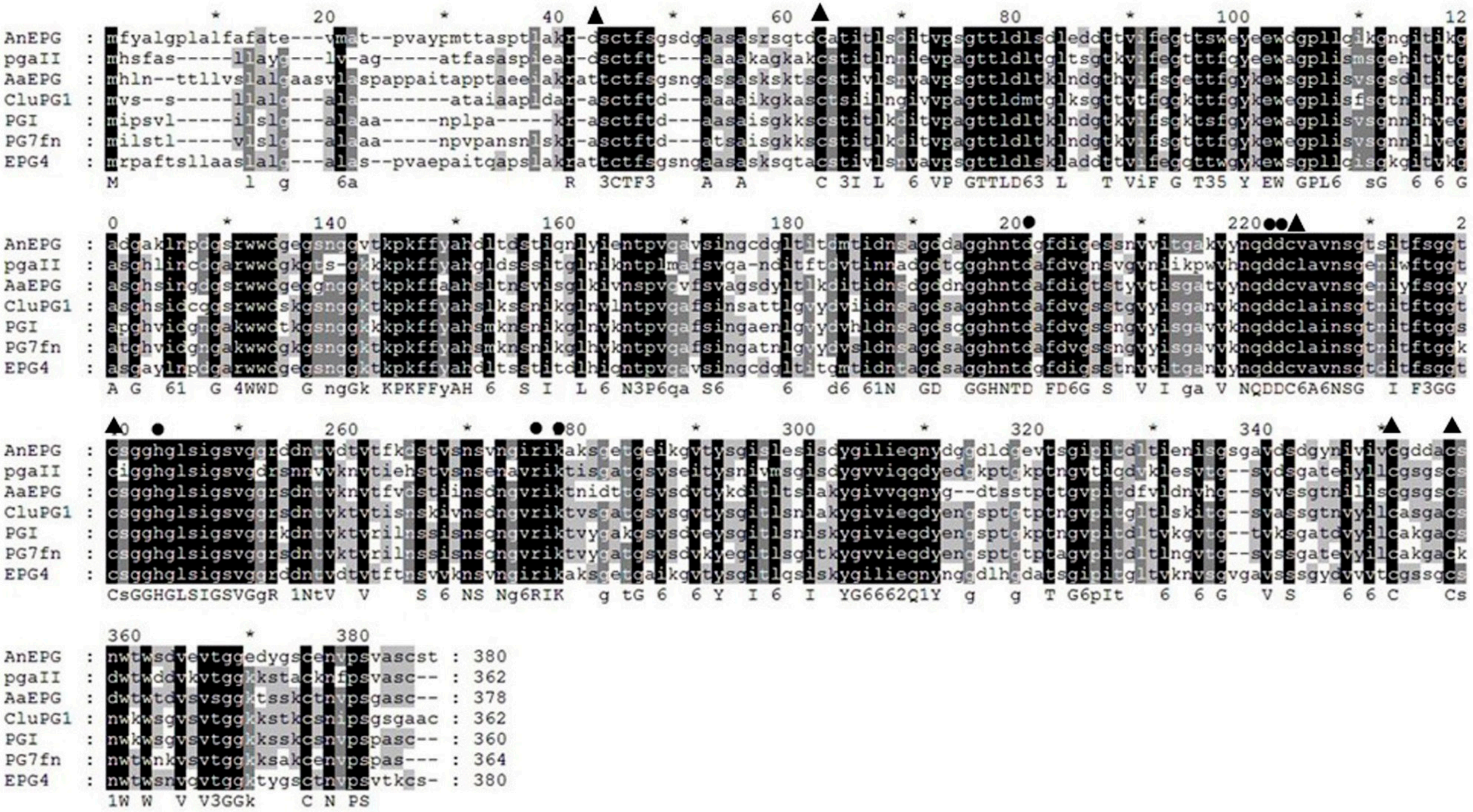

**Figure S1.** Multiple amino acid sequence alignment of GH 28 endo-PGs. The homologous sequences were found using BLASTp at NCBI. Sequences included in the alignment were derived from *Aspergillus niger* (pgaII, Genbank accession no. CAA41694) [31], *Aspergillus aculeatus* (AaEPG, Genbank accession no. AC23565) [15], *Colletotrichum lupini var. setosum* (CluPG1, Genbank accession no ABL01533) [33], *Achaetomium* sp. Xz8 (PG I, Genbank accession no. AGR51994)

[22], *Thielavia arenaria* XZ7 (endo-PG7fn, Genbank accession no. AIZ95162) [24], *Penicillium oxalicum* CZ1028 (EPG4, Genbank accession no. APZ75903.1) [17], *Aspergillus mulundensis* (hypothetical protein, Genbank accession no. XP_026600476.1), *Aspergillus calidoustus* (putative glycoside hydrolase family 28, Genbank accession no. CEN62944.1), *Penicillium antarcticum* (hypothetical protein, OQD89725.1), but only some representatives are displayed here. Putative conserved residues involved in enzyme catalysis and substrate binding were marked with black dots. Highly conserved disulfide bonds were marked with black triangles.

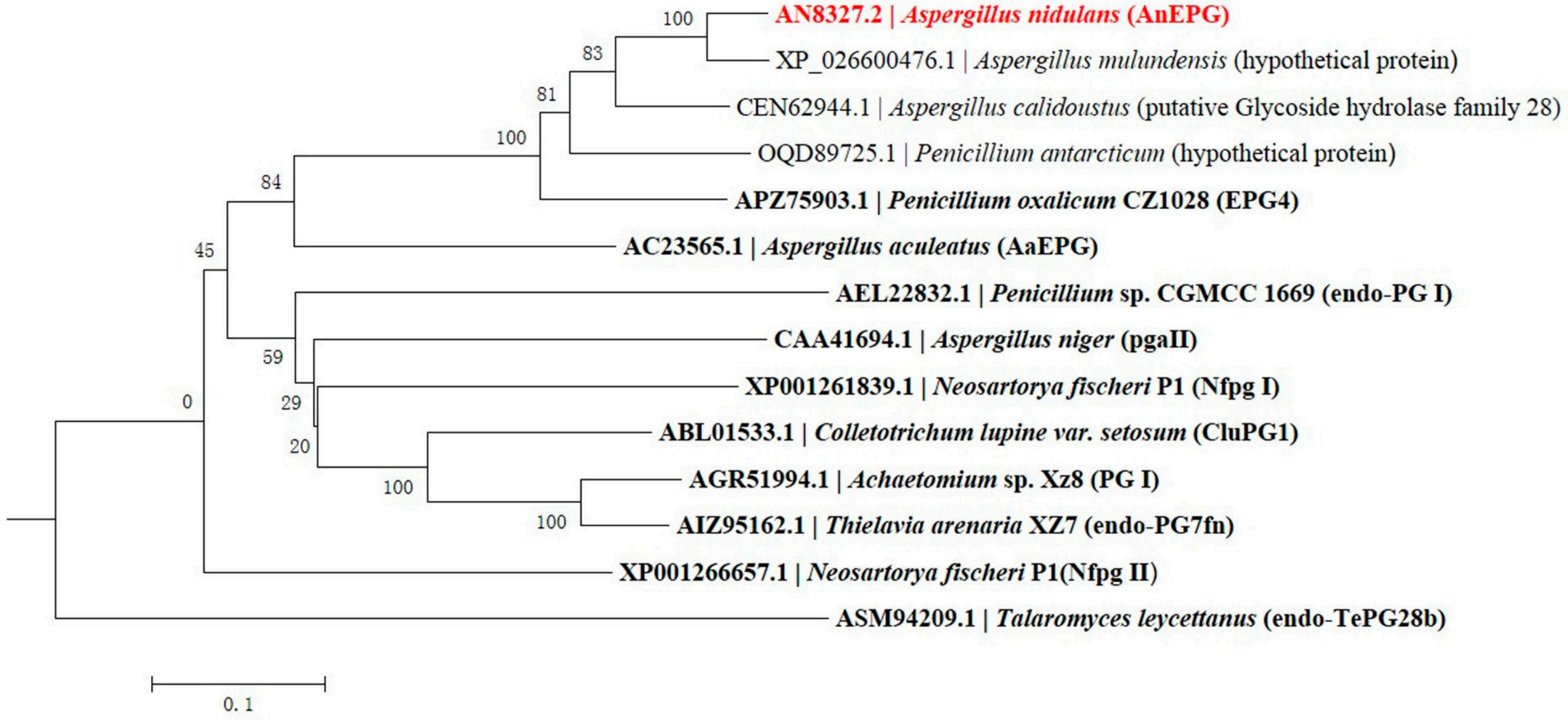


**Figure S2.** Phylogenetic analysis of GH 28 endo-PGs. The homologous sequences were found using BLASTp at NCBI. Sequences included in the alignment were derived from *Aspergillus niger* (pgaII, Genbank accession no. CAA41694) [31], *Aspergillus aculeatus* (AaEPG, Genbank accession no. AC23565) [15], *Colletotrichum lupini var. setosum* (CluPG1, Genbank accession no ABL01533) [33], *Achaetomium* sp. Xz8 (PG I, Genbank accession no. AGR51994) [22], *Thielavia*

*arenaria* XZ7 (endo-PG7fn, Genbank accession no. AIZ95162) [24], *Penicillium oxalicum* CZ1028 (EPG4, Genbank accession no. APZ75903.1) [17], *Aspergillus mulundensis* (hypothetical protein, Genbank accession no. XP_026600476.1), *Aspergillus calidoustus* (putative glycoside hydrolase family 28, Genbank accession no. CEN62944.1), *Penicillium antarcticum* (hypothetical protein, OQD89725.1), but only some representatives are displayed here. AnEPG was highlighted in bold and red. Characterized GH 28 endo-PGs were marked in bold.

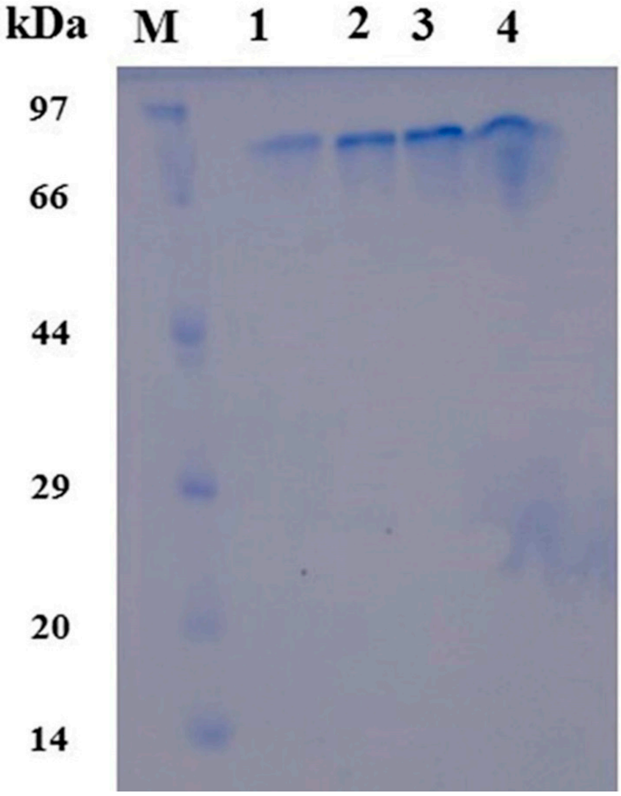


**Figure S3.** SDS-PAGE analysis of overexpressed AnEPG over induction time. M, protein marker; Lane 1, induced with 0.5% methanol for 24 h; Lane 2 induced with 0.5% methanol for 48 h; Lane 3, induced with 0.5% methanol for 72 h; Lane 4, induced with 0.5% methanol for 96 h.

**Table S1.** Comparison of effects of divalent metal ions on different GH 28 endo-PGs. ND, not determined.

| | $Ni^{2+}$ | $Ba^{2+}$ | $Ca^{2+}$ | $Cu^{2+}$ | $Mn^{2+}$ | $Zn^{2+}$ | $Co^{2+}$ | $Mg^{2+}$ | $Fe^{2+}$ | $Pb^{2+}$ | Ref |
|---|---|---|---|---|---|---|---|---|---|---|---|
| AnEPG | ↑ 9.4% | ↑ 12.2% | ↓ 14.8% | ↓ 12.8% | ↓ 10.2% | No effect | No effect | No effect | No effect | No effect | This study |
| endo-PG I | ↓ partial | ND | ↓ strong | ↓ strong | ↓ partial | ↓ strong | ↓ partial | No effect | ND | ↓ strong | [18] |
| Nfpg II | ↓ 70.2% | ND | ↓ 9.0% | ↓ 60.6% | No effect | ↓ 6.8% | ↑ 42.6% | ↓ 18.7% | ND | ↑ 8.6% | [20] |
| PGA-ZJ5A | ↓ 26.7% | ND | ↓ 46.8% | ND | ↓ 10.5% | ↓ 29.5% | ↓ 8.4% | ↓ 11.5% | No effect | ↓ 64.8% | [12] |
| endo-PGA1 | No effect | ND | ↓ 43.8% | ↓ 77.8% | ↑ 41.2% | ↓ 31.4% | No effect | No effect | ND | ↓ 85.5% | [21] |
| PG7fn | No effect | ND | ↓ partial | No effect | No effect | No effect | No effect | No effect | ND | No effect | [24] |
| PG I | No effect | ND | ↓ strong | ↓ <40% | ND | No effect | No effect | ↓ <40% | ND | No effect | [22] |
| AaEPG | ND | ND | No effect | ↑ 12.7% | ↓ 26.2% | ↓ 11.2% | ND | ↓ 4.4% | ↑ 12.0% | ND | [15] |
| PG2 | ND | ↓ 57.5% | ↓ 3.1% | ↓ 96.4% | ↓ 100% | ND | ↓ 80.7% | ↑ 32.9% | ↓ 13.4% | ND | [19] |

**Coordinates of modelled structure of AnEPG without the 36 (predicted signal peptide by Signal P 4.0) initial N-terminal amino acids**

| | | | | | | | | | | |
|---|---|---|---|---|---|---|---|---|---|---|
| ATOM | 1 | N | ASP | A | 1 | 96.198 | 61.001 | 59.909 | 1.00 | 4.06 |
| ATOM | 2 | CA | ASP | A | 1 | 96.401 | 59.914 | 58.945 | 1.00 | 4.06 |
| ATOM | 3 | HA | ASP | A | 1 | 96.887 | 60.352 | 58.074 | 1.00 | 4.06 |
| ATOM | 4 | CB | ASP | A | 1 | 97.385 | 58.879 | 59.522 | 1.00 | 4.06 |
| ATOM | 5 | HB1 | ASP | A | 1 | 96.857 | 58.198 | 60.191 | 1.00 | 4.06 |
| ATOM | 6 | HB2 | ASP | A | 1 | 98.155 | 59.394 | 60.100 | 1.00 | 4.06 |
| ATOM | 7 | CG | ASP | A | 1 | 98.079 | 58.098 | 58.400 | 1.00 | 4.06 |
| ATOM | 8 | OD1 | ASP | A | 1 | 97.566 | 57.008 | 58.058 | 1.00 | 4.06 |
| ATOM | 9 | OD2 | ASP | A | 1 | 99.069 | 58.619 | 57.842 | 1.00 | 4.06 |
| ATOM | 10 | C | ASP | A | 1 | 95.057 | 59.337 | 58.456 | 1.00 | 4.06 |
| ATOM | 11 | O | ASP | A | 1 | 94.027 | 60.014 | 58.510 | 1.00 | 4.06 |
| ATOM | 12 | N | SER | A | 2 | 95.010 | 58.117 | 57.918 | 1.00 | 3.44 |
| ATOM | 13 | H | SER | A | 2 | 95.862 | 57.553 | 57.928 | 1.00 | 3.44 |
| ATOM | 14 | CA | SER | A | 2 | 93.782 | 57.499 | 57.410 | 1.00 | 3.44 |
| ATOM | 15 | HA | SER | A | 2 | 92.936 | 58.003 | 57.873 | 1.00 | 3.44 |
| ATOM | 16 | CB | SER | A | 2 | 93.615 | 57.725 | 55.914 | 1.00 | 3.44 |
| ATOM | 17 | HB1 | SER | A | 2 | 92.848 | 57.058 | 55.517 | 1.00 | 3.44 |
| ATOM | 18 | HB2 | SER | A | 2 | 94.560 | 57.553 | 55.397 | 1.00 | 3.44 |
| ATOM | 19 | OG | SER | A | 2 | 93.188 | 59.070 | 55.764 | 1.00 | 3.44 |
| ATOM | 20 | HG | SER | A | 2 | 93.685 | 59.584 | 56.423 | 1.00 | 3.44 |
| ATOM | 21 | C | SER | A | 2 | 93.634 | 56.048 | 57.838 | 1.00 | 3.44 |
| ATOM | 22 | O | SER | A | 2 | 94.000 | 55.095 | 57.152 | 1.00 | 3.44 |
| ATOM | 23 | N | CYS | A | 3 | 93.043 | 55.912 | 59.019 | 1.00 | 2.63 |
| ATOM | 24 | H | CYS | A | 3 | 92.739 | 56.757 | 59.485 | 1.00 | 2.63 |
| ATOM | 25 | CA | CYS | A | 3 | 92.693 | 54.654 | 59.644 | 1.00 | 2.63 |
| ATOM | 26 | HA | CYS | A | 3 | 93.595 | 54.045 | 59.731 | 1.00 | 2.63 |
| ATOM | 27 | CB | CYS | A | 3 | 92.184 | 54.985 | 61.043 | 1.00 | 2.63 |
| ATOM | 28 | HB1 | CYS | A | 3 | 91.882 | 54.066 | 61.539 | 1.00 | 2.63 |
| ATOM | 29 | HB2 | CYS | A | 3 | 91.314 | 55.636 | 60.955 | 1.00 | 2.63 |
| ATOM | 30 | SG | CYS | A | 3 | 93.419 | 55.818 | 62.070 | 1.00 | 2.63 |
| ATOM | 31 | C | CYS | A | 3 | 91.646 | 53.877 | 58.843 | 1.00 | 2.63 |
| ATOM | 32 | O | CYS | A | 3 | 90.697 | 54.466 | 58.334 | 1.00 | 2.63 |
| ATOM | 33 | N | THR | A | 4 | 91.723 | 52.545 | 58.846 | 1.00 | 2.06 |
| ATOM | 34 | H | THR | A | 4 | 92.519 | 52.100 | 59.282 | 1.00 | 2.06 |
| ATOM | 35 | CA | THR | A | 4 | 90.562 | 51.710 | 58.517 | 1.00 | 2.06 |
| ATOM | 36 | HA | THR | A | 4 | 89.677 | 52.339 | 58.584 | 1.00 | 2.06 |
| ATOM | 37 | CB | THR | A | 4 | 90.570 | 51.162 | 57.080 | 1.00 | 2.06 |
| ATOM | 38 | HB | THR | A | 4 | 90.699 | 52.003 | 56.398 | 1.00 | 2.06 |
| ATOM | 39 | CG2 | THR | A | 4 | 91.664 | 50.136 | 56.789 | 1.00 | 2.06 |
| ATOM | 40 | 1HG2 | THR | A | 4 | 91.646 | 49.879 | 55.730 | 1.00 | 2.06 |

| | | | | | | | | | | |
|---|---|---|---|---|---|---|---|---|---|---|
| ATOM | 41 | 2HG2 | THR | A | 4 | 92.639 | 50.557 | 57.031 | 1.00 | 2.06 |
| ATOM | 42 | 3HG2 | THR | A | 4 | 91.503 | 49.233 | 57.378 | 1.00 | 2.06 |
| ATOM | 43 | OG1 | THR | A | 4 | 89.321 | 50.552 | 56.801 | 1.00 | 2.06 |
| ATOM | 44 | HG1 | THR | A | 4 | 89.291 | 50.387 | 55.849 | 1.00 | 2.06 |
| ATOM | 45 | C | THR | A | 4 | 90.363 | 50.626 | 59.564 | 1.00 | 2.06 |
| ATOM | 46 | O | THR | A | 4 | 91.311 | 49.951 | 59.967 | 1.00 | 2.06 |
| ATOM | 47 | N | PHE | A | 5 | 89.129 | 50.504 | 60.050 | 1.00 | 1.75 |
| ATOM | 48 | H | PHE | A | 5 | 88.387 | 51.085 | 59.668 | 1.00 | 1.75 |
| ATOM | 49 | CA | PHE | A | 5 | 88.797 | 49.656 | 61.183 | 1.00 | 1.75 |
| ATOM | 50 | HA | PHE | A | 5 | 89.622 | 48.970 | 61.368 | 1.00 | 1.75 |
| ATOM | 51 | CB | PHE | A | 5 | 88.580 | 50.491 | 62.455 | 1.00 | 1.75 |
| ATOM | 52 | HB1 | PHE | A | 5 | 88.250 | 49.823 | 63.251 | 1.00 | 1.75 |
| ATOM | 53 | HB2 | PHE | A | 5 | 87.771 | 51.199 | 62.271 | 1.00 | 1.75 |
| ATOM | 54 | CG | PHE | A | 5 | 89.801 | 51.239 | 62.964 | 1.00 | 1.75 |
| ATOM | 55 | CD1 | PHE | A | 5 | 91.040 | 50.582 | 63.112 | 1.00 | 1.75 |
| ATOM | 56 | HD1 | PHE | A | 5 | 91.145 | 49.542 | 62.845 | 1.00 | 1.75 |
| ATOM | 57 | CE1 | PHE | A | 5 | 92.166 | 51.283 | 63.576 | 1.00 | 1.75 |
| ATOM | 58 | HE1 | PHE | A | 5 | 93.125 | 50.788 | 63.655 | 1.00 | 1.75 |
| ATOM | 59 | CZ | PHE | A | 5 | 92.053 | 52.638 | 63.922 | 1.00 | 1.75 |
| ATOM | 60 | HZ | PHE | A | 5 | 92.925 | 53.183 | 64.256 | 1.00 | 1.75 |
| ATOM | 61 | CE2 | PHE | A | 5 | 90.814 | 53.290 | 63.807 | 1.00 | 1.75 |
| ATOM | 62 | HE2 | PHE | A | 5 | 90.736 | 54.333 | 64.079 | 1.00 | 1.75 |
| ATOM | 63 | CD2 | PHE | A | 5 | 89.695 | 52.598 | 63.312 | 1.00 | 1.75 |
| ATOM | 64 | HD2 | PHE | A | 5 | 88.750 | 53.112 | 63.210 | 1.00 | 1.75 |
| ATOM | 65 | C | PHE | A | 5 | 87.577 | 48.805 | 60.865 | 1.00 | 1.75 |
| ATOM | 66 | O | PHE | A | 5 | 86.509 | 49.331 | 60.559 | 1.00 | 1.75 |
| ATOM | 67 | N | SER | A | 6 | 87.775 | 47.486 | 60.901 | 1.00 | 1.91 |
| ATOM | 68 | H | SER | A | 6 | 88.680 | 47.157 | 61.188 | 1.00 | 1.91 |
| ATOM | 69 | CA | SER | A | 6 | 87.013 | 46.584 | 60.033 | 1.00 | 1.91 |
| ATOM | 70 | HA | SER | A | 6 | 86.483 | 47.213 | 59.321 | 1.00 | 1.91 |
| ATOM | 71 | CB | SER | A | 6 | 87.950 | 45.745 | 59.159 | 1.00 | 1.91 |
| ATOM | 72 | HB1 | SER | A | 6 | 87.374 | 45.244 | 58.378 | 1.00 | 1.91 |
| ATOM | 73 | HB2 | SER | A | 6 | 88.468 | 45.004 | 59.769 | 1.00 | 1.91 |
| ATOM | 74 | OG | SER | A | 6 | 88.897 | 46.607 | 58.563 | 1.00 | 1.91 |
| ATOM | 75 | HG | SER | A | 6 | 88.394 | 47.294 | 58.094 | 1.00 | 1.91 |
| ATOM | 76 | C | SER | A | 6 | 85.882 | 45.857 | 60.731 | 1.00 | 1.91 |
| ATOM | 77 | O | SER | A | 6 | 85.111 | 46.578 | 61.350 | 1.00 | 1.91 |
| ATOM | 78 | N | GLY | A | 7 | 85.842 | 44.520 | 60.588 | 1.00 | 2.24 |
| ATOM | 79 | H | GLY | A | 7 | 86.334 | 44.221 | 59.766 | 1.00 | 2.24 |
| ATOM | 80 | CA | GLY | A | 7 | 85.219 | 43.390 | 61.306 | 1.00 | 2.24 |
| ATOM | 81 | HA1 | GLY | A | 7 | 85.919 | 42.559 | 61.232 | 1.00 | 2.24 |
| ATOM | 82 | HA2 | GLY | A | 7 | 84.340 | 43.084 | 60.739 | 1.00 | 2.24 |
| ATOM | 83 | C | GLY | A | 7 | 84.769 | 43.487 | 62.777 | 1.00 | 2.24 |
| ATOM | 84 | O | GLY | A | 7 | 84.260 | 44.482 | 63.305 | 1.00 | 2.24 |

| | | | | | | | | | | |
|---|---|---|---|---|---|---|---|---|---|---|
| ATOM | 85 | N | SER | A | 8 | 84.900 | 42.326 | 63.432 | 1.00 | 2.33 |
| ATOM | 86 | H | SER | A | 8 | 85.181 | 41.526 | 62.886 | 1.00 | 2.33 |
| ATOM | 87 | CA | SER | A | 8 | 84.354 | 42.034 | 64.764 | 1.00 | 2.33 |
| ATOM | 88 | HA | SER | A | 8 | 83.304 | 42.283 | 64.735 | 1.00 | 2.33 |
| ATOM | 89 | CB | SER | A | 8 | 84.465 | 40.542 | 65.088 | 1.00 | 2.33 |
| ATOM | 90 | HB1 | SER | A | 8 | 84.004 | 40.349 | 66.058 | 1.00 | 2.33 |
| ATOM | 91 | HB2 | SER | A | 8 | 85.516 | 40.252 | 65.124 | 1.00 | 2.33 |
| ATOM | 92 | OG | SER | A | 8 | 83.793 | 39.799 | 64.090 | 1.00 | 2.33 |
| ATOM | 93 | HG | SER | A | 8 | 83.732 | 38.880 | 64.367 | 1.00 | 2.33 |
| ATOM | 94 | C | SER | A | 8 | 84.936 | 42.839 | 65.926 | 1.00 | 2.33 |
| ATOM | 95 | O | SER | A | 8 | 84.200 | 43.159 | 66.856 | 1.00 | 2.33 |
| ATOM | 96 | N | ASP | A | 9 | 86.218 | 43.201 | 65.858 | 1.00 | 1.93 |
| ATOM | 97 | H | ASP | A | 9 | 86.749 | 42.917 | 65.052 | 1.00 | 1.93 |
| ATOM | 98 | CA | ASP | A | 9 | 86.863 | 44.118 | 66.810 | 1.00 | 1.93 |
| ATOM | 99 | HA | ASP | A | 9 | 86.397 | 44.025 | 67.793 | 1.00 | 1.93 |
| ATOM | 100 | CB | ASP | A | 9 | 88.359 | 43.754 | 66.946 | 1.00 | 1.93 |
| ATOM | 101 | HB1 | ASP | A | 9 | 88.825 | 43.801 | 65.959 | 1.00 | 1.93 |
| ATOM | 102 | HB2 | ASP | A | 9 | 88.439 | 42.726 | 67.304 | 1.00 | 1.93 |
| ATOM | 103 | CG | ASP | A | 9 | 89.135 | 44.675 | 67.912 | 1.00 | 1.93 |
| ATOM | 104 | OD1 | ASP | A | 9 | 88.919 | 44.584 | 69.139 | 1.00 | 1.93 |
| ATOM | 105 | OD2 | ASP | A | 9 | 89.961 | 45.505 | 67.472 | 1.00 | 1.93 |
| ATOM | 106 | C | ASP | A | 9 | 86.760 | 45.594 | 66.379 | 1.00 | 1.93 |
| ATOM | 107 | O | ASP | A | 9 | 87.141 | 46.502 | 67.129 | 1.00 | 1.93 |
| ATOM | 108 | N | GLY | A | 10 | 86.262 | 45.859 | 65.162 | 1.00 | 1.36 |
| ATOM | 109 | H | GLY | A | 10 | 85.820 | 45.127 | 64.619 | 1.00 | 1.36 |
| ATOM | 110 | CA | GLY | A | 10 | 86.441 | 47.158 | 64.523 | 1.00 | 1.36 |
| ATOM | 111 | HA1 | GLY | A | 10 | 85.930 | 47.149 | 63.564 | 1.00 | 1.36 |
| ATOM | 112 | HA2 | GLY | A | 10 | 87.502 | 47.333 | 64.348 | 1.00 | 1.36 |
| ATOM | 113 | C | GLY | A | 10 | 85.884 | 48.332 | 65.325 | 1.00 | 1.36 |
| ATOM | 114 | O | GLY | A | 10 | 86.473 | 49.408 | 65.319 | 1.00 | 1.36 |
| ATOM | 115 | N | ALA | A | 11 | 84.823 | 48.100 | 66.100 | 1.00 | 0.93 |
| ATOM | 116 | H | ALA | A | 11 | 84.399 | 47.187 | 66.050 | 1.00 | 0.93 |
| ATOM | 117 | CA | ALA | A | 11 | 84.283 | 49.077 | 67.041 | 1.00 | 0.93 |
| ATOM | 118 | HA | ALA | A | 11 | 84.083 | 49.996 | 66.488 | 1.00 | 0.93 |
| ATOM | 119 | CB | ALA | A | 11 | 82.944 | 48.532 | 67.559 | 1.00 | 0.93 |
| ATOM | 120 | HB1 | ALA | A | 11 | 82.478 | 49.271 | 68.214 | 1.00 | 0.93 |
| ATOM | 121 | HB2 | ALA | A | 11 | 82.275 | 48.337 | 66.719 | 1.00 | 0.93 |
| ATOM | 122 | HB3 | ALA | A | 11 | 83.102 | 47.610 | 68.119 | 1.00 | 0.93 |
| ATOM | 123 | C | ALA | A | 11 | 85.224 | 49.460 | 68.195 | 1.00 | 0.93 |
| ATOM | 124 | O | ALA | A | 11 | 85.368 | 50.637 | 68.537 | 1.00 | 0.93 |
| ATOM | 125 | N | ALA | A | 12 | 85.889 | 48.466 | 68.780 | 1.00 | 0.93 |
| ATOM | 126 | H | ALA | A | 12 | 85.838 | 47.543 | 68.366 | 1.00 | 0.93 |
| ATOM | 127 | CA | ALA | A | 12 | 86.847 | 48.671 | 69.857 | 1.00 | 0.93 |
| ATOM | 128 | HA | ALA | A | 12 | 86.405 | 49.337 | 70.600 | 1.00 | 0.93 |

| | | | | | | | | | | |
|---|---|---|---|---|---|---|---|---|---|---|
| ATOM | 129 | CB | ALA | A | 12 | 87.101 | 47.324 | 70.530 | 1.00 | 0.93 |
| ATOM | 130 | HB1 | ALA | A | 12 | 87.747 | 47.471 | 71.395 | 1.00 | 0.93 |
| ATOM | 131 | HB2 | ALA | A | 12 | 86.158 | 46.894 | 70.870 | 1.00 | 0.93 |
| ATOM | 132 | HB3 | ALA | A | 12 | 87.571 | 46.639 | 69.826 | 1.00 | 0.93 |
| ATOM | 133 | C | ALA | A | 12 | 88.119 | 49.349 | 69.334 | 1.00 | 0.93 |
| ATOM | 134 | O | ALA | A | 12 | 88.578 | 50.310 | 69.944 | 1.00 | 0.93 |
| ATOM | 135 | N | SER | A | 13 | 88.664 | 48.936 | 68.183 | 1.00 | 0.86 |
| ATOM | 136 | H | SER | A | 13 | 88.296 | 48.107 | 67.723 | 1.00 | 0.86 |
| ATOM | 137 | CA | SER | A | 13 | 89.823 | 49.656 | 67.621 | 1.00 | 0.86 |
| ATOM | 138 | HA | SER | A | 13 | 90.591 | 49.715 | 68.385 | 1.00 | 0.86 |
| ATOM | 139 | CB | SER | A | 13 | 90.402 | 48.913 | 66.420 | 1.00 | 0.86 |
| ATOM | 140 | HB1 | SER | A | 13 | 89.793 | 49.082 | 65.531 | 1.00 | 0.86 |
| ATOM | 141 | HB2 | SER | A | 13 | 90.437 | 47.844 | 66.629 | 1.00 | 0.86 |
| ATOM | 142 | OG | SER | A | 13 | 91.721 | 49.375 | 66.215 | 1.00 | 0.86 |
| ATOM | 143 | HG | SER | A | 13 | 91.694 | 50.233 | 65.767 | 1.00 | 0.86 |
| ATOM | 144 | C | SER | A | 13 | 89.510 | 51.093 | 67.204 | 1.00 | 0.86 |
| ATOM | 145 | O | SER | A | 13 | 90.327 | 51.986 | 67.431 | 1.00 | 0.86 |
| ATOM | 146 | N | ALA | A | 14 | 88.319 | 51.329 | 66.643 | 1.00 | 0.75 |
| ATOM | 147 | H | ALA | A | 14 | 87.700 | 50.556 | 66.425 | 1.00 | 0.75 |
| ATOM | 148 | CA | ALA | A | 14 | 87.857 | 52.673 | 66.349 | 1.00 | 0.75 |
| ATOM | 149 | HA | ALA | A | 14 | 88.569 | 53.140 | 65.671 | 1.00 | 0.75 |
| ATOM | 150 | CB | ALA | A | 14 | 86.494 | 52.620 | 65.645 | 1.00 | 0.75 |
| ATOM | 151 | HB1 | ALA | A | 14 | 86.173 | 53.635 | 65.404 | 1.00 | 0.75 |
| ATOM | 152 | HB2 | ALA | A | 14 | 86.575 | 52.048 | 64.720 | 1.00 | 0.75 |
| ATOM | 153 | HB3 | ALA | A | 14 | 85.744 | 52.160 | 66.289 | 1.00 | 0.75 |
| ATOM | 154 | C | ALA | A | 14 | 87.837 | 53.527 | 67.615 | 1.00 | 0.75 |
| ATOM | 155 | O | ALA | A | 14 | 88.600 | 54.475 | 67.705 | 1.00 | 0.75 |
| ATOM | 156 | N | SER | A | 15 | 87.109 | 53.116 | 68.650 | 1.00 | 0.86 |
| ATOM | 157 | H | SER | A | 15 | 86.533 | 52.290 | 68.547 | 1.00 | 0.86 |
| ATOM | 158 | CA | SER | A | 15 | 87.046 | 53.887 | 69.902 | 1.00 | 0.86 |
| ATOM | 159 | HA | SER | A | 15 | 86.741 | 54.900 | 69.636 | 1.00 | 0.86 |
| ATOM | 160 | CB | SER | A | 15 | 85.937 | 53.345 | 70.811 | 1.00 | 0.86 |
| ATOM | 161 | HB1 | SER | A | 15 | 84.970 | 53.616 | 70.385 | 1.00 | 0.86 |
| ATOM | 162 | HB2 | SER | A | 15 | 86.018 | 53.805 | 71.797 | 1.00 | 0.86 |
| ATOM | 163 | OG | SER | A | 15 | 86.004 | 51.934 | 70.929 | 1.00 | 0.86 |
| ATOM | 164 | HG | SER | A | 15 | 85.636 | 51.549 | 70.117 | 1.00 | 0.86 |
| ATOM | 165 | C | SER | A | 15 | 88.373 | 54.053 | 70.669 | 1.00 | 0.86 |
| ATOM | 166 | O | SER | A | 15 | 88.530 | 55.023 | 71.408 | 1.00 | 0.86 |
| ATOM | 167 | N | ARG | A | 16 | 89.358 | 53.165 | 70.471 | 1.00 | 0.93 |
| ATOM | 168 | H | ARG | A | 16 | 89.125 | 52.336 | 69.937 | 1.00 | 0.93 |
| ATOM | 169 | CA | ARG | A | 16 | 90.747 | 53.343 | 70.952 | 1.00 | 0.93 |
| ATOM | 170 | HA | ARG | A | 16 | 90.751 | 53.655 | 71.997 | 1.00 | 0.93 |
| ATOM | 171 | CB | ARG | A | 16 | 91.540 | 52.019 | 70.783 | 1.00 | 0.93 |
| ATOM | 172 | HB1 | ARG | A | 16 | 92.598 | 52.228 | 70.963 | 1.00 | 0.93 |

| | | | | | | | | | | |
|---|---|---|---|---|---|---|---|---|---|---|
| ATOM | 173 | HB2 | ARG | A | 16 | 91.455 | 51.729 | 69.736 | 1.00 | 0.93 |
| ATOM | 174 | CG | ARG | A | 16 | 91.177 | 50.799 | 71.654 | 1.00 | 0.93 |
| ATOM | 175 | HG1 | ARG | A | 16 | 90.100 | 50.686 | 71.708 | 1.00 | 0.93 |
| ATOM | 176 | HG2 | ARG | A | 16 | 91.537 | 50.960 | 72.670 | 1.00 | 0.93 |
| ATOM | 177 | CD | ARG | A | 16 | 91.834 | 49.524 | 71.065 | 1.00 | 0.93 |
| ATOM | 178 | HD1 | ARG | A | 16 | 92.850 | 49.447 | 71.457 | 1.00 | 0.93 |
| ATOM | 179 | HD2 | ARG | A | 16 | 91.923 | 49.636 | 69.986 | 1.00 | 0.93 |
| ATOM | 180 | NE | ARG | A | 16 | 91.096 | 48.272 | 71.365 | 1.00 | 0.93 |
| ATOM | 181 | HE | ARG | A | 16 | 90.733 | 48.181 | 72.297 | 1.00 | 0.93 |
| ATOM | 182 | CZ | ARG | A | 16 | 90.817 | 47.268 | 70.537 | 1.00 | 0.93 |
| ATOM | 183 | NH1 | ARG | A | 16 | 91.166 | 47.247 | 69.282 | 1.00 | 0.93 |
| ATOM | 184 | 1HH1 | ARG | A | 16 | 91.686 | 47.996 | 68.869 | 1.00 | 0.93 |
| ATOM | 185 | 2HH1 | ARG | A | 16 | 90.794 | 46.508 | 68.668 | 1.00 | 0.93 |
| ATOM | 186 | NH2 | ARG | A | 16 | 90.141 | 46.240 | 70.957 | 1.00 | 0.93 |
| ATOM | 187 | 1HH2 | ARG | A | 16 | 89.830 | 46.151 | 71.900 | 1.00 | 0.93 |
| ATOM | 188 | 2HH2 | ARG | A | 16 | 89.811 | 45.559 | 70.257 | 1.00 | 0.93 |
| ATOM | 189 | C | ARG | A | 16 | 91.552 | 54.383 | 70.177 | 1.00 | 0.93 |
| ATOM | 190 | O | ARG | A | 16 | 92.362 | 55.058 | 70.801 | 1.00 | 0.93 |
| ATOM | 191 | N | SER | A | 17 | 91.407 | 54.430 | 68.848 | 1.00 | 0.89 |
| ATOM | 192 | H | SER | A | 17 | 90.702 | 53.844 | 68.417 | 1.00 | 0.89 |
| ATOM | 193 | CA | SER | A | 17 | 92.357 | 55.148 | 67.975 | 1.00 | 0.89 |
| ATOM | 194 | HA | SER | A | 17 | 93.164 | 55.540 | 68.597 | 1.00 | 0.89 |
| ATOM | 195 | CB | SER | A | 17 | 93.026 | 54.245 | 66.930 | 1.00 | 0.89 |
| ATOM | 196 | HB1 | SER | A | 17 | 92.832 | 54.635 | 65.930 | 1.00 | 0.89 |
| ATOM | 197 | HB2 | SER | A | 17 | 92.620 | 53.235 | 66.996 | 1.00 | 0.89 |
| ATOM | 198 | OG | SER | A | 17 | 94.428 | 54.195 | 67.130 | 1.00 | 0.89 |
| ATOM | 199 | HG | SER | A | 17 | 94.807 | 55.108 | 66.987 | 1.00 | 0.89 |
| ATOM | 200 | C | SER | A | 17 | 91.774 | 56.386 | 67.313 | 1.00 | 0.89 |
| ATOM | 201 | O | SER | A | 17 | 92.492 | 57.132 | 66.675 | 1.00 | 0.89 |
| ATOM | 202 | N | GLN | A | 18 | 90.480 | 56.651 | 67.478 | 1.00 | 0.85 |
| ATOM | 203 | H | GLN | A | 18 | 89.922 | 55.955 | 67.957 | 1.00 | 0.85 |
| ATOM | 204 | CA | GLN | A | 18 | 89.754 | 57.721 | 66.801 | 1.00 | 0.85 |
| ATOM | 205 | HA | GLN | A | 18 | 89.688 | 57.449 | 65.747 | 1.00 | 0.85 |
| ATOM | 206 | CB | GLN | A | 18 | 88.305 | 57.799 | 67.335 | 1.00 | 0.85 |
| ATOM | 207 | HB1 | GLN | A | 18 | 87.797 | 56.885 | 67.025 | 1.00 | 0.85 |
| ATOM | 208 | HB2 | GLN | A | 18 | 87.803 | 58.627 | 66.839 | 1.00 | 0.85 |
| ATOM | 209 | CG | GLN | A | 18 | 88.130 | 57.963 | 68.864 | 1.00 | 0.85 |
| ATOM | 210 | HG1 | GLN | A | 18 | 88.480 | 58.954 | 69.147 | 1.00 | 0.85 |
| ATOM | 211 | HG2 | GLN | A | 18 | 88.728 | 57.223 | 69.394 | 1.00 | 0.85 |
| ATOM | 212 | CD | GLN | A | 18 | 86.679 | 57.807 | 69.340 | 1.00 | 0.85 |
| ATOM | 213 | OE1 | GLN | A | 18 | 85.863 | 57.158 | 68.711 | 1.00 | 0.85 |
| ATOM | 214 | NE2 | GLN | A | 18 | 86.313 | 58.402 | 70.456 | 1.00 | 0.85 |
| ATOM | 215 | 1HE2 | GLN | A | 18 | 86.937 | 59.051 | 70.898 | 1.00 | 0.85 |
| ATOM | 216 | 2HE2 | GLN | A | 18 | 85.323 | 58.443 | 70.645 | 1.00 | 0.85 |

| | | | | | | | | | | |
|---|---|---|---|---|---|---|---|---|---|---|
| ATOM | 217 | C | GLN | A | 18 | 90.510 | 59.063 | 66.825 | 1.00 | 0.85 |
| ATOM | 218 | O | GLN | A | 18 | 90.727 | 59.679 | 65.781 | 1.00 | 0.85 |
| ATOM | 219 | N | THR | A | 19 | 91.025 | 59.444 | 67.994 | 1.00 | 0.87 |
| ATOM | 220 | H | THR | A | 19 | 90.913 | 58.837 | 68.793 | 1.00 | 0.87 |
| ATOM | 221 | CA | THR | A | 19 | 91.762 | 60.691 | 68.220 | 1.00 | 0.87 |
| ATOM | 222 | HA | THR | A | 19 | 91.181 | 61.503 | 67.786 | 1.00 | 0.87 |
| ATOM | 223 | CB | THR | A | 19 | 91.882 | 60.955 | 69.735 | 1.00 | 0.87 |
| ATOM | 224 | HB | THR | A | 19 | 92.494 | 61.839 | 69.916 | 1.00 | 0.87 |
| ATOM | 225 | CG2 | THR | A | 19 | 90.524 | 61.186 | 70.399 | 1.00 | 0.87 |
| ATOM | 226 | 1HG2 | THR | A | 19 | 90.000 | 61.996 | 69.891 | 1.00 | 0.87 |
| ATOM | 227 | 2HG2 | THR | A | 19 | 89.922 | 60.281 | 70.369 | 1.00 | 0.87 |
| ATOM | 228 | 3HG2 | THR | A | 19 | 90.680 | 61.475 | 71.438 | 1.00 | 0.87 |
| ATOM | 229 | OG1 | THR | A | 19 | 92.471 | 59.834 | 70.370 | 1.00 | 0.87 |
| ATOM | 230 | HG1 | THR | A | 19 | 93.399 | 59.823 | 70.112 | 1.00 | 0.87 |
| ATOM | 231 | C | THR | A | 19 | 93.167 | 60.809 | 67.599 | 1.00 | 0.87 |
| ATOM | 232 | O | THR | A | 19 | 93.858 | 61.789 | 67.864 | 1.00 | 0.87 |
| ATOM | 233 | N | ASP | A | 20 | 93.626 | 59.849 | 66.799 | 1.00 | 0.97 |
| ATOM | 234 | H | ASP | A | 20 | 93.075 | 59.003 | 66.678 | 1.00 | 0.97 |
| ATOM | 235 | CA | ASP | A | 20 | 94.840 | 59.977 | 65.981 | 1.00 | 0.97 |
| ATOM | 236 | HA | ASP | A | 20 | 95.327 | 60.925 | 66.206 | 1.00 | 0.97 |
| ATOM | 237 | CB | ASP | A | 20 | 95.851 | 58.868 | 66.350 | 1.00 | 0.97 |
| ATOM | 238 | HB1 | ASP | A | 20 | 96.061 | 58.939 | 67.419 | 1.00 | 0.97 |
| ATOM | 239 | HB2 | ASP | A | 20 | 96.788 | 59.070 | 65.828 | 1.00 | 0.97 |
| ATOM | 240 | CG | ASP | A | 20 | 95.417 | 57.429 | 66.017 | 1.00 | 0.97 |
| ATOM | 241 | OD1 | ASP | A | 20 | 95.448 | 56.574 | 66.936 | 1.00 | 0.97 |
| ATOM | 242 | OD2 | ASP | A | 20 | 95.087 | 57.161 | 64.849 | 1.00 | 0.97 |
| ATOM | 243 | C | ASP | A | 20 | 94.538 | 60.031 | 64.472 | 1.00 | 0.97 |
| ATOM | 244 | O | ASP | A | 20 | 95.419 | 60.367 | 63.683 | 1.00 | 0.97 |
| ATOM | 245 | N | CYS | A | 21 | 93.281 | 59.784 | 64.082 | 1.00 | 0.95 |
| ATOM | 246 | H | CYS | A | 21 | 92.588 | 59.604 | 64.795 | 1.00 | 0.95 |
| ATOM | 247 | CA | CYS | A | 21 | 92.910 | 59.474 | 62.708 | 1.00 | 0.95 |
| ATOM | 248 | HA | CYS | A | 21 | 93.785 | 59.138 | 62.146 | 1.00 | 0.95 |
| ATOM | 249 | CB | CYS | A | 21 | 91.876 | 58.337 | 62.756 | 1.00 | 0.95 |
| ATOM | 250 | HB1 | CYS | A | 21 | 91.535 | 58.150 | 61.739 | 1.00 | 0.95 |
| ATOM | 251 | HB2 | CYS | A | 21 | 91.030 | 58.716 | 63.330 | 1.00 | 0.95 |
| ATOM | 252 | SG | CYS | A | 21 | 92.304 | 56.741 | 63.489 | 1.00 | 0.95 |
| ATOM | 253 | C | CYS | A | 21 | 92.252 | 60.653 | 61.984 | 1.00 | 0.95 |
| ATOM | 254 | O | CYS | A | 21 | 91.074 | 60.919 | 62.224 | 1.00 | 0.95 |
| ATOM | 255 | N | ALA | A | 22 | 92.927 | 61.331 | 61.046 | 1.00 | 0.97 |
| ATOM | 256 | H | ALA | A | 22 | 93.877 | 61.064 | 60.807 | 1.00 | 0.97 |
| ATOM | 257 | CA | ALA | A | 22 | 92.309 | 62.463 | 60.332 | 1.00 | 0.97 |
| ATOM | 258 | HA | ALA | A | 22 | 91.897 | 63.151 | 61.072 | 1.00 | 0.97 |
| ATOM | 259 | CB | ALA | A | 22 | 93.400 | 63.218 | 59.565 | 1.00 | 0.97 |
| ATOM | 260 | HB1 | ALA | A | 22 | 92.974 | 64.111 | 59.105 | 1.00 | 0.97 |

| | | | | | | | | | | |
|---|---|---|---|---|---|---|---|---|---|---|
| ATOM | 261 | HB2 | ALA | A | 22 | 94.193 | 63.514 | 60.253 | 1.00 | 0.97 |
| ATOM | 262 | HB3 | ALA | A | 22 | 93.826 | 62.584 | 58.788 | 1.00 | 0.97 |
| ATOM | 263 | C | ALA | A | 22 | 91.153 | 62.040 | 59.400 | 1.00 | 0.97 |
| ATOM | 264 | O | ALA | A | 22 | 90.162 | 62.761 | 59.210 | 1.00 | 0.97 |
| ATOM | 265 | N | THR | A | 23 | 91.221 | 60.810 | 58.893 | 1.00 | 1.09 |
| ATOM | 266 | H | THR | A | 23 | 92.088 | 60.299 | 59.032 | 1.00 | 1.09 |
| ATOM | 267 | CA | THR | A | 23 | 90.059 | 60.092 | 58.384 | 1.00 | 1.09 |
| ATOM | 268 | HA | THR | A | 23 | 89.185 | 60.632 | 58.735 | 1.00 | 1.09 |
| ATOM | 269 | CB | THR | A | 23 | 89.946 | 60.082 | 56.846 | 1.00 | 1.09 |
| ATOM | 270 | HB | THR | A | 23 | 88.884 | 60.080 | 56.614 | 1.00 | 1.09 |
| ATOM | 271 | CG2 | THR | A | 23 | 90.579 | 61.266 | 56.111 | 1.00 | 1.09 |
| ATOM | 272 | 1HG2 | THR | A | 23 | 90.366 | 61.192 | 55.045 | 1.00 | 1.09 |
| ATOM | 273 | 2HG2 | THR | A | 23 | 90.177 | 62.203 | 56.493 | 1.00 | 1.09 |
| ATOM | 274 | 3HG2 | THR | A | 23 | 91.660 | 61.268 | 56.256 | 1.00 | 1.09 |
| ATOM | 275 | OG1 | THR | A | 23 | 90.463 | 58.921 | 56.261 | 1.00 | 1.09 |
| ATOM | 276 | HG1 | THR | A | 23 | 91.429 | 59.019 | 56.187 | 1.00 | 1.09 |
| ATOM | 277 | C | THR | A | 23 | 89.966 | 58.704 | 59.010 | 1.00 | 1.09 |
| ATOM | 278 | O | THR | A | 23 | 90.961 | 58.049 | 59.319 | 1.00 | 1.09 |
| ATOM | 279 | N | ILE | A | 24 | 88.727 | 58.272 | 59.220 | 1.00 | 1.37 |
| ATOM | 280 | H | ILE | A | 24 | 87.977 | 58.925 | 59.041 | 1.00 | 1.37 |
| ATOM | 281 | CA | ILE | A | 24 | 88.357 | 57.008 | 59.840 | 1.00 | 1.37 |
| ATOM | 282 | HA | ILE | A | 24 | 89.248 | 56.393 | 59.972 | 1.00 | 1.37 |
| ATOM | 283 | CB | ILE | A | 24 | 87.690 | 57.215 | 61.227 | 1.00 | 1.37 |
| ATOM | 284 | HB | ILE | A | 24 | 86.738 | 57.727 | 61.081 | 1.00 | 1.37 |
| ATOM | 285 | CG2 | ILE | A | 24 | 87.424 | 55.835 | 61.864 | 1.00 | 1.37 |
| ATOM | 286 | 1HG2 | ILE | A | 24 | 86.889 | 55.946 | 62.805 | 1.00 | 1.37 |
| ATOM | 287 | 2HG2 | ILE | A | 24 | 86.810 | 55.220 | 61.208 | 1.00 | 1.37 |
| ATOM | 288 | 3HG2 | ILE | A | 24 | 88.368 | 55.320 | 62.049 | 1.00 | 1.37 |
| ATOM | 289 | CG1 | ILE | A | 24 | 88.538 | 58.062 | 62.197 | 1.00 | 1.37 |
| ATOM | 290 | 1HG1 | ILE | A | 24 | 88.891 | 58.963 | 61.696 | 1.00 | 1.37 |
| ATOM | 291 | 2HG1 | ILE | A | 24 | 89.399 | 57.470 | 62.498 | 1.00 | 1.37 |
| ATOM | 292 | CD1 | ILE | A | 24 | 87.794 | 58.518 | 63.460 | 1.00 | 1.37 |
| ATOM | 293 | HD1 | ILE | A | 24 | 88.433 | 59.197 | 64.025 | 1.00 | 1.37 |
| ATOM | 294 | HD2 | ILE | A | 24 | 86.879 | 59.045 | 63.185 | 1.00 | 1.37 |
| ATOM | 295 | HD3 | ILE | A | 24 | 87.550 | 57.667 | 64.094 | 1.00 | 1.37 |
| ATOM | 296 | C | ILE | A | 24 | 87.398 | 56.273 | 58.910 | 1.00 | 1.37 |
| ATOM | 297 | O | ILE | A | 24 | 86.243 | 56.668 | 58.746 | 1.00 | 1.37 |
| ATOM | 298 | N | THR | A | 25 | 87.854 | 55.190 | 58.301 | 1.00 | 1.22 |
| ATOM | 299 | H | THR | A | 25 | 88.794 | 54.859 | 58.493 | 1.00 | 1.22 |
| ATOM | 300 | CA | THR | A | 25 | 87.015 | 54.289 | 57.533 | 1.00 | 1.22 |
| ATOM | 301 | HA | THR | A | 25 | 86.161 | 54.817 | 57.117 | 1.00 | 1.22 |
| ATOM | 302 | CB | THR | A | 25 | 87.819 | 53.660 | 56.391 | 1.00 | 1.22 |
| ATOM | 303 | HB | THR | A | 25 | 88.606 | 53.037 | 56.804 | 1.00 | 1.22 |
| ATOM | 304 | CG2 | THR | A | 25 | 86.960 | 52.839 | 55.441 | 1.00 | 1.22 |

| | | | | | | | | | | |
|---|---|---|---|---|---|---|---|---|---|---|
| ATOM | 305 | 1HG2 | THR | A | 25 | 87.572 | 52.470 | 54.617 | 1.00 | 1.22 |
| ATOM | 306 | 2HG2 | THR | A | 25 | 86.542 | 51.982 | 55.962 | 1.00 | 1.22 |
| ATOM | 307 | 3HG2 | THR | A | 25 | 86.150 | 53.442 | 55.033 | 1.00 | 1.22 |
| ATOM | 308 | OG1 | THR | A | 25 | 88.408 | 54.667 | 55.613 | 1.00 | 1.22 |
| ATOM | 309 | HG1 | THR | A | 25 | 88.889 | 54.238 | 54.902 | 1.00 | 1.22 |
| ATOM | 310 | C | THR | A | 25 | 86.539 | 53.206 | 58.496 | 1.00 | 1.22 |
| ATOM | 311 | O | THR | A | 25 | 87.285 | 52.773 | 59.380 | 1.00 | 1.22 |
| ATOM | 312 | N | LEU | A | 26 | 85.289 | 52.787 | 58.337 | 1.00 | 1.04 |
| ATOM | 313 | H | LEU | A | 26 | 84.777 | 53.113 | 57.535 | 1.00 | 1.04 |
| ATOM | 314 | CA | LEU | A | 26 | 84.585 | 51.885 | 59.240 | 1.00 | 1.04 |
| ATOM | 315 | HA | LEU | A | 26 | 85.289 | 51.405 | 59.918 | 1.00 | 1.04 |
| ATOM | 316 | CB | LEU | A | 26 | 83.541 | 52.675 | 60.051 | 1.00 | 1.04 |
| ATOM | 317 | HB1 | LEU | A | 26 | 82.902 | 51.971 | 60.584 | 1.00 | 1.04 |
| ATOM | 318 | HB2 | LEU | A | 26 | 82.903 | 53.217 | 59.352 | 1.00 | 1.04 |
| ATOM | 319 | CG | LEU | A | 26 | 84.118 | 53.678 | 61.060 | 1.00 | 1.04 |
| ATOM | 320 | HG | LEU | A | 26 | 84.723 | 54.416 | 60.535 | 1.00 | 1.04 |
| ATOM | 321 | CD1 | LEU | A | 26 | 82.951 | 54.396 | 61.725 | 1.00 | 1.04 |
| ATOM | 322 | 1HD1 | LEU | A | 26 | 83.331 | 55.197 | 62.356 | 1.00 | 1.04 |
| ATOM | 323 | 2HD1 | LEU | A | 26 | 82.309 | 54.837 | 60.963 | 1.00 | 1.04 |
| ATOM | 324 | 3HD1 | LEU | A | 26 | 82.370 | 53.698 | 62.325 | 1.00 | 1.04 |
| ATOM | 325 | CD2 | LEU | A | 26 | 84.949 | 53.037 | 62.174 | 1.00 | 1.04 |
| ATOM | 326 | 1HD2 | LEU | A | 26 | 85.288 | 53.806 | 62.866 | 1.00 | 1.04 |
| ATOM | 327 | 2HD2 | LEU | A | 26 | 84.350 | 52.308 | 62.718 | 1.00 | 1.04 |
| ATOM | 328 | 3HD2 | LEU | A | 26 | 85.823 | 52.546 | 61.747 | 1.00 | 1.04 |
| ATOM | 329 | C | LEU | A | 26 | 83.911 | 50.774 | 58.405 | 1.00 | 1.04 |
| ATOM | 330 | O | LEU | A | 26 | 82.973 | 51.107 | 57.689 | 1.00 | 1.04 |
| ATOM | 331 | N | SER | A | 27 | 84.420 | 49.533 | 58.390 | 1.00 | 0.95 |
| ATOM | 332 | H | SER | A | 27 | 85.077 | 49.267 | 59.113 | 1.00 | 0.95 |
| ATOM | 333 | CA | SER | A | 27 | 84.577 | 48.801 | 57.123 | 1.00 | 0.95 |
| ATOM | 334 | HA | SER | A | 27 | 83.944 | 49.246 | 56.369 | 1.00 | 0.95 |
| ATOM | 335 | CB | SER | A | 27 | 86.006 | 48.936 | 56.554 | 1.00 | 0.95 |
| ATOM | 336 | HB1 | SER | A | 27 | 86.066 | 49.895 | 56.046 | 1.00 | 0.95 |
| ATOM | 337 | HB2 | SER | A | 27 | 86.171 | 48.166 | 55.800 | 1.00 | 0.95 |
| ATOM | 338 | OG | SER | A | 27 | 87.054 | 48.888 | 57.521 | 1.00 | 0.95 |
| ATOM | 339 | HG | SER | A | 27 | 87.753 | 49.493 | 57.224 | 1.00 | 0.95 |
| ATOM | 340 | C | SER | A | 27 | 84.149 | 47.336 | 57.269 | 1.00 | 0.95 |
| ATOM | 341 | O | SER | A | 27 | 84.960 | 46.471 | 57.603 | 1.00 | 0.95 |
| ATOM | 342 | N | ASP | A | 28 | 82.852 | 47.077 | 57.068 | 1.00 | 0.49 |
| ATOM | 343 | H | ASP | A | 28 | 82.277 | 47.847 | 56.740 | 1.00 | 0.49 |
| ATOM | 344 | CA | ASP | A | 28 | 82.168 | 45.851 | 57.503 | 1.00 | 0.49 |
| ATOM | 345 | HA | ASP | A | 28 | 81.106 | 45.974 | 57.379 | 1.00 | 0.49 |
| ATOM | 346 | CB | ASP | A | 28 | 82.510 | 44.639 | 56.593 | 1.00 | 0.49 |
| ATOM | 347 | HB1 | ASP | A | 28 | 82.383 | 43.721 | 57.166 | 1.00 | 0.49 |
| ATOM | 348 | HB2 | ASP | A | 28 | 83.554 | 44.682 | 56.279 | 1.00 | 0.49 |

| | | | | | | | | | | |
|---|---|---|---|---|---|---|---|---|---|---|
| ATOM | 349 | CG | ASP | A | 28 | 81.590 | 44.523 | 55.370 | 1.00 | 0.49 |
| ATOM | 350 | OD1 | ASP | A | 28 | 82.059 | 44.668 | 54.222 | 1.00 | 0.49 |
| ATOM | 351 | OD2 | ASP | A | 28 | 80.368 | 44.300 | 55.576 | 1.00 | 0.49 |
| ATOM | 352 | C | ASP | A | 28 | 82.383 | 45.621 | 59.006 | 1.00 | 0.49 |
| ATOM | 353 | O | ASP | A | 28 | 82.997 | 44.639 | 59.443 | 1.00 | 0.49 |
| ATOM | 354 | N | ILE | A | 29 | 81.905 | 46.579 | 59.806 | 1.00 | 0.42 |
| ATOM | 355 | H | ILE | A | 29 | 81.360 | 47.314 | 59.387 | 1.00 | 0.42 |
| ATOM | 356 | CA | ILE | A | 29 | 81.951 | 46.451 | 61.276 | 1.00 | 0.42 |
| ATOM | 357 | HA | ILE | A | 29 | 82.915 | 46.079 | 61.579 | 1.00 | 0.42 |
| ATOM | 358 | CB | ILE | A | 29 | 81.678 | 47.803 | 62.009 | 1.00 | 0.42 |
| ATOM | 359 | HB | ILE | A | 29 | 80.614 | 47.986 | 61.900 | 1.00 | 0.42 |
| ATOM | 360 | CG2 | ILE | A | 29 | 81.998 | 47.716 | 63.521 | 1.00 | 0.42 |
| ATOM | 361 | 1HG2 | ILE | A | 29 | 83.042 | 47.443 | 63.672 | 1.00 | 0.42 |
| ATOM | 362 | 2HG2 | ILE | A | 29 | 81.783 | 48.667 | 64.005 | 1.00 | 0.42 |
| ATOM | 363 | 3HG2 | ILE | A | 29 | 81.395 | 46.969 | 64.016 | 1.00 | 0.42 |
| ATOM | 364 | CG1 | ILE | A | 29 | 82.388 | 49.059 | 61.419 | 1.00 | 0.42 |
| ATOM | 365 | 1HG1 | ILE | A | 29 | 81.672 | 49.884 | 61.481 | 1.00 | 0.42 |
| ATOM | 366 | 2HG1 | ILE | A | 29 | 82.582 | 48.910 | 60.357 | 1.00 | 0.42 |
| ATOM | 367 | CD1 | ILE | A | 29 | 83.677 | 49.526 | 62.099 | 1.00 | 0.42 |
| ATOM | 368 | HD1 | ILE | A | 29 | 84.374 | 48.710 | 62.253 | 1.00 | 0.42 |
| ATOM | 369 | HD2 | ILE | A | 29 | 84.181 | 50.261 | 61.485 | 1.00 | 0.42 |
| ATOM | 370 | HD3 | ILE | A | 29 | 83.474 | 49.991 | 63.063 | 1.00 | 0.42 |
| ATOM | 371 | C | ILE | A | 29 | 80.919 | 45.369 | 61.642 | 1.00 | 0.42 |
| ATOM | 372 | O | ILE | A | 29 | 79.822 | 45.270 | 61.026 | 1.00 | 0.42 |
| ATOM | 373 | N | THR | A | 30 | 81.185 | 44.668 | 62.746 | 1.00 | 0.46 |
| ATOM | 374 | H | THR | A | 30 | 82.090 | 44.773 | 63.170 | 1.00 | 0.46 |
| ATOM | 375 | CA | THR | A | 30 | 80.077 | 44.288 | 63.632 | 1.00 | 0.46 |
| ATOM | 376 | HA | THR | A | 30 | 79.342 | 43.683 | 63.114 | 1.00 | 0.46 |
| ATOM | 377 | CB | THR | A | 30 | 80.550 | 43.547 | 64.877 | 1.00 | 0.46 |
| ATOM | 378 | HB | THR | A | 30 | 79.799 | 43.497 | 65.657 | 1.00 | 0.46 |
| ATOM | 379 | CG2 | THR | A | 30 | 80.817 | 42.067 | 64.523 | 1.00 | 0.46 |
| ATOM | 380 | 1HG2 | THR | A | 30 | 81.255 | 41.562 | 65.382 | 1.00 | 0.46 |
| ATOM | 381 | 2HG2 | THR | A | 30 | 79.865 | 41.593 | 64.288 | 1.00 | 0.46 |
| ATOM | 382 | 3HG2 | THR | A | 30 | 81.465 | 42.026 | 63.647 | 1.00 | 0.46 |
| ATOM | 383 | OG1 | THR | A | 30 | 81.719 | 44.140 | 65.413 | 1.00 | 0.46 |
| ATOM | 384 | HG1 | THR | A | 30 | 82.106 | 43.568 | 66.085 | 1.00 | 0.46 |
| ATOM | 385 | C | THR | A | 30 | 79.244 | 45.606 | 64.074 | 1.00 | 0.46 |
| ATOM | 386 | O | THR | A | 30 | 78.978 | 46.424 | 63.188 | 1.00 | 0.46 |
| ATOM | 387 | N | VAL | A | 31 | 78.669 | 45.832 | 65.269 | 1.00 | 0.51 |
| ATOM | 388 | H | VAL | A | 31 | 77.834 | 46.379 | 65.088 | 1.00 | 0.51 |
| ATOM | 389 | CA | VAL | A | 31 | 79.206 | 46.198 | 66.673 | 1.00 | 0.51 |
| ATOM | 390 | HA | VAL | A | 31 | 80.252 | 46.364 | 66.601 | 1.00 | 0.51 |
| ATOM | 391 | CB | VAL | A | 31 | 78.491 | 47.520 | 67.084 | 1.00 | 0.51 |
| ATOM | 392 | HB | VAL | A | 31 | 77.442 | 47.253 | 67.204 | 1.00 | 0.51 |

| | | | | | | | | | | |
|---|---|---|---|---|---|---|---|---|---|---|
| ATOM | 393 | CG1 | VAL | A | 31 | 79.013 | 48.089 | 68.395 | 1.00 | 0.51 |
| ATOM | 394 | 1HG1 | VAL | A | 31 | 78.508 | 49.030 | 68.612 | 1.00 | 0.51 |
| ATOM | 395 | 2HG1 | VAL | A | 31 | 78.782 | 47.427 | 69.227 | 1.00 | 0.51 |
| ATOM | 396 | 3HG1 | VAL | A | 31 | 80.086 | 48.272 | 68.359 | 1.00 | 0.51 |
| ATOM | 397 | CG2 | VAL | A | 31 | 78.582 | 48.611 | 66.020 | 1.00 | 0.51 |
| ATOM | 398 | 1HG2 | VAL | A | 31 | 77.991 | 49.465 | 66.346 | 1.00 | 0.51 |
| ATOM | 399 | 2HG2 | VAL | A | 31 | 79.621 | 48.891 | 65.847 | 1.00 | 0.51 |
| ATOM | 400 | 3HG2 | VAL | A | 31 | 78.153 | 48.305 | 65.070 | 1.00 | 0.51 |
| ATOM | 401 | C | VAL | A | 31 | 78.833 | 45.118 | 67.672 | 1.00 | 0.51 |
| ATOM | 402 | O | VAL | A | 31 | 77.774 | 44.512 | 67.501 | 1.00 | 0.51 |
| ATOM | 403 | N | PRO | A | 32 | 79.621 | 44.835 | 68.713 | 1.00 | 1.03 |
| ATOM | 404 | CD | PRO | A | 32 | 80.976 | 45.316 | 68.958 | 1.00 | 1.03 |
| ATOM | 405 | HD1 | PRO | A | 32 | 80.931 | 46.328 | 69.362 | 1.00 | 1.03 |
| ATOM | 406 | HD2 | PRO | A | 32 | 81.575 | 45.285 | 68.047 | 1.00 | 1.03 |
| ATOM | 407 | CG | PRO | A | 32 | 81.563 | 44.376 | 70.007 | 1.00 | 1.03 |
| ATOM | 408 | HG1 | PRO | A | 32 | 82.311 | 44.877 | 70.619 | 1.00 | 1.03 |
| ATOM | 409 | HG2 | PRO | A | 32 | 81.997 | 43.503 | 69.519 | 1.00 | 1.03 |
| ATOM | 410 | CB | PRO | A | 32 | 80.338 | 43.957 | 70.817 | 1.00 | 1.03 |
| ATOM | 411 | HB1 | PRO | A | 32 | 80.119 | 44.717 | 71.564 | 1.00 | 1.03 |
| ATOM | 412 | HB2 | PRO | A | 32 | 80.492 | 42.995 | 71.305 | 1.00 | 1.03 |
| ATOM | 413 | CA | PRO | A | 32 | 79.214 | 43.909 | 69.781 | 1.00 | 1.03 |
| ATOM | 414 | HA | PRO | A | 32 | 79.187 | 42.906 | 69.345 | 1.00 | 1.03 |
| ATOM | 415 | C | PRO | A | 32 | 77.840 | 44.218 | 70.410 | 1.00 | 1.03 |
| ATOM | 416 | O | PRO | A | 32 | 77.422 | 45.375 | 70.566 | 1.00 | 1.03 |
| ATOM | 417 | N | SER | A | 33 | 77.171 | 43.154 | 70.862 | 1.00 | 1.00 |
| ATOM | 418 | H | SER | A | 33 | 77.527 | 42.229 | 70.693 | 1.00 | 1.00 |
| ATOM | 419 | CA | SER | A | 33 | 75.979 | 43.251 | 71.705 | 1.00 | 1.00 |
| ATOM | 420 | HA | SER | A | 33 | 75.137 | 43.567 | 71.103 | 1.00 | 1.00 |
| ATOM | 421 | CB | SER | A | 33 | 75.666 | 41.908 | 72.376 | 1.00 | 1.00 |
| ATOM | 422 | HB1 | SER | A | 33 | 74.918 | 42.046 | 73.158 | 1.00 | 1.00 |
| ATOM | 423 | HB2 | SER | A | 33 | 76.570 | 41.514 | 72.843 | 1.00 | 1.00 |
| ATOM | 424 | OG | SER | A | 33 | 75.165 | 40.961 | 71.458 | 1.00 | 1.00 |
| ATOM | 425 | HG | SER | A | 33 | 74.217 | 40.838 | 71.660 | 1.00 | 1.00 |
| ATOM | 426 | C | SER | A | 33 | 76.133 | 44.255 | 72.842 | 1.00 | 1.00 |
| ATOM | 427 | O | SER | A | 33 | 77.213 | 44.398 | 73.414 | 1.00 | 1.00 |
| ATOM | 428 | N | GLY | A | 34 | 75.038 | 44.947 | 73.154 | 1.00 | 0.96 |
| ATOM | 429 | H | GLY | A | 34 | 74.178 | 44.735 | 72.672 | 1.00 | 0.96 |
| ATOM | 430 | CA | GLY | A | 34 | 75.003 | 45.950 | 74.214 | 1.00 | 0.96 |
| ATOM | 431 | HA1 | GLY | A | 34 | 75.133 | 45.448 | 75.174 | 1.00 | 0.96 |
| ATOM | 432 | HA2 | GLY | A | 34 | 74.027 | 46.430 | 74.223 | 1.00 | 0.96 |
| ATOM | 433 | C | GLY | A | 34 | 76.075 | 47.031 | 74.119 | 1.00 | 0.96 |
| ATOM | 434 | O | GLY | A | 34 | 76.437 | 47.621 | 75.133 | 1.00 | 0.96 |
| ATOM | 435 | N | THR | A | 35 | 76.623 | 47.279 | 72.920 | 1.00 | 0.95 |
| ATOM | 436 | H | THR | A | 35 | 76.354 | 46.735 | 72.112 | 1.00 | 0.95 |

| | | | | | | | | | | |
|---|---|---|---|---|---|---|---|---|---|---|
| ATOM | 437 | CA | THR | A | 35 | 77.716 | 48.235 | 72.785 | 1.00 | 0.95 |
| ATOM | 438 | HA | THR | A | 35 | 77.910 | 48.730 | 73.736 | 1.00 | 0.95 |
| ATOM | 439 | CB | THR | A | 35 | 78.999 | 47.481 | 72.400 | 1.00 | 0.95 |
| ATOM | 440 | HB | THR | A | 35 | 78.885 | 47.048 | 71.412 | 1.00 | 0.95 |
| ATOM | 441 | CG2 | THR | A | 35 | 80.246 | 48.359 | 72.378 | 1.00 | 0.95 |
| ATOM | 442 | 1HG2 | THR | A | 35 | 81.127 | 47.733 | 72.248 | 1.00 | 0.95 |
| ATOM | 443 | 2HG2 | THR | A | 35 | 80.190 | 49.050 | 71.541 | 1.00 | 0.95 |
| ATOM | 444 | 3HG2 | THR | A | 35 | 80.330 | 48.911 | 73.315 | 1.00 | 0.95 |
| ATOM | 445 | OG1 | THR | A | 35 | 79.283 | 46.448 | 73.313 | 1.00 | 0.95 |
| ATOM | 446 | HG1 | THR | A | 35 | 78.505 | 45.868 | 73.366 | 1.00 | 0.95 |
| ATOM | 447 | C | THR | A | 35 | 77.402 | 49.310 | 71.754 | 1.00 | 0.95 |
| ATOM | 448 | O | THR | A | 35 | 76.859 | 49.047 | 70.680 | 1.00 | 0.95 |
| ATOM | 449 | N | THR | A | 36 | 77.772 | 50.541 | 72.093 | 1.00 | 0.67 |
| ATOM | 450 | H | THR | A | 36 | 78.147 | 50.698 | 73.016 | 1.00 | 0.67 |
| ATOM | 451 | CA | THR | A | 36 | 77.796 | 51.676 | 71.177 | 1.00 | 0.67 |
| ATOM | 452 | HA | THR | A | 36 | 76.917 | 51.644 | 70.534 | 1.00 | 0.67 |
| ATOM | 453 | CB | THR | A | 36 | 77.780 | 52.990 | 71.979 | 1.00 | 0.67 |
| ATOM | 454 | HB | THR | A | 36 | 78.663 | 53.041 | 72.617 | 1.00 | 0.67 |
| ATOM | 455 | CG2 | THR | A | 36 | 77.701 | 54.249 | 71.120 | 1.00 | 0.67 |
| ATOM | 456 | 1HG2 | THR | A | 36 | 77.476 | 55.102 | 71.759 | 1.00 | 0.67 |
| ATOM | 457 | 2HG2 | THR | A | 36 | 78.665 | 54.435 | 70.660 | 1.00 | 0.67 |
| ATOM | 458 | 3HG2 | THR | A | 36 | 76.937 | 54.149 | 70.350 | 1.00 | 0.67 |
| ATOM | 459 | OG1 | THR | A | 36 | 76.634 | 53.007 | 72.796 | 1.00 | 0.67 |
| ATOM | 460 | HG1 | THR | A | 36 | 75.867 | 53.036 | 72.194 | 1.00 | 0.67 |
| ATOM | 461 | C | THR | A | 36 | 79.048 | 51.631 | 70.311 | 1.00 | 0.67 |
| ATOM | 462 | O | THR | A | 36 | 80.157 | 51.451 | 70.811 | 1.00 | 0.67 |
| ATOM | 463 | N | LEU | A | 37 | 78.902 | 51.841 | 69.005 | 1.00 | 0.42 |
| ATOM | 464 | H | LEU | A | 37 | 77.959 | 51.897 | 68.635 | 1.00 | 0.42 |
| ATOM | 465 | CA | LEU | A | 37 | 79.980 | 52.330 | 68.154 | 1.00 | 0.42 |
| ATOM | 466 | HA | LEU | A | 37 | 80.892 | 51.779 | 68.391 | 1.00 | 0.42 |
| ATOM | 467 | CB | LEU | A | 37 | 79.614 | 52.058 | 66.691 | 1.00 | 0.42 |
| ATOM | 468 | HB1 | LEU | A | 37 | 78.667 | 52.549 | 66.461 | 1.00 | 0.42 |
| ATOM | 469 | HB2 | LEU | A | 37 | 79.472 | 50.992 | 66.605 | 1.00 | 0.42 |
| ATOM | 470 | CG | LEU | A | 37 | 80.655 | 52.466 | 65.639 | 1.00 | 0.42 |
| ATOM | 471 | HG | LEU | A | 37 | 80.740 | 53.545 | 65.648 | 1.00 | 0.42 |
| ATOM | 472 | CD1 | LEU | A | 37 | 82.036 | 51.844 | 65.857 | 1.00 | 0.42 |
| ATOM | 473 | 1HD1 | LEU | A | 37 | 82.703 | 52.141 | 65.048 | 1.00 | 0.42 |
| ATOM | 474 | 2HD1 | LEU | A | 37 | 82.465 | 52.210 | 66.791 | 1.00 | 0.42 |
| ATOM | 475 | 3HD1 | LEU | A | 37 | 81.957 | 50.759 | 65.882 | 1.00 | 0.42 |
| ATOM | 476 | CD2 | LEU | A | 37 | 80.164 | 52.023 | 64.259 | 1.00 | 0.42 |
| ATOM | 477 | 1HD2 | LEU | A | 37 | 80.866 | 52.354 | 63.497 | 1.00 | 0.42 |
| ATOM | 478 | 2HD2 | LEU | A | 37 | 80.073 | 50.937 | 64.214 | 1.00 | 0.42 |
| ATOM | 479 | 3HD2 | LEU | A | 37 | 79.193 | 52.473 | 64.055 | 1.00 | 0.42 |
| ATOM | 480 | C | LEU | A | 37 | 80.198 | 53.802 | 68.486 | 1.00 | 0.42 |

| | | | | | | | | | | |
|---|---|---|---|---|---|---|---|---|---|---|
| ATOM | 481 | O | LEU | A | 37 | 79.554 | 54.714 | 67.954 | 1.00 | 0.42 |
| ATOM | 482 | N | ASP | A | 38 | 81.049 | 54.000 | 69.482 | 1.00 | 0.45 |
| ATOM | 483 | H | ASP | A | 38 | 81.398 | 53.201 | 69.997 | 1.00 | 0.45 |
| ATOM | 484 | CA | ASP | A | 38 | 81.495 | 55.316 | 69.884 | 1.00 | 0.45 |
| ATOM | 485 | HA | ASP | A | 38 | 80.619 | 55.966 | 69.936 | 1.00 | 0.45 |
| ATOM | 486 | CB | ASP | A | 38 | 82.111 | 55.263 | 71.289 | 1.00 | 0.45 |
| ATOM | 487 | HB1 | ASP | A | 38 | 83.013 | 55.876 | 71.319 | 1.00 | 0.45 |
| ATOM | 488 | HB2 | ASP | A | 38 | 82.383 | 54.240 | 71.560 | 1.00 | 0.45 |
| ATOM | 489 | CG | ASP | A | 38 | 81.099 | 55.832 | 72.279 | 1.00 | 0.45 |
| ATOM | 490 | OD1 | ASP | A | 38 | 81.024 | 57.084 | 72.335 | 1.00 | 0.45 |
| ATOM | 491 | OD2 | ASP | A | 38 | 80.341 | 55.062 | 72.901 | 1.00 | 0.45 |
| ATOM | 492 | C | ASP | A | 38 | 82.421 | 55.909 | 68.817 | 1.00 | 0.45 |
| ATOM | 493 | O | ASP | A | 38 | 83.511 | 55.401 | 68.569 | 1.00 | 0.45 |
| ATOM | 494 | N | LEU | A | 39 | 81.931 | 56.977 | 68.181 | 1.00 | 0.55 |
| ATOM | 495 | H | LEU | A | 39 | 80.957 | 57.201 | 68.361 | 1.00 | 0.55 |
| ATOM | 496 | CA | LEU | A | 39 | 82.678 | 58.014 | 67.458 | 1.00 | 0.55 |
| ATOM | 497 | HA | LEU | A | 39 | 83.741 | 57.895 | 67.665 | 1.00 | 0.55 |
| ATOM | 498 | CB | LEU | A | 39 | 82.446 | 57.913 | 65.931 | 1.00 | 0.55 |
| ATOM | 499 | HB1 | LEU | A | 39 | 82.787 | 58.851 | 65.504 | 1.00 | 0.55 |
| ATOM | 500 | HB2 | LEU | A | 39 | 81.371 | 57.862 | 65.753 | 1.00 | 0.55 |
| ATOM | 501 | CG | LEU | A | 39 | 83.140 | 56.808 | 65.098 | 1.00 | 0.55 |
| ATOM | 502 | HG | LEU | A | 39 | 83.060 | 57.112 | 64.053 | 1.00 | 0.55 |
| ATOM | 503 | CD1 | LEU | A | 39 | 84.628 | 56.576 | 65.376 | 1.00 | 0.55 |
| ATOM | 504 | 1HD1 | LEU | A | 39 | 85.018 | 55.812 | 64.706 | 1.00 | 0.55 |
| ATOM | 505 | 2HD1 | LEU | A | 39 | 85.185 | 57.495 | 65.213 | 1.00 | 0.55 |
| ATOM | 506 | 3HD1 | LEU | A | 39 | 84.774 | 56.249 | 66.407 | 1.00 | 0.55 |
| ATOM | 507 | CD2 | LEU | A | 39 | 82.425 | 55.469 | 65.210 | 1.00 | 0.55 |
| ATOM | 508 | 1HD2 | LEU | A | 39 | 81.385 | 55.591 | 64.915 | 1.00 | 0.55 |
| ATOM | 509 | 2HD2 | LEU | A | 39 | 82.903 | 54.728 | 64.574 | 1.00 | 0.55 |
| ATOM | 510 | 3HD2 | LEU | A | 39 | 82.471 | 55.112 | 66.235 | 1.00 | 0.55 |
| ATOM | 511 | C | LEU | A | 39 | 82.266 | 59.398 | 68.040 | 1.00 | 0.55 |
| ATOM | 512 | O | LEU | A | 39 | 82.029 | 60.419 | 67.379 | 1.00 | 0.55 |
| ATOM | 513 | N | SER | A | 40 | 82.086 | 59.392 | 69.352 | 1.00 | 0.74 |
| ATOM | 514 | H | SER | A | 40 | 82.198 | 58.498 | 69.806 | 1.00 | 0.74 |
| ATOM | 515 | CA | SER | A | 40 | 82.316 | 60.548 | 70.217 | 1.00 | 0.74 |
| ATOM | 516 | HA | SER | A | 40 | 81.535 | 61.273 | 70.014 | 1.00 | 0.74 |
| ATOM | 517 | CB | SER | A | 40 | 82.175 | 60.081 | 71.678 | 1.00 | 0.74 |
| ATOM | 518 | HB1 | SER | A | 40 | 81.118 | 59.910 | 71.891 | 1.00 | 0.74 |
| ATOM | 519 | HB2 | SER | A | 40 | 82.544 | 60.851 | 72.357 | 1.00 | 0.74 |
| ATOM | 520 | OG | SER | A | 40 | 82.874 | 58.868 | 71.905 | 1.00 | 0.74 |
| ATOM | 521 | HG | SER | A | 40 | 82.211 | 58.181 | 72.164 | 1.00 | 0.74 |
| ATOM | 522 | C | SER | A | 40 | 83.638 | 61.258 | 70.025 | 1.00 | 0.74 |
| ATOM | 523 | O | SER | A | 40 | 84.607 | 60.639 | 69.603 | 1.00 | 0.74 |
| ATOM | 524 | N | ASP | A | 41 | 83.644 | 62.533 | 70.423 | 1.00 | 0.84 |

| | | | | | | | | | | |
|---|---|---|---|---|---|---|---|---|---|---|
| ATOM | 525 | H | ASP | A | 41 | 82.788 | 62.904 | 70.799 | 1.00 | 0.84 |
| ATOM | 526 | CA | ASP | A | 41 | 84.797 | 63.396 | 70.679 | 1.00 | 0.84 |
| ATOM | 527 | HA | ASP | A | 41 | 84.467 | 64.423 | 70.516 | 1.00 | 0.84 |
| ATOM | 528 | CB | ASP | A | 41 | 85.173 | 63.288 | 72.170 | 1.00 | 0.84 |
| ATOM | 529 | HB1 | ASP | A | 41 | 85.985 | 63.986 | 72.378 | 1.00 | 0.84 |
| ATOM | 530 | HB2 | ASP | A | 41 | 85.533 | 62.277 | 72.373 | 1.00 | 0.84 |
| ATOM | 531 | CG | ASP | A | 41 | 84.000 | 63.598 | 73.130 | 1.00 | 0.84 |
| ATOM | 532 | OD1 | ASP | A | 41 | 84.207 | 63.525 | 74.360 | 1.00 | 0.84 |
| ATOM | 533 | OD2 | ASP | A | 41 | 82.862 | 63.881 | 72.673 | 1.00 | 0.84 |
| ATOM | 534 | C | ASP | A | 41 | 85.911 | 63.166 | 69.658 | 1.00 | 0.84 |
| ATOM | 535 | O | ASP | A | 41 | 87.071 | 62.920 | 69.995 | 1.00 | 0.84 |
| ATOM | 536 | N | LEU | A | 42 | 85.492 | 63.161 | 68.388 | 1.00 | 0.79 |
| ATOM | 537 | H | LEU | A | 42 | 84.517 | 63.361 | 68.203 | 1.00 | 0.79 |
| ATOM | 538 | CA | LEU | A | 42 | 86.411 | 63.108 | 67.274 | 1.00 | 0.79 |
| ATOM | 539 | HA | LEU | A | 42 | 87.147 | 62.328 | 67.471 | 1.00 | 0.79 |
| ATOM | 540 | CB | LEU | A | 42 | 85.704 | 62.809 | 65.954 | 1.00 | 0.79 |
| ATOM | 541 | HB1 | LEU | A | 42 | 86.492 | 62.699 | 65.217 | 1.00 | 0.79 |
| ATOM | 542 | HB2 | LEU | A | 42 | 85.084 | 63.664 | 65.688 | 1.00 | 0.79 |
| ATOM | 543 | CG | LEU | A | 42 | 84.847 | 61.550 | 65.895 | 1.00 | 0.79 |
| ATOM | 544 | HG | LEU | A | 42 | 83.973 | 61.684 | 66.525 | 1.00 | 0.79 |
| ATOM | 545 | CD1 | LEU | A | 42 | 84.407 | 61.344 | 64.445 | 1.00 | 0.79 |
| ATOM | 546 | 1HD1 | LEU | A | 42 | 83.768 | 60.470 | 64.369 | 1.00 | 0.79 |
| ATOM | 547 | 2HD1 | LEU | A | 42 | 83.844 | 62.213 | 64.119 | 1.00 | 0.79 |
| ATOM | 548 | 3HD1 | LEU | A | 42 | 85.277 | 61.224 | 63.800 | 1.00 | 0.79 |
| ATOM | 549 | CD2 | LEU | A | 42 | 85.617 | 60.306 | 66.329 | 1.00 | 0.79 |
| ATOM | 550 | 1HD2 | LEU | A | 42 | 84.980 | 59.433 | 66.246 | 1.00 | 0.79 |
| ATOM | 551 | 2HD2 | LEU | A | 42 | 86.506 | 60.192 | 65.711 | 1.00 | 0.79 |
| ATOM | 552 | 3HD2 | LEU | A | 42 | 85.906 | 60.403 | 67.375 | 1.00 | 0.79 |
| ATOM | 553 | C | LEU | A | 42 | 87.141 | 64.439 | 67.166 | 1.00 | 0.79 |
| ATOM | 554 | O | LEU | A | 42 | 86.554 | 65.503 | 67.372 | 1.00 | 0.79 |
| ATOM | 555 | N | GLU | A | 43 | 88.415 | 64.355 | 66.797 | 1.00 | 1.00 |
| ATOM | 556 | H | GLU | A | 43 | 88.843 | 63.448 | 66.706 | 1.00 | 1.00 |
| ATOM | 557 | CA | GLU | A | 43 | 89.214 | 65.549 | 66.557 | 1.00 | 1.00 |
| ATOM | 558 | HA | GLU | A | 43 | 89.199 | 66.157 | 67.464 | 1.00 | 1.00 |
| ATOM | 559 | CB | GLU | A | 43 | 90.678 | 65.166 | 66.271 | 1.00 | 1.00 |
| ATOM | 560 | HB1 | GLU | A | 43 | 91.227 | 66.050 | 65.942 | 1.00 | 1.00 |
| ATOM | 561 | HB2 | GLU | A | 43 | 90.695 | 64.448 | 65.449 | 1.00 | 1.00 |
| ATOM | 562 | CG | GLU | A | 43 | 91.415 | 64.544 | 67.475 | 1.00 | 1.00 |
| ATOM | 563 | HG1 | GLU | A | 43 | 92.366 | 64.150 | 67.110 | 1.00 | 1.00 |
| ATOM | 564 | HG2 | GLU | A | 43 | 90.834 | 63.696 | 67.842 | 1.00 | 1.00 |
| ATOM | 565 | CD | GLU | A | 43 | 91.707 | 65.505 | 68.644 | 1.00 | 1.00 |
| ATOM | 566 | OE1 | GLU | A | 43 | 91.487 | 66.731 | 68.491 | 1.00 | 1.00 |
| ATOM | 567 | OE2 | GLU | A | 43 | 92.176 | 64.995 | 69.690 | 1.00 | 1.00 |
| ATOM | 568 | C | GLU | A | 43 | 88.647 | 66.417 | 65.429 | 1.00 | 1.00 |

| | | | | | | | | | | |
|---|---|---|---|---|---|---|---|---|---|---|
| ATOM | 569 | O | GLU | A | 43 | 88.092 | 65.918 | 64.436 | 1.00 | 1.00 |
| ATOM | 570 | N | ASP | A | 44 | 88.790 | 67.728 | 65.599 | 1.00 | 0.99 |
| ATOM | 571 | H | ASP | A | 44 | 89.273 | 68.075 | 66.413 | 1.00 | 0.99 |
| ATOM | 572 | CA | ASP | A | 44 | 88.166 | 68.676 | 64.690 | 1.00 | 0.99 |
| ATOM | 573 | HA | ASP | A | 44 | 87.122 | 68.389 | 64.658 | 1.00 | 0.99 |
| ATOM | 574 | CB | ASP | A | 44 | 88.248 | 70.115 | 65.245 | 1.00 | 0.99 |
| ATOM | 575 | HB1 | ASP | A | 44 | 88.049 | 70.804 | 64.423 | 1.00 | 0.99 |
| ATOM | 576 | HB2 | ASP | A | 44 | 89.263 | 70.309 | 65.597 | 1.00 | 0.99 |
| ATOM | 577 | CG | ASP | A | 44 | 87.244 | 70.446 | 66.370 | 1.00 | 0.99 |
| ATOM | 578 | OD1 | ASP | A | 44 | 86.466 | 69.579 | 66.825 | 1.00 | 0.99 |
| ATOM | 579 | OD2 | ASP | A | 44 | 87.190 | 71.629 | 66.787 | 1.00 | 0.99 |
| ATOM | 580 | C | ASP | A | 44 | 88.709 | 68.551 | 63.256 | 1.00 | 0.99 |
| ATOM | 581 | O | ASP | A | 44 | 89.923 | 68.406 | 63.083 | 1.00 | 0.99 |
| ATOM | 582 | N | ASP | A | 45 | 87.807 | 68.576 | 62.255 | 1.00 | 0.88 |
| ATOM | 583 | H | ASP | A | 45 | 86.834 | 68.711 | 62.501 | 1.00 | 0.88 |
| ATOM | 584 | CA | ASP | A | 45 | 88.105 | 68.318 | 60.818 | 1.00 | 0.88 |
| ATOM | 585 | HA | ASP | A | 45 | 87.338 | 68.834 | 60.246 | 1.00 | 0.88 |
| ATOM | 586 | CB | ASP | A | 45 | 89.428 | 68.982 | 60.341 | 1.00 | 0.88 |
| ATOM | 587 | HB1 | ASP | A | 45 | 90.262 | 68.335 | 60.617 | 1.00 | 0.88 |
| ATOM | 588 | HB2 | ASP | A | 45 | 89.546 | 69.931 | 60.866 | 1.00 | 0.88 |
| ATOM | 589 | CG | ASP | A | 45 | 89.550 | 69.293 | 58.836 | 1.00 | 0.88 |
| ATOM | 590 | OD1 | ASP | A | 45 | 88.537 | 69.641 | 58.185 | 1.00 | 0.88 |
| ATOM | 591 | OD2 | ASP | A | 45 | 90.706 | 69.269 | 58.345 | 1.00 | 0.88 |
| ATOM | 592 | C | ASP | A | 45 | 88.001 | 66.834 | 60.413 | 1.00 | 0.88 |
| ATOM | 593 | O | ASP | A | 45 | 88.079 | 66.497 | 59.225 | 1.00 | 0.88 |
| ATOM | 594 | N | THR | A | 46 | 87.838 | 65.903 | 61.370 | 1.00 | 0.57 |
| ATOM | 595 | H | THR | A | 46 | 87.814 | 66.187 | 62.344 | 1.00 | 0.57 |
| ATOM | 596 | CA | THR | A | 46 | 87.839 | 64.473 | 61.015 | 1.00 | 0.57 |
| ATOM | 597 | HA | THR | A | 46 | 88.780 | 64.323 | 60.489 | 1.00 | 0.57 |
| ATOM | 598 | CB | THR | A | 46 | 87.888 | 63.517 | 62.208 | 1.00 | 0.57 |
| ATOM | 599 | HB | THR | A | 46 | 87.667 | 62.507 | 61.861 | 1.00 | 0.57 |
| ATOM | 600 | CG2 | THR | A | 46 | 89.266 | 63.500 | 62.859 | 1.00 | 0.57 |
| ATOM | 601 | 1HG2 | THR | A | 46 | 89.979 | 63.109 | 62.144 | 1.00 | 0.57 |
| ATOM | 602 | 2HG2 | THR | A | 46 | 89.588 | 64.504 | 63.128 | 1.00 | 0.57 |
| ATOM | 603 | 3HG2 | THR | A | 46 | 89.257 | 62.861 | 63.742 | 1.00 | 0.57 |
| ATOM | 604 | OG1 | THR | A | 46 | 86.946 | 63.875 | 63.170 | 1.00 | 0.57 |
| ATOM | 605 | HG1 | THR | A | 46 | 87.336 | 64.634 | 63.654 | 1.00 | 0.57 |
| ATOM | 606 | C | THR | A | 46 | 86.734 | 64.062 | 60.034 | 1.00 | 0.57 |
| ATOM | 607 | O | THR | A | 46 | 85.521 | 64.261 | 60.207 | 1.00 | 0.57 |
| ATOM | 608 | N | THR | A | 47 | 87.178 | 63.368 | 58.986 | 1.00 | 0.42 |
| ATOM | 609 | H | THR | A | 47 | 88.183 | 63.249 | 58.900 | 1.00 | 0.42 |
| ATOM | 610 | CA | THR | A | 47 | 86.273 | 62.554 | 58.171 | 1.00 | 0.42 |
| ATOM | 611 | HA | THR | A | 47 | 85.351 | 63.102 | 58.024 | 1.00 | 0.42 |
| ATOM | 612 | CB | THR | A | 47 | 86.904 | 62.255 | 56.800 | 1.00 | 0.42 |

| | | | | | | | | | | |
|---|---|---|---|---|---|---|---|---|---|---|
| ATOM | 613 | HB | THR | A | 47 | 87.973 | 62.100 | 56.927 | 1.00 | 0.42 |
| ATOM | 614 | CG2 | THR | A | 47 | 86.325 | 61.052 | 56.045 | 1.00 | 0.42 |
| ATOM | 615 | 1HG2 | THR | A | 47 | 86.722 | 61.029 | 55.030 | 1.00 | 0.42 |
| ATOM | 616 | 2HG2 | THR | A | 47 | 86.614 | 60.126 | 56.540 | 1.00 | 0.42 |
| ATOM | 617 | 3HG2 | THR | A | 47 | 85.239 | 61.101 | 56.013 | 1.00 | 0.42 |
| ATOM | 618 | OG1 | THR | A | 47 | 86.701 | 63.363 | 55.953 | 1.00 | 0.42 |
| ATOM | 619 | HG1 | THR | A | 47 | 87.188 | 64.100 | 56.356 | 1.00 | 0.42 |
| ATOM | 620 | C | THR | A | 47 | 85.958 | 61.253 | 58.901 | 1.00 | 0.42 |
| ATOM | 621 | O | THR | A | 47 | 86.858 | 60.554 | 59.354 | 1.00 | 0.42 |
| ATOM | 622 | N | VAL | A | 48 | 84.703 | 60.821 | 58.842 | 1.00 | 0.38 |
| ATOM | 623 | H | VAL | A | 48 | 84.017 | 61.431 | 58.406 | 1.00 | 0.38 |
| ATOM | 624 | CA | VAL | A | 48 | 84.339 | 59.403 | 58.934 | 1.00 | 0.38 |
| ATOM | 625 | HA | VAL | A | 48 | 85.234 | 58.807 | 59.089 | 1.00 | 0.38 |
| ATOM | 626 | CB | VAL | A | 48 | 83.370 | 59.118 | 60.102 | 1.00 | 0.38 |
| ATOM | 627 | HB | VAL | A | 48 | 82.397 | 59.554 | 59.881 | 1.00 | 0.38 |
| ATOM | 628 | CG1 | VAL | A | 48 | 83.186 | 57.614 | 60.337 | 1.00 | 0.38 |
| ATOM | 629 | 1HG1 | VAL | A | 48 | 82.515 | 57.454 | 61.180 | 1.00 | 0.38 |
| ATOM | 630 | 2HG1 | VAL | A | 48 | 82.738 | 57.144 | 59.463 | 1.00 | 0.38 |
| ATOM | 631 | 3HG1 | VAL | A | 48 | 84.146 | 57.144 | 60.554 | 1.00 | 0.38 |
| ATOM | 632 | CG2 | VAL | A | 48 | 83.882 | 59.699 | 61.425 | 1.00 | 0.38 |
| ATOM | 633 | 1HG2 | VAL | A | 48 | 83.219 | 59.417 | 62.242 | 1.00 | 0.38 |
| ATOM | 634 | 2HG2 | VAL | A | 48 | 84.887 | 59.330 | 61.635 | 1.00 | 0.38 |
| ATOM | 635 | 3HG2 | VAL | A | 48 | 83.913 | 60.788 | 61.375 | 1.00 | 0.38 |
| ATOM | 636 | C | VAL | A | 48 | 83.717 | 59.035 | 57.597 | 1.00 | 0.38 |
| ATOM | 637 | O | VAL | A | 48 | 83.012 | 59.850 | 56.981 | 1.00 | 0.38 |
| ATOM | 638 | N | ILE | A | 49 | 84.031 | 57.815 | 57.158 | 1.00 | 0.53 |
| ATOM | 639 | H | ILE | A | 49 | 84.683 | 57.274 | 57.718 | 1.00 | 0.53 |
| ATOM | 640 | CA | ILE | A | 49 | 83.382 | 57.160 | 56.025 | 1.00 | 0.53 |
| ATOM | 641 | HA | ILE | A | 49 | 83.080 | 57.968 | 55.358 | 1.00 | 0.53 |
| ATOM | 642 | CB | ILE | A | 49 | 84.362 | 56.320 | 55.156 | 1.00 | 0.53 |
| ATOM | 643 | HB | ILE | A | 49 | 84.525 | 55.348 | 55.621 | 1.00 | 0.53 |
| ATOM | 644 | CG2 | ILE | A | 49 | 83.771 | 56.111 | 53.746 | 1.00 | 0.53 |
| ATOM | 645 | 1HG2 | ILE | A | 49 | 84.341 | 55.343 | 53.218 | 1.00 | 0.53 |
| ATOM | 646 | 2HG2 | ILE | A | 49 | 82.731 | 55.795 | 53.769 | 1.00 | 0.53 |
| ATOM | 647 | 3HG2 | ILE | A | 49 | 83.813 | 57.042 | 53.180 | 1.00 | 0.53 |
| ATOM | 648 | CG1 | ILE | A | 49 | 85.729 | 57.049 | 55.023 | 1.00 | 0.53 |
| ATOM | 649 | 1HG1 | ILE | A | 49 | 86.281 | 56.923 | 55.955 | 1.00 | 0.53 |
| ATOM | 650 | 2HG1 | ILE | A | 49 | 85.553 | 58.117 | 54.884 | 1.00 | 0.53 |
| ATOM | 651 | CD1 | ILE | A | 49 | 86.641 | 56.594 | 53.874 | 1.00 | 0.53 |
| ATOM | 652 | HD1 | ILE | A | 49 | 87.641 | 57.004 | 54.024 | 1.00 | 0.53 |
| ATOM | 653 | HD2 | ILE | A | 49 | 86.698 | 55.508 | 53.842 | 1.00 | 0.53 |
| ATOM | 654 | HD3 | ILE | A | 49 | 86.259 | 56.958 | 52.921 | 1.00 | 0.53 |
| ATOM | 655 | C | ILE | A | 49 | 82.042 | 56.560 | 56.489 | 1.00 | 0.53 |
| ATOM | 656 | O | ILE | A | 49 | 81.258 | 57.283 | 57.137 | 1.00 | 0.53 |

| | | | | | | | | | | |
|---|---|---|---|---|---|---|---|---|---|---|
| ATOM | 657 | N | PHE | A | 50 | 81.839 | 55.307 | 56.061 | 1.00 | 0.41 |
| ATOM | 658 | H | PHE | A | 50 | 82.466 | 55.066 | 55.310 | 1.00 | 0.41 |
| ATOM | 659 | CA | PHE | A | 50 | 81.028 | 54.146 | 56.487 | 1.00 | 0.41 |
| ATOM | 660 | HA | PHE | A | 50 | 81.577 | 53.602 | 57.257 | 1.00 | 0.41 |
| ATOM | 661 | CB | PHE | A | 50 | 79.654 | 54.542 | 57.056 | 1.00 | 0.41 |
| ATOM | 662 | HB1 | PHE | A | 50 | 78.921 | 53.803 | 56.731 | 1.00 | 0.41 |
| ATOM | 663 | HB2 | PHE | A | 50 | 79.336 | 55.501 | 56.644 | 1.00 | 0.41 |
| ATOM | 664 | CG | PHE | A | 50 | 79.616 | 54.570 | 58.573 | 1.00 | 0.41 |
| ATOM | 665 | CD1 | PHE | A | 50 | 79.485 | 55.783 | 59.279 | 1.00 | 0.41 |
| ATOM | 666 | HD1 | PHE | A | 50 | 79.412 | 56.717 | 58.741 | 1.00 | 0.41 |
| ATOM | 667 | CE1 | PHE | A | 50 | 79.469 | 55.785 | 60.686 | 1.00 | 0.41 |
| ATOM | 668 | HE1 | PHE | A | 50 | 79.392 | 56.717 | 61.228 | 1.00 | 0.41 |
| ATOM | 669 | CZ | PHE | A | 50 | 79.572 | 54.571 | 61.389 | 1.00 | 0.41 |
| ATOM | 670 | HZ | PHE | A | 50 | 79.577 | 54.573 | 62.469 | 1.00 | 0.41 |
| ATOM | 671 | CE2 | PHE | A | 50 | 79.698 | 53.358 | 60.688 | 1.00 | 0.41 |
| ATOM | 672 | HE2 | PHE | A | 50 | 79.792 | 52.423 | 61.222 | 1.00 | 0.41 |
| ATOM | 673 | CD2 | PHE | A | 50 | 79.720 | 53.358 | 59.284 | 1.00 | 0.41 |
| ATOM | 674 | HD2 | PHE | A | 50 | 79.826 | 52.420 | 58.752 | 1.00 | 0.41 |
| ATOM | 675 | C | PHE | A | 50 | 80.904 | 53.220 | 55.274 | 1.00 | 0.41 |
| ATOM | 676 | O | PHE | A | 50 | 80.413 | 53.644 | 54.233 | 1.00 | 0.41 |
| ATOM | 677 | N | GLU | A | 51 | 81.412 | 52.000 | 55.376 | 1.00 | 0.39 |
| ATOM | 678 | H | GLU | A | 51 | 81.650 | 51.623 | 56.299 | 1.00 | 0.39 |
| ATOM | 679 | CA | GLU | A | 51 | 81.918 | 51.208 | 54.250 | 1.00 | 0.39 |
| ATOM | 680 | HA | GLU | A | 51 | 81.446 | 51.514 | 53.317 | 1.00 | 0.39 |
| ATOM | 681 | CB | GLU | A | 51 | 83.438 | 51.445 | 54.151 | 1.00 | 0.39 |
| ATOM | 682 | HB1 | GLU | A | 51 | 83.895 | 51.307 | 55.131 | 1.00 | 0.39 |
| ATOM | 683 | HB2 | GLU | A | 51 | 83.593 | 52.485 | 53.857 | 1.00 | 0.39 |
| ATOM | 684 | CG | GLU | A | 51 | 84.159 | 50.527 | 53.153 | 1.00 | 0.39 |
| ATOM | 685 | HG1 | GLU | A | 51 | 83.643 | 50.575 | 52.192 | 1.00 | 0.39 |
| ATOM | 686 | HG2 | GLU | A | 51 | 84.119 | 49.496 | 53.513 | 1.00 | 0.39 |
| ATOM | 687 | CD | GLU | A | 51 | 85.618 | 50.967 | 52.978 | 1.00 | 0.39 |
| ATOM | 688 | OE1 | GLU | A | 51 | 85.792 | 52.071 | 52.415 | 1.00 | 0.39 |
| ATOM | 689 | OE2 | GLU | A | 51 | 86.530 | 50.234 | 53.431 | 1.00 | 0.39 |
| ATOM | 690 | C | GLU | A | 51 | 81.598 | 49.737 | 54.527 | 1.00 | 0.39 |
| ATOM | 691 | O | GLU | A | 51 | 81.813 | 49.240 | 55.643 | 1.00 | 0.39 |
| ATOM | 692 | N | GLY | A | 52 | 81.056 | 49.083 | 53.494 | 1.00 | 0.49 |
| ATOM | 693 | H | GLY | A | 52 | 80.953 | 49.576 | 52.621 | 1.00 | 0.49 |
| ATOM | 694 | CA | GLY | A | 52 | 80.463 | 47.753 | 53.579 | 1.00 | 0.49 |
| ATOM | 695 | HA1 | GLY | A | 52 | 81.196 | 47.066 | 54.006 | 1.00 | 0.49 |
| ATOM | 696 | HA2 | GLY | A | 52 | 80.204 | 47.393 | 52.584 | 1.00 | 0.49 |
| ATOM | 697 | C | GLY | A | 52 | 79.208 | 47.731 | 54.445 | 1.00 | 0.49 |
| ATOM | 698 | O | GLY | A | 52 | 78.341 | 48.598 | 54.295 | 1.00 | 0.49 |
| ATOM | 699 | N | THR | A | 53 | 79.071 | 46.748 | 55.332 | 1.00 | 0.41 |
| ATOM | 700 | H | THR | A | 53 | 79.806 | 46.058 | 55.459 | 1.00 | 0.41 |

| | | | | | | | | | | |
|---|---|---|---|---|---|---|---|---|---|---|
| ATOM | 701 | CA | THR | A | 53 | 77.946 | 46.675 | 56.230 | 1.00 | 0.41 |
| ATOM | 702 | HA | THR | A | 53 | 77.073 | 47.152 | 55.793 | 1.00 | 0.41 |
| ATOM | 703 | CB | THR | A | 53 | 77.603 | 45.217 | 56.604 | 1.00 | 0.41 |
| ATOM | 704 | HB | THR | A | 53 | 78.303 | 44.863 | 57.362 | 1.00 | 0.41 |
| ATOM | 705 | CG2 | THR | A | 53 | 76.176 | 45.066 | 57.136 | 1.00 | 0.41 |
| ATOM | 706 | 1HG2 | THR | A | 53 | 75.960 | 44.007 | 57.277 | 1.00 | 0.41 |
| ATOM | 707 | 2HG2 | THR | A | 53 | 76.077 | 45.566 | 58.096 | 1.00 | 0.41 |
| ATOM | 708 | 3HG2 | THR | A | 53 | 75.471 | 45.490 | 56.420 | 1.00 | 0.41 |
| ATOM | 709 | OG1 | THR | A | 53 | 77.703 | 44.362 | 55.492 | 1.00 | 0.41 |
| ATOM | 710 | HG1 | THR | A | 53 | 78.670 | 44.234 | 55.358 | 1.00 | 0.41 |
| ATOM | 711 | C | THR | A | 53 | 78.414 | 47.410 | 57.493 | 1.00 | 0.41 |
| ATOM | 712 | O | THR | A | 53 | 79.592 | 47.490 | 57.879 | 1.00 | 0.41 |
| ATOM | 713 | N | THR | A | 54 | 77.455 | 47.883 | 58.256 | 1.00 | 0.32 |
| ATOM | 714 | H | THR | A | 54 | 76.501 | 47.881 | 57.907 | 1.00 | 0.32 |
| ATOM | 715 | CA | THR | A | 54 | 77.693 | 47.975 | 59.687 | 1.00 | 0.32 |
| ATOM | 716 | HA | THR | A | 54 | 78.562 | 47.380 | 59.979 | 1.00 | 0.32 |
| ATOM | 717 | CB | THR | A | 54 | 77.994 | 49.422 | 60.102 | 1.00 | 0.32 |
| ATOM | 718 | HB | THR | A | 54 | 77.284 | 50.091 | 59.615 | 1.00 | 0.32 |
| ATOM | 719 | CG2 | THR | A | 54 | 77.956 | 49.670 | 61.610 | 1.00 | 0.32 |
| ATOM | 720 | 1HG2 | THR | A | 54 | 78.293 | 50.682 | 61.826 | 1.00 | 0.32 |
| ATOM | 721 | 2HG2 | THR | A | 54 | 76.938 | 49.558 | 61.982 | 1.00 | 0.32 |
| ATOM | 722 | 3HG2 | THR | A | 54 | 78.599 | 48.961 | 62.127 | 1.00 | 0.32 |
| ATOM | 723 | OG1 | THR | A | 54 | 79.299 | 49.734 | 59.669 | 1.00 | 0.32 |
| ATOM | 724 | HG1 | THR | A | 54 | 79.515 | 49.150 | 58.923 | 1.00 | 0.32 |
| ATOM | 725 | C | THR | A | 54 | 76.501 | 47.379 | 60.360 | 1.00 | 0.32 |
| ATOM | 726 | O | THR | A | 54 | 75.363 | 47.792 | 60.080 | 1.00 | 0.32 |
| ATOM | 727 | N | SER | A | 55 | 76.840 | 46.358 | 61.151 | 1.00 | 0.42 |
| ATOM | 728 | H | SER | A | 55 | 77.831 | 46.224 | 61.347 | 1.00 | 0.42 |
| ATOM | 729 | CA | SER | A | 55 | 75.919 | 45.507 | 61.893 | 1.00 | 0.42 |
| ATOM | 730 | HA | SER | A | 55 | 74.915 | 45.603 | 61.478 | 1.00 | 0.42 |
| ATOM | 731 | CB | SER | A | 55 | 76.357 | 44.043 | 61.714 | 1.00 | 0.42 |
| ATOM | 732 | HB1 | SER | A | 55 | 75.605 | 43.381 | 62.147 | 1.00 | 0.42 |
| ATOM | 733 | HB2 | SER | A | 55 | 77.307 | 43.880 | 62.223 | 1.00 | 0.42 |
| ATOM | 734 | OG | SER | A | 55 | 76.502 | 43.733 | 60.338 | 1.00 | 0.42 |
| ATOM | 735 | HG | SER | A | 55 | 77.372 | 44.039 | 60.050 | 1.00 | 0.42 |
| ATOM | 736 | C | SER | A | 55 | 75.866 | 45.947 | 63.363 | 1.00 | 0.42 |
| ATOM | 737 | O | SER | A | 55 | 76.300 | 47.017 | 63.791 | 1.00 | 0.42 |
| ATOM | 738 | N | TRP | A | 56 | 75.301 | 45.082 | 64.170 | 1.00 | 0.80 |
| ATOM | 739 | H | TRP | A | 56 | 75.010 | 44.191 | 63.782 | 1.00 | 0.80 |
| ATOM | 740 | CA | TRP | A | 56 | 74.983 | 45.208 | 65.583 | 1.00 | 0.80 |
| ATOM | 741 | HA | TRP | A | 56 | 75.750 | 45.765 | 66.123 | 1.00 | 0.80 |
| ATOM | 742 | CB | TRP | A | 56 | 73.627 | 45.886 | 65.709 | 1.00 | 0.80 |
| ATOM | 743 | HB1 | TRP | A | 56 | 73.670 | 46.882 | 65.266 | 1.00 | 0.80 |
| ATOM | 744 | HB2 | TRP | A | 56 | 73.379 | 45.980 | 66.765 | 1.00 | 0.80 |

| | | | | | | | | | | |
|---|---|---|---|---|---|---|---|---|---|---|
| ATOM | 745 | CG | TRP | A | 56 | 72.565 | 45.091 | 65.041 | 1.00 | 0.80 |
| ATOM | 746 | CD1 | TRP | A | 56 | 72.380 | 44.961 | 63.711 | 1.00 | 0.80 |
| ATOM | 747 | HD1 | TRP | A | 56 | 72.948 | 45.473 | 62.946 | 1.00 | 0.80 |
| ATOM | 748 | NE1 | TRP | A | 56 | 71.395 | 44.035 | 63.476 | 1.00 | 0.80 |
| ATOM | 749 | HE1 | TRP | A | 56 | 71.041 | 43.823 | 62.551 | 1.00 | 0.80 |
| ATOM | 750 | CE2 | TRP | A | 56 | 70.848 | 43.570 | 64.650 | 1.00 | 0.80 |
| ATOM | 751 | CZ2 | TRP | A | 56 | 69.787 | 42.711 | 64.950 | 1.00 | 0.80 |
| ATOM | 752 | HZ2 | TRP | A | 56 | 69.194 | 42.263 | 64.176 | 1.00 | 0.80 |
| ATOM | 753 | CH2 | TRP | A | 56 | 69.465 | 42.487 | 66.290 | 1.00 | 0.80 |
| ATOM | 754 | HH2 | TRP | A | 56 | 68.651 | 41.856 | 66.569 | 1.00 | 0.80 |
| ATOM | 755 | CZ3 | TRP | A | 56 | 70.178 | 43.120 | 67.300 | 1.00 | 0.80 |
| ATOM | 756 | HZ3 | TRP | A | 56 | 69.783 | 43.065 | 68.294 | 1.00 | 0.80 |
| ATOM | 757 | CE3 | TRP | A | 56 | 71.299 | 43.909 | 67.008 | 1.00 | 0.80 |
| ATOM | 758 | HE3 | TRP | A | 56 | 71.854 | 44.384 | 67.800 | 1.00 | 0.80 |
| ATOM | 759 | CD2 | TRP | A | 56 | 71.616 | 44.198 | 65.669 | 1.00 | 0.80 |
| ATOM | 760 | C | TRP | A | 56 | 74.959 | 43.754 | 66.065 | 1.00 | 0.80 |
| ATOM | 761 | O | TRP | A | 56 | 74.803 | 42.848 | 65.242 | 1.00 | 0.80 |
| ATOM | 762 | N | GLU | A | 57 | 75.021 | 43.508 | 67.365 | 1.00 | 1.04 |
| ATOM | 763 | H | GLU | A | 57 | 75.260 | 44.235 | 68.022 | 1.00 | 1.04 |
| ATOM | 764 | CA | GLU | A | 57 | 74.541 | 42.240 | 67.880 | 1.00 | 1.04 |
| ATOM | 765 | HA | GLU | A | 57 | 73.868 | 41.854 | 67.116 | 1.00 | 1.04 |
| ATOM | 766 | CB | GLU | A | 57 | 75.629 | 41.164 | 68.072 | 1.00 | 1.04 |
| ATOM | 767 | HB1 | GLU | A | 57 | 75.415 | 40.590 | 68.970 | 1.00 | 1.04 |
| ATOM | 768 | HB2 | GLU | A | 57 | 76.606 | 41.633 | 68.198 | 1.00 | 1.04 |
| ATOM | 769 | CG | GLU | A | 57 | 75.676 | 40.160 | 66.894 | 1.00 | 1.04 |
| ATOM | 770 | HG1 | GLU | A | 57 | 76.373 | 39.360 | 67.154 | 1.00 | 1.04 |
| ATOM | 771 | HG2 | GLU | A | 57 | 76.080 | 40.665 | 66.015 | 1.00 | 1.04 |
| ATOM | 772 | CD | GLU | A | 57 | 74.299 | 39.547 | 66.567 | 1.00 | 1.04 |
| ATOM | 773 | OE1 | GLU | A | 57 | 73.525 | 39.242 | 67.505 | 1.00 | 1.04 |
| ATOM | 774 | OE2 | GLU | A | 57 | 73.910 | 39.437 | 65.384 | 1.00 | 1.04 |
| ATOM | 775 | C | GLU | A | 57 | 73.542 | 42.415 | 69.016 | 1.00 | 1.04 |
| ATOM | 776 | O | GLU | A | 57 | 73.419 | 43.490 | 69.612 | 1.00 | 1.04 |
| ATOM | 777 | N | TYR | A | 58 | 72.694 | 41.400 | 69.175 | 1.00 | 1.03 |
| ATOM | 778 | H | TYR | A | 58 | 72.854 | 40.536 | 68.657 | 1.00 | 1.03 |
| ATOM | 779 | CA | TYR | A | 58 | 71.453 | 41.571 | 69.906 | 1.00 | 1.03 |
| ATOM | 780 | HA | TYR | A | 58 | 71.016 | 42.507 | 69.609 | 1.00 | 1.03 |
| ATOM | 781 | CB | TYR | A | 58 | 70.472 | 40.413 | 69.658 | 1.00 | 1.03 |
| ATOM | 782 | HB1 | TYR | A | 58 | 70.956 | 39.474 | 69.934 | 1.00 | 1.03 |
| ATOM | 783 | HB2 | TYR | A | 58 | 70.246 | 40.369 | 68.593 | 1.00 | 1.03 |
| ATOM | 784 | CG | TYR | A | 58 | 69.168 | 40.545 | 70.437 | 1.00 | 1.03 |
| ATOM | 785 | CD1 | TYR | A | 58 | 68.079 | 41.231 | 69.866 | 1.00 | 1.03 |
| ATOM | 786 | HD1 | TYR | A | 58 | 68.147 | 41.640 | 68.871 | 1.00 | 1.03 |
| ATOM | 787 | CE1 | TYR | A | 58 | 66.885 | 41.390 | 70.592 | 1.00 | 1.03 |
| ATOM | 788 | HE1 | TYR | A | 58 | 66.046 | 41.911 | 70.162 | 1.00 | 1.03 |

| | | | | | | | | | | |
|---|---|---|---|---|---|---|---|---|---|---|
| ATOM | 789 | CZ | TYR | A | 58 | 66.766 | 40.882 | 71.899 | 1.00 | 1.03 |
| ATOM | 790 | OH | TYR | A | 58 | 65.600 | 41.063 | 72.569 | 1.00 | 1.03 |
| ATOM | 791 | HH | TYR | A | 58 | 65.617 | 40.737 | 73.501 | 1.00 | 1.03 |
| ATOM | 792 | CE2 | TYR | A | 58 | 67.859 | 40.207 | 72.484 | 1.00 | 1.03 |
| ATOM | 793 | HE2 | TYR | A | 58 | 67.760 | 39.829 | 73.493 | 1.00 | 1.03 |
| ATOM | 794 | CD2 | TYR | A | 58 | 69.052 | 40.029 | 71.749 | 1.00 | 1.03 |
| ATOM | 795 | HD2 | TYR | A | 58 | 69.889 | 39.510 | 72.198 | 1.00 | 1.03 |
| ATOM | 796 | C | TYR | A | 58 | 71.714 | 41.721 | 71.397 | 1.00 | 1.03 |
| ATOM | 797 | O | TYR | A | 58 | 72.436 | 40.929 | 72.009 | 1.00 | 1.03 |
| ATOM | 798 | N | GLU | A | 59 | 71.056 | 42.726 | 71.967 | 1.00 | 0.94 |
| ATOM | 799 | H | GLU | A | 59 | 70.519 | 43.342 | 71.375 | 1.00 | 0.94 |
| ATOM | 800 | CA | GLU | A | 59 | 70.792 | 42.823 | 73.391 | 1.00 | 0.94 |
| ATOM | 801 | HA | GLU | A | 59 | 70.554 | 41.829 | 73.772 | 1.00 | 0.94 |
| ATOM | 802 | CB | GLU | A | 59 | 72.029 | 43.354 | 74.141 | 1.00 | 0.94 |
| ATOM | 803 | HB1 | GLU | A | 59 | 72.158 | 44.417 | 73.930 | 1.00 | 0.94 |
| ATOM | 804 | HB2 | GLU | A | 59 | 72.918 | 42.829 | 73.795 | 1.00 | 0.94 |
| ATOM | 805 | CG | GLU | A | 59 | 71.905 | 43.132 | 75.655 | 1.00 | 0.94 |
| ATOM | 806 | HG1 | GLU | A | 59 | 71.761 | 42.065 | 75.846 | 1.00 | 0.94 |
| ATOM | 807 | HG2 | GLU | A | 59 | 71.029 | 43.661 | 76.034 | 1.00 | 0.94 |
| ATOM | 808 | CD | GLU | A | 59 | 73.151 | 43.632 | 76.391 | 1.00 | 0.94 |
| ATOM | 809 | OE1 | GLU | A | 59 | 74.042 | 42.798 | 76.668 | 1.00 | 0.94 |
| ATOM | 810 | OE2 | GLU | A | 59 | 73.196 | 44.852 | 76.657 | 1.00 | 0.94 |
| ATOM | 811 | C | GLU | A | 59 | 69.611 | 43.751 | 73.636 | 1.00 | 0.94 |
| ATOM | 812 | O | GLU | A | 59 | 69.464 | 44.762 | 72.950 | 1.00 | 0.94 |
| ATOM | 813 | N | GLU | A | 60 | 68.752 | 43.433 | 74.594 | 1.00 | 0.89 |
| ATOM | 814 | H | GLU | A | 60 | 68.924 | 42.631 | 75.182 | 1.00 | 0.89 |
| ATOM | 815 | CA | GLU | A | 60 | 67.628 | 44.298 | 74.920 | 1.00 | 0.89 |
| ATOM | 816 | HA | GLU | A | 60 | 67.328 | 44.806 | 74.006 | 1.00 | 0.89 |
| ATOM | 817 | CB | GLU | A | 60 | 66.376 | 43.497 | 75.281 | 1.00 | 0.89 |
| ATOM | 818 | HB1 | GLU | A | 60 | 66.048 | 43.073 | 74.334 | 1.00 | 0.89 |
| ATOM | 819 | HB2 | GLU | A | 60 | 65.595 | 44.178 | 75.619 | 1.00 | 0.89 |
| ATOM | 820 | CG | GLU | A | 60 | 66.498 | 42.341 | 76.280 | 1.00 | 0.89 |
| ATOM | 821 | HG1 | GLU | A | 60 | 66.422 | 42.721 | 77.301 | 1.00 | 0.89 |
| ATOM | 822 | HG2 | GLU | A | 60 | 67.460 | 41.836 | 76.170 | 1.00 | 0.89 |
| ATOM | 823 | CD | GLU | A | 60 | 65.373 | 41.348 | 75.972 | 1.00 | 0.89 |
| ATOM | 824 | OE1 | GLU | A | 60 | 64.264 | 41.526 | 76.522 | 1.00 | 0.89 |
| ATOM | 825 | OE2 | GLU | A | 60 | 65.612 | 40.479 | 75.095 | 1.00 | 0.89 |
| ATOM | 826 | C | GLU | A | 60 | 68.000 | 45.421 | 75.883 | 1.00 | 0.89 |
| ATOM | 827 | O | GLU | A | 60 | 67.883 | 45.321 | 77.102 | 1.00 | 0.89 |
| ATOM | 828 | N | TRP | A | 61 | 68.450 | 46.510 | 75.265 | 1.00 | 1.00 |
| ATOM | 829 | H | TRP | A | 61 | 68.456 | 46.495 | 74.257 | 1.00 | 1.00 |
| ATOM | 830 | CA | TRP | A | 61 | 69.025 | 47.696 | 75.895 | 1.00 | 1.00 |
| ATOM | 831 | HA | TRP | A | 61 | 68.646 | 47.764 | 76.915 | 1.00 | 1.00 |
| ATOM | 832 | CB | TRP | A | 61 | 70.557 | 47.562 | 75.967 | 1.00 | 1.00 |

| | | | | | | | | | | |
|---|---|---|---|---|---|---|---|---|---|---|
| ATOM | 833 | HB1 | TRP | A | 61 | 70.816 | 46.536 | 76.235 | 1.00 | 1.00 |
| ATOM | 834 | HB2 | TRP | A | 61 | 70.889 | 48.186 | 76.797 | 1.00 | 1.00 |
| ATOM | 835 | CG | TRP | A | 61 | 71.374 | 47.958 | 74.765 | 1.00 | 1.00 |
| ATOM | 836 | CD1 | TRP | A | 61 | 71.179 | 47.582 | 73.481 | 1.00 | 1.00 |
| ATOM | 837 | HD1 | TRP | A | 61 | 70.379 | 46.938 | 73.147 | 1.00 | 1.00 |
| ATOM | 838 | NE1 | TRP | A | 61 | 72.155 | 48.135 | 72.672 | 1.00 | 1.00 |
| ATOM | 839 | HE1 | TRP | A | 61 | 72.215 | 47.971 | 71.679 | 1.00 | 1.00 |
| ATOM | 840 | CE2 | TRP | A | 61 | 73.037 | 48.900 | 73.400 | 1.00 | 1.00 |
| ATOM | 841 | CZ2 | TRP | A | 61 | 74.167 | 49.656 | 73.051 | 1.00 | 1.00 |
| ATOM | 842 | HZ2 | TRP | A | 61 | 74.491 | 49.723 | 72.023 | 1.00 | 1.00 |
| ATOM | 843 | CH2 | TRP | A | 61 | 74.867 | 50.327 | 74.068 | 1.00 | 1.00 |
| ATOM | 844 | HH2 | TRP | A | 61 | 75.745 | 50.907 | 73.838 | 1.00 | 1.00 |
| ATOM | 845 | CZ3 | TRP | A | 61 | 74.439 | 50.235 | 75.402 | 1.00 | 1.00 |
| ATOM | 846 | HZ3 | TRP | A | 61 | 75.000 | 50.730 | 76.182 | 1.00 | 1.00 |
| ATOM | 847 | CE3 | TRP | A | 61 | 73.298 | 49.483 | 75.735 | 1.00 | 1.00 |
| ATOM | 848 | HE3 | TRP | A | 61 | 72.985 | 49.405 | 76.765 | 1.00 | 1.00 |
| ATOM | 849 | CD2 | TRP | A | 61 | 72.567 | 48.797 | 74.744 | 1.00 | 1.00 |
| ATOM | 850 | C | TRP | A | 61 | 68.582 | 48.948 | 75.143 | 1.00 | 1.00 |
| ATOM | 851 | O | TRP | A | 61 | 68.124 | 48.872 | 74.006 | 1.00 | 1.00 |
| ATOM | 852 | N | ASP | A | 62 | 68.697 | 50.115 | 75.766 | 1.00 | 0.87 |
| ATOM | 853 | H | ASP | A | 62 | 69.133 | 50.169 | 76.688 | 1.00 | 0.87 |
| ATOM | 854 | CA | ASP | A | 62 | 68.239 | 51.394 | 75.211 | 1.00 | 0.87 |
| ATOM | 855 | HA | ASP | A | 62 | 67.367 | 51.203 | 74.584 | 1.00 | 0.87 |
| ATOM | 856 | CB | ASP | A | 62 | 67.770 | 52.295 | 76.367 | 1.00 | 0.87 |
| ATOM | 857 | HB1 | ASP | A | 62 | 67.002 | 51.769 | 76.937 | 1.00 | 0.87 |
| ATOM | 858 | HB2 | ASP | A | 62 | 67.310 | 53.193 | 75.950 | 1.00 | 0.87 |
| ATOM | 859 | CG | ASP | A | 62 | 68.906 | 52.692 | 77.317 | 1.00 | 0.87 |
| ATOM | 860 | OD1 | ASP | A | 62 | 69.592 | 51.760 | 77.799 | 1.00 | 0.87 |
| ATOM | 861 | OD2 | ASP | A | 62 | 69.053 | 53.909 | 77.569 | 1.00 | 0.87 |
| ATOM | 862 | C | ASP | A | 62 | 69.250 | 52.127 | 74.326 | 1.00 | 0.87 |
| ATOM | 863 | O | ASP | A | 62 | 68.930 | 53.163 | 73.732 | 1.00 | 0.87 |
| ATOM | 864 | N | GLY | A | 63 | 70.446 | 51.548 | 74.233 | 1.00 | 0.96 |
| ATOM | 865 | H | GLY | A | 63 | 70.567 | 50.678 | 74.732 | 1.00 | 0.96 |
| ATOM | 866 | CA | GLY | A | 63 | 71.649 | 52.207 | 73.778 | 1.00 | 0.96 |
| ATOM | 867 | HA1 | GLY | A | 63 | 72.438 | 51.475 | 73.659 | 1.00 | 0.96 |
| ATOM | 868 | HA2 | GLY | A | 63 | 71.941 | 52.867 | 74.592 | 1.00 | 0.96 |
| ATOM | 869 | C | GLY | A | 63 | 71.555 | 52.989 | 72.493 | 1.00 | 0.96 |
| ATOM | 870 | O | GLY | A | 63 | 70.897 | 52.538 | 71.558 | 1.00 | 0.96 |
| ATOM | 871 | N | PRO | A | 64 | 72.335 | 54.074 | 72.380 | 1.00 | 0.93 |
| ATOM | 872 | CD | PRO | A | 64 | 73.062 | 54.774 | 73.438 | 1.00 | 0.93 |
| ATOM | 873 | HD1 | PRO | A | 64 | 73.561 | 54.080 | 74.116 | 1.00 | 0.93 |
| ATOM | 874 | HD2 | PRO | A | 64 | 72.369 | 55.410 | 73.990 | 1.00 | 0.93 |
| ATOM | 875 | CG | PRO | A | 64 | 74.121 | 55.635 | 72.754 | 1.00 | 0.93 |
| ATOM | 876 | HG1 | PRO | A | 64 | 75.081 | 55.122 | 72.779 | 1.00 | 0.93 |

| | | | | | | | | | | |
|---|---|---|---|---|---|---|---|---|---|---|
| ATOM | 877 | HG2 | PRO | A | 64 | 74.211 | 56.614 | 73.224 | 1.00 | 0.93 |
| ATOM | 878 | CB | PRO | A | 64 | 73.635 | 55.740 | 71.313 | 1.00 | 0.93 |
| ATOM | 879 | HB1 | PRO | A | 64 | 74.467 | 55.824 | 70.616 | 1.00 | 0.93 |
| ATOM | 880 | HB2 | PRO | A | 64 | 72.982 | 56.602 | 71.218 | 1.00 | 0.93 |
| ATOM | 881 | CA | PRO | A | 64 | 72.800 | 54.481 | 71.081 | 1.00 | 0.93 |
| ATOM | 882 | HA | PRO | A | 64 | 71.994 | 54.771 | 70.436 | 1.00 | 0.93 |
| ATOM | 883 | C | PRO | A | 64 | 73.626 | 53.353 | 70.468 | 1.00 | 0.93 |
| ATOM | 884 | O | PRO | A | 64 | 74.699 | 53.014 | 70.969 | 1.00 | 0.93 |
| ATOM | 885 | N | LEU | A | 65 | 73.140 | 52.765 | 69.382 | 1.00 | 0.48 |
| ATOM | 886 | H | LEU | A | 65 | 72.216 | 53.043 | 69.071 | 1.00 | 0.48 |
| ATOM | 887 | CA | LEU | A | 65 | 73.927 | 51.807 | 68.606 | 1.00 | 0.48 |
| ATOM | 888 | HA | LEU | A | 65 | 74.290 | 51.054 | 69.306 | 1.00 | 0.48 |
| ATOM | 889 | CB | LEU | A | 65 | 73.029 | 51.131 | 67.548 | 1.00 | 0.48 |
| ATOM | 890 | HB1 | LEU | A | 65 | 73.444 | 51.312 | 66.556 | 1.00 | 0.48 |
| ATOM | 891 | HB2 | LEU | A | 65 | 72.037 | 51.580 | 67.581 | 1.00 | 0.48 |
| ATOM | 892 | CG | LEU | A | 65 | 72.870 | 49.616 | 67.751 | 1.00 | 0.48 |
| ATOM | 893 | HG | LEU | A | 65 | 72.460 | 49.432 | 68.745 | 1.00 | 0.48 |
| ATOM | 894 | CD1 | LEU | A | 65 | 71.899 | 49.050 | 66.707 | 1.00 | 0.48 |
| ATOM | 895 | 1HD1 | LEU | A | 65 | 71.766 | 47.982 | 66.872 | 1.00 | 0.48 |
| ATOM | 896 | 2HD1 | LEU | A | 65 | 70.927 | 49.534 | 66.813 | 1.00 | 0.48 |
| ATOM | 897 | 3HD1 | LEU | A | 65 | 72.285 | 49.213 | 65.702 | 1.00 | 0.48 |
| ATOM | 898 | CD2 | LEU | A | 65 | 74.200 | 48.873 | 67.615 | 1.00 | 0.48 |
| ATOM | 899 | 1HD2 | LEU | A | 65 | 74.060 | 47.823 | 67.861 | 1.00 | 0.48 |
| ATOM | 900 | 2HD2 | LEU | A | 65 | 74.591 | 48.963 | 66.602 | 1.00 | 0.48 |
| ATOM | 901 | 3HD2 | LEU | A | 65 | 74.944 | 49.262 | 68.311 | 1.00 | 0.48 |
| ATOM | 902 | C | LEU | A | 65 | 75.169 | 52.447 | 67.968 | 1.00 | 0.48 |
| ATOM | 903 | O | LEU | A | 65 | 76.197 | 51.797 | 67.792 | 1.00 | 0.48 |
| ATOM | 904 | N | LEU | A | 66 | 75.086 | 53.748 | 67.690 | 1.00 | 0.47 |
| ATOM | 905 | H | LEU | A | 66 | 74.171 | 54.175 | 67.799 | 1.00 | 0.47 |
| ATOM | 906 | CA | LEU | A | 66 | 76.151 | 54.632 | 67.231 | 1.00 | 0.47 |
| ATOM | 907 | HA | LEU | A | 66 | 77.114 | 54.201 | 67.508 | 1.00 | 0.47 |
| ATOM | 908 | CB | LEU | A | 66 | 76.075 | 54.706 | 65.691 | 1.00 | 0.47 |
| ATOM | 909 | HB1 | LEU | A | 66 | 75.030 | 54.874 | 65.421 | 1.00 | 0.47 |
| ATOM | 910 | HB2 | LEU | A | 66 | 76.349 | 53.719 | 65.315 | 1.00 | 0.47 |
| ATOM | 911 | CG | LEU | A | 66 | 76.909 | 55.764 | 64.929 | 1.00 | 0.47 |
| ATOM | 912 | HG | LEU | A | 66 | 76.499 | 56.757 | 65.118 | 1.00 | 0.47 |
| ATOM | 913 | CD1 | LEU | A | 66 | 78.413 | 55.784 | 65.208 | 1.00 | 0.47 |
| ATOM | 914 | 1HD1 | LEU | A | 66 | 78.804 | 54.770 | 65.192 | 1.00 | 0.47 |
| ATOM | 915 | 2HD1 | LEU | A | 66 | 78.931 | 56.388 | 64.465 | 1.00 | 0.47 |
| ATOM | 916 | 3HD1 | LEU | A | 66 | 78.611 | 56.229 | 66.177 | 1.00 | 0.47 |
| ATOM | 917 | CD2 | LEU | A | 66 | 76.771 | 55.448 | 63.437 | 1.00 | 0.47 |
| ATOM | 918 | 1HD2 | LEU | A | 66 | 77.225 | 56.237 | 62.839 | 1.00 | 0.47 |
| ATOM | 919 | 2HD2 | LEU | A | 66 | 77.253 | 54.496 | 63.211 | 1.00 | 0.47 |
| ATOM | 920 | 3HD2 | LEU | A | 66 | 75.719 | 55.362 | 63.181 | 1.00 | 0.47 |

| | | | | | | | | | | |
|---|---|---|---|---|---|---|---|---|---|---|
| ATOM | 921 | C | LEU | A | 66 | 76.022 | 55.987 | 67.914 | 1.00 | 0.47 |
| ATOM | 922 | O | LEU | A | 66 | 74.908 | 56.467 | 68.103 | 1.00 | 0.47 |
| ATOM | 923 | N | GLN | A | 67 | 77.143 | 56.620 | 68.258 | 1.00 | 0.46 |
| ATOM | 924 | H | GLN | A | 67 | 78.036 | 56.158 | 68.107 | 1.00 | 0.46 |
| ATOM | 925 | CA | GLN | A | 67 | 77.149 | 57.956 | 68.871 | 1.00 | 0.46 |
| ATOM | 926 | HA | GLN | A | 67 | 76.180 | 58.434 | 68.737 | 1.00 | 0.46 |
| ATOM | 927 | CB | GLN | A | 67 | 77.414 | 57.788 | 70.379 | 1.00 | 0.46 |
| ATOM | 928 | HB1 | GLN | A | 67 | 78.301 | 57.167 | 70.521 | 1.00 | 0.46 |
| ATOM | 929 | HB2 | GLN | A | 67 | 76.561 | 57.271 | 70.821 | 1.00 | 0.46 |
| ATOM | 930 | CG | GLN | A | 67 | 77.629 | 59.110 | 71.129 | 1.00 | 0.46 |
| ATOM | 931 | HG1 | GLN | A | 67 | 76.826 | 59.804 | 70.882 | 1.00 | 0.46 |
| ATOM | 932 | HG2 | GLN | A | 67 | 78.580 | 59.551 | 70.830 | 1.00 | 0.46 |
| ATOM | 933 | CD | GLN | A | 67 | 77.645 | 58.904 | 72.641 | 1.00 | 0.46 |
| ATOM | 934 | OE1 | GLN | A | 67 | 76.743 | 59.310 | 73.353 | 1.00 | 0.46 |
| ATOM | 935 | NE2 | GLN | A | 67 | 78.645 | 58.272 | 73.207 | 1.00 | 0.46 |
| ATOM | 936 | 1HE2 | GLN | A | 67 | 79.427 | 57.847 | 72.694 | 1.00 | 0.46 |
| ATOM | 937 | 2HE2 | GLN | A | 67 | 78.575 | 58.108 | 74.192 | 1.00 | 0.46 |
| ATOM | 938 | C | GLN | A | 67 | 78.180 | 58.836 | 68.203 | 1.00 | 0.46 |
| ATOM | 939 | O | GLN | A | 67 | 79.329 | 58.424 | 68.187 | 1.00 | 0.46 |
| ATOM | 940 | N | ILE | A | 68 | 77.807 | 60.010 | 67.671 | 1.00 | 0.53 |
| ATOM | 941 | H | ILE | A | 68 | 76.828 | 60.276 | 67.642 | 1.00 | 0.53 |
| ATOM | 942 | CA | ILE | A | 68 | 78.814 | 60.886 | 67.041 | 1.00 | 0.53 |
| ATOM | 943 | HA | ILE | A | 68 | 79.778 | 60.498 | 67.328 | 1.00 | 0.53 |
| ATOM | 944 | CB | ILE | A | 68 | 78.762 | 60.740 | 65.508 | 1.00 | 0.53 |
| ATOM | 945 | HB | ILE | A | 68 | 78.802 | 59.668 | 65.304 | 1.00 | 0.53 |
| ATOM | 946 | CG2 | ILE | A | 68 | 77.450 | 61.260 | 64.904 | 1.00 | 0.53 |
| ATOM | 947 | 1HG2 | ILE | A | 68 | 77.480 | 61.159 | 63.821 | 1.00 | 0.53 |
| ATOM | 948 | 2HG2 | ILE | A | 68 | 76.616 | 60.671 | 65.283 | 1.00 | 0.53 |
| ATOM | 949 | 3HG2 | ILE | A | 68 | 77.301 | 62.306 | 65.169 | 1.00 | 0.53 |
| ATOM | 950 | CG1 | ILE | A | 68 | 79.981 | 61.395 | 64.818 | 1.00 | 0.53 |
| ATOM | 951 | 1HG1 | ILE | A | 68 | 80.768 | 61.601 | 65.540 | 1.00 | 0.53 |
| ATOM | 952 | 2HG1 | ILE | A | 68 | 79.695 | 62.342 | 64.363 | 1.00 | 0.53 |
| ATOM | 953 | CD1 | ILE | A | 68 | 80.578 | 60.480 | 63.747 | 1.00 | 0.53 |
| ATOM | 954 | HD1 | ILE | A | 68 | 80.883 | 59.536 | 64.191 | 1.00 | 0.53 |
| ATOM | 955 | HD2 | ILE | A | 68 | 79.841 | 60.280 | 62.971 | 1.00 | 0.53 |
| ATOM | 956 | HD3 | ILE | A | 68 | 81.457 | 60.959 | 63.318 | 1.00 | 0.53 |
| ATOM | 957 | C | ILE | A | 68 | 78.798 | 62.328 | 67.562 | 1.00 | 0.53 |
| ATOM | 958 | O | ILE | A | 68 | 77.727 | 62.950 | 67.698 | 1.00 | 0.53 |
| ATOM | 959 | N | LYS | A | 69 | 80.020 | 62.808 | 67.876 | 1.00 | 0.42 |
| ATOM | 960 | H | LYS | A | 69 | 80.838 | 62.233 | 67.691 | 1.00 | 0.42 |
| ATOM | 961 | CA | LYS | A | 69 | 80.247 | 64.094 | 68.570 | 1.00 | 0.42 |
| ATOM | 962 | HA | LYS | A | 69 | 79.434 | 64.785 | 68.332 | 1.00 | 0.42 |
| ATOM | 963 | CB | LYS | A | 69 | 80.261 | 63.903 | 70.103 | 1.00 | 0.42 |
| ATOM | 964 | HB1 | LYS | A | 69 | 80.021 | 64.873 | 70.528 | 1.00 | 0.42 |

| | | | | | | | | | | |
|---|---|---|---|---|---|---|---|---|---|---|
| ATOM | 965 | HB2 | LYS | A | 69 | 81.273 | 63.658 | 70.410 | 1.00 | 0.42 |
| ATOM | 966 | CG | LYS | A | 69 | 79.322 | 62.850 | 70.731 | 1.00 | 0.42 |
| ATOM | 967 | HG1 | LYS | A | 69 | 79.462 | 61.892 | 70.236 | 1.00 | 0.42 |
| ATOM | 968 | HG2 | LYS | A | 69 | 78.283 | 63.136 | 70.589 | 1.00 | 0.42 |
| ATOM | 969 | CD | LYS | A | 69 | 79.585 | 62.614 | 72.229 | 1.00 | 0.42 |
| ATOM | 970 | HD1 | LYS | A | 69 | 80.618 | 62.302 | 72.376 | 1.00 | 0.42 |
| ATOM | 971 | HD2 | LYS | A | 69 | 78.944 | 61.791 | 72.548 | 1.00 | 0.42 |
| ATOM | 972 | CE | LYS | A | 69 | 79.293 | 63.806 | 73.145 | 1.00 | 0.42 |
| ATOM | 973 | HE1 | LYS | A | 69 | 79.168 | 63.435 | 74.164 | 1.00 | 0.42 |
| ATOM | 974 | HE2 | LYS | A | 69 | 78.354 | 64.281 | 72.838 | 1.00 | 0.42 |
| ATOM | 975 | NZ | LYS | A | 69 | 80.368 | 64.822 | 73.142 | 1.00 | 0.42 |
| ATOM | 976 | HZ1 | LYS | A | 69 | 80.126 | 65.585 | 73.766 | 1.00 | 0.42 |
| ATOM | 977 | HZ2 | LYS | A | 69 | 81.314 | 64.459 | 73.266 | 1.00 | 0.42 |
| ATOM | 978 | HZ3 | LYS | A | 69 | 80.366 | 65.350 | 72.266 | 1.00 | 0.42 |
| ATOM | 979 | C | LYS | A | 69 | 81.570 | 64.751 | 68.156 | 1.00 | 0.42 |
| ATOM | 980 | O | LYS | A | 69 | 82.553 | 64.025 | 68.004 | 1.00 | 0.42 |
| ATOM | 981 | N | GLY | A | 70 | 81.613 | 66.079 | 67.998 | 1.00 | 0.67 |
| ATOM | 982 | H | GLY | A | 70 | 80.789 | 66.630 | 68.233 | 1.00 | 0.67 |
| ATOM | 983 | CA | GLY | A | 70 | 82.823 | 66.817 | 67.570 | 1.00 | 0.67 |
| ATOM | 984 | HA1 | GLY | A | 70 | 83.516 | 66.147 | 67.059 | 1.00 | 0.67 |
| ATOM | 985 | HA2 | GLY | A | 70 | 83.336 | 67.191 | 68.456 | 1.00 | 0.67 |
| ATOM | 986 | C | GLY | A | 70 | 82.551 | 68.017 | 66.641 | 1.00 | 0.67 |
| ATOM | 987 | O | GLY | A | 70 | 81.397 | 68.238 | 66.251 | 1.00 | 0.67 |
| ATOM | 988 | N | ASN | A | 71 | 83.589 | 68.769 | 66.228 | 1.00 | 0.68 |
| ATOM | 989 | H | ASN | A | 71 | 84.540 | 68.581 | 66.549 | 1.00 | 0.68 |
| ATOM | 990 | CA | ASN | A | 71 | 83.398 | 69.919 | 65.330 | 1.00 | 0.68 |
| ATOM | 991 | HA | ASN | A | 71 | 82.339 | 70.004 | 65.128 | 1.00 | 0.68 |
| ATOM | 992 | CB | ASN | A | 71 | 83.807 | 71.256 | 65.985 | 1.00 | 0.68 |
| ATOM | 993 | HB1 | ASN | A | 71 | 83.248 | 72.062 | 65.515 | 1.00 | 0.68 |
| ATOM | 994 | HB2 | ASN | A | 71 | 84.855 | 71.448 | 65.769 | 1.00 | 0.68 |
| ATOM | 995 | CG | ASN | A | 71 | 83.597 | 71.343 | 67.483 | 1.00 | 0.68 |
| ATOM | 996 | OD1 | ASN | A | 71 | 82.476 | 71.369 | 67.977 | 1.00 | 0.68 |
| ATOM | 997 | ND2 | ASN | A | 71 | 84.662 | 71.423 | 68.243 | 1.00 | 0.68 |
| ATOM | 998 | 1HD2 | ASN | A | 71 | 85.583 | 71.303 | 67.794 | 1.00 | 0.68 |
| ATOM | 999 | 2HD2 | ASN | A | 71 | 84.535 | 71.253 | 69.218 | 1.00 | 0.68 |
| ATOM | 1000 | C | ASN | A | 71 | 84.101 | 69.811 | 63.964 | 1.00 | 0.68 |
| ATOM | 1001 | O | ASN | A | 71 | 85.237 | 69.343 | 63.836 | 1.00 | 0.68 |
| ATOM | 1002 | N | GLY | A | 72 | 83.440 | 70.309 | 62.913 | 1.00 | 0.60 |
| ATOM | 1003 | H | GLY | A | 72 | 82.509 | 70.694 | 63.012 | 1.00 | 0.60 |
| ATOM | 1004 | CA | GLY | A | 72 | 84.007 | 70.194 | 61.574 | 1.00 | 0.60 |
| ATOM | 1005 | HA1 | GLY | A | 72 | 85.014 | 70.612 | 61.577 | 1.00 | 0.60 |
| ATOM | 1006 | HA2 | GLY | A | 72 | 83.435 | 70.752 | 60.839 | 1.00 | 0.60 |
| ATOM | 1007 | C | GLY | A | 72 | 84.104 | 68.737 | 61.143 | 1.00 | 0.60 |
| ATOM | 1008 | O | GLY | A | 72 | 85.149 | 68.299 | 60.685 | 1.00 | 0.60 |

| | | | | | | | | | | |
|---|---|---|---|---|---|---|---|---|---|---|
| ATOM | 1009 | N | ILE | A | 73 | 83.066 | 67.941 | 61.398 | 1.00 | 0.47 |
| ATOM | 1010 | H | ILE | A | 73 | 82.236 | 68.350 | 61.809 | 1.00 | 0.47 |
| ATOM | 1011 | CA | ILE | A | 73 | 83.109 | 66.490 | 61.216 | 1.00 | 0.47 |
| ATOM | 1012 | HA | ILE | A | 73 | 84.142 | 66.174 | 61.067 | 1.00 | 0.47 |
| ATOM | 1013 | CB | ILE | A | 73 | 82.567 | 65.770 | 62.473 | 1.00 | 0.47 |
| ATOM | 1014 | HB | ILE | A | 73 | 81.484 | 65.898 | 62.495 | 1.00 | 0.47 |
| ATOM | 1015 | CG2 | ILE | A | 73 | 82.857 | 64.266 | 62.389 | 1.00 | 0.47 |
| ATOM | 1016 | 1HG2 | ILE | A | 73 | 82.435 | 63.765 | 63.260 | 1.00 | 0.47 |
| ATOM | 1017 | 2HG2 | ILE | A | 73 | 82.422 | 63.846 | 61.494 | 1.00 | 0.47 |
| ATOM | 1018 | 3HG2 | ILE | A | 73 | 83.934 | 64.088 | 62.357 | 1.00 | 0.47 |
| ATOM | 1019 | CG1 | ILE | A | 73 | 83.110 | 66.309 | 63.814 | 1.00 | 0.47 |
| ATOM | 1020 | 1HG1 | ILE | A | 73 | 82.643 | 65.754 | 64.629 | 1.00 | 0.47 |
| ATOM | 1021 | 2HG1 | ILE | A | 73 | 82.798 | 67.345 | 63.921 | 1.00 | 0.47 |
| ATOM | 1022 | CD1 | ILE | A | 73 | 84.633 | 66.231 | 63.991 | 1.00 | 0.47 |
| ATOM | 1023 | HD1 | ILE | A | 73 | 84.924 | 66.715 | 64.923 | 1.00 | 0.47 |
| ATOM | 1024 | HD2 | ILE | A | 73 | 84.944 | 65.194 | 64.042 | 1.00 | 0.47 |
| ATOM | 1025 | HD3 | ILE | A | 73 | 85.148 | 66.720 | 63.165 | 1.00 | 0.47 |
| ATOM | 1026 | C | ILE | A | 73 | 82.300 | 66.128 | 59.983 | 1.00 | 0.47 |
| ATOM | 1027 | O | ILE | A | 73 | 81.179 | 66.611 | 59.808 | 1.00 | 0.47 |
| ATOM | 1028 | N | THR | A | 74 | 82.827 | 65.256 | 59.124 | 1.00 | 0.45 |
| ATOM | 1029 | H | THR | A | 74 | 83.708 | 64.799 | 59.350 | 1.00 | 0.45 |
| ATOM | 1030 | CA | THR | A | 74 | 82.205 | 65.006 | 57.817 | 1.00 | 0.45 |
| ATOM | 1031 | HA | THR | A | 74 | 81.178 | 65.361 | 57.844 | 1.00 | 0.45 |
| ATOM | 1032 | CB | THR | A | 74 | 82.866 | 65.818 | 56.692 | 1.00 | 0.45 |
| ATOM | 1033 | HB | THR | A | 74 | 82.537 | 66.852 | 56.771 | 1.00 | 0.45 |
| ATOM | 1034 | CG2 | THR | A | 74 | 84.393 | 65.834 | 56.713 | 1.00 | 0.45 |
| ATOM | 1035 | 1HG2 | THR | A | 74 | 84.775 | 66.296 | 55.803 | 1.00 | 0.45 |
| ATOM | 1036 | 2HG2 | THR | A | 74 | 84.743 | 66.424 | 57.564 | 1.00 | 0.45 |
| ATOM | 1037 | 3HG2 | THR | A | 74 | 84.781 | 64.829 | 56.812 | 1.00 | 0.45 |
| ATOM | 1038 | OG1 | THR | A | 74 | 82.455 | 65.311 | 55.436 | 1.00 | 0.45 |
| ATOM | 1039 | HG1 | THR | A | 74 | 81.532 | 65.573 | 55.273 | 1.00 | 0.45 |
| ATOM | 1040 | C | THR | A | 74 | 82.124 | 63.503 | 57.580 | 1.00 | 0.45 |
| ATOM | 1041 | O | THR | A | 74 | 83.124 | 62.825 | 57.356 | 1.00 | 0.45 |
| ATOM | 1042 | N | ILE | A | 75 | 80.909 | 62.977 | 57.663 | 1.00 | 0.40 |
| ATOM | 1043 | H | ILE | A | 75 | 80.131 | 63.629 | 57.667 | 1.00 | 0.40 |
| ATOM | 1044 | CA | ILE | A | 75 | 80.506 | 61.602 | 57.875 | 1.00 | 0.40 |
| ATOM | 1045 | HA | ILE | A | 75 | 81.398 | 61.014 | 57.830 | 1.00 | 0.40 |
| ATOM | 1046 | CB | ILE | A | 75 | 79.978 | 61.344 | 59.304 | 1.00 | 0.40 |
| ATOM | 1047 | HB | ILE | A | 75 | 80.034 | 60.266 | 59.467 | 1.00 | 0.40 |
| ATOM | 1048 | CG2 | ILE | A | 75 | 80.913 | 62.022 | 60.317 | 1.00 | 0.40 |
| ATOM | 1049 | 1HG2 | ILE | A | 75 | 80.741 | 61.627 | 61.310 | 1.00 | 0.40 |
| ATOM | 1050 | 2HG2 | ILE | A | 75 | 81.958 | 61.851 | 60.059 | 1.00 | 0.40 |
| ATOM | 1051 | 3HG2 | ILE | A | 75 | 80.714 | 63.093 | 60.330 | 1.00 | 0.40 |
| ATOM | 1052 | CG1 | ILE | A | 75 | 78.522 | 61.770 | 59.557 | 1.00 | 0.40 |

| | | | | | | | | | | |
|---|---|---|---|---|---|---|---|---|---|---|
| ATOM | 1053 | 1HG1 | ILE | A | 75 | 77.883 | 61.238 | 58.856 | 1.00 | 0.40 |
| ATOM | 1054 | 2HG1 | ILE | A | 75 | 78.425 | 62.843 | 59.394 | 1.00 | 0.40 |
| ATOM | 1055 | CD1 | ILE | A | 75 | 78.027 | 61.432 | 60.968 | 1.00 | 0.40 |
| ATOM | 1056 | HD1 | ILE | A | 75 | 78.261 | 60.394 | 61.202 | 1.00 | 0.40 |
| ATOM | 1057 | HD2 | ILE | A | 75 | 78.494 | 62.091 | 61.701 | 1.00 | 0.40 |
| ATOM | 1058 | HD3 | ILE | A | 75 | 76.949 | 61.567 | 61.023 | 1.00 | 0.40 |
| ATOM | 1059 | C | ILE | A | 75 | 79.580 | 61.134 | 56.743 | 1.00 | 0.40 |
| ATOM | 1060 | O | ILE | A | 75 | 78.573 | 61.809 | 56.448 | 1.00 | 0.40 |
| ATOM | 1061 | N | LYS | A | 76 | 80.037 | 60.049 | 56.072 | 1.00 | 0.45 |
| ATOM | 1062 | H | LYS | A | 76 | 80.780 | 59.503 | 56.498 | 1.00 | 0.45 |
| ATOM | 1063 | CA | LYS | A | 76 | 79.891 | 59.892 | 54.607 | 1.00 | 0.45 |
| ATOM | 1064 | HA | LYS | A | 76 | 78.975 | 60.373 | 54.267 | 1.00 | 0.45 |
| ATOM | 1065 | CB | LYS | A | 76 | 81.106 | 60.522 | 53.885 | 1.00 | 0.45 |
| ATOM | 1066 | HB1 | LYS | A | 76 | 81.058 | 60.229 | 52.834 | 1.00 | 0.45 |
| ATOM | 1067 | HB2 | LYS | A | 76 | 82.026 | 60.111 | 54.303 | 1.00 | 0.45 |
| ATOM | 1068 | CG | LYS | A | 76 | 81.174 | 62.055 | 53.939 | 1.00 | 0.45 |
| ATOM | 1069 | HG1 | LYS | A | 76 | 81.320 | 62.391 | 54.964 | 1.00 | 0.45 |
| ATOM | 1070 | HG2 | LYS | A | 76 | 80.226 | 62.444 | 53.581 | 1.00 | 0.45 |
| ATOM | 1071 | CD | LYS | A | 76 | 82.283 | 62.638 | 53.044 | 1.00 | 0.45 |
| ATOM | 1072 | HD1 | LYS | A | 76 | 82.127 | 63.715 | 52.961 | 1.00 | 0.45 |
| ATOM | 1073 | HD2 | LYS | A | 76 | 82.170 | 62.221 | 52.042 | 1.00 | 0.45 |
| ATOM | 1074 | CE | LYS | A | 76 | 83.719 | 62.383 | 53.527 | 1.00 | 0.45 |
| ATOM | 1075 | HE1 | LYS | A | 76 | 84.403 | 62.708 | 52.739 | 1.00 | 0.45 |
| ATOM | 1076 | HE2 | LYS | A | 76 | 83.866 | 61.310 | 53.685 | 1.00 | 0.45 |
| ATOM | 1077 | NZ | LYS | A | 76 | 84.029 | 63.132 | 54.765 | 1.00 | 0.45 |
| ATOM | 1078 | HZ1 | LYS | A | 76 | 85.025 | 63.158 | 54.981 | 1.00 | 0.45 |
| ATOM | 1079 | HZ2 | LYS | A | 76 | 83.674 | 64.087 | 54.725 | 1.00 | 0.45 |
| ATOM | 1080 | HZ3 | LYS | A | 76 | 83.558 | 62.716 | 55.567 | 1.00 | 0.45 |
| ATOM | 1081 | C | LYS | A | 76 | 79.837 | 58.416 | 54.210 | 1.00 | 0.45 |
| ATOM | 1082 | O | LYS | A | 76 | 80.852 | 57.720 | 54.313 | 1.00 | 0.45 |
| ATOM | 1083 | N | GLY | A | 77 | 78.704 | 57.926 | 53.717 | 1.00 | 0.76 |
| ATOM | 1084 | H | GLY | A | 77 | 77.886 | 58.513 | 53.611 | 1.00 | 0.76 |
| ATOM | 1085 | CA | GLY | A | 77 | 78.713 | 56.530 | 53.241 | 1.00 | 0.76 |
| ATOM | 1086 | HA1 | GLY | A | 77 | 77.688 | 56.220 | 53.041 | 1.00 | 0.76 |
| ATOM | 1087 | HA2 | GLY | A | 77 | 79.112 | 55.882 | 54.023 | 1.00 | 0.76 |
| ATOM | 1088 | C | GLY | A | 77 | 79.525 | 56.311 | 51.956 | 1.00 | 0.76 |
| ATOM | 1089 | O | GLY | A | 77 | 79.808 | 57.255 | 51.215 | 1.00 | 0.76 |
| ATOM | 1090 | N | ALA | A | 78 | 79.862 | 55.050 | 51.681 | 1.00 | 1.29 |
| ATOM | 1091 | H | ALA | A | 78 | 79.651 | 54.344 | 52.376 | 1.00 | 1.29 |
| ATOM | 1092 | CA | ALA | A | 78 | 80.723 | 54.643 | 50.578 | 1.00 | 1.29 |
| ATOM | 1093 | HA | ALA | A | 78 | 80.972 | 55.516 | 49.974 | 1.00 | 1.29 |
| ATOM | 1094 | CB | ALA | A | 78 | 82.044 | 54.101 | 51.135 | 1.00 | 1.29 |
| ATOM | 1095 | HB1 | ALA | A | 78 | 82.665 | 53.728 | 50.320 | 1.00 | 1.29 |
| ATOM | 1096 | HB2 | ALA | A | 78 | 82.578 | 54.902 | 51.637 | 1.00 | 1.29 |

| | | | | | | | | | | |
|---|---|---|---|---|---|---|---|---|---|---|
| ATOM | 1097 | HB3 | ALA | A | 78 | 81.856 | 53.287 | 51.834 | 1.00 | 1.29 |
| ATOM | 1098 | C | ALA | A | 78 | 80.044 | 53.628 | 49.654 | 1.00 | 1.29 |
| ATOM | 1099 | O | ALA | A | 78 | 79.883 | 52.462 | 50.008 | 1.00 | 1.29 |
| ATOM | 1100 | N | ASP | A | 79 | 79.700 | 54.087 | 48.452 | 1.00 | 1.63 |
| ATOM | 1101 | H | ASP | A | 79 | 79.858 | 55.062 | 48.262 | 1.00 | 1.63 |
| ATOM | 1102 | CA | ASP | A | 79 | 79.104 | 53.296 | 47.373 | 1.00 | 1.63 |
| ATOM | 1103 | HA | ASP | A | 79 | 78.665 | 53.991 | 46.656 | 1.00 | 1.63 |
| ATOM | 1104 | CB | ASP | A | 79 | 80.236 | 52.568 | 46.621 | 1.00 | 1.63 |
| ATOM | 1105 | HB1 | ASP | A | 79 | 80.782 | 51.929 | 47.318 | 1.00 | 1.63 |
| ATOM | 1106 | HB2 | ASP | A | 79 | 80.938 | 53.310 | 46.234 | 1.00 | 1.63 |
| ATOM | 1107 | CG | ASP | A | 79 | 79.723 | 51.721 | 45.456 | 1.00 | 1.63 |
| ATOM | 1108 | OD1 | ASP | A | 79 | 79.340 | 52.323 | 44.431 | 1.00 | 1.63 |
| ATOM | 1109 | OD2 | ASP | A | 79 | 79.702 | 50.482 | 45.640 | 1.00 | 1.63 |
| ATOM | 1110 | C | ASP | A | 79 | 77.928 | 52.408 | 47.826 | 1.00 | 1.63 |
| ATOM | 1111 | O | ASP | A | 79 | 76.835 | 52.919 | 48.059 | 1.00 | 1.63 |
| ATOM | 1112 | N | GLY | A | 80 | 78.135 | 51.102 | 47.990 | 1.00 | 1.21 |
| ATOM | 1113 | H | GLY | A | 80 | 79.024 | 50.729 | 47.673 | 1.00 | 1.21 |
| ATOM | 1114 | CA | GLY | A | 80 | 77.097 | 50.152 | 48.389 | 1.00 | 1.21 |
| ATOM | 1115 | HA1 | GLY | A | 80 | 77.424 | 49.152 | 48.108 | 1.00 | 1.21 |
| ATOM | 1116 | HA2 | GLY | A | 80 | 76.187 | 50.375 | 47.832 | 1.00 | 1.21 |
| ATOM | 1117 | C | GLY | A | 80 | 76.739 | 50.128 | 49.872 | 1.00 | 1.21 |
| ATOM | 1118 | O | GLY | A | 80 | 75.883 | 49.338 | 50.277 | 1.00 | 1.21 |
| ATOM | 1119 | N | ALA | A | 81 | 77.435 | 50.919 | 50.688 | 1.00 | 0.92 |
| ATOM | 1120 | H | ALA | A | 81 | 78.129 | 51.534 | 50.277 | 1.00 | 0.92 |
| ATOM | 1121 | CA | ALA | A | 81 | 77.438 | 50.767 | 52.133 | 1.00 | 0.92 |
| ATOM | 1122 | HA | ALA | A | 81 | 77.808 | 49.764 | 52.331 | 1.00 | 0.92 |
| ATOM | 1123 | CB | ALA | A | 81 | 78.416 | 51.769 | 52.747 | 1.00 | 0.92 |
| ATOM | 1124 | HB1 | ALA | A | 81 | 78.453 | 51.632 | 53.828 | 1.00 | 0.92 |
| ATOM | 1125 | HB2 | ALA | A | 81 | 79.413 | 51.605 | 52.343 | 1.00 | 0.92 |
| ATOM | 1126 | HB3 | ALA | A | 81 | 78.096 | 52.787 | 52.522 | 1.00 | 0.92 |
| ATOM | 1127 | C | ALA | A | 81 | 76.056 | 50.896 | 52.779 | 1.00 | 0.92 |
| ATOM | 1128 | O | ALA | A | 81 | 75.289 | 51.816 | 52.486 | 1.00 | 0.92 |
| ATOM | 1129 | N | LYS | A | 82 | 75.784 | 50.014 | 53.747 | 1.00 | 0.60 |
| ATOM | 1130 | H | LYS | A | 82 | 76.484 | 49.309 | 53.963 | 1.00 | 0.60 |
| ATOM | 1131 | CA | LYS | A | 82 | 74.588 | 50.102 | 54.581 | 1.00 | 0.60 |
| ATOM | 1132 | HA | LYS | A | 82 | 74.332 | 51.162 | 54.638 | 1.00 | 0.60 |
| ATOM | 1133 | CB | LYS | A | 82 | 73.372 | 49.428 | 53.925 | 1.00 | 0.60 |
| ATOM | 1134 | HB1 | LYS | A | 82 | 73.068 | 50.050 | 53.082 | 1.00 | 0.60 |
| ATOM | 1135 | HB2 | LYS | A | 82 | 72.561 | 49.430 | 54.653 | 1.00 | 0.60 |
| ATOM | 1136 | CG | LYS | A | 82 | 73.591 | 47.994 | 53.413 | 1.00 | 0.60 |
| ATOM | 1137 | HG1 | LYS | A | 82 | 74.073 | 47.397 | 54.188 | 1.00 | 0.60 |
| ATOM | 1138 | HG2 | LYS | A | 82 | 74.259 | 48.035 | 52.552 | 1.00 | 0.60 |
| ATOM | 1139 | CD | LYS | A | 82 | 72.296 | 47.272 | 52.987 | 1.00 | 0.60 |
| ATOM | 1140 | HD1 | LYS | A | 82 | 71.793 | 46.882 | 53.874 | 1.00 | 0.60 |

| | | | | | | | | | | |
|---|---|---|---|---|---|---|---|---|---|---|
| ATOM | 1141 | HD2 | LYS | A | 82 | 72.582 | 46.417 | 52.373 | 1.00 | 0.60 |
| ATOM | 1142 | CE | LYS | A | 82 | 71.314 | 48.142 | 52.182 | 1.00 | 0.60 |
| ATOM | 1143 | HE1 | LYS | A | 82 | 70.760 | 47.507 | 51.486 | 1.00 | 0.60 |
| ATOM | 1144 | HE2 | LYS | A | 82 | 71.885 | 48.872 | 51.602 | 1.00 | 0.60 |
| ATOM | 1145 | NZ | LYS | A | 82 | 70.349 | 48.826 | 53.073 | 1.00 | 0.60 |
| ATOM | 1146 | HZ1 | LYS | A | 82 | 69.914 | 49.640 | 52.666 | 1.00 | 0.60 |
| ATOM | 1147 | HZ2 | LYS | A | 82 | 69.611 | 48.193 | 53.401 | 1.00 | 0.60 |
| ATOM | 1148 | HZ3 | LYS | A | 82 | 70.763 | 49.070 | 53.971 | 1.00 | 0.60 |
| ATOM | 1149 | C | LYS | A | 82 | 74.763 | 49.646 | 56.035 | 1.00 | 0.60 |
| ATOM | 1150 | O | LYS | A | 82 | 75.235 | 48.557 | 56.356 | 1.00 | 0.60 |
| ATOM | 1151 | N | LEU | A | 83 | 74.349 | 50.548 | 56.913 | 1.00 | 0.48 |
| ATOM | 1152 | H | LEU | A | 83 | 73.933 | 51.370 | 56.502 | 1.00 | 0.48 |
| ATOM | 1153 | CA | LEU | A | 83 | 74.066 | 50.413 | 58.313 | 1.00 | 0.48 |
| ATOM | 1154 | HA | LEU | A | 83 | 74.876 | 49.874 | 58.805 | 1.00 | 0.48 |
| ATOM | 1155 | CB | LEU | A | 83 | 73.941 | 51.825 | 58.922 | 1.00 | 0.48 |
| ATOM | 1156 | HB1 | LEU | A | 83 | 73.448 | 51.729 | 59.889 | 1.00 | 0.48 |
| ATOM | 1157 | HB2 | LEU | A | 83 | 73.290 | 52.430 | 58.291 | 1.00 | 0.48 |
| ATOM | 1158 | CG | LEU | A | 83 | 75.267 | 52.586 | 59.148 | 1.00 | 0.48 |
| ATOM | 1159 | HG | LEU | A | 83 | 75.843 | 52.058 | 59.909 | 1.00 | 0.48 |
| ATOM | 1160 | CD1 | LEU | A | 83 | 76.151 | 52.766 | 57.909 | 1.00 | 0.48 |
| ATOM | 1161 | 1HD1 | LEU | A | 83 | 76.978 | 53.434 | 58.146 | 1.00 | 0.48 |
| ATOM | 1162 | 2HD1 | LEU | A | 83 | 76.576 | 51.809 | 57.606 | 1.00 | 0.48 |
| ATOM | 1163 | 3HD1 | LEU | A | 83 | 75.574 | 53.191 | 57.088 | 1.00 | 0.48 |
| ATOM | 1164 | CD2 | LEU | A | 83 | 74.971 | 53.996 | 59.658 | 1.00 | 0.48 |
| ATOM | 1165 | 1HD2 | LEU | A | 83 | 75.909 | 54.507 | 59.883 | 1.00 | 0.48 |
| ATOM | 1166 | 2HD2 | LEU | A | 83 | 74.424 | 54.562 | 58.904 | 1.00 | 0.48 |
| ATOM | 1167 | 3HD2 | LEU | A | 83 | 74.385 | 53.950 | 60.571 | 1.00 | 0.48 |
| ATOM | 1168 | C | LEU | A | 83 | 72.770 | 49.627 | 58.465 | 1.00 | 0.48 |
| ATOM | 1169 | O | LEU | A | 83 | 71.726 | 50.070 | 57.999 | 1.00 | 0.48 |
| ATOM | 1170 | N | ASN | A | 84 | 72.827 | 48.487 | 59.138 | 1.00 | 0.71 |
| ATOM | 1171 | H | ASN | A | 84 | 73.725 | 48.233 | 59.536 | 1.00 | 0.71 |
| ATOM | 1172 | CA | ASN | A | 84 | 71.864 | 47.400 | 58.983 | 1.00 | 0.71 |
| ATOM | 1173 | HA | ASN | A | 84 | 71.005 | 47.730 | 58.427 | 1.00 | 0.71 |
| ATOM | 1174 | CB | ASN | A | 84 | 72.615 | 46.368 | 58.100 | 1.00 | 0.71 |
| ATOM | 1175 | HB1 | ASN | A | 84 | 73.584 | 46.165 | 58.558 | 1.00 | 0.71 |
| ATOM | 1176 | HB2 | ASN | A | 84 | 72.796 | 46.817 | 57.123 | 1.00 | 0.71 |
| ATOM | 1177 | CG | ASN | A | 84 | 71.918 | 45.046 | 57.863 | 1.00 | 0.71 |
| ATOM | 1178 | OD1 | ASN | A | 84 | 70.707 | 44.913 | 57.867 | 1.00 | 0.71 |
| ATOM | 1179 | ND2 | ASN | A | 84 | 72.677 | 43.991 | 57.641 | 1.00 | 0.71 |
| ATOM | 1180 | 1HD2 | ASN | A | 84 | 73.681 | 44.059 | 57.666 | 1.00 | 0.71 |
| ATOM | 1181 | 2HD2 | ASN | A | 84 | 72.194 | 43.115 | 57.553 | 1.00 | 0.71 |
| ATOM | 1182 | C | ASN | A | 84 | 71.415 | 46.880 | 60.358 | 1.00 | 0.71 |
| ATOM | 1183 | O | ASN | A | 84 | 71.847 | 45.799 | 60.768 | 1.00 | 0.71 |
| ATOM | 1184 | N | PRO | A | 85 | 70.643 | 47.675 | 61.137 | 1.00 | 0.89 |

| | | | | | | | | | | |
|---|---|---|---|---|---|---|---|---|---|---|
| ATOM | 1185 | CD | PRO | A | 85 | 70.205 | 49.044 | 60.897 | 1.00 | 0.89 |
| ATOM | 1186 | HD1 | PRO | A | 85 | 69.154 | 49.043 | 60.603 | 1.00 | 0.89 |
| ATOM | 1187 | HD2 | PRO | A | 85 | 70.810 | 49.582 | 60.187 | 1.00 | 0.89 |
| ATOM | 1188 | CG | PRO | A | 85 | 70.301 | 49.737 | 62.244 | 1.00 | 0.89 |
| ATOM | 1189 | HG1 | PRO | A | 85 | 69.698 | 50.645 | 62.286 | 1.00 | 0.89 |
| ATOM | 1190 | HG2 | PRO | A | 85 | 71.349 | 49.933 | 62.463 | 1.00 | 0.89 |
| ATOM | 1191 | CB | PRO | A | 85 | 69.776 | 48.645 | 63.157 | 1.00 | 0.89 |
| ATOM | 1192 | HB1 | PRO | A | 85 | 68.693 | 48.646 | 63.133 | 1.00 | 0.89 |
| ATOM | 1193 | HB2 | PRO | A | 85 | 70.133 | 48.766 | 64.179 | 1.00 | 0.89 |
| ATOM | 1194 | CA | PRO | A | 85 | 70.306 | 47.357 | 62.515 | 1.00 | 0.89 |
| ATOM | 1195 | HA | PRO | A | 85 | 71.239 | 47.128 | 63.023 | 1.00 | 0.89 |
| ATOM | 1196 | C | PRO | A | 85 | 69.368 | 46.173 | 62.753 | 1.00 | 0.89 |
| ATOM | 1197 | O | PRO | A | 85 | 68.871 | 46.078 | 63.871 | 1.00 | 0.89 |
| ATOM | 1198 | N | ASP | A | 86 | 69.117 | 45.331 | 61.733 | 1.00 | 1.03 |
| ATOM | 1199 | H | ASP | A | 86 | 69.666 | 45.467 | 60.897 | 1.00 | 1.03 |
| ATOM | 1200 | CA | ASP | A | 86 | 68.154 | 44.211 | 61.690 | 1.00 | 1.03 |
| ATOM | 1201 | HA | ASP | A | 86 | 67.585 | 44.365 | 60.773 | 1.00 | 1.03 |
| ATOM | 1202 | CB | ASP | A | 86 | 68.848 | 42.854 | 61.462 | 1.00 | 1.03 |
| ATOM | 1203 | HB1 | ASP | A | 86 | 69.736 | 42.779 | 62.078 | 1.00 | 1.03 |
| ATOM | 1204 | HB2 | ASP | A | 86 | 69.186 | 42.808 | 60.426 | 1.00 | 1.03 |
| ATOM | 1205 | CG | ASP | A | 86 | 67.963 | 41.628 | 61.740 | 1.00 | 1.03 |
| ATOM | 1206 | OD1 | ASP | A | 86 | 66.775 | 41.646 | 61.329 | 1.00 | 1.03 |
| ATOM | 1207 | OD2 | ASP | A | 86 | 68.504 | 40.698 | 62.390 | 1.00 | 1.03 |
| ATOM | 1208 | C | ASP | A | 86 | 67.064 | 44.266 | 62.786 | 1.00 | 1.03 |
| ATOM | 1209 | O | ASP | A | 86 | 66.861 | 43.375 | 63.622 | 1.00 | 1.03 |
| ATOM | 1210 | N | GLY | A | 87 | 66.352 | 45.389 | 62.805 | 1.00 | 1.05 |
| ATOM | 1211 | H | GLY | A | 87 | 66.638 | 46.130 | 62.178 | 1.00 | 1.05 |
| ATOM | 1212 | CA | GLY | A | 87 | 65.495 | 45.760 | 63.918 | 1.00 | 1.05 |
| ATOM | 1213 | HA1 | GLY | A | 87 | 65.106 | 46.761 | 63.733 | 1.00 | 1.05 |
| ATOM | 1214 | HA2 | GLY | A | 87 | 66.088 | 45.781 | 64.828 | 1.00 | 1.05 |
| ATOM | 1215 | C | GLY | A | 87 | 64.303 | 44.844 | 64.129 | 1.00 | 1.05 |
| ATOM | 1216 | O | GLY | A | 87 | 63.719 | 44.879 | 65.207 | 1.00 | 1.05 |
| ATOM | 1217 | N | SER | A | 88 | 64.020 | 43.974 | 63.156 | 1.00 | 0.93 |
| ATOM | 1218 | H | SER | A | 88 | 64.642 | 43.973 | 62.361 | 1.00 | 0.93 |
| ATOM | 1219 | CA | SER | A | 88 | 63.087 | 42.856 | 63.291 | 1.00 | 0.93 |
| ATOM | 1220 | HA | SER | A | 88 | 62.076 | 43.250 | 63.323 | 1.00 | 0.93 |
| ATOM | 1221 | CB | SER | A | 88 | 63.207 | 41.922 | 62.071 | 1.00 | 0.93 |
| ATOM | 1222 | HB1 | SER | A | 88 | 63.286 | 42.511 | 61.156 | 1.00 | 0.93 |
| ATOM | 1223 | HB2 | SER | A | 88 | 62.287 | 41.339 | 62.012 | 1.00 | 0.93 |
| ATOM | 1224 | OG | SER | A | 88 | 64.288 | 40.999 | 62.146 | 1.00 | 0.93 |
| ATOM | 1225 | HG | SER | A | 88 | 65.149 | 41.367 | 61.835 | 1.00 | 0.93 |
| ATOM | 1226 | C | SER | A | 88 | 63.291 | 42.083 | 64.581 | 1.00 | 0.93 |
| ATOM | 1227 | O | SER | A | 88 | 62.325 | 41.830 | 65.298 | 1.00 | 0.93 |
| ATOM | 1228 | N | ARG | A | 89 | 64.553 | 41.816 | 64.938 | 1.00 | 0.89 |

| | | | | | | | | | | |
|---|---|---|---|---|---|---|---|---|---|---|
| ATOM | 1229 | H | ARG | A | 89 | 65.294 | 42.093 | 64.299 | 1.00 | 0.89 |
| ATOM | 1230 | CA | ARG | A | 89 | 64.903 | 41.040 | 66.131 | 1.00 | 0.89 |
| ATOM | 1231 | HA | ARG | A | 89 | 64.333 | 40.111 | 66.108 | 1.00 | 0.89 |
| ATOM | 1232 | CB | ARG | A | 89 | 66.402 | 40.707 | 66.076 | 1.00 | 0.89 |
| ATOM | 1233 | HB1 | ARG | A | 89 | 66.767 | 40.578 | 67.096 | 1.00 | 0.89 |
| ATOM | 1234 | HB2 | ARG | A | 89 | 66.906 | 41.552 | 65.613 | 1.00 | 0.89 |
| ATOM | 1235 | CG | ARG | A | 89 | 66.749 | 39.437 | 65.278 | 1.00 | 0.89 |
| ATOM | 1236 | HG1 | ARG | A | 89 | 66.652 | 39.630 | 64.208 | 1.00 | 0.89 |
| ATOM | 1237 | HG2 | ARG | A | 89 | 66.029 | 38.664 | 65.548 | 1.00 | 0.89 |
| ATOM | 1238 | CD | ARG | A | 89 | 68.155 | 38.885 | 65.601 | 1.00 | 0.89 |
| ATOM | 1239 | HD1 | ARG | A | 89 | 68.168 | 37.828 | 65.327 | 1.00 | 0.89 |
| ATOM | 1240 | HD2 | ARG | A | 89 | 68.324 | 38.956 | 66.677 | 1.00 | 0.89 |
| ATOM | 1241 | NE | ARG | A | 89 | 69.252 | 39.548 | 64.865 | 1.00 | 0.89 |
| ATOM | 1242 | HE | ARG | A | 89 | 68.998 | 39.996 | 63.978 | 1.00 | 0.89 |
| ATOM | 1243 | CZ | ARG | A | 89 | 70.550 | 39.516 | 65.157 | 1.00 | 0.89 |
| ATOM | 1244 | NH1 | ARG | A | 89 | 71.016 | 38.953 | 66.237 | 1.00 | 0.89 |
| ATOM | 1245 | 1HH1 | ARG | A | 89 | 70.397 | 38.564 | 66.911 | 1.00 | 0.89 |
| ATOM | 1246 | 2HH1 | ARG | A | 89 | 72.026 | 39.004 | 66.432 | 1.00 | 0.89 |
| ATOM | 1247 | NH2 | ARG | A | 89 | 71.433 | 40.036 | 64.361 | 1.00 | 0.89 |
| ATOM | 1248 | 1HH2 | ARG | A | 89 | 71.128 | 40.579 | 63.575 | 1.00 | 0.89 |
| ATOM | 1249 | 2HH2 | ARG | A | 89 | 72.425 | 39.943 | 64.611 | 1.00 | 0.89 |
| ATOM | 1250 | C | ARG | A | 89 | 64.520 | 41.689 | 67.466 | 1.00 | 0.89 |
| ATOM | 1251 | O | ARG | A | 89 | 64.527 | 40.998 | 68.479 | 1.00 | 0.89 |
| ATOM | 1252 | N | TRP | A | 90 | 64.151 | 42.972 | 67.477 | 1.00 | 0.76 |
| ATOM | 1253 | H | TRP | A | 90 | 64.153 | 43.469 | 66.595 | 1.00 | 0.76 |
| ATOM | 1254 | CA | TRP | A | 90 | 63.556 | 43.653 | 68.632 | 1.00 | 0.76 |
| ATOM | 1255 | HA | TRP | A | 90 | 63.532 | 42.988 | 69.497 | 1.00 | 0.76 |
| ATOM | 1256 | CB | TRP | A | 90 | 64.380 | 44.903 | 68.995 | 1.00 | 0.76 |
| ATOM | 1257 | HB1 | TRP | A | 90 | 63.818 | 45.475 | 69.733 | 1.00 | 0.76 |
| ATOM | 1258 | HB2 | TRP | A | 90 | 64.440 | 45.532 | 68.106 | 1.00 | 0.76 |
| ATOM | 1259 | CG | TRP | A | 90 | 65.761 | 44.765 | 69.562 | 1.00 | 0.76 |
| ATOM | 1260 | CD1 | TRP | A | 90 | 66.062 | 44.567 | 70.867 | 1.00 | 0.76 |
| ATOM | 1261 | HD1 | TRP | A | 90 | 65.343 | 44.392 | 71.656 | 1.00 | 0.76 |
| ATOM | 1262 | NE1 | TRP | A | 90 | 67.424 | 44.696 | 71.059 | 1.00 | 0.76 |
| ATOM | 1263 | HE1 | TRP | A | 90 | 67.888 | 44.624 | 71.965 | 1.00 | 0.76 |
| ATOM | 1264 | CE2 | TRP | A | 90 | 68.052 | 45.100 | 69.911 | 1.00 | 0.76 |
| ATOM | 1265 | CZ2 | TRP | A | 90 | 69.354 | 45.542 | 69.649 | 1.00 | 0.76 |
| ATOM | 1266 | HZ2 | TRP | A | 90 | 70.085 | 45.581 | 70.443 | 1.00 | 0.76 |
| ATOM | 1267 | CH2 | TRP | A | 90 | 69.661 | 45.998 | 68.360 | 1.00 | 0.76 |
| ATOM | 1268 | HH2 | TRP | A | 90 | 70.648 | 46.377 | 68.139 | 1.00 | 0.76 |
| ATOM | 1269 | CZ3 | TRP | A | 90 | 68.716 | 45.869 | 67.328 | 1.00 | 0.76 |
| ATOM | 1270 | HZ3 | TRP | A | 90 | 68.990 | 46.128 | 66.316 | 1.00 | 0.76 |
| ATOM | 1271 | CE3 | TRP | A | 90 | 67.427 | 45.373 | 67.598 | 1.00 | 0.76 |
| ATOM | 1272 | HE3 | TRP | A | 90 | 66.707 | 45.273 | 66.800 | 1.00 | 0.76 |

| | | | | | | | | | | |
|---|---|---|---|---|---|---|---|---|---|---|
| ATOM | 1273 | CD2 | TRP | A | 90 | 67.036 | 45.057 | 68.914 | 1.00 | 0.76 |
| ATOM | 1274 | C | TRP | A | 90 | 62.116 | 44.123 | 68.378 | 1.00 | 0.76 |
| ATOM | 1275 | O | TRP | A | 90 | 61.473 | 44.594 | 69.312 | 1.00 | 0.76 |
| ATOM | 1276 | N | TRP | A | 91 | 61.604 | 44.094 | 67.147 | 1.00 | 0.79 |
| ATOM | 1277 | H | TRP | A | 91 | 62.112 | 43.643 | 66.398 | 1.00 | 0.79 |
| ATOM | 1278 | CA | TRP | A | 91 | 60.263 | 44.585 | 66.859 | 1.00 | 0.79 |
| ATOM | 1279 | HA | TRP | A | 91 | 60.123 | 45.554 | 67.334 | 1.00 | 0.79 |
| ATOM | 1280 | CB | TRP | A | 91 | 60.044 | 44.745 | 65.358 | 1.00 | 0.79 |
| ATOM | 1281 | HB1 | TRP | A | 91 | 58.979 | 44.890 | 65.201 | 1.00 | 0.79 |
| ATOM | 1282 | HB2 | TRP | A | 91 | 60.316 | 43.812 | 64.862 | 1.00 | 0.79 |
| ATOM | 1283 | CG | TRP | A | 91 | 60.730 | 45.882 | 64.672 | 1.00 | 0.79 |
| ATOM | 1284 | CD1 | TRP | A | 91 | 61.192 | 47.022 | 65.233 | 1.00 | 0.79 |
| ATOM | 1285 | HD1 | TRP | A | 91 | 61.154 | 47.265 | 66.285 | 1.00 | 0.79 |
| ATOM | 1286 | NE1 | TRP | A | 91 | 61.747 | 47.830 | 64.259 | 1.00 | 0.79 |
| ATOM | 1287 | HE1 | TRP | A | 91 | 62.204 | 48.715 | 64.478 | 1.00 | 0.79 |
| ATOM | 1288 | CE2 | TRP | A | 91 | 61.712 | 47.227 | 63.025 | 1.00 | 0.79 |
| ATOM | 1289 | CZ2 | TRP | A | 91 | 62.169 | 47.607 | 61.758 | 1.00 | 0.79 |
| ATOM | 1290 | HZ2 | TRP | A | 91 | 62.661 | 48.558 | 61.617 | 1.00 | 0.79 |
| ATOM | 1291 | CH2 | TRP | A | 91 | 61.968 | 46.726 | 60.686 | 1.00 | 0.79 |
| ATOM | 1292 | HH2 | TRP | A | 91 | 62.273 | 47.008 | 59.699 | 1.00 | 0.79 |
| ATOM | 1293 | CZ3 | TRP | A | 91 | 61.325 | 45.492 | 60.881 | 1.00 | 0.79 |
| ATOM | 1294 | HZ3 | TRP | A | 91 | 61.161 | 44.825 | 60.047 | 1.00 | 0.79 |
| ATOM | 1295 | CE3 | TRP | A | 91 | 60.843 | 45.142 | 62.152 | 1.00 | 0.79 |
| ATOM | 1296 | HE3 | TRP | A | 91 | 60.312 | 44.210 | 62.300 | 1.00 | 0.79 |
| ATOM | 1297 | CD2 | TRP | A | 91 | 61.043 | 45.990 | 63.256 | 1.00 | 0.79 |
| ATOM | 1298 | C | TRP | A | 91 | 59.222 | 43.617 | 67.427 | 1.00 | 0.79 |
| ATOM | 1299 | O | TRP | A | 91 | 59.259 | 42.407 | 67.217 | 1.00 | 0.79 |
| ATOM | 1300 | N | ASP | A | 92 | 58.281 | 44.186 | 68.166 | 1.00 | 1.03 |
| ATOM | 1301 | H | ASP | A | 92 | 58.358 | 45.173 | 68.351 | 1.00 | 1.03 |
| ATOM | 1302 | CA | ASP | A | 92 | 57.302 | 43.455 | 68.967 | 1.00 | 1.03 |
| ATOM | 1303 | HA | ASP | A | 92 | 57.198 | 42.445 | 68.569 | 1.00 | 1.03 |
| ATOM | 1304 | CB | ASP | A | 92 | 57.818 | 43.337 | 70.409 | 1.00 | 1.03 |
| ATOM | 1305 | HB1 | ASP | A | 92 | 58.760 | 42.785 | 70.395 | 1.00 | 1.03 |
| ATOM | 1306 | HB2 | ASP | A | 92 | 57.106 | 42.754 | 70.996 | 1.00 | 1.03 |
| ATOM | 1307 | CG | ASP | A | 92 | 58.053 | 44.679 | 71.108 | 1.00 | 1.03 |
| ATOM | 1308 | OD1 | ASP | A | 92 | 57.761 | 45.762 | 70.536 | 1.00 | 1.03 |
| ATOM | 1309 | OD2 | ASP | A | 92 | 58.629 | 44.660 | 72.217 | 1.00 | 1.03 |
| ATOM | 1310 | C | ASP | A | 92 | 55.896 | 44.077 | 68.939 | 1.00 | 1.03 |
| ATOM | 1311 | O | ASP | A | 92 | 54.959 | 43.546 | 69.531 | 1.00 | 1.03 |
| ATOM | 1312 | N | GLY | A | 93 | 55.747 | 45.201 | 68.237 | 1.00 | 1.09 |
| ATOM | 1313 | H | GLY | A | 93 | 56.539 | 45.515 | 67.693 | 1.00 | 1.09 |
| ATOM | 1314 | CA | GLY | A | 93 | 54.540 | 46.013 | 68.162 | 1.00 | 1.09 |
| ATOM | 1315 | HA1 | GLY | A | 93 | 53.667 | 45.373 | 68.293 | 1.00 | 1.09 |
| ATOM | 1316 | HA2 | GLY | A | 93 | 54.486 | 46.472 | 67.175 | 1.00 | 1.09 |

| | | | | | | | | | | |
|---|---|---|---|---|---|---|---|---|---|---|
| ATOM | 1317 | C | GLY | A | 93 | 54.465 | 47.125 | 69.209 | 1.00 | 1.09 |
| ATOM | 1318 | O | GLY | A | 93 | 53.653 | 48.037 | 69.065 | 1.00 | 1.09 |
| ATOM | 1319 | N | GLU | A | 94 | 55.319 | 47.093 | 70.236 | 1.00 | 1.04 |
| ATOM | 1320 | H | GLU | A | 94 | 55.997 | 46.333 | 70.283 | 1.00 | 1.04 |
| ATOM | 1321 | CA | GLU | A | 94 | 55.273 | 48.020 | 71.369 | 1.00 | 1.04 |
| ATOM | 1322 | HA | GLU | A | 94 | 54.275 | 48.459 | 71.416 | 1.00 | 1.04 |
| ATOM | 1323 | CB | GLU | A | 94 | 55.488 | 47.285 | 72.705 | 1.00 | 1.04 |
| ATOM | 1324 | HB1 | GLU | A | 94 | 55.553 | 48.038 | 73.492 | 1.00 | 1.04 |
| ATOM | 1325 | HB2 | GLU | A | 94 | 56.430 | 46.737 | 72.684 | 1.00 | 1.04 |
| ATOM | 1326 | CG | GLU | A | 94 | 54.333 | 46.330 | 73.049 | 1.00 | 1.04 |
| ATOM | 1327 | HG1 | GLU | A | 94 | 54.333 | 45.495 | 72.344 | 1.00 | 1.04 |
| ATOM | 1328 | HG2 | GLU | A | 94 | 53.386 | 46.864 | 72.939 | 1.00 | 1.04 |
| ATOM | 1329 | CD | GLU | A | 94 | 54.447 | 45.808 | 74.489 | 1.00 | 1.04 |
| ATOM | 1330 | OE1 | GLU | A | 94 | 54.766 | 44.614 | 74.671 | 1.00 | 1.04 |
| ATOM | 1331 | OE2 | GLU | A | 94 | 54.262 | 46.621 | 75.425 | 1.00 | 1.04 |
| ATOM | 1332 | C | GLU | A | 94 | 56.234 | 49.199 | 71.246 | 1.00 | 1.04 |
| ATOM | 1333 | O | GLU | A | 94 | 55.997 | 50.265 | 71.820 | 1.00 | 1.04 |
| ATOM | 1334 | N | GLY | A | 95 | 57.294 | 49.066 | 70.450 | 1.00 | 0.94 |
| ATOM | 1335 | H | GLY | A | 95 | 57.436 | 48.161 | 70.013 | 1.00 | 0.94 |
| ATOM | 1336 | CA | GLY | A | 95 | 58.229 | 50.164 | 70.189 | 1.00 | 0.94 |
| ATOM | 1337 | HA1 | GLY | A | 95 | 57.681 | 51.007 | 69.767 | 1.00 | 0.94 |
| ATOM | 1338 | HA2 | GLY | A | 95 | 58.968 | 49.844 | 69.454 | 1.00 | 0.94 |
| ATOM | 1339 | C | GLY | A | 95 | 58.962 | 50.619 | 71.455 | 1.00 | 0.94 |
| ATOM | 1340 | O | GLY | A | 95 | 59.509 | 49.800 | 72.185 | 1.00 | 0.94 |
| ATOM | 1341 | N | SER | A | 96 | 58.990 | 51.925 | 71.729 | 1.00 | 0.94 |
| ATOM | 1342 | H | SER | A | 96 | 58.443 | 52.553 | 71.147 | 1.00 | 0.94 |
| ATOM | 1343 | CA | SER | A | 96 | 59.519 | 52.438 | 73.005 | 1.00 | 0.94 |
| ATOM | 1344 | HA | SER | A | 96 | 60.386 | 51.849 | 73.309 | 1.00 | 0.94 |
| ATOM | 1345 | CB | SER | A | 96 | 59.958 | 53.904 | 72.860 | 1.00 | 0.94 |
| ATOM | 1346 | HB1 | SER | A | 96 | 60.280 | 54.281 | 73.832 | 1.00 | 0.94 |
| ATOM | 1347 | HB2 | SER | A | 96 | 59.114 | 54.505 | 72.519 | 1.00 | 0.94 |
| ATOM | 1348 | OG | SER | A | 96 | 61.037 | 54.038 | 71.951 | 1.00 | 0.94 |
| ATOM | 1349 | HG | SER | A | 96 | 60.740 | 53.688 | 71.102 | 1.00 | 0.94 |
| ATOM | 1350 | C | SER | A | 96 | 58.510 | 52.384 | 74.157 | 1.00 | 0.94 |
| ATOM | 1351 | O | SER | A | 96 | 58.889 | 52.692 | 75.284 | 1.00 | 0.94 |
| ATOM | 1352 | N | ASN | A | 97 | 57.242 | 52.051 | 73.897 | 1.00 | 1.12 |
| ATOM | 1353 | H | ASN | A | 97 | 56.984 | 51.743 | 72.968 | 1.00 | 1.12 |
| ATOM | 1354 | CA | ASN | A | 97 | 56.176 | 52.168 | 74.897 | 1.00 | 1.12 |
| ATOM | 1355 | HA | ASN | A | 97 | 56.344 | 53.082 | 75.471 | 1.00 | 1.12 |
| ATOM | 1356 | CB | ASN | A | 97 | 54.803 | 52.294 | 74.215 | 1.00 | 1.12 |
| ATOM | 1357 | HB1 | ASN | A | 97 | 54.064 | 52.598 | 74.956 | 1.00 | 1.12 |
| ATOM | 1358 | HB2 | ASN | A | 97 | 54.501 | 51.325 | 73.817 | 1.00 | 1.12 |
| ATOM | 1359 | CG | ASN | A | 97 | 54.815 | 53.317 | 73.107 | 1.00 | 1.12 |
| ATOM | 1360 | OD1 | ASN | A | 97 | 54.755 | 54.517 | 73.307 | 1.00 | 1.12 |

| | | | | | | | | | | |
|---|---|---|---|---|---|---|---|---|---|---|
| ATOM | 1361 | ND2 | ASN | A | 97 | 54.975 | 52.855 | 71.897 | 1.00 | 1.12 |
| ATOM | 1362 | 1HD2 | ASN | A | 97 | 55.133 | 51.857 | 71.784 | 1.00 | 1.12 |
| ATOM | 1363 | 2HD2 | ASN | A | 97 | 55.377 | 53.505 | 71.237 | 1.00 | 1.12 |
| ATOM | 1364 | C | ASN | A | 97 | 56.145 | 51.017 | 75.910 | 1.00 | 1.12 |
| ATOM | 1365 | O | ASN | A | 97 | 55.500 | 51.136 | 76.949 | 1.00 | 1.12 |
| ATOM | 1366 | N | GLY | A | 98 | 56.816 | 49.913 | 75.588 | 1.00 | 1.27 |
| ATOM | 1367 | H | GLY | A | 98 | 57.344 | 49.899 | 74.730 | 1.00 | 1.27 |
| ATOM | 1368 | CA | GLY | A | 98 | 56.731 | 48.659 | 76.315 | 1.00 | 1.27 |
| ATOM | 1369 | HA1 | GLY | A | 98 | 55.686 | 48.360 | 76.390 | 1.00 | 1.27 |
| ATOM | 1370 | HA2 | GLY | A | 98 | 57.116 | 48.793 | 77.325 | 1.00 | 1.27 |
| ATOM | 1371 | C | GLY | A | 98 | 57.521 | 47.541 | 75.644 | 1.00 | 1.27 |
| ATOM | 1372 | O | GLY | A | 98 | 58.438 | 47.803 | 74.857 | 1.00 | 1.27 |
| ATOM | 1373 | N | GLY | A | 99 | 57.149 | 46.300 | 75.941 | 1.00 | 1.15 |
| ATOM | 1374 | H | GLY | A | 99 | 56.250 | 46.173 | 76.394 | 1.00 | 1.15 |
| ATOM | 1375 | CA | GLY | A | 99 | 57.782 | 45.112 | 75.387 | 1.00 | 1.15 |
| ATOM | 1376 | HA1 | GLY | A | 99 | 57.608 | 45.104 | 74.314 | 1.00 | 1.15 |
| ATOM | 1377 | HA2 | GLY | A | 99 | 57.301 | 44.225 | 75.797 | 1.00 | 1.15 |
| ATOM | 1378 | C | GLY | A | 99 | 59.279 | 45.030 | 75.678 | 1.00 | 1.15 |
| ATOM | 1379 | O | GLY | A | 99 | 59.745 | 45.368 | 76.766 | 1.00 | 1.15 |
| ATOM | 1380 | N | VAL | A | 100 | 60.037 | 44.560 | 74.694 | 1.00 | 1.02 |
| ATOM | 1381 | H | VAL | A | 100 | 59.574 | 44.350 | 73.810 | 1.00 | 1.02 |
| ATOM | 1382 | CA | VAL | A | 100 | 61.501 | 44.467 | 74.743 | 1.00 | 1.02 |
| ATOM | 1383 | HA | VAL | A | 100 | 61.793 | 43.850 | 75.594 | 1.00 | 1.02 |
| ATOM | 1384 | CB | VAL | A | 100 | 61.977 | 43.790 | 73.436 | 1.00 | 1.02 |
| ATOM | 1385 | HB | VAL | A | 100 | 61.733 | 44.438 | 72.594 | 1.00 | 1.02 |
| ATOM | 1386 | CG1 | VAL | A | 100 | 63.481 | 43.543 | 73.419 | 1.00 | 1.02 |
| ATOM | 1387 | 1HG1 | VAL | A | 100 | 63.761 | 43.047 | 72.489 | 1.00 | 1.02 |
| ATOM | 1388 | 2HG1 | VAL | A | 100 | 64.027 | 44.479 | 73.496 | 1.00 | 1.02 |
| ATOM | 1389 | 3HG1 | VAL | A | 100 | 63.743 | 42.889 | 74.249 | 1.00 | 1.02 |
| ATOM | 1390 | CG2 | VAL | A | 100 | 61.316 | 42.422 | 73.196 | 1.00 | 1.02 |
| ATOM | 1391 | 1HG2 | VAL | A | 100 | 61.719 | 41.968 | 72.290 | 1.00 | 1.02 |
| ATOM | 1392 | 2HG2 | VAL | A | 100 | 61.510 | 41.761 | 74.042 | 1.00 | 1.02 |
| ATOM | 1393 | 3HG2 | VAL | A | 100 | 60.241 | 42.533 | 73.058 | 1.00 | 1.02 |
| ATOM | 1394 | C | VAL | A | 100 | 62.128 | 45.857 | 74.893 | 1.00 | 1.02 |
| ATOM | 1395 | O | VAL | A | 100 | 61.639 | 46.817 | 74.302 | 1.00 | 1.02 |
| ATOM | 1396 | N | THR | A | 101 | 63.248 | 46.016 | 75.599 | 1.00 | 0.98 |
| ATOM | 1397 | H | THR | A | 101 | 63.620 | 45.234 | 76.122 | 1.00 | 0.98 |
| ATOM | 1398 | CA | THR | A | 101 | 64.004 | 47.287 | 75.598 | 1.00 | 0.98 |
| ATOM | 1399 | HA | THR | A | 101 | 63.310 | 48.123 | 75.686 | 1.00 | 0.98 |
| ATOM | 1400 | CB | THR | A | 101 | 65.005 | 47.338 | 76.765 | 1.00 | 0.98 |
| ATOM | 1401 | HB | THR | A | 101 | 65.877 | 46.746 | 76.513 | 1.00 | 0.98 |
| ATOM | 1402 | CG2 | THR | A | 101 | 65.448 | 48.767 | 77.076 | 1.00 | 0.98 |
| ATOM | 1403 | 1HG2 | THR | A | 101 | 66.198 | 48.750 | 77.867 | 1.00 | 0.98 |
| ATOM | 1404 | 2HG2 | THR | A | 101 | 65.887 | 49.224 | 76.190 | 1.00 | 0.98 |

| | | | | | | | | | | |
|---|---|---|---|---|---|---|---|---|---|---|
| ATOM | 1405 | 3HG2 | THR | A | 101 | 64.593 | 49.357 | 77.405 | 1.00 | 0.98 |
| ATOM | 1406 | OG1 | THR | A | 101 | 64.459 | 46.802 | 77.943 | 1.00 | 0.98 |
| ATOM | 1407 | HG1 | THR | A | 101 | 65.148 | 46.250 | 78.329 | 1.00 | 0.98 |
| ATOM | 1408 | C | THR | A | 101 | 64.821 | 47.406 | 74.312 | 1.00 | 0.98 |
| ATOM | 1409 | O | THR | A | 101 | 65.635 | 46.532 | 74.012 | 1.00 | 0.98 |
| ATOM | 1410 | N | LYS | A | 102 | 64.618 | 48.475 | 73.540 | 1.00 | 0.78 |
| ATOM | 1411 | H | LYS | A | 102 | 64.002 | 49.199 | 73.876 | 1.00 | 0.78 |
| ATOM | 1412 | CA | LYS | A | 102 | 65.082 | 48.563 | 72.145 | 1.00 | 0.78 |
| ATOM | 1413 | HA | LYS | A | 102 | 65.616 | 47.646 | 71.899 | 1.00 | 0.78 |
| ATOM | 1414 | CB | LYS | A | 102 | 63.880 | 48.675 | 71.183 | 1.00 | 0.78 |
| ATOM | 1415 | HB1 | LYS | A | 102 | 64.209 | 48.402 | 70.181 | 1.00 | 0.78 |
| ATOM | 1416 | HB2 | LYS | A | 102 | 63.525 | 49.705 | 71.170 | 1.00 | 0.78 |
| ATOM | 1417 | CG | LYS | A | 102 | 62.702 | 47.784 | 71.590 | 1.00 | 0.78 |
| ATOM | 1418 | HG1 | LYS | A | 102 | 62.282 | 48.215 | 72.494 | 1.00 | 0.78 |
| ATOM | 1419 | HG2 | LYS | A | 102 | 63.061 | 46.771 | 71.783 | 1.00 | 0.78 |
| ATOM | 1420 | CD | LYS | A | 102 | 61.573 | 47.732 | 70.566 | 1.00 | 0.78 |
| ATOM | 1421 | HD1 | LYS | A | 102 | 61.972 | 47.404 | 69.605 | 1.00 | 0.78 |
| ATOM | 1422 | HD2 | LYS | A | 102 | 61.129 | 48.717 | 70.446 | 1.00 | 0.78 |
| ATOM | 1423 | CE | LYS | A | 102 | 60.494 | 46.739 | 71.001 | 1.00 | 0.78 |
| ATOM | 1424 | HE1 | LYS | A | 102 | 60.940 | 45.747 | 71.110 | 1.00 | 0.78 |
| ATOM | 1425 | HE2 | LYS | A | 102 | 59.760 | 46.662 | 70.197 | 1.00 | 0.78 |
| ATOM | 1426 | NZ | LYS | A | 102 | 59.790 | 47.074 | 72.263 | 1.00 | 0.78 |
| ATOM | 1427 | HZ1 | LYS | A | 102 | 59.044 | 46.386 | 72.394 | 1.00 | 0.78 |
| ATOM | 1428 | HZ2 | LYS | A | 102 | 60.388 | 46.988 | 73.080 | 1.00 | 0.78 |
| ATOM | 1429 | HZ3 | LYS | A | 102 | 59.381 | 47.998 | 72.260 | 1.00 | 0.78 |
| ATOM | 1430 | C | LYS | A | 102 | 66.023 | 49.756 | 71.978 | 1.00 | 0.78 |
| ATOM | 1431 | O | LYS | A | 102 | 65.639 | 50.858 | 72.390 | 1.00 | 0.78 |
| ATOM | 1432 | N | PRO | A | 103 | 67.208 | 49.573 | 71.371 | 1.00 | 0.78 |
| ATOM | 1433 | CD | PRO | A | 103 | 67.782 | 48.326 | 70.892 | 1.00 | 0.78 |
| ATOM | 1434 | HD1 | PRO | A | 103 | 67.140 | 47.862 | 70.142 | 1.00 | 0.78 |
| ATOM | 1435 | HD2 | PRO | A | 103 | 67.930 | 47.644 | 71.731 | 1.00 | 0.78 |
| ATOM | 1436 | CG | PRO | A | 103 | 69.136 | 48.683 | 70.279 | 1.00 | 0.78 |
| ATOM | 1437 | HG1 | PRO | A | 103 | 69.049 | 48.765 | 69.200 | 1.00 | 0.78 |
| ATOM | 1438 | HG2 | PRO | A | 103 | 69.902 | 47.957 | 70.544 | 1.00 | 0.78 |
| ATOM | 1439 | CB | PRO | A | 103 | 69.493 | 50.042 | 70.847 | 1.00 | 0.78 |
| ATOM | 1440 | HB1 | PRO | A | 103 | 70.000 | 50.650 | 70.097 | 1.00 | 0.78 |
| ATOM | 1441 | HB2 | PRO | A | 103 | 70.129 | 49.910 | 71.724 | 1.00 | 0.78 |
| ATOM | 1442 | CA | PRO | A | 103 | 68.165 | 50.654 | 71.288 | 1.00 | 0.78 |
| ATOM | 1443 | HA | PRO | A | 103 | 68.299 | 51.067 | 72.281 | 1.00 | 0.78 |
| ATOM | 1444 | C | PRO | A | 103 | 67.681 | 51.776 | 70.378 | 1.00 | 0.78 |
| ATOM | 1445 | O | PRO | A | 103 | 66.968 | 51.532 | 69.402 | 1.00 | 0.78 |
| ATOM | 1446 | N | LYS | A | 104 | 68.075 | 53.006 | 70.707 | 1.00 | 0.55 |
| ATOM | 1447 | H | LYS | A | 104 | 68.694 | 53.082 | 71.511 | 1.00 | 0.55 |
| ATOM | 1448 | CA | LYS | A | 104 | 67.925 | 54.185 | 69.847 | 1.00 | 0.55 |

| | | | | | | | | | | |
|---|---|---|---|---|---|---|---|---|---|---|
| ATOM | 1449 | HA | LYS | A | 104 | 67.042 | 54.056 | 69.216 | 1.00 | 0.55 |
| ATOM | 1450 | CB | LYS | A | 104 | 67.753 | 55.442 | 70.715 | 1.00 | 0.55 |
| ATOM | 1451 | HB1 | LYS | A | 104 | 67.661 | 56.313 | 70.064 | 1.00 | 0.55 |
| ATOM | 1452 | HB2 | LYS | A | 104 | 68.626 | 55.567 | 71.359 | 1.00 | 0.55 |
| ATOM | 1453 | CG | LYS | A | 104 | 66.486 | 55.308 | 71.578 | 1.00 | 0.55 |
| ATOM | 1454 | HG1 | LYS | A | 104 | 66.617 | 54.488 | 72.285 | 1.00 | 0.55 |
| ATOM | 1455 | HG2 | LYS | A | 104 | 65.646 | 55.069 | 70.924 | 1.00 | 0.55 |
| ATOM | 1456 | CD | LYS | A | 104 | 66.155 | 56.576 | 72.375 | 1.00 | 0.55 |
| ATOM | 1457 | HD1 | LYS | A | 104 | 65.902 | 57.390 | 71.692 | 1.00 | 0.55 |
| ATOM | 1458 | HD2 | LYS | A | 104 | 67.034 | 56.868 | 72.954 | 1.00 | 0.55 |
| ATOM | 1459 | CE | LYS | A | 104 | 64.992 | 56.321 | 73.346 | 1.00 | 0.55 |
| ATOM | 1460 | HE1 | LYS | A | 104 | 64.815 | 57.231 | 73.927 | 1.00 | 0.55 |
| ATOM | 1461 | HE2 | LYS | A | 104 | 65.293 | 55.532 | 74.044 | 1.00 | 0.55 |
| ATOM | 1462 | NZ | LYS | A | 104 | 63.749 | 55.922 | 72.636 | 1.00 | 0.55 |
| ATOM | 1463 | HZ1 | LYS | A | 104 | 62.997 | 55.691 | 73.269 | 1.00 | 0.55 |
| ATOM | 1464 | HZ2 | LYS | A | 104 | 63.415 | 56.673 | 72.029 | 1.00 | 0.55 |
| ATOM | 1465 | HZ3 | LYS | A | 104 | 63.911 | 55.121 | 72.045 | 1.00 | 0.55 |
| ATOM | 1466 | C | LYS | A | 104 | 69.139 | 54.257 | 68.912 | 1.00 | 0.55 |
| ATOM | 1467 | O | LYS | A | 104 | 70.263 | 54.067 | 69.342 | 1.00 | 0.55 |
| ATOM | 1468 | N | PHE | A | 105 | 68.959 | 54.419 | 67.606 | 1.00 | 0.43 |
| ATOM | 1469 | H | PHE | A | 105 | 68.025 | 54.593 | 67.249 | 1.00 | 0.43 |
| ATOM | 1470 | CA | PHE | A | 105 | 70.024 | 54.043 | 66.679 | 1.00 | 0.43 |
| ATOM | 1471 | HA | PHE | A | 105 | 70.422 | 53.100 | 67.052 | 1.00 | 0.43 |
| ATOM | 1472 | CB | PHE | A | 105 | 69.463 | 53.716 | 65.288 | 1.00 | 0.43 |
| ATOM | 1473 | HB1 | PHE | A | 105 | 68.977 | 54.599 | 64.893 | 1.00 | 0.43 |
| ATOM | 1474 | HB2 | PHE | A | 105 | 68.717 | 52.924 | 65.376 | 1.00 | 0.43 |
| ATOM | 1475 | CG | PHE | A | 105 | 70.548 | 53.270 | 64.324 | 1.00 | 0.43 |
| ATOM | 1476 | CD1 | PHE | A | 105 | 71.331 | 52.139 | 64.622 | 1.00 | 0.43 |
| ATOM | 1477 | HD1 | PHE | A | 105 | 71.095 | 51.529 | 65.479 | 1.00 | 0.43 |
| ATOM | 1478 | CE1 | PHE | A | 105 | 72.459 | 51.833 | 63.842 | 1.00 | 0.43 |
| ATOM | 1479 | HE1 | PHE | A | 105 | 73.083 | 50.988 | 64.097 | 1.00 | 0.43 |
| ATOM | 1480 | CZ | PHE | A | 105 | 72.779 | 52.626 | 62.730 | 1.00 | 0.43 |
| ATOM | 1481 | HZ | PHE | A | 105 | 73.652 | 52.389 | 62.141 | 1.00 | 0.43 |
| ATOM | 1482 | CE2 | PHE | A | 105 | 71.971 | 53.726 | 62.398 | 1.00 | 0.43 |
| ATOM | 1483 | HE2 | PHE | A | 105 | 72.219 | 54.343 | 61.548 | 1.00 | 0.43 |
| ATOM | 1484 | CD2 | PHE | A | 105 | 70.862 | 54.052 | 63.199 | 1.00 | 0.43 |
| ATOM | 1485 | HD2 | PHE | A | 105 | 70.274 | 54.931 | 62.979 | 1.00 | 0.43 |
| ATOM | 1486 | C | PHE | A | 105 | 71.228 | 54.969 | 66.606 | 1.00 | 0.43 |
| ATOM | 1487 | O | PHE | A | 105 | 72.343 | 54.506 | 66.840 | 1.00 | 0.43 |
| ATOM | 1488 | N | PHE | A | 106 | 71.019 | 56.246 | 66.299 | 1.00 | 0.47 |
| ATOM | 1489 | H | PHE | A | 106 | 70.080 | 56.550 | 66.070 | 1.00 | 0.47 |
| ATOM | 1490 | CA | PHE | A | 106 | 72.148 | 57.150 | 65.958 | 1.00 | 0.47 |
| ATOM | 1491 | HA | PHE | A | 106 | 73.108 | 56.667 | 66.148 | 1.00 | 0.47 |
| ATOM | 1492 | CB | PHE | A | 106 | 72.042 | 57.471 | 64.454 | 1.00 | 0.47 |

| | | | | | | | | | | |
|---|---|---|---|---|---|---|---|---|---|---|
| ATOM | 1493 | HB1 | PHE | A | 106 | 71.253 | 58.201 | 64.299 | 1.00 | 0.47 |
| ATOM | 1494 | HB2 | PHE | A | 106 | 71.726 | 56.564 | 63.941 | 1.00 | 0.47 |
| ATOM | 1495 | CG | PHE | A | 106 | 73.261 | 57.992 | 63.725 | 1.00 | 0.47 |
| ATOM | 1496 | CD1 | PHE | A | 106 | 73.729 | 59.303 | 63.940 | 1.00 | 0.47 |
| ATOM | 1497 | HD1 | PHE | A | 106 | 73.269 | 59.932 | 64.690 | 1.00 | 0.47 |
| ATOM | 1498 | CE1 | PHE | A | 106 | 74.778 | 59.811 | 63.152 | 1.00 | 0.47 |
| ATOM | 1499 | HE1 | PHE | A | 106 | 75.125 | 60.821 | 63.310 | 1.00 | 0.47 |
| ATOM | 1500 | CZ | PHE | A | 106 | 75.355 | 59.014 | 62.147 | 1.00 | 0.47 |
| ATOM | 1501 | HZ | PHE | A | 106 | 76.151 | 59.404 | 61.534 | 1.00 | 0.47 |
| ATOM | 1502 | CE2 | PHE | A | 106 | 74.887 | 57.709 | 61.928 | 1.00 | 0.47 |
| ATOM | 1503 | HE2 | PHE | A | 106 | 75.331 | 57.094 | 61.157 | 1.00 | 0.47 |
| ATOM | 1504 | CD2 | PHE | A | 106 | 73.838 | 57.204 | 62.713 | 1.00 | 0.47 |
| ATOM | 1505 | HD2 | PHE | A | 106 | 73.471 | 56.206 | 62.528 | 1.00 | 0.47 |
| ATOM | 1506 | C | PHE | A | 106 | 72.031 | 58.383 | 66.836 | 1.00 | 0.47 |
| ATOM | 1507 | O | PHE | A | 106 | 71.085 | 59.148 | 66.666 | 1.00 | 0.47 |
| ATOM | 1508 | N | TYR | A | 107 | 72.935 | 58.601 | 67.794 | 1.00 | 0.49 |
| ATOM | 1509 | H | TYR | A | 107 | 73.715 | 57.960 | 67.927 | 1.00 | 0.49 |
| ATOM | 1510 | CA | TYR | A | 107 | 72.959 | 59.906 | 68.447 | 1.00 | 0.49 |
| ATOM | 1511 | HA | TYR | A | 107 | 71.961 | 60.336 | 68.544 | 1.00 | 0.49 |
| ATOM | 1512 | CB | TYR | A | 107 | 73.566 | 59.769 | 69.849 | 1.00 | 0.49 |
| ATOM | 1513 | HB1 | TYR | A | 107 | 73.911 | 60.753 | 70.162 | 1.00 | 0.49 |
| ATOM | 1514 | HB2 | TYR | A | 107 | 74.443 | 59.126 | 69.802 | 1.00 | 0.49 |
| ATOM | 1515 | CG | TYR | A | 107 | 72.638 | 59.228 | 70.932 | 1.00 | 0.49 |
| ATOM | 1516 | CD1 | TYR | A | 107 | 71.328 | 58.770 | 70.650 | 1.00 | 0.49 |
| ATOM | 1517 | HD1 | TYR | A | 107 | 70.943 | 58.760 | 69.641 | 1.00 | 0.49 |
| ATOM | 1518 | CE1 | TYR | A | 107 | 70.502 | 58.302 | 71.686 | 1.00 | 0.49 |
| ATOM | 1519 | HE1 | TYR | A | 107 | 69.506 | 57.944 | 71.476 | 1.00 | 0.49 |
| ATOM | 1520 | CZ | TYR | A | 107 | 70.970 | 58.292 | 73.013 | 1.00 | 0.49 |
| ATOM | 1521 | OH | TYR | A | 107 | 70.156 | 57.845 | 74.001 | 1.00 | 0.49 |
| ATOM | 1522 | HH | TYR | A | 107 | 70.593 | 57.814 | 74.854 | 1.00 | 0.49 |
| ATOM | 1523 | CE2 | TYR | A | 107 | 72.267 | 58.762 | 73.306 | 1.00 | 0.49 |
| ATOM | 1524 | HE2 | TYR | A | 107 | 72.627 | 58.784 | 74.323 | 1.00 | 0.49 |
| ATOM | 1525 | CD2 | TYR | A | 107 | 73.101 | 59.213 | 72.265 | 1.00 | 0.49 |
| ATOM | 1526 | HD2 | TYR | A | 107 | 74.093 | 59.587 | 72.496 | 1.00 | 0.49 |
| ATOM | 1527 | C | TYR | A | 107 | 73.758 | 60.800 | 67.517 | 1.00 | 0.49 |
| ATOM | 1528 | O | TYR | A | 107 | 74.779 | 60.365 | 67.001 | 1.00 | 0.49 |
| ATOM | 1529 | N | ALA | A | 108 | 73.291 | 62.011 | 67.259 | 1.00 | 0.46 |
| ATOM | 1530 | H | ALA | A | 108 | 72.355 | 62.262 | 67.556 | 1.00 | 0.46 |
| ATOM | 1531 | CA | ALA | A | 108 | 74.116 | 63.059 | 66.683 | 1.00 | 0.46 |
| ATOM | 1532 | HA | ALA | A | 108 | 75.158 | 62.741 | 66.609 | 1.00 | 0.46 |
| ATOM | 1533 | CB | ALA | A | 108 | 73.599 | 63.395 | 65.277 | 1.00 | 0.46 |
| ATOM | 1534 | HB1 | ALA | A | 108 | 74.160 | 64.236 | 64.873 | 1.00 | 0.46 |
| ATOM | 1535 | HB2 | ALA | A | 108 | 73.718 | 62.531 | 64.624 | 1.00 | 0.46 |
| ATOM | 1536 | HB3 | ALA | A | 108 | 72.542 | 63.664 | 65.322 | 1.00 | 0.46 |

| | | | | | | | | | | |
|---|---|---|---|---|---|---|---|---|---|---|
| ATOM | 1537 | C | ALA | A | 108 | 74.033 | 64.216 | 67.683 | 1.00 | 0.46 |
| ATOM | 1538 | O | ALA | A | 108 | 73.018 | 64.927 | 67.744 | 1.00 | 0.46 |
| ATOM | 1539 | N | HIS | A | 109 | 75.037 | 64.287 | 68.567 | 1.00 | 0.48 |
| ATOM | 1540 | H | HIS | A | 109 | 75.883 | 63.740 | 68.435 | 1.00 | 0.48 |
| ATOM | 1541 | CA | HIS | A | 109 | 74.874 | 65.005 | 69.842 | 1.00 | 0.48 |
| ATOM | 1542 | HA | HIS | A | 109 | 74.021 | 65.658 | 69.798 | 1.00 | 0.48 |
| ATOM | 1543 | CB | HIS | A | 109 | 74.654 | 64.016 | 71.013 | 1.00 | 0.48 |
| ATOM | 1544 | HB1 | HIS | A | 109 | 74.803 | 64.569 | 71.942 | 1.00 | 0.48 |
| ATOM | 1545 | HB2 | HIS | A | 109 | 75.431 | 63.251 | 70.976 | 1.00 | 0.48 |
| ATOM | 1546 | CG | HIS | A | 109 | 73.314 | 63.315 | 71.159 | 1.00 | 0.48 |
| ATOM | 1547 | ND1 | HIS | A | 109 | 73.036 | 62.364 | 72.144 | 1.00 | 0.48 |
| ATOM | 1548 | CE1 | HIS | A | 109 | 71.734 | 62.059 | 72.026 | 1.00 | 0.48 |
| ATOM | 1549 | HE1 | HIS | A | 109 | 71.207 | 61.358 | 72.659 | 1.00 | 0.48 |
| ATOM | 1550 | NE2 | HIS | A | 109 | 71.181 | 62.738 | 71.010 | 1.00 | 0.48 |
| ATOM | 1551 | HE2 | HIS | A | 109 | 70.191 | 62.740 | 70.770 | 1.00 | 0.48 |
| ATOM | 1552 | CD2 | HIS | A | 109 | 72.160 | 63.543 | 70.466 | 1.00 | 0.48 |
| ATOM | 1553 | HD2 | HIS | A | 109 | 72.015 | 64.288 | 69.710 | 1.00 | 0.48 |
| ATOM | 1554 | C | HIS | A | 109 | 76.111 | 65.848 | 70.143 | 1.00 | 0.48 |
| ATOM | 1555 | O | HIS | A | 109 | 77.199 | 65.284 | 70.134 | 1.00 | 0.48 |
| ATOM | 1556 | N | ASP | A | 110 | 75.988 | 67.169 | 70.353 | 1.00 | 0.91 |
| ATOM | 1557 | H | ASP | A | 110 | 75.074 | 67.605 | 70.409 | 1.00 | 0.91 |
| ATOM | 1558 | CA | ASP | A | 110 | 77.183 | 68.041 | 70.263 | 1.00 | 0.91 |
| ATOM | 1559 | HA | ASP | A | 110 | 76.813 | 69.012 | 69.979 | 1.00 | 0.91 |
| ATOM | 1560 | CB | ASP | A | 110 | 77.885 | 68.343 | 71.604 | 1.00 | 0.91 |
| ATOM | 1561 | HB1 | ASP | A | 110 | 77.118 | 68.621 | 72.330 | 1.00 | 0.91 |
| ATOM | 1562 | HB2 | ASP | A | 110 | 78.507 | 69.228 | 71.457 | 1.00 | 0.91 |
| ATOM | 1563 | CG | ASP | A | 110 | 78.769 | 67.253 | 72.211 | 1.00 | 0.91 |
| ATOM | 1564 | OD1 | ASP | A | 110 | 78.373 | 66.682 | 73.256 | 1.00 | 0.91 |
| ATOM | 1565 | OD2 | ASP | A | 110 | 79.914 | 67.031 | 71.756 | 1.00 | 0.91 |
| ATOM | 1566 | C | ASP | A | 110 | 78.075 | 67.774 | 69.025 | 1.00 | 0.91 |
| ATOM | 1567 | O | ASP | A | 110 | 79.305 | 67.751 | 69.043 | 1.00 | 0.91 |
| ATOM | 1568 | N | LEU | A | 111 | 77.419 | 67.630 | 67.870 | 1.00 | 1.12 |
| ATOM | 1569 | H | LEU | A | 111 | 76.411 | 67.623 | 67.904 | 1.00 | 1.12 |
| ATOM | 1570 | CA | LEU | A | 111 | 78.039 | 68.047 | 66.618 | 1.00 | 1.12 |
| ATOM | 1571 | HA | LEU | A | 111 | 79.098 | 67.804 | 66.652 | 1.00 | 1.12 |
| ATOM | 1572 | CB | LEU | A | 111 | 77.417 | 67.305 | 65.419 | 1.00 | 1.12 |
| ATOM | 1573 | HB1 | LEU | A | 111 | 77.692 | 67.848 | 64.518 | 1.00 | 1.12 |
| ATOM | 1574 | HB2 | LEU | A | 111 | 76.333 | 67.349 | 65.509 | 1.00 | 1.12 |
| ATOM | 1575 | CG | LEU | A | 111 | 77.844 | 65.838 | 65.223 | 1.00 | 1.12 |
| ATOM | 1576 | HG | LEU | A | 111 | 77.530 | 65.248 | 66.084 | 1.00 | 1.12 |
| ATOM | 1577 | CD1 | LEU | A | 111 | 77.153 | 65.288 | 63.969 | 1.00 | 1.12 |
| ATOM | 1578 | 1HD1 | LEU | A | 111 | 77.414 | 64.239 | 63.840 | 1.00 | 1.12 |
| ATOM | 1579 | 2HD1 | LEU | A | 111 | 76.073 | 65.377 | 64.080 | 1.00 | 1.12 |
| ATOM | 1580 | 3HD1 | LEU | A | 111 | 77.477 | 65.839 | 63.086 | 1.00 | 1.12 |

| | | | | | | | | | | |
|---|---|---|---|---|---|---|---|---|---|---|
| ATOM | 1581 | CD2 | LEU | A | 111 | 79.354 | 65.673 | 65.016 | 1.00 | 1.12 |
| ATOM | 1582 | 1HD2 | LEU | A | 111 | 79.878 | 65.938 | 65.930 | 1.00 | 1.12 |
| ATOM | 1583 | 2HD2 | LEU | A | 111 | 79.595 | 64.639 | 64.781 | 1.00 | 1.12 |
| ATOM | 1584 | 3HD2 | LEU | A | 111 | 79.704 | 66.324 | 64.216 | 1.00 | 1.12 |
| ATOM | 1585 | C | LEU | A | 111 | 77.934 | 69.555 | 66.394 | 1.00 | 1.12 |
| ATOM | 1586 | O | LEU | A | 111 | 76.846 | 70.133 | 66.472 | 1.00 | 1.12 |
| ATOM | 1587 | N | THR | A | 112 | 79.036 | 70.149 | 65.951 | 1.00 | 1.07 |
| ATOM | 1588 | H | THR | A | 112 | 79.895 | 69.609 | 65.908 | 1.00 | 1.07 |
| ATOM | 1589 | CA | THR | A | 112 | 79.063 | 71.553 | 65.512 | 1.00 | 1.07 |
| ATOM | 1590 | HA | THR | A | 112 | 78.051 | 71.938 | 65.456 | 1.00 | 1.07 |
| ATOM | 1591 | CB | THR | A | 112 | 79.867 | 72.438 | 66.483 | 1.00 | 1.07 |
| ATOM | 1592 | HB | THR | A | 112 | 80.922 | 72.377 | 66.222 | 1.00 | 1.07 |
| ATOM | 1593 | CG2 | THR | A | 112 | 79.438 | 73.902 | 66.417 | 1.00 | 1.07 |
| ATOM | 1594 | 1HG2 | THR | A | 112 | 80.045 | 74.491 | 67.105 | 1.00 | 1.07 |
| ATOM | 1595 | 2HG2 | THR | A | 112 | 79.574 | 74.292 | 65.409 | 1.00 | 1.07 |
| ATOM | 1596 | 3HG2 | THR | A | 112 | 78.390 | 73.988 | 66.700 | 1.00 | 1.07 |
| ATOM | 1597 | OG1 | THR | A | 112 | 79.732 | 72.052 | 67.831 | 1.00 | 1.07 |
| ATOM | 1598 | HG1 | THR | A | 112 | 80.574 | 71.630 | 68.072 | 1.00 | 1.07 |
| ATOM | 1599 | C | THR | A | 112 | 79.712 | 71.646 | 64.142 | 1.00 | 1.07 |
| ATOM | 1600 | O | THR | A | 112 | 80.636 | 70.882 | 63.858 | 1.00 | 1.07 |
| ATOM | 1601 | N | ASP | A | 113 | 79.235 | 72.554 | 63.291 | 1.00 | 0.96 |
| ATOM | 1602 | H | ASP | A | 113 | 78.442 | 73.112 | 63.595 | 1.00 | 0.96 |
| ATOM | 1603 | CA | ASP | A | 113 | 79.653 | 72.705 | 61.884 | 1.00 | 0.96 |
| ATOM | 1604 | HA | ASP | A | 113 | 78.787 | 73.086 | 61.346 | 1.00 | 0.96 |
| ATOM | 1605 | CB | ASP | A | 113 | 80.738 | 73.780 | 61.730 | 1.00 | 0.96 |
| ATOM | 1606 | HB1 | ASP | A | 113 | 81.678 | 73.425 | 62.158 | 1.00 | 0.96 |
| ATOM | 1607 | HB2 | ASP | A | 113 | 80.431 | 74.679 | 62.268 | 1.00 | 0.96 |
| ATOM | 1608 | CG | ASP | A | 113 | 80.915 | 74.132 | 60.248 | 1.00 | 0.96 |
| ATOM | 1609 | OD1 | ASP | A | 113 | 79.870 | 74.339 | 59.585 | 1.00 | 0.96 |
| ATOM | 1610 | OD2 | ASP | A | 113 | 82.075 | 74.213 | 59.792 | 1.00 | 0.96 |
| ATOM | 1611 | C | ASP | A | 113 | 80.023 | 71.383 | 61.209 | 1.00 | 0.96 |
| ATOM | 1612 | O | ASP | A | 113 | 81.183 | 71.044 | 61.014 | 1.00 | 0.96 |
| ATOM | 1613 | N | SER | A | 114 | 79.020 | 70.533 | 61.043 | 1.00 | 0.76 |
| ATOM | 1614 | H | SER | A | 114 | 78.079 | 70.886 | 61.197 | 1.00 | 0.76 |
| ATOM | 1615 | CA | SER | A | 114 | 79.230 | 69.127 | 60.690 | 1.00 | 0.76 |
| ATOM | 1616 | HA | SER | A | 114 | 80.217 | 68.998 | 60.244 | 1.00 | 0.76 |
| ATOM | 1617 | CB | SER | A | 114 | 79.168 | 68.269 | 61.953 | 1.00 | 0.76 |
| ATOM | 1618 | HB1 | SER | A | 114 | 79.088 | 67.217 | 61.677 | 1.00 | 0.76 |
| ATOM | 1619 | HB2 | SER | A | 114 | 78.295 | 68.552 | 62.539 | 1.00 | 0.76 |
| ATOM | 1620 | OG | SER | A | 114 | 80.343 | 68.439 | 62.721 | 1.00 | 0.76 |
| ATOM | 1621 | HG | SER | A | 114 | 80.438 | 69.382 | 62.970 | 1.00 | 0.76 |
| ATOM | 1622 | C | SER | A | 114 | 78.206 | 68.689 | 59.647 | 1.00 | 0.76 |
| ATOM | 1623 | O | SER | A | 114 | 77.137 | 69.282 | 59.463 | 1.00 | 0.76 |
| ATOM | 1624 | N | THR | A | 115 | 78.557 | 67.633 | 58.927 | 1.00 | 0.45 |

| | | | | | | | | | | |
|---|---|---|---|---|---|---|---|---|---|---|
| ATOM | 1625 | H | THR | A | 115 | 79.450 | 67.192 | 59.137 | 1.00 | 0.45 |
| ATOM | 1626 | CA | THR | A | 115 | 77.835 | 67.191 | 57.742 | 1.00 | 0.45 |
| ATOM | 1627 | HA | THR | A | 115 | 76.799 | 67.510 | 57.834 | 1.00 | 0.45 |
| ATOM | 1628 | CB | THR | A | 115 | 78.394 | 67.895 | 56.489 | 1.00 | 0.45 |
| ATOM | 1629 | HB | THR | A | 115 | 78.375 | 68.968 | 56.685 | 1.00 | 0.45 |
| ATOM | 1630 | CG2 | THR | A | 115 | 79.826 | 67.546 | 56.093 | 1.00 | 0.45 |
| ATOM | 1631 | 1HG2 | THR | A | 115 | 80.136 | 68.196 | 55.275 | 1.00 | 0.45 |
| ATOM | 1632 | 2HG2 | THR | A | 115 | 80.485 | 67.713 | 56.944 | 1.00 | 0.45 |
| ATOM | 1633 | 3HG2 | THR | A | 115 | 79.884 | 66.513 | 55.762 | 1.00 | 0.45 |
| ATOM | 1634 | OG1 | THR | A | 115 | 77.565 | 67.665 | 55.381 | 1.00 | 0.45 |
| ATOM | 1635 | HG1 | THR | A | 115 | 76.781 | 68.213 | 55.540 | 1.00 | 0.45 |
| ATOM | 1636 | C | THR | A | 115 | 77.816 | 65.665 | 57.655 | 1.00 | 0.45 |
| ATOM | 1637 | O | THR | A | 115 | 78.831 | 65.012 | 57.437 | 1.00 | 0.45 |
| ATOM | 1638 | N | ILE | A | 116 | 76.642 | 65.105 | 57.927 | 1.00 | 0.49 |
| ATOM | 1639 | H | ILE | A | 116 | 75.884 | 65.760 | 58.067 | 1.00 | 0.49 |
| ATOM | 1640 | CA | ILE | A | 116 | 76.178 | 63.761 | 57.628 | 1.00 | 0.49 |
| ATOM | 1641 | HA | ILE | A | 116 | 77.000 | 63.062 | 57.708 | 1.00 | 0.49 |
| ATOM | 1642 | CB | ILE | A | 116 | 75.028 | 63.356 | 58.594 | 1.00 | 0.49 |
| ATOM | 1643 | HB | ILE | A | 116 | 74.117 | 63.829 | 58.243 | 1.00 | 0.49 |
| ATOM | 1644 | CG2 | ILE | A | 116 | 74.775 | 61.840 | 58.541 | 1.00 | 0.49 |
| ATOM | 1645 | 1HG2 | ILE | A | 116 | 74.590 | 61.518 | 57.518 | 1.00 | 0.49 |
| ATOM | 1646 | 2HG2 | ILE | A | 116 | 75.625 | 61.299 | 58.948 | 1.00 | 0.49 |
| ATOM | 1647 | 3HG2 | ILE | A | 116 | 73.882 | 61.591 | 59.112 | 1.00 | 0.49 |
| ATOM | 1648 | CG1 | ILE | A | 116 | 75.258 | 63.864 | 60.042 | 1.00 | 0.49 |
| ATOM | 1649 | 1HG1 | ILE | A | 116 | 75.176 | 64.951 | 60.038 | 1.00 | 0.49 |
| ATOM | 1650 | 2HG1 | ILE | A | 116 | 76.269 | 63.615 | 60.360 | 1.00 | 0.49 |
| ATOM | 1651 | CD1 | ILE | A | 116 | 74.273 | 63.357 | 61.103 | 1.00 | 0.49 |
| ATOM | 1652 | HD1 | ILE | A | 116 | 74.456 | 63.878 | 62.043 | 1.00 | 0.49 |
| ATOM | 1653 | HD2 | ILE | A | 116 | 73.249 | 63.551 | 60.784 | 1.00 | 0.49 |
| ATOM | 1654 | HD3 | ILE | A | 116 | 74.409 | 62.288 | 61.269 | 1.00 | 0.49 |
| ATOM | 1655 | C | ILE | A | 116 | 75.665 | 63.806 | 56.182 | 1.00 | 0.49 |
| ATOM | 1656 | O | ILE | A | 116 | 75.243 | 64.867 | 55.705 | 1.00 | 0.49 |
| ATOM | 1657 | N | GLN | A | 117 | 75.811 | 62.683 | 55.481 | 1.00 | 0.54 |
| ATOM | 1658 | H | GLN | A | 117 | 76.160 | 61.883 | 55.983 | 1.00 | 0.54 |
| ATOM | 1659 | CA | GLN | A | 117 | 75.902 | 62.603 | 54.019 | 1.00 | 0.54 |
| ATOM | 1660 | HA | GLN | A | 117 | 75.132 | 63.228 | 53.583 | 1.00 | 0.54 |
| ATOM | 1661 | CB | GLN | A | 117 | 77.273 | 63.134 | 53.525 | 1.00 | 0.54 |
| ATOM | 1662 | HB1 | GLN | A | 117 | 77.272 | 63.087 | 52.435 | 1.00 | 0.54 |
| ATOM | 1663 | HB2 | GLN | A | 117 | 78.053 | 62.468 | 53.884 | 1.00 | 0.54 |
| ATOM | 1664 | CG | GLN | A | 117 | 77.645 | 64.569 | 53.942 | 1.00 | 0.54 |
| ATOM | 1665 | HG1 | GLN | A | 117 | 77.909 | 64.584 | 54.997 | 1.00 | 0.54 |
| ATOM | 1666 | HG2 | GLN | A | 117 | 76.786 | 65.219 | 53.781 | 1.00 | 0.54 |
| ATOM | 1667 | CD | GLN | A | 117 | 78.814 | 65.152 | 53.165 | 1.00 | 0.54 |
| ATOM | 1668 | OE1 | GLN | A | 117 | 79.979 | 65.070 | 53.535 | 1.00 | 0.54 |

| | | | | | | | | | | |
|---|---|---|---|---|---|---|---|---|---|---|
| ATOM | 1669 | NE2 | GLN | A | 117 | 78.534 | 65.781 | 52.044 | 1.00 | 0.54 |
| ATOM | 1670 | 1HE2 | GLN | A | 117 | 77.577 | 65.869 | 51.751 | 1.00 | 0.54 |
| ATOM | 1671 | 2HE2 | GLN | A | 117 | 79.303 | 66.185 | 51.548 | 1.00 | 0.54 |
| ATOM | 1672 | C | GLN | A | 117 | 75.776 | 61.140 | 53.497 | 1.00 | 0.54 |
| ATOM | 1673 | O | GLN | A | 117 | 76.572 | 60.240 | 53.832 | 1.00 | 0.54 |
| ATOM | 1674 | N | ASN | A | 118 | 74.817 | 60.937 | 52.587 | 1.00 | 0.93 |
| ATOM | 1675 | H | ASN | A | 118 | 74.159 | 61.694 | 52.426 | 1.00 | 0.93 |
| ATOM | 1676 | CA | ASN | A | 118 | 74.589 | 59.674 | 51.870 | 1.00 | 0.93 |
| ATOM | 1677 | HA | ASN | A | 118 | 73.621 | 59.743 | 51.386 | 1.00 | 0.93 |
| ATOM | 1678 | CB | ASN | A | 118 | 75.619 | 59.505 | 50.722 | 1.00 | 0.93 |
| ATOM | 1679 | HB1 | ASN | A | 118 | 75.582 | 58.488 | 50.336 | 1.00 | 0.93 |
| ATOM | 1680 | HB2 | ASN | A | 118 | 76.626 | 59.677 | 51.099 | 1.00 | 0.93 |
| ATOM | 1681 | CG | ASN | A | 118 | 75.374 | 60.395 | 49.523 | 1.00 | 0.93 |
| ATOM | 1682 | OD1 | ASN | A | 118 | 76.218 | 61.178 | 49.122 | 1.00 | 0.93 |
| ATOM | 1683 | ND2 | ASN | A | 118 | 74.227 | 60.317 | 48.886 | 1.00 | 0.93 |
| ATOM | 1684 | 1HD2 | ASN | A | 118 | 73.459 | 59.749 | 49.216 | 1.00 | 0.93 |
| ATOM | 1685 | 2HD2 | ASN | A | 118 | 74.119 | 60.935 | 48.105 | 1.00 | 0.93 |
| ATOM | 1686 | C | ASN | A | 118 | 74.483 | 58.422 | 52.752 | 1.00 | 0.93 |
| ATOM | 1687 | O | ASN | A | 118 | 74.921 | 57.339 | 52.357 | 1.00 | 0.93 |
| ATOM | 1688 | N | LEU | A | 119 | 73.925 | 58.540 | 53.961 | 1.00 | 0.61 |
| ATOM | 1689 | H | LEU | A | 119 | 73.488 | 59.412 | 54.221 | 1.00 | 0.61 |
| ATOM | 1690 | CA | LEU | A | 119 | 73.926 | 57.384 | 54.878 | 1.00 | 0.61 |
| ATOM | 1691 | HA | LEU | A | 119 | 74.755 | 56.724 | 54.628 | 1.00 | 0.61 |
| ATOM | 1692 | CB | LEU | A | 119 | 74.090 | 57.785 | 56.354 | 1.00 | 0.61 |
| ATOM | 1693 | HB1 | LEU | A | 119 | 73.754 | 56.947 | 56.967 | 1.00 | 0.61 |
| ATOM | 1694 | HB2 | LEU | A | 119 | 73.438 | 58.633 | 56.555 | 1.00 | 0.61 |
| ATOM | 1695 | CG | LEU | A | 119 | 75.528 | 58.146 | 56.794 | 1.00 | 0.61 |
| ATOM | 1696 | HG | LEU | A | 119 | 75.833 | 59.070 | 56.303 | 1.00 | 0.61 |
| ATOM | 1697 | CD1 | LEU | A | 119 | 75.546 | 58.358 | 58.309 | 1.00 | 0.61 |
| ATOM | 1698 | 1HD1 | LEU | A | 119 | 76.522 | 58.733 | 58.618 | 1.00 | 0.61 |
| ATOM | 1699 | 2HD1 | LEU | A | 119 | 74.784 | 59.082 | 58.586 | 1.00 | 0.61 |
| ATOM | 1700 | 3HD1 | LEU | A | 119 | 75.342 | 57.416 | 58.816 | 1.00 | 0.61 |
| ATOM | 1701 | CD2 | LEU | A | 119 | 76.569 | 57.056 | 56.508 | 1.00 | 0.61 |
| ATOM | 1702 | 1HD2 | LEU | A | 119 | 77.534 | 57.340 | 56.930 | 1.00 | 0.61 |
| ATOM | 1703 | 2HD2 | LEU | A | 119 | 76.251 | 56.103 | 56.932 | 1.00 | 0.61 |
| ATOM | 1704 | 3HD2 | LEU | A | 119 | 76.706 | 56.947 | 55.435 | 1.00 | 0.61 |
| ATOM | 1705 | C | LEU | A | 119 | 72.637 | 56.586 | 54.607 | 1.00 | 0.61 |
| ATOM | 1706 | O | LEU | A | 119 | 71.509 | 57.090 | 54.722 | 1.00 | 0.61 |
| ATOM | 1707 | N | TYR | A | 120 | 72.850 | 55.323 | 54.226 | 1.00 | 0.41 |
| ATOM | 1708 | H | TYR | A | 120 | 73.800 | 55.047 | 54.037 | 1.00 | 0.41 |
| ATOM | 1709 | CA | TYR | A | 120 | 71.834 | 54.285 | 54.115 | 1.00 | 0.41 |
| ATOM | 1710 | HA | TYR | A | 120 | 70.873 | 54.758 | 53.943 | 1.00 | 0.41 |
| ATOM | 1711 | CB | TYR | A | 120 | 72.104 | 53.359 | 52.901 | 1.00 | 0.41 |
| ATOM | 1712 | HB1 | TYR | A | 120 | 72.857 | 52.619 | 53.167 | 1.00 | 0.41 |

| | | | | | | | | | | |
|---|---|---|---|---|---|---|---|---|---|---|
| ATOM | 1713 | HB2 | TYR | A | 120 | 72.536 | 53.975 | 52.110 | 1.00 | 0.41 |
| ATOM | 1714 | CG | TYR | A | 120 | 70.890 | 52.637 | 52.309 | 1.00 | 0.41 |
| ATOM | 1715 | CD1 | TYR | A | 120 | 70.763 | 52.533 | 50.908 | 1.00 | 0.41 |
| ATOM | 1716 | HD1 | TYR | A | 120 | 71.544 | 52.924 | 50.266 | 1.00 | 0.41 |
| ATOM | 1717 | CE1 | TYR | A | 120 | 69.617 | 51.939 | 50.335 | 1.00 | 0.41 |
| ATOM | 1718 | HE1 | TYR | A | 120 | 69.508 | 51.878 | 49.262 | 1.00 | 0.41 |
| ATOM | 1719 | CZ | TYR | A | 120 | 68.567 | 51.461 | 51.152 | 1.00 | 0.41 |
| ATOM | 1720 | OH | TYR | A | 120 | 67.452 | 50.934 | 50.576 | 1.00 | 0.41 |
| ATOM | 1721 | HH | TYR | A | 120 | 66.709 | 50.753 | 51.200 | 1.00 | 0.41 |
| ATOM | 1722 | CE2 | TYR | A | 120 | 68.720 | 51.509 | 52.552 | 1.00 | 0.41 |
| ATOM | 1723 | HE2 | TYR | A | 120 | 67.931 | 51.130 | 53.188 | 1.00 | 0.41 |
| ATOM | 1724 | CD2 | TYR | A | 120 | 69.881 | 52.075 | 53.121 | 1.00 | 0.41 |
| ATOM | 1725 | HD2 | TYR | A | 120 | 69.967 | 52.094 | 54.194 | 1.00 | 0.41 |
| ATOM | 1726 | C | TYR | A | 120 | 71.738 | 53.514 | 55.425 | 1.00 | 0.41 |
| ATOM | 1727 | O | TYR | A | 120 | 72.535 | 52.619 | 55.717 | 1.00 | 0.41 |
| ATOM | 1728 | N | ILE | A | 121 | 70.736 | 53.883 | 56.211 | 1.00 | 0.29 |
| ATOM | 1729 | H | ILE | A | 121 | 70.189 | 54.690 | 55.920 | 1.00 | 0.29 |
| ATOM | 1730 | CA | ILE | A | 121 | 70.178 | 53.071 | 57.291 | 1.00 | 0.29 |
| ATOM | 1731 | HA | ILE | A | 121 | 70.959 | 52.412 | 57.666 | 1.00 | 0.29 |
| ATOM | 1732 | CB | ILE | A | 121 | 69.713 | 53.987 | 58.453 | 1.00 | 0.29 |
| ATOM | 1733 | HB | ILE | A | 121 | 68.843 | 54.554 | 58.120 | 1.00 | 0.29 |
| ATOM | 1734 | CG2 | ILE | A | 121 | 69.311 | 53.145 | 59.681 | 1.00 | 0.29 |
| ATOM | 1735 | 1HG2 | ILE | A | 121 | 68.871 | 53.778 | 60.447 | 1.00 | 0.29 |
| ATOM | 1736 | 2HG2 | ILE | A | 121 | 68.553 | 52.419 | 59.398 | 1.00 | 0.29 |
| ATOM | 1737 | 3HG2 | ILE | A | 121 | 70.176 | 52.620 | 60.087 | 1.00 | 0.29 |
| ATOM | 1738 | CG1 | ILE | A | 121 | 70.818 | 55.007 | 58.872 | 1.00 | 0.29 |
| ATOM | 1739 | 1HG1 | ILE | A | 121 | 71.131 | 55.559 | 57.984 | 1.00 | 0.29 |
| ATOM | 1740 | 2HG1 | ILE | A | 121 | 71.677 | 54.438 | 59.229 | 1.00 | 0.29 |
| ATOM | 1741 | CD1 | ILE | A | 121 | 70.396 | 56.035 | 59.919 | 1.00 | 0.29 |
| ATOM | 1742 | HD1 | ILE | A | 121 | 71.246 | 56.669 | 60.166 | 1.00 | 0.29 |
| ATOM | 1743 | HD2 | ILE | A | 121 | 69.592 | 56.664 | 59.542 | 1.00 | 0.29 |
| ATOM | 1744 | HD3 | ILE | A | 121 | 70.068 | 55.568 | 60.842 | 1.00 | 0.29 |
| ATOM | 1745 | C | ILE | A | 121 | 69.025 | 52.262 | 56.704 | 1.00 | 0.29 |
| ATOM | 1746 | O | ILE | A | 121 | 68.219 | 52.774 | 55.926 | 1.00 | 0.29 |
| ATOM | 1747 | N | GLU | A | 122 | 69.031 | 50.991 | 57.136 | 1.00 | 0.42 |
| ATOM | 1748 | H | GLU | A | 122 | 69.911 | 50.689 | 57.538 | 1.00 | 0.42 |
| ATOM | 1749 | CA | GLU | A | 122 | 67.921 | 50.034 | 57.301 | 1.00 | 0.42 |
| ATOM | 1750 | HA | GLU | A | 122 | 67.589 | 49.759 | 56.310 | 1.00 | 0.42 |
| ATOM | 1751 | CB | GLU | A | 122 | 68.516 | 48.820 | 58.005 | 1.00 | 0.42 |
| ATOM | 1752 | HB1 | GLU | A | 122 | 67.941 | 48.545 | 58.900 | 1.00 | 0.42 |
| ATOM | 1753 | HB2 | GLU | A | 122 | 69.486 | 49.042 | 58.359 | 1.00 | 0.42 |
| ATOM | 1754 | CG | GLU | A | 122 | 68.656 | 47.551 | 57.146 | 1.00 | 0.42 |
| ATOM | 1755 | HG1 | GLU | A | 122 | 67.671 | 47.135 | 56.963 | 1.00 | 0.42 |
| ATOM | 1756 | HG2 | GLU | A | 122 | 69.218 | 46.823 | 57.714 | 1.00 | 0.42 |

| | | | | | | | | | | |
|---|---|---|---|---|---|---|---|---|---|---|
| ATOM | 1757 | CD | GLU | A | 122 | 69.321 | 47.832 | 55.804 | 1.00 | 0.42 |
| ATOM | 1758 | OE1 | GLU | A | 122 | 70.493 | 48.264 | 55.753 | 1.00 | 0.42 |
| ATOM | 1759 | OE2 | GLU | A | 122 | 68.626 | 47.700 | 54.776 | 1.00 | 0.42 |
| ATOM | 1760 | C | GLU | A | 122 | 66.672 | 50.678 | 58.037 | 1.00 | 0.42 |
| ATOM | 1761 | O | GLU | A | 122 | 66.473 | 51.921 | 57.867 | 1.00 | 0.42 |
| ATOM | 1762 | N | ASN | A | 123 | 65.713 | 49.979 | 58.768 | 1.00 | 0.56 |
| ATOM | 1763 | H | ASN | A | 123 | 64.915 | 50.591 | 58.803 | 1.00 | 0.56 |
| ATOM | 1764 | CA | ASN | A | 123 | 65.501 | 48.740 | 59.689 | 1.00 | 0.56 |
| ATOM | 1765 | HA | ASN | A | 123 | 64.441 | 48.851 | 59.975 | 1.00 | 0.56 |
| ATOM | 1766 | CB | ASN | A | 123 | 65.500 | 47.363 | 58.983 | 1.00 | 0.56 |
| ATOM | 1767 | HB1 | ASN | A | 123 | 65.064 | 46.636 | 59.662 | 1.00 | 0.56 |
| ATOM | 1768 | HB2 | ASN | A | 123 | 66.510 | 47.036 | 58.768 | 1.00 | 0.56 |
| ATOM | 1769 | CG | ASN | A | 123 | 64.631 | 47.361 | 57.721 | 1.00 | 0.56 |
| ATOM | 1770 | OD1 | ASN | A | 123 | 63.421 | 47.464 | 57.780 | 1.00 | 0.56 |
| ATOM | 1771 | ND2 | ASN | A | 123 | 65.202 | 47.276 | 56.547 | 1.00 | 0.56 |
| ATOM | 1772 | 1HD2 | ASN | A | 123 | 66.196 | 47.255 | 56.443 | 1.00 | 0.56 |
| ATOM | 1773 | 2HD2 | ASN | A | 123 | 64.632 | 47.583 | 55.776 | 1.00 | 0.56 |
| ATOM | 1774 | C | ASN | A | 123 | 66.153 | 48.929 | 61.051 | 1.00 | 0.56 |
| ATOM | 1775 | O | ASN | A | 123 | 66.812 | 48.038 | 61.592 | 1.00 | 0.56 |
| ATOM | 1776 | N | THR | A | 124 | 65.968 | 50.128 | 61.594 | 1.00 | 0.92 |
| ATOM | 1777 | H | THR | A | 124 | 65.447 | 50.789 | 61.036 | 1.00 | 0.92 |
| ATOM | 1778 | CA | THR | A | 124 | 66.238 | 50.477 | 62.998 | 1.00 | 0.92 |
| ATOM | 1779 | HA | THR | A | 124 | 67.271 | 50.230 | 63.213 | 1.00 | 0.92 |
| ATOM | 1780 | CB | THR | A | 124 | 66.050 | 51.984 | 63.220 | 1.00 | 0.92 |
| ATOM | 1781 | HB | THR | A | 124 | 66.228 | 52.207 | 64.273 | 1.00 | 0.92 |
| ATOM | 1782 | CG2 | THR | A | 124 | 67.027 | 52.807 | 62.382 | 1.00 | 0.92 |
| ATOM | 1783 | 1HG2 | THR | A | 124 | 66.959 | 53.848 | 62.683 | 1.00 | 0.92 |
| ATOM | 1784 | 2HG2 | THR | A | 124 | 68.044 | 52.460 | 62.558 | 1.00 | 0.92 |
| ATOM | 1785 | 3HG2 | THR | A | 124 | 66.785 | 52.720 | 61.322 | 1.00 | 0.92 |
| ATOM | 1786 | OG1 | THR | A | 124 | 64.746 | 52.391 | 62.874 | 1.00 | 0.92 |
| ATOM | 1787 | HG1 | THR | A | 124 | 64.590 | 52.224 | 61.931 | 1.00 | 0.92 |
| ATOM | 1788 | C | THR | A | 124 | 65.299 | 49.722 | 63.942 | 1.00 | 0.92 |
| ATOM | 1789 | O | THR | A | 124 | 64.137 | 49.508 | 63.595 | 1.00 | 0.92 |
| ATOM | 1790 | N | PRO | A | 125 | 65.761 | 49.353 | 65.151 | 1.00 | 0.87 |
| ATOM | 1791 | CD | PRO | A | 125 | 67.158 | 49.282 | 65.543 | 1.00 | 0.87 |
| ATOM | 1792 | HD1 | PRO | A | 125 | 67.701 | 50.194 | 65.294 | 1.00 | 0.87 |
| ATOM | 1793 | HD2 | PRO | A | 125 | 67.615 | 48.418 | 65.061 | 1.00 | 0.87 |
| ATOM | 1794 | CG | PRO | A | 125 | 67.169 | 49.074 | 67.044 | 1.00 | 0.87 |
| ATOM | 1795 | HG1 | PRO | A | 125 | 67.249 | 50.043 | 67.523 | 1.00 | 0.87 |
| ATOM | 1796 | HG2 | PRO | A | 125 | 67.996 | 48.434 | 67.335 | 1.00 | 0.87 |
| ATOM | 1797 | CB | PRO | A | 125 | 65.819 | 48.440 | 67.373 | 1.00 | 0.87 |
| ATOM | 1798 | HB1 | PRO | A | 125 | 65.447 | 48.802 | 68.330 | 1.00 | 0.87 |
| ATOM | 1799 | HB2 | PRO | A | 125 | 65.907 | 47.362 | 67.407 | 1.00 | 0.87 |
| ATOM | 1800 | CA | PRO | A | 125 | 64.903 | 48.808 | 66.203 | 1.00 | 0.87 |

| | | | | | | | | | | |
|---|---|---|---|---|---|---|---|---|---|---|
| ATOM | 1801 | HA | PRO | A | 125 | 64.429 | 47.897 | 65.833 | 1.00 | 0.87 |
| ATOM | 1802 | C | PRO | A | 125 | 63.791 | 49.771 | 66.608 | 1.00 | 0.87 |
| ATOM | 1803 | O | PRO | A | 125 | 62.613 | 49.525 | 66.370 | 1.00 | 0.87 |
| ATOM | 1804 | N | VAL | A | 126 | 64.192 | 50.911 | 67.152 | 1.00 | 0.78 |
| ATOM | 1805 | H | VAL | A | 126 | 65.187 | 51.038 | 67.296 | 1.00 | 0.78 |
| ATOM | 1806 | CA | VAL | A | 126 | 63.372 | 52.111 | 67.316 | 1.00 | 0.78 |
| ATOM | 1807 | HA | VAL | A | 126 | 62.545 | 52.094 | 66.606 | 1.00 | 0.78 |
| ATOM | 1808 | CB | VAL | A | 126 | 62.786 | 52.238 | 68.739 | 1.00 | 0.78 |
| ATOM | 1809 | HB | VAL | A | 126 | 62.202 | 53.152 | 68.795 | 1.00 | 0.78 |
| ATOM | 1810 | CG1 | VAL | A | 126 | 61.833 | 51.087 | 69.064 | 1.00 | 0.78 |
| ATOM | 1811 | 1HG1 | VAL | A | 126 | 61.386 | 51.245 | 70.043 | 1.00 | 0.78 |
| ATOM | 1812 | 2HG1 | VAL | A | 126 | 61.051 | 51.041 | 68.306 | 1.00 | 0.78 |
| ATOM | 1813 | 3HG1 | VAL | A | 126 | 62.377 | 50.144 | 69.061 | 1.00 | 0.78 |
| ATOM | 1814 | CG2 | VAL | A | 126 | 63.844 | 52.325 | 69.843 | 1.00 | 0.78 |
| ATOM | 1815 | 1HG2 | VAL | A | 126 | 63.363 | 52.445 | 70.814 | 1.00 | 0.78 |
| ATOM | 1816 | 2HG2 | VAL | A | 126 | 64.443 | 51.416 | 69.854 | 1.00 | 0.78 |
| ATOM | 1817 | 3HG2 | VAL | A | 126 | 64.504 | 53.172 | 69.667 | 1.00 | 0.78 |
| ATOM | 1818 | C | VAL | A | 126 | 64.273 | 53.308 | 66.968 | 1.00 | 0.78 |
| ATOM | 1819 | O | VAL | A | 126 | 65.485 | 53.172 | 66.800 | 1.00 | 0.78 |
| ATOM | 1820 | N | GLN | A | 127 | 63.643 | 54.471 | 66.876 | 1.00 | 0.47 |
| ATOM | 1821 | H | GLN | A | 127 | 62.633 | 54.428 | 66.979 | 1.00 | 0.47 |
| ATOM | 1822 | CA | GLN | A | 127 | 64.195 | 55.826 | 66.800 | 1.00 | 0.47 |
| ATOM | 1823 | HA | GLN | A | 127 | 63.554 | 56.344 | 66.083 | 1.00 | 0.47 |
| ATOM | 1824 | CB | GLN | A | 127 | 63.966 | 56.541 | 68.143 | 1.00 | 0.47 |
| ATOM | 1825 | HB1 | GLN | A | 127 | 64.255 | 57.587 | 68.035 | 1.00 | 0.47 |
| ATOM | 1826 | HB2 | GLN | A | 127 | 64.576 | 56.082 | 68.923 | 1.00 | 0.47 |
| ATOM | 1827 | CG | GLN | A | 127 | 62.479 | 56.470 | 68.530 | 1.00 | 0.47 |
| ATOM | 1828 | HG1 | GLN | A | 127 | 62.242 | 55.470 | 68.886 | 1.00 | 0.47 |
| ATOM | 1829 | HG2 | GLN | A | 127 | 61.881 | 56.677 | 67.644 | 1.00 | 0.47 |
| ATOM | 1830 | CD | GLN | A | 127 | 62.070 | 57.437 | 69.619 | 1.00 | 0.47 |
| ATOM | 1831 | OE1 | GLN | A | 127 | 62.678 | 57.496 | 70.687 | 1.00 | 0.47 |
| ATOM | 1832 | NE2 | GLN | A | 127 | 61.007 | 58.174 | 69.403 | 1.00 | 0.47 |
| ATOM | 1833 | 1HE2 | GLN | A | 127 | 60.456 | 58.047 | 68.540 | 1.00 | 0.47 |
| ATOM | 1834 | 2HE2 | GLN | A | 127 | 60.701 | 58.837 | 70.087 | 1.00 | 0.47 |
| ATOM | 1835 | C | GLN | A | 127 | 65.647 | 56.067 | 66.284 | 1.00 | 0.47 |
| ATOM | 1836 | O | GLN | A | 127 | 66.686 | 55.739 | 66.880 | 1.00 | 0.47 |
| ATOM | 1837 | N | ALA | A | 128 | 65.721 | 56.736 | 65.137 | 1.00 | 0.40 |
| ATOM | 1838 | H | ALA | A | 128 | 64.907 | 57.231 | 64.799 | 1.00 | 0.40 |
| ATOM | 1839 | CA | ALA | A | 128 | 66.752 | 56.492 | 64.151 | 1.00 | 0.40 |
| ATOM | 1840 | HA | ALA | A | 128 | 67.182 | 55.513 | 64.348 | 1.00 | 0.40 |
| ATOM | 1841 | CB | ALA | A | 128 | 66.092 | 56.419 | 62.769 | 1.00 | 0.40 |
| ATOM | 1842 | HB1 | ALA | A | 128 | 66.839 | 56.176 | 62.013 | 1.00 | 0.40 |
| ATOM | 1843 | HB2 | ALA | A | 128 | 65.312 | 55.656 | 62.761 | 1.00 | 0.40 |
| ATOM | 1844 | HB3 | ALA | A | 128 | 65.643 | 57.376 | 62.517 | 1.00 | 0.40 |

| | | | | | | | | | | |
|---|---|---|---|---|---|---|---|---|---|---|
| ATOM | 1845 | C | ALA | A | 128 | 67.879 | 57.500 | 64.164 | 1.00 | 0.40 |
| ATOM | 1846 | O | ALA | A | 128 | 69.029 | 57.081 | 64.223 | 1.00 | 0.40 |
| ATOM | 1847 | N | VAL | A | 129 | 67.571 | 58.796 | 64.166 | 1.00 | 0.62 |
| ATOM | 1848 | H | VAL | A | 129 | 66.612 | 59.068 | 63.979 | 1.00 | 0.62 |
| ATOM | 1849 | CA | VAL | A | 129 | 68.574 | 59.853 | 64.312 | 1.00 | 0.62 |
| ATOM | 1850 | HA | VAL | A | 129 | 69.507 | 59.427 | 64.672 | 1.00 | 0.62 |
| ATOM | 1851 | CB | VAL | A | 129 | 68.876 | 60.547 | 62.963 | 1.00 | 0.62 |
| ATOM | 1852 | HB | VAL | A | 129 | 68.003 | 61.114 | 62.643 | 1.00 | 0.62 |
| ATOM | 1853 | CG1 | VAL | A | 129 | 70.065 | 61.505 | 63.101 | 1.00 | 0.62 |
| ATOM | 1854 | 1HG1 | VAL | A | 129 | 70.245 | 62.016 | 62.156 | 1.00 | 0.62 |
| ATOM | 1855 | 2HG1 | VAL | A | 129 | 69.848 | 62.250 | 63.862 | 1.00 | 0.62 |
| ATOM | 1856 | 3HG1 | VAL | A | 129 | 70.961 | 60.956 | 63.391 | 1.00 | 0.62 |
| ATOM | 1857 | CG2 | VAL | A | 129 | 69.235 | 59.559 | 61.844 | 1.00 | 0.62 |
| ATOM | 1858 | 1HG2 | VAL | A | 129 | 69.451 | 60.109 | 60.930 | 1.00 | 0.62 |
| ATOM | 1859 | 2HG2 | VAL | A | 129 | 70.098 | 58.957 | 62.130 | 1.00 | 0.62 |
| ATOM | 1860 | 3HG2 | VAL | A | 129 | 68.389 | 58.903 | 61.642 | 1.00 | 0.62 |
| ATOM | 1861 | C | VAL | A | 129 | 68.100 | 60.836 | 65.369 | 1.00 | 0.62 |
| ATOM | 1862 | O | VAL | A | 129 | 67.244 | 61.671 | 65.084 | 1.00 | 0.62 |
| ATOM | 1863 | N | SER | A | 130 | 68.638 | 60.734 | 66.591 | 1.00 | 0.59 |
| ATOM | 1864 | H | SER | A | 130 | 69.460 | 60.145 | 66.716 | 1.00 | 0.59 |
| ATOM | 1865 | CA | SER | A | 130 | 68.389 | 61.824 | 67.541 | 1.00 | 0.59 |
| ATOM | 1866 | HA | SER | A | 130 | 67.363 | 62.179 | 67.450 | 1.00 | 0.59 |
| ATOM | 1867 | CB | SER | A | 130 | 68.612 | 61.394 | 68.990 | 1.00 | 0.59 |
| ATOM | 1868 | HB1 | SER | A | 130 | 69.658 | 61.120 | 69.134 | 1.00 | 0.59 |
| ATOM | 1869 | HB2 | SER | A | 130 | 67.986 | 60.526 | 69.202 | 1.00 | 0.59 |
| ATOM | 1870 | OG | SER | A | 130 | 68.264 | 62.439 | 69.882 | 1.00 | 0.59 |
| ATOM | 1871 | HG | SER | A | 130 | 67.316 | 62.705 | 69.716 | 1.00 | 0.59 |
| ATOM | 1872 | C | SER | A | 130 | 69.321 | 62.947 | 67.136 | 1.00 | 0.59 |
| ATOM | 1873 | O | SER | A | 130 | 70.381 | 62.676 | 66.582 | 1.00 | 0.59 |
| ATOM | 1874 | N | ILE | A | 131 | 68.943 | 64.192 | 67.358 | 1.00 | 0.45 |
| ATOM | 1875 | H | ILE | A | 131 | 67.999 | 64.358 | 67.686 | 1.00 | 0.45 |
| ATOM | 1876 | CA | ILE | A | 131 | 69.748 | 65.345 | 66.990 | 1.00 | 0.45 |
| ATOM | 1877 | HA | ILE | A | 131 | 70.791 | 65.049 | 66.922 | 1.00 | 0.45 |
| ATOM | 1878 | CB | ILE | A | 131 | 69.341 | 65.965 | 65.628 | 1.00 | 0.45 |
| ATOM | 1879 | HB | ILE | A | 131 | 68.340 | 66.392 | 65.703 | 1.00 | 0.45 |
| ATOM | 1880 | CG2 | ILE | A | 131 | 70.348 | 67.094 | 65.344 | 1.00 | 0.45 |
| ATOM | 1881 | 1HG2 | ILE | A | 131 | 70.157 | 67.558 | 64.382 | 1.00 | 0.45 |
| ATOM | 1882 | 2HG2 | ILE | A | 131 | 70.266 | 67.879 | 66.096 | 1.00 | 0.45 |
| ATOM | 1883 | 3HG2 | ILE | A | 131 | 71.357 | 66.683 | 65.363 | 1.00 | 0.45 |
| ATOM | 1884 | CG1 | ILE | A | 131 | 69.343 | 64.940 | 64.471 | 1.00 | 0.45 |
| ATOM | 1885 | 1HG1 | ILE | A | 131 | 68.659 | 64.130 | 64.721 | 1.00 | 0.45 |
| ATOM | 1886 | 2HG1 | ILE | A | 131 | 70.346 | 64.525 | 64.367 | 1.00 | 0.45 |
| ATOM | 1887 | CD1 | ILE | A | 131 | 68.891 | 65.484 | 63.108 | 1.00 | 0.45 |
| ATOM | 1888 | HD1 | ILE | A | 131 | 69.567 | 66.257 | 62.749 | 1.00 | 0.45 |

| | | | | | | | | | | |
|---|---|---|---|---|---|---|---|---|---|---|
| ATOM | 1889 | HD2 | ILE | A | 131 | 68.890 | 64.671 | 62.384 | 1.00 | 0.45 |
| ATOM | 1890 | HD3 | ILE | A | 131 | 67.883 | 65.890 | 63.185 | 1.00 | 0.45 |
| ATOM | 1891 | C | ILE | A | 131 | 69.595 | 66.353 | 68.117 | 1.00 | 0.45 |
| ATOM | 1892 | O | ILE | A | 131 | 68.557 | 67.002 | 68.251 | 1.00 | 0.45 |
| ATOM | 1893 | N | ASN | A | 132 | 70.630 | 66.461 | 68.944 | 1.00 | 0.85 |
| ATOM | 1894 | H | ASN | A | 132 | 71.489 | 65.961 | 68.742 | 1.00 | 0.85 |
| ATOM | 1895 | CA | ASN | A | 132 | 70.531 | 67.217 | 70.188 | 1.00 | 0.85 |
| ATOM | 1896 | HA | ASN | A | 132 | 69.747 | 67.963 | 70.098 | 1.00 | 0.85 |
| ATOM | 1897 | CB | ASN | A | 132 | 70.119 | 66.268 | 71.338 | 1.00 | 0.85 |
| ATOM | 1898 | HB1 | ASN | A | 132 | 70.196 | 65.234 | 71.026 | 1.00 | 0.85 |
| ATOM | 1899 | HB2 | ASN | A | 132 | 69.066 | 66.442 | 71.561 | 1.00 | 0.85 |
| ATOM | 1900 | CG | ASN | A | 132 | 70.930 | 66.420 | 72.619 | 1.00 | 0.85 |
| ATOM | 1901 | OD1 | ASN | A | 132 | 72.084 | 66.047 | 72.709 | 1.00 | 0.85 |
| ATOM | 1902 | ND2 | ASN | A | 132 | 70.355 | 66.995 | 73.646 | 1.00 | 0.85 |
| ATOM | 1903 | 1HD2 | ASN | A | 132 | 69.391 | 67.343 | 73.570 | 1.00 | 0.85 |
| ATOM | 1904 | 2HD2 | ASN | A | 132 | 70.885 | 67.079 | 74.488 | 1.00 | 0.85 |
| ATOM | 1905 | C | ASN | A | 132 | 71.823 | 67.989 | 70.467 | 1.00 | 0.85 |
| ATOM | 1906 | O | ASN | A | 132 | 72.930 | 67.547 | 70.144 | 1.00 | 0.85 |
| ATOM | 1907 | N | GLY | A | 133 | 71.683 | 69.178 | 71.056 | 1.00 | 1.11 |
| ATOM | 1908 | H | GLY | A | 133 | 70.765 | 69.553 | 71.253 | 1.00 | 1.11 |
| ATOM | 1909 | CA | GLY | A | 133 | 72.829 | 70.011 | 71.391 | 1.00 | 1.11 |
| ATOM | 1910 | HA1 | GLY | A | 133 | 73.380 | 69.543 | 72.207 | 1.00 | 1.11 |
| ATOM | 1911 | HA2 | GLY | A | 133 | 72.490 | 70.989 | 71.730 | 1.00 | 1.11 |
| ATOM | 1912 | C | GLY | A | 133 | 73.783 | 70.209 | 70.230 | 1.00 | 1.11 |
| ATOM | 1913 | O | GLY | A | 133 | 74.984 | 70.107 | 70.420 | 1.00 | 1.11 |
| ATOM | 1914 | N | CYS | A | 134 | 73.244 | 70.358 | 69.022 | 1.00 | 0.94 |
| ATOM | 1915 | H | CYS | A | 134 | 72.246 | 70.455 | 68.978 | 1.00 | 0.94 |
| ATOM | 1916 | CA | CYS | A | 134 | 74.007 | 70.501 | 67.791 | 1.00 | 0.94 |
| ATOM | 1917 | HA | CYS | A | 134 | 75.065 | 70.345 | 68.006 | 1.00 | 0.94 |
| ATOM | 1918 | CB | CYS | A | 134 | 73.552 | 69.467 | 66.754 | 1.00 | 0.94 |
| ATOM | 1919 | HB1 | CYS | A | 134 | 74.007 | 69.710 | 65.793 | 1.00 | 0.94 |
| ATOM | 1920 | HB2 | CYS | A | 134 | 72.466 | 69.493 | 66.650 | 1.00 | 0.94 |
| ATOM | 1921 | SG | CYS | A | 134 | 74.096 | 67.810 | 67.236 | 1.00 | 0.94 |
| ATOM | 1922 | HG | CYS | A | 134 | 73.532 | 67.802 | 68.452 | 1.00 | 0.94 |
| ATOM | 1923 | C | CYS | A | 134 | 73.851 | 71.912 | 67.247 | 1.00 | 0.94 |
| ATOM | 1924 | O | CYS | A | 134 | 72.773 | 72.492 | 67.382 | 1.00 | 0.94 |
| ATOM | 1925 | N | ASP | A | 135 | 74.879 | 72.441 | 66.584 | 1.00 | 0.93 |
| ATOM | 1926 | H | ASP | A | 135 | 75.748 | 71.921 | 66.505 | 1.00 | 0.93 |
| ATOM | 1927 | CA | ASP | A | 135 | 74.784 | 73.766 | 65.967 | 1.00 | 0.93 |
| ATOM | 1928 | HA | ASP | A | 135 | 73.733 | 73.981 | 65.807 | 1.00 | 0.93 |
| ATOM | 1929 | CB | ASP | A | 135 | 75.297 | 74.868 | 66.910 | 1.00 | 0.93 |
| ATOM | 1930 | HB1 | ASP | A | 135 | 76.347 | 75.083 | 66.711 | 1.00 | 0.93 |
| ATOM | 1931 | HB2 | ASP | A | 135 | 75.215 | 74.528 | 67.944 | 1.00 | 0.93 |
| ATOM | 1932 | CG | ASP | A | 135 | 74.469 | 76.151 | 66.753 | 1.00 | 0.93 |

| | | | | | | | | | | |
|---|---|---|---|---|---|---|---|---|---|---|
| ATOM | 1933 | OD1 | ASP | A | 135 | 74.318 | 76.649 | 65.610 | 1.00 | 0.93 |
| ATOM | 1934 | OD2 | ASP | A | 135 | 73.900 | 76.602 | 67.772 | 1.00 | 0.93 |
| ATOM | 1935 | C | ASP | A | 135 | 75.460 | 73.818 | 64.598 | 1.00 | 0.93 |
| ATOM | 1936 | O | ASP | A | 135 | 76.664 | 73.567 | 64.468 | 1.00 | 0.93 |
| ATOM | 1937 | N | GLY | A | 136 | 74.668 | 74.093 | 63.558 | 1.00 | 0.92 |
| ATOM | 1938 | H | GLY | A | 136 | 73.685 | 74.282 | 63.729 | 1.00 | 0.92 |
| ATOM | 1939 | CA | GLY | A | 136 | 75.169 | 74.067 | 62.186 | 1.00 | 0.92 |
| ATOM | 1940 | HA1 | GLY | A | 136 | 76.107 | 74.621 | 62.129 | 1.00 | 0.92 |
| ATOM | 1941 | HA2 | GLY | A | 136 | 74.451 | 74.541 | 61.520 | 1.00 | 0.92 |
| ATOM | 1942 | C | GLY | A | 136 | 75.423 | 72.637 | 61.710 | 1.00 | 0.92 |
| ATOM | 1943 | O | GLY | A | 136 | 76.556 | 72.227 | 61.467 | 1.00 | 0.92 |
| ATOM | 1944 | N | LEU | A | 137 | 74.358 | 71.844 | 61.625 | 1.00 | 0.43 |
| ATOM | 1945 | H | LEU | A | 137 | 73.453 | 72.266 | 61.806 | 1.00 | 0.43 |
| ATOM | 1946 | CA | LEU | A | 137 | 74.431 | 70.424 | 61.295 | 1.00 | 0.43 |
| ATOM | 1947 | HA | LEU | A | 137 | 75.460 | 70.162 | 61.052 | 1.00 | 0.43 |
| ATOM | 1948 | CB | LEU | A | 137 | 74.032 | 69.617 | 62.541 | 1.00 | 0.43 |
| ATOM | 1949 | HB1 | LEU | A | 137 | 73.046 | 69.954 | 62.867 | 1.00 | 0.43 |
| ATOM | 1950 | HB2 | LEU | A | 137 | 74.742 | 69.840 | 63.340 | 1.00 | 0.43 |
| ATOM | 1951 | CG | LEU | A | 137 | 73.975 | 68.093 | 62.330 | 1.00 | 0.43 |
| ATOM | 1952 | HG | LEU | A | 137 | 73.219 | 67.857 | 61.584 | 1.00 | 0.43 |
| ATOM | 1953 | CD1 | LEU | A | 137 | 75.311 | 67.496 | 61.892 | 1.00 | 0.43 |
| ATOM | 1954 | 1HD1 | LEU | A | 137 | 75.249 | 66.410 | 61.911 | 1.00 | 0.43 |
| ATOM | 1955 | 2HD1 | LEU | A | 137 | 75.537 | 67.805 | 60.874 | 1.00 | 0.43 |
| ATOM | 1956 | 3HD1 | LEU | A | 137 | 76.098 | 67.833 | 62.565 | 1.00 | 0.43 |
| ATOM | 1957 | CD2 | LEU | A | 137 | 73.571 | 67.434 | 63.642 | 1.00 | 0.43 |
| ATOM | 1958 | 1HD2 | LEU | A | 137 | 73.371 | 66.375 | 63.481 | 1.00 | 0.43 |
| ATOM | 1959 | 2HD2 | LEU | A | 137 | 74.368 | 67.546 | 64.374 | 1.00 | 0.43 |
| ATOM | 1960 | 3HD2 | LEU | A | 137 | 72.673 | 67.916 | 64.023 | 1.00 | 0.43 |
| ATOM | 1961 | C | LEU | A | 137 | 73.559 | 70.109 | 60.071 | 1.00 | 0.43 |
| ATOM | 1962 | O | LEU | A | 137 | 72.327 | 70.152 | 60.121 | 1.00 | 0.43 |
| ATOM | 1963 | N | THR | A | 138 | 74.217 | 69.735 | 58.977 | 1.00 | 0.45 |
| ATOM | 1964 | H | THR | A | 138 | 75.226 | 69.643 | 59.044 | 1.00 | 0.45 |
| ATOM | 1965 | CA | THR | A | 138 | 73.564 | 69.264 | 57.750 | 1.00 | 0.45 |
| ATOM | 1966 | HA | THR | A | 138 | 72.560 | 69.675 | 57.694 | 1.00 | 0.45 |
| ATOM | 1967 | CB | THR | A | 138 | 74.328 | 69.718 | 56.490 | 1.00 | 0.45 |
| ATOM | 1968 | HB | THR | A | 138 | 73.937 | 69.177 | 55.628 | 1.00 | 0.45 |
| ATOM | 1969 | CG2 | THR | A | 138 | 74.186 | 71.213 | 56.214 | 1.00 | 0.45 |
| ATOM | 1970 | 1HG2 | THR | A | 138 | 74.893 | 71.514 | 55.440 | 1.00 | 0.45 |
| ATOM | 1971 | 2HG2 | THR | A | 138 | 73.182 | 71.416 | 55.847 | 1.00 | 0.45 |
| ATOM | 1972 | 3HG2 | THR | A | 138 | 74.373 | 71.793 | 57.119 | 1.00 | 0.45 |
| ATOM | 1973 | OG1 | THR | A | 138 | 75.710 | 69.472 | 56.575 | 1.00 | 0.45 |
| ATOM | 1974 | HG1 | THR | A | 138 | 76.073 | 70.081 | 57.232 | 1.00 | 0.45 |
| ATOM | 1975 | C | THR | A | 138 | 73.441 | 67.734 | 57.731 | 1.00 | 0.45 |
| ATOM | 1976 | O | THR | A | 138 | 74.343 | 67.039 | 58.210 | 1.00 | 0.45 |

| | | | | | | | | | | |
|---|---|---|---|---|---|---|---|---|---|---|
| ATOM | 1977 | N | ILE | A | 139 | 72.337 | 67.186 | 57.182 | 1.00 | 0.45 |
| ATOM | 1978 | H | ILE | A | 139 | 71.574 | 67.749 | 56.841 | 1.00 | 0.45 |
| ATOM | 1979 | CA | ILE | A | 139 | 72.046 | 65.749 | 57.238 | 1.00 | 0.45 |
| ATOM | 1980 | HA | ILE | A | 139 | 72.740 | 65.319 | 57.952 | 1.00 | 0.45 |
| ATOM | 1981 | CB | ILE | A | 139 | 70.642 | 65.500 | 57.840 | 1.00 | 0.45 |
| ATOM | 1982 | HB | ILE | A | 139 | 70.093 | 66.439 | 57.826 | 1.00 | 0.45 |
| ATOM | 1983 | CG2 | ILE | A | 139 | 69.804 | 64.474 | 57.066 | 1.00 | 0.45 |
| ATOM | 1984 | 1HG2 | ILE | A | 139 | 69.662 | 64.794 | 56.034 | 1.00 | 0.45 |
| ATOM | 1985 | 2HG2 | ILE | A | 139 | 70.299 | 63.503 | 57.088 | 1.00 | 0.45 |
| ATOM | 1986 | 3HG2 | ILE | A | 139 | 68.834 | 64.364 | 57.545 | 1.00 | 0.45 |
| ATOM | 1987 | CG1 | ILE | A | 139 | 70.725 | 65.028 | 59.311 | 1.00 | 0.45 |
| ATOM | 1988 | 1HG1 | ILE | A | 139 | 71.275 | 64.087 | 59.357 | 1.00 | 0.45 |
| ATOM | 1989 | 2HG1 | ILE | A | 139 | 69.717 | 64.840 | 59.683 | 1.00 | 0.45 |
| ATOM | 1990 | CD1 | ILE | A | 139 | 71.388 | 66.035 | 60.259 | 1.00 | 0.45 |
| ATOM | 1991 | HD1 | ILE | A | 139 | 71.340 | 65.653 | 61.279 | 1.00 | 0.45 |
| ATOM | 1992 | HD2 | ILE | A | 139 | 72.437 | 66.171 | 60.002 | 1.00 | 0.45 |
| ATOM | 1993 | HD3 | ILE | A | 139 | 70.871 | 66.994 | 60.209 | 1.00 | 0.45 |
| ATOM | 1994 | C | ILE | A | 139 | 72.371 | 64.942 | 55.931 | 1.00 | 0.45 |
| ATOM | 1995 | O | ILE | A | 139 | 72.595 | 63.727 | 55.963 | 1.00 | 0.45 |
| ATOM | 1996 | N | THR | A | 140 | 72.465 | 65.654 | 54.804 | 1.00 | 0.50 |
| ATOM | 1997 | H | THR | A | 140 | 72.722 | 66.614 | 54.954 | 1.00 | 0.50 |
| ATOM | 1998 | CA | THR | A | 140 | 71.881 | 65.358 | 53.473 | 1.00 | 0.50 |
| ATOM | 1999 | HA | THR | A | 140 | 70.823 | 65.589 | 53.569 | 1.00 | 0.50 |
| ATOM | 2000 | CB | THR | A | 140 | 72.440 | 66.376 | 52.452 | 1.00 | 0.50 |
| ATOM | 2001 | HB | THR | A | 140 | 72.373 | 67.369 | 52.894 | 1.00 | 0.50 |
| ATOM | 2002 | CG2 | THR | A | 140 | 73.909 | 66.154 | 52.083 | 1.00 | 0.50 |
| ATOM | 2003 | 1HG2 | THR | A | 140 | 74.251 | 66.978 | 51.457 | 1.00 | 0.50 |
| ATOM | 2004 | 2HG2 | THR | A | 140 | 74.514 | 66.127 | 52.990 | 1.00 | 0.50 |
| ATOM | 2005 | 3HG2 | THR | A | 140 | 74.038 | 65.217 | 51.542 | 1.00 | 0.50 |
| ATOM | 2006 | OG1 | THR | A | 140 | 71.704 | 66.393 | 51.245 | 1.00 | 0.50 |
| ATOM | 2007 | HG1 | THR | A | 140 | 71.993 | 65.635 | 50.727 | 1.00 | 0.50 |
| ATOM | 2008 | C | THR | A | 140 | 71.960 | 63.966 | 52.845 | 1.00 | 0.50 |
| ATOM | 2009 | O | THR | A | 140 | 72.950 | 63.249 | 52.917 | 1.00 | 0.50 |
| ATOM | 2010 | N | ASP | A | 141 | 70.915 | 63.647 | 52.071 | 1.00 | 0.52 |
| ATOM | 2011 | H | ASP | A | 141 | 70.069 | 64.183 | 52.214 | 1.00 | 0.52 |
| ATOM | 2012 | CA | ASP | A | 141 | 70.707 | 62.368 | 51.406 | 1.00 | 0.52 |
| ATOM | 2013 | HA | ASP | A | 141 | 69.646 | 62.362 | 51.160 | 1.00 | 0.52 |
| ATOM | 2014 | CB | ASP | A | 141 | 71.462 | 62.222 | 50.078 | 1.00 | 0.52 |
| ATOM | 2015 | HB1 | ASP | A | 141 | 72.464 | 61.834 | 50.255 | 1.00 | 0.52 |
| ATOM | 2016 | HB2 | ASP | A | 141 | 71.540 | 63.196 | 49.592 | 1.00 | 0.52 |
| ATOM | 2017 | CG | ASP | A | 141 | 70.644 | 61.293 | 49.181 | 1.00 | 0.52 |
| ATOM | 2018 | OD1 | ASP | A | 141 | 69.490 | 61.675 | 48.876 | 1.00 | 0.52 |
| ATOM | 2019 | OD2 | ASP | A | 141 | 71.028 | 60.125 | 48.952 | 1.00 | 0.52 |
| ATOM | 2020 | C | ASP | A | 141 | 70.876 | 61.240 | 52.430 | 1.00 | 0.52 |

| | | | | | | | | | | |
|---|---|---|---|---|---|---|---|---|---|---|
| ATOM | 2021 | O | ASP | A | 141 | 71.604 | 60.247 | 52.299 | 1.00 | 0.52 |
| ATOM | 2022 | N | MET | A | 142 | 70.163 | 61.446 | 53.532 | 1.00 | 0.59 |
| ATOM | 2023 | H | MET | A | 142 | 69.657 | 62.306 | 53.652 | 1.00 | 0.59 |
| ATOM | 2024 | CA | MET | A | 142 | 69.835 | 60.282 | 54.341 | 1.00 | 0.59 |
| ATOM | 2025 | HA | MET | A | 142 | 70.736 | 59.696 | 54.542 | 1.00 | 0.59 |
| ATOM | 2026 | CB | MET | A | 142 | 69.222 | 60.763 | 55.667 | 1.00 | 0.59 |
| ATOM | 2027 | HB1 | MET | A | 142 | 68.136 | 60.691 | 55.606 | 1.00 | 0.59 |
| ATOM | 2028 | HB2 | MET | A | 142 | 69.473 | 61.808 | 55.840 | 1.00 | 0.59 |
| ATOM | 2029 | CG | MET | A | 142 | 69.702 | 59.954 | 56.871 | 1.00 | 0.59 |
| ATOM | 2030 | HG1 | MET | A | 142 | 69.662 | 58.891 | 56.636 | 1.00 | 0.59 |
| ATOM | 2031 | HG2 | MET | A | 142 | 69.024 | 60.148 | 57.700 | 1.00 | 0.59 |
| ATOM | 2032 | SD | MET | A | 142 | 71.369 | 60.374 | 57.428 | 1.00 | 0.59 |
| ATOM | 2033 | CE | MET | A | 142 | 71.573 | 59.067 | 58.662 | 1.00 | 0.59 |
| ATOM | 2034 | HE1 | MET | A | 142 | 71.526 | 58.097 | 58.167 | 1.00 | 0.59 |
| ATOM | 2035 | HE2 | MET | A | 142 | 72.534 | 59.176 | 59.162 | 1.00 | 0.59 |
| ATOM | 2036 | HE3 | MET | A | 142 | 70.774 | 59.129 | 59.398 | 1.00 | 0.59 |
| ATOM | 2037 | C | MET | A | 142 | 68.811 | 59.420 | 53.580 | 1.00 | 0.59 |
| ATOM | 2038 | O | MET | A | 142 | 68.195 | 59.829 | 52.574 | 1.00 | 0.59 |
| ATOM | 2039 | N | THR | A | 143 | 68.670 | 58.219 | 54.126 | 1.00 | 0.31 |
| ATOM | 2040 | H | THR | A | 143 | 69.223 | 58.015 | 54.954 | 1.00 | 0.31 |
| ATOM | 2041 | CA | THR | A | 143 | 67.979 | 57.051 | 53.642 | 1.00 | 0.31 |
| ATOM | 2042 | HA | THR | A | 143 | 66.961 | 57.275 | 53.347 | 1.00 | 0.31 |
| ATOM | 2043 | CB | THR | A | 143 | 68.776 | 56.341 | 52.534 | 1.00 | 0.31 |
| ATOM | 2044 | HB | THR | A | 143 | 69.737 | 56.029 | 52.928 | 1.00 | 0.31 |
| ATOM | 2045 | CG2 | THR | A | 143 | 68.048 | 55.115 | 51.995 | 1.00 | 0.31 |
| ATOM | 2046 | 1HG2 | THR | A | 143 | 67.023 | 55.363 | 51.725 | 1.00 | 0.31 |
| ATOM | 2047 | 2HG2 | THR | A | 143 | 68.570 | 54.728 | 51.120 | 1.00 | 0.31 |
| ATOM | 2048 | 3HG2 | THR | A | 143 | 68.032 | 54.333 | 52.753 | 1.00 | 0.31 |
| ATOM | 2049 | OG1 | THR | A | 143 | 69.045 | 57.198 | 51.449 | 1.00 | 0.31 |
| ATOM | 2050 | HG1 | THR | A | 143 | 69.870 | 57.650 | 51.670 | 1.00 | 0.31 |
| ATOM | 2051 | C | THR | A | 143 | 68.020 | 56.182 | 54.902 | 1.00 | 0.31 |
| ATOM | 2052 | O | THR | A | 143 | 68.960 | 56.188 | 55.693 | 1.00 | 0.31 |
| ATOM | 2053 | N | ILE | A | 144 | 66.929 | 55.506 | 55.121 | 1.00 | 0.57 |
| ATOM | 2054 | H | ILE | A | 144 | 66.357 | 55.354 | 54.302 | 1.00 | 0.57 |
| ATOM | 2055 | CA | ILE | A | 144 | 66.143 | 55.334 | 56.341 | 1.00 | 0.57 |
| ATOM | 2056 | HA | ILE | A | 144 | 66.595 | 54.577 | 56.983 | 1.00 | 0.57 |
| ATOM | 2057 | CB | ILE | A | 144 | 65.816 | 56.626 | 57.160 | 1.00 | 0.57 |
| ATOM | 2058 | HB | ILE | A | 144 | 65.269 | 57.318 | 56.520 | 1.00 | 0.57 |
| ATOM | 2059 | CG2 | ILE | A | 144 | 64.906 | 56.230 | 58.338 | 1.00 | 0.57 |
| ATOM | 2060 | 1HG2 | ILE | A | 144 | 64.511 | 57.114 | 58.837 | 1.00 | 0.57 |
| ATOM | 2061 | 2HG2 | ILE | A | 144 | 64.053 | 55.675 | 57.972 | 1.00 | 0.57 |
| ATOM | 2062 | 3HG2 | ILE | A | 144 | 65.460 | 55.618 | 59.051 | 1.00 | 0.57 |
| ATOM | 2063 | CG1 | ILE | A | 144 | 67.052 | 57.372 | 57.710 | 1.00 | 0.57 |
| ATOM | 2064 | 1HG1 | ILE | A | 144 | 67.534 | 57.886 | 56.882 | 1.00 | 0.57 |

| | | | | | | | | | | |
|---|---|---|---|---|---|---|---|---|---|---|
| ATOM | 2065 | 2HG1 | ILE | A | 144 | 67.747 | 56.645 | 58.125 | 1.00 | 0.57 |
| ATOM | 2066 | CD1 | ILE | A | 144 | 66.792 | 58.447 | 58.781 | 1.00 | 0.57 |
| ATOM | 2067 | HD1 | ILE | A | 144 | 67.724 | 58.961 | 59.012 | 1.00 | 0.57 |
| ATOM | 2068 | HD2 | ILE | A | 144 | 66.065 | 59.173 | 58.414 | 1.00 | 0.57 |
| ATOM | 2069 | HD3 | ILE | A | 144 | 66.432 | 57.994 | 59.705 | 1.00 | 0.57 |
| ATOM | 2070 | C | ILE | A | 144 | 64.922 | 54.802 | 55.624 | 1.00 | 0.57 |
| ATOM | 2071 | O | ILE | A | 144 | 64.434 | 55.521 | 54.709 | 1.00 | 0.57 |
| ATOM | 2072 | N | ASP | A | 145 | 64.696 | 53.523 | 55.963 | 1.00 | 0.56 |
| ATOM | 2073 | H | ASP | A | 145 | 65.225 | 53.232 | 56.778 | 1.00 | 0.56 |
| ATOM | 2074 | CA | ASP | A | 145 | 64.259 | 52.348 | 55.168 | 1.00 | 0.56 |
| ATOM | 2075 | HA | ASP | A | 145 | 63.357 | 52.659 | 54.653 | 1.00 | 0.56 |
| ATOM | 2076 | CB | ASP | A | 145 | 65.237 | 51.935 | 54.076 | 1.00 | 0.56 |
| ATOM | 2077 | HB1 | ASP | A | 145 | 66.104 | 51.452 | 54.528 | 1.00 | 0.56 |
| ATOM | 2078 | HB2 | ASP | A | 145 | 65.572 | 52.826 | 53.541 | 1.00 | 0.56 |
| ATOM | 2079 | CG | ASP | A | 145 | 64.567 | 50.985 | 53.068 | 1.00 | 0.56 |
| ATOM | 2080 | OD1 | ASP | A | 145 | 63.360 | 50.640 | 53.214 | 1.00 | 0.56 |
| ATOM | 2081 | OD2 | ASP | A | 145 | 65.301 | 50.642 | 52.113 | 1.00 | 0.56 |
| ATOM | 2082 | C | ASP | A | 145 | 63.858 | 51.188 | 56.081 | 1.00 | 0.56 |
| ATOM | 2083 | O | ASP | A | 145 | 64.390 | 50.083 | 56.075 | 1.00 | 0.56 |
| ATOM | 2084 | N | ASN | A | 146 | 62.917 | 51.510 | 56.955 | 1.00 | 0.73 |
| ATOM | 2085 | H | ASN | A | 146 | 62.526 | 52.443 | 56.873 | 1.00 | 0.73 |
| ATOM | 2086 | CA | ASN | A | 146 | 62.280 | 50.641 | 57.929 | 1.00 | 0.73 |
| ATOM | 2087 | HA | ASN | A | 146 | 62.955 | 49.813 | 58.137 | 1.00 | 0.73 |
| ATOM | 2088 | CB | ASN | A | 146 | 62.051 | 51.422 | 59.245 | 1.00 | 0.73 |
| ATOM | 2089 | HB1 | ASN | A | 146 | 61.927 | 50.711 | 60.062 | 1.00 | 0.73 |
| ATOM | 2090 | HB2 | ASN | A | 146 | 61.126 | 51.989 | 59.178 | 1.00 | 0.73 |
| ATOM | 2091 | CG | ASN | A | 146 | 63.178 | 52.377 | 59.593 | 1.00 | 0.73 |
| ATOM | 2092 | OD1 | ASN | A | 146 | 64.158 | 52.009 | 60.233 | 1.00 | 0.73 |
| ATOM | 2093 | ND2 | ASN | A | 146 | 63.097 | 53.607 | 59.136 | 1.00 | 0.73 |
| ATOM | 2094 | 1HD2 | ASN | A | 146 | 62.320 | 53.857 | 58.532 | 1.00 | 0.73 |
| ATOM | 2095 | 2HD2 | ASN | A | 146 | 63.898 | 54.202 | 59.251 | 1.00 | 0.73 |
| ATOM | 2096 | C | ASN | A | 146 | 60.993 | 50.035 | 57.384 | 1.00 | 0.73 |
| ATOM | 2097 | O | ASN | A | 146 | 60.148 | 49.635 | 58.170 | 1.00 | 0.73 |
| ATOM | 2098 | N | SER | A | 147 | 60.841 | 49.971 | 56.064 | 1.00 | 0.95 |
| ATOM | 2099 | H | SER | A | 147 | 61.630 | 50.245 | 55.496 | 1.00 | 0.95 |
| ATOM | 2100 | CA | SER | A | 147 | 59.700 | 49.385 | 55.353 | 1.00 | 0.95 |
| ATOM | 2101 | HA | SER | A | 147 | 58.851 | 50.067 | 55.429 | 1.00 | 0.95 |
| ATOM | 2102 | CB | SER | A | 147 | 60.105 | 49.268 | 53.870 | 1.00 | 0.95 |
| ATOM | 2103 | HB1 | SER | A | 147 | 60.120 | 50.264 | 53.426 | 1.00 | 0.95 |
| ATOM | 2104 | HB2 | SER | A | 147 | 59.367 | 48.663 | 53.341 | 1.00 | 0.95 |
| ATOM | 2105 | OG | SER | A | 147 | 61.398 | 48.683 | 53.719 | 1.00 | 0.95 |
| ATOM | 2106 | HG | SER | A | 147 | 62.054 | 49.363 | 53.437 | 1.00 | 0.95 |
| ATOM | 2107 | C | SER | A | 147 | 59.247 | 48.037 | 55.933 | 1.00 | 0.95 |
| ATOM | 2108 | O | SER | A | 147 | 58.062 | 47.845 | 56.188 | 1.00 | 0.95 |

| | | | | | | | | | | |
|---|---|---|---|---|---|---|---|---|---|---|
| ATOM | 2109 | N | ALA | A | 148 | 60.181 | 47.126 | 56.234 | 1.00 | 1.21 |
| ATOM | 2110 | H | ALA | A | 148 | 61.142 | 47.340 | 56.007 | 1.00 | 1.21 |
| ATOM | 2111 | CA | ALA | A | 148 | 59.860 | 45.823 | 56.821 | 1.00 | 1.21 |
| ATOM | 2112 | HA | ALA | A | 148 | 59.237 | 45.289 | 56.102 | 1.00 | 1.21 |
| ATOM | 2113 | CB | ALA | A | 148 | 61.153 | 45.019 | 57.000 | 1.00 | 1.21 |
| ATOM | 2114 | HB1 | ALA | A | 148 | 60.907 | 44.005 | 57.318 | 1.00 | 1.21 |
| ATOM | 2115 | HB2 | ALA | A | 148 | 61.699 | 44.973 | 56.057 | 1.00 | 1.21 |
| ATOM | 2116 | HB3 | ALA | A | 148 | 61.782 | 45.483 | 57.758 | 1.00 | 1.21 |
| ATOM | 2117 | C | ALA | A | 148 | 59.040 | 45.893 | 58.132 | 1.00 | 1.21 |
| ATOM | 2118 | O | ALA | A | 148 | 58.165 | 45.060 | 58.404 | 1.00 | 1.21 |
| ATOM | 2119 | N | GLY | A | 149 | 59.301 | 46.942 | 58.910 | 1.00 | 1.27 |
| ATOM | 2120 | H | GLY | A | 149 | 59.940 | 47.639 | 58.547 | 1.00 | 1.27 |
| ATOM | 2121 | CA | GLY | A | 149 | 58.598 | 47.299 | 60.129 | 1.00 | 1.27 |
| ATOM | 2122 | HA1 | GLY | A | 149 | 58.895 | 48.306 | 60.420 | 1.00 | 1.27 |
| ATOM | 2123 | HA2 | GLY | A | 149 | 58.865 | 46.607 | 60.926 | 1.00 | 1.27 |
| ATOM | 2124 | C | GLY | A | 149 | 57.088 | 47.285 | 59.973 | 1.00 | 1.27 |
| ATOM | 2125 | O | GLY | A | 149 | 56.418 | 46.767 | 60.861 | 1.00 | 1.27 |
| ATOM | 2126 | N | ASP | A | 150 | 56.562 | 47.772 | 58.848 | 1.00 | 1.29 |
| ATOM | 2127 | H | ASP | A | 150 | 57.168 | 48.148 | 58.125 | 1.00 | 1.29 |
| ATOM | 2128 | CA | ASP | A | 150 | 55.118 | 47.841 | 58.612 | 1.00 | 1.29 |
| ATOM | 2129 | HA | ASP | A | 150 | 54.664 | 48.432 | 59.410 | 1.00 | 1.29 |
| ATOM | 2130 | CB | ASP | A | 150 | 54.855 | 48.572 | 57.282 | 1.00 | 1.29 |
| ATOM | 2131 | HB1 | ASP | A | 150 | 53.790 | 48.510 | 57.049 | 1.00 | 1.29 |
| ATOM | 2132 | HB2 | ASP | A | 150 | 55.397 | 48.077 | 56.475 | 1.00 | 1.29 |
| ATOM | 2133 | CG | ASP | A | 150 | 55.241 | 50.051 | 57.350 | 1.00 | 1.29 |
| ATOM | 2134 | OD1 | ASP | A | 150 | 56.314 | 50.428 | 56.824 | 1.00 | 1.29 |
| ATOM | 2135 | OD2 | ASP | A | 150 | 54.491 | 50.814 | 57.987 | 1.00 | 1.29 |
| ATOM | 2136 | C | ASP | A | 150 | 54.404 | 46.482 | 58.619 | 1.00 | 1.29 |
| ATOM | 2137 | O | ASP | A | 150 | 53.209 | 46.430 | 58.911 | 1.00 | 1.29 |
| ATOM | 2138 | N | ASP | A | 151 | 55.117 | 45.385 | 58.346 | 1.00 | 1.51 |
| ATOM | 2139 | H | ASP | A | 151 | 56.095 | 45.490 | 58.113 | 1.00 | 1.51 |
| ATOM | 2140 | CA | ASP | A | 151 | 54.583 | 44.026 | 58.518 | 1.00 | 1.51 |
| ATOM | 2141 | HA | ASP | A | 151 | 53.493 | 44.028 | 58.468 | 1.00 | 1.51 |
| ATOM | 2142 | CB | ASP | A | 151 | 55.113 | 43.170 | 57.356 | 1.00 | 1.51 |
| ATOM | 2143 | HB1 | ASP | A | 151 | 56.205 | 43.171 | 57.379 | 1.00 | 1.51 |
| ATOM | 2144 | HB2 | ASP | A | 151 | 54.804 | 43.627 | 56.413 | 1.00 | 1.51 |
| ATOM | 2145 | CG | ASP | A | 151 | 54.611 | 41.723 | 57.384 | 1.00 | 1.51 |
| ATOM | 2146 | OD1 | ASP | A | 151 | 55.404 | 40.853 | 57.813 | 1.00 | 1.51 |
| ATOM | 2147 | OD2 | ASP | A | 151 | 53.474 | 41.490 | 56.918 | 1.00 | 1.51 |
| ATOM | 2148 | C | ASP | A | 151 | 54.996 | 43.441 | 59.881 | 1.00 | 1.51 |
| ATOM | 2149 | O | ASP | A | 151 | 54.221 | 42.749 | 60.544 | 1.00 | 1.51 |
| ATOM | 2150 | N | ALA | A | 152 | 56.200 | 43.777 | 60.352 | 1.00 | 1.43 |
| ATOM | 2151 | H | ALA | A | 152 | 56.755 | 44.424 | 59.807 | 1.00 | 1.43 |
| ATOM | 2152 | CA | ALA | A | 152 | 56.874 | 42.994 | 61.383 | 1.00 | 1.43 |

| | | | | | | | | | | |
|---|---|---|---|---|---|---|---|---|---|---|
| ATOM | 2153 | HA | ALA | A | 152 | 56.352 | 42.043 | 61.497 | 1.00 | 1.43 |
| ATOM | 2154 | CB | ALA | A | 152 | 58.279 | 42.677 | 60.851 | 1.00 | 1.43 |
| ATOM | 2155 | HB1 | ALA | A | 152 | 58.826 | 42.070 | 61.573 | 1.00 | 1.43 |
| ATOM | 2156 | HB2 | ALA | A | 152 | 58.193 | 42.124 | 59.914 | 1.00 | 1.43 |
| ATOM | 2157 | HB3 | ALA | A | 152 | 58.818 | 43.605 | 60.665 | 1.00 | 1.43 |
| ATOM | 2158 | C | ALA | A | 152 | 56.875 | 43.653 | 62.779 | 1.00 | 1.43 |
| ATOM | 2159 | O | ALA | A | 152 | 57.743 | 43.366 | 63.601 | 1.00 | 1.43 |
| ATOM | 2160 | N | GLY | A | 153 | 55.921 | 44.546 | 63.061 | 1.00 | 1.30 |
| ATOM | 2161 | H | GLY | A | 153 | 55.283 | 44.796 | 62.315 | 1.00 | 1.30 |
| ATOM | 2162 | CA | GLY | A | 153 | 55.736 | 45.121 | 64.401 | 1.00 | 1.30 |
| ATOM | 2163 | HA1 | GLY | A | 153 | 55.882 | 44.343 | 65.152 | 1.00 | 1.30 |
| ATOM | 2164 | HA2 | GLY | A | 153 | 54.713 | 45.487 | 64.476 | 1.00 | 1.30 |
| ATOM | 2165 | C | GLY | A | 153 | 56.673 | 46.282 | 64.730 | 1.00 | 1.30 |
| ATOM | 2166 | O | GLY | A | 153 | 56.980 | 46.522 | 65.899 | 1.00 | 1.30 |
| ATOM | 2167 | N | GLY | A | 154 | 57.144 | 47.005 | 63.719 | 1.00 | 1.01 |
| ATOM | 2168 | H | GLY | A | 154 | 56.822 | 46.781 | 62.782 | 1.00 | 1.01 |
| ATOM | 2169 | CA | GLY | A | 154 | 57.872 | 48.248 | 63.918 | 1.00 | 1.01 |
| ATOM | 2170 | HA1 | GLY | A | 154 | 58.280 | 48.586 | 62.966 | 1.00 | 1.01 |
| ATOM | 2171 | HA2 | GLY | A | 154 | 58.702 | 48.076 | 64.603 | 1.00 | 1.01 |
| ATOM | 2172 | C | GLY | A | 154 | 56.967 | 49.349 | 64.474 | 1.00 | 1.01 |
| ATOM | 2173 | O | GLY | A | 154 | 55.875 | 49.594 | 63.970 | 1.00 | 1.01 |
| ATOM | 2174 | N | HIS | A | 155 | 57.429 | 50.023 | 65.526 | 1.00 | 0.88 |
| ATOM | 2175 | H | HIS | A | 155 | 58.319 | 49.758 | 65.921 | 1.00 | 0.88 |
| ATOM | 2176 | CA | HIS | A | 155 | 56.724 | 51.152 | 66.137 | 1.00 | 0.88 |
| ATOM | 2177 | HA | HIS | A | 155 | 56.227 | 51.730 | 65.354 | 1.00 | 0.88 |
| ATOM | 2178 | CB | HIS | A | 155 | 55.647 | 50.615 | 67.100 | 1.00 | 0.88 |
| ATOM | 2179 | HB1 | HIS | A | 155 | 56.126 | 50.036 | 67.891 | 1.00 | 0.88 |
| ATOM | 2180 | HB2 | HIS | A | 155 | 54.978 | 49.947 | 66.556 | 1.00 | 0.88 |
| ATOM | 2181 | CG | HIS | A | 155 | 54.803 | 51.696 | 67.728 | 1.00 | 0.88 |
| ATOM | 2182 | ND1 | HIS | A | 155 | 54.535 | 52.943 | 67.164 | 1.00 | 0.88 |
| ATOM | 2183 | CE1 | HIS | A | 155 | 53.871 | 53.645 | 68.090 | 1.00 | 0.88 |
| ATOM | 2184 | HE1 | HIS | A | 155 | 53.562 | 54.677 | 67.973 | 1.00 | 0.88 |
| ATOM | 2185 | NE2 | HIS | A | 155 | 53.680 | 52.891 | 69.187 | 1.00 | 0.88 |
| ATOM | 2186 | HE2 | HIS | A | 155 | 53.239 | 53.198 | 70.045 | 1.00 | 0.88 |
| ATOM | 2187 | CD2 | HIS | A | 155 | 54.267 | 51.660 | 68.978 | 1.00 | 0.88 |
| ATOM | 2188 | HD2 | HIS | A | 155 | 54.325 | 50.831 | 69.670 | 1.00 | 0.88 |
| ATOM | 2189 | C | HIS | A | 155 | 57.722 | 52.078 | 66.852 | 1.00 | 0.88 |
| ATOM | 2190 | O | HIS | A | 155 | 58.781 | 51.622 | 67.283 | 1.00 | 0.88 |
| ATOM | 2191 | N | ASN | A | 156 | 57.379 | 53.362 | 67.007 | 1.00 | 0.74 |
| ATOM | 2192 | H | ASN | A | 156 | 56.430 | 53.619 | 66.743 | 1.00 | 0.74 |
| ATOM | 2193 | CA | ASN | A | 156 | 58.290 | 54.465 | 67.332 | 1.00 | 0.74 |
| ATOM | 2194 | HA | ASN | A | 156 | 57.824 | 55.394 | 67.004 | 1.00 | 0.74 |
| ATOM | 2195 | CB | ASN | A | 156 | 58.541 | 54.602 | 68.837 | 1.00 | 0.74 |
| ATOM | 2196 | HB1 | ASN | A | 156 | 59.138 | 55.498 | 69.004 | 1.00 | 0.74 |

| | | | | | | | | | | |
|---|---|---|---|---|---|---|---|---|---|---|
| ATOM | 2197 | HB2 | ASN | A | 156 | 59.114 | 53.740 | 69.174 | 1.00 | 0.74 |
| ATOM | 2198 | CG | ASN | A | 156 | 57.318 | 54.723 | 69.704 | 1.00 | 0.74 |
| ATOM | 2199 | OD1 | ASN | A | 156 | 57.192 | 53.955 | 70.644 | 1.00 | 0.74 |
| ATOM | 2200 | ND2 | ASN | A | 156 | 56.441 | 55.676 | 69.498 | 1.00 | 0.74 |
| ATOM | 2201 | 1HD2 | ASN | A | 156 | 56.460 | 56.227 | 68.630 | 1.00 | 0.74 |
| ATOM | 2202 | 2HD2 | ASN | A | 156 | 55.692 | 55.797 | 70.151 | 1.00 | 0.74 |
| ATOM | 2203 | C | ASN | A | 156 | 59.628 | 54.385 | 66.577 | 1.00 | 0.74 |
| ATOM | 2204 | O | ASN | A | 156 | 60.718 | 54.382 | 67.160 | 1.00 | 0.74 |
| ATOM | 2205 | N | THR | A | 157 | 59.560 | 54.256 | 65.258 | 1.00 | 0.76 |
| ATOM | 2206 | H | THR | A | 157 | 58.656 | 54.246 | 64.806 | 1.00 | 0.76 |
| ATOM | 2207 | CA | THR | A | 157 | 60.745 | 54.245 | 64.395 | 1.00 | 0.76 |
| ATOM | 2208 | HA | THR | A | 157 | 61.637 | 54.040 | 64.982 | 1.00 | 0.76 |
| ATOM | 2209 | CB | THR | A | 157 | 60.650 | 53.125 | 63.331 | 1.00 | 0.76 |
| ATOM | 2210 | HB | THR | A | 157 | 61.449 | 53.239 | 62.597 | 1.00 | 0.76 |
| ATOM | 2211 | CG2 | THR | A | 157 | 60.770 | 51.732 | 63.946 | 1.00 | 0.76 |
| ATOM | 2212 | 1HG2 | THR | A | 157 | 60.654 | 50.972 | 63.173 | 1.00 | 0.76 |
| ATOM | 2213 | 2HG2 | THR | A | 157 | 61.755 | 51.615 | 64.396 | 1.00 | 0.76 |
| ATOM | 2214 | 3HG2 | THR | A | 157 | 60.005 | 51.586 | 64.707 | 1.00 | 0.76 |
| ATOM | 2215 | OG1 | THR | A | 157 | 59.402 | 53.163 | 62.677 | 1.00 | 0.76 |
| ATOM | 2216 | HG1 | THR | A | 157 | 59.307 | 52.338 | 62.153 | 1.00 | 0.76 |
| ATOM | 2217 | C | THR | A | 157 | 60.965 | 55.625 | 63.767 | 1.00 | 0.76 |
| ATOM | 2218 | O | THR | A | 157 | 61.299 | 55.736 | 62.589 | 1.00 | 0.76 |
| ATOM | 2219 | N | ASP | A | 158 | 60.758 | 56.687 | 64.552 | 1.00 | 0.70 |
| ATOM | 2220 | H | ASP | A | 158 | 60.390 | 56.544 | 65.489 | 1.00 | 0.70 |
| ATOM | 2221 | CA | ASP | A | 158 | 60.987 | 58.090 | 64.184 | 1.00 | 0.70 |
| ATOM | 2222 | HA | ASP | A | 158 | 60.148 | 58.407 | 63.571 | 1.00 | 0.70 |
| ATOM | 2223 | CB | ASP | A | 158 | 61.003 | 58.975 | 65.447 | 1.00 | 0.70 |
| ATOM | 2224 | HB1 | ASP | A | 158 | 61.115 | 60.016 | 65.136 | 1.00 | 0.70 |
| ATOM | 2225 | HB2 | ASP | A | 158 | 61.875 | 58.716 | 66.051 | 1.00 | 0.70 |
| ATOM | 2226 | CG | ASP | A | 158 | 59.754 | 58.848 | 66.329 | 1.00 | 0.70 |
| ATOM | 2227 | OD1 | ASP | A | 158 | 59.609 | 57.760 | 66.944 | 1.00 | 0.70 |
| ATOM | 2228 | OD2 | ASP | A | 158 | 59.002 | 59.835 | 66.430 | 1.00 | 0.70 |
| ATOM | 2229 | C | ASP | A | 158 | 62.294 | 58.293 | 63.401 | 1.00 | 0.70 |
| ATOM | 2230 | O | ASP | A | 158 | 63.355 | 57.818 | 63.814 | 1.00 | 0.70 |
| ATOM | 2231 | N | GLY | A | 159 | 62.223 | 59.008 | 62.277 | 1.00 | 0.79 |
| ATOM | 2232 | H | GLY | A | 159 | 61.330 | 59.427 | 62.032 | 1.00 | 0.79 |
| ATOM | 2233 | CA | GLY | A | 159 | 63.373 | 59.195 | 61.389 | 1.00 | 0.79 |
| ATOM | 2234 | HA1 | GLY | A | 159 | 63.018 | 59.551 | 60.421 | 1.00 | 0.79 |
| ATOM | 2235 | HA2 | GLY | A | 159 | 63.888 | 58.246 | 61.244 | 1.00 | 0.79 |
| ATOM | 2236 | C | GLY | A | 159 | 64.364 | 60.220 | 61.936 | 1.00 | 0.79 |
| ATOM | 2237 | O | GLY | A | 159 | 65.520 | 59.893 | 62.212 | 1.00 | 0.79 |
| ATOM | 2238 | N | PHE | A | 160 | 63.881 | 61.441 | 62.169 | 1.00 | 0.48 |
| ATOM | 2239 | H | PHE | A | 160 | 62.925 | 61.646 | 61.915 | 1.00 | 0.48 |
| ATOM | 2240 | CA | PHE | A | 160 | 64.670 | 62.500 | 62.800 | 1.00 | 0.48 |

| | | | | | | | | | | |
|---|---|---|---|---|---|---|---|---|---|---|
| ATOM | 2241 | HA | PHE | A | 160 | 65.636 | 62.108 | 63.120 | 1.00 | 0.48 |
| ATOM | 2242 | CB | PHE | A | 160 | 64.924 | 63.624 | 61.780 | 1.00 | 0.48 |
| ATOM | 2243 | HB1 | PHE | A | 160 | 65.538 | 64.389 | 62.257 | 1.00 | 0.48 |
| ATOM | 2244 | HB2 | PHE | A | 160 | 63.972 | 64.090 | 61.522 | 1.00 | 0.48 |
| ATOM | 2245 | CG | PHE | A | 160 | 65.612 | 63.184 | 60.502 | 1.00 | 0.48 |
| ATOM | 2246 | CD1 | PHE | A | 160 | 66.998 | 62.945 | 60.501 | 1.00 | 0.48 |
| ATOM | 2247 | HD1 | PHE | A | 160 | 67.563 | 63.065 | 61.412 | 1.00 | 0.48 |
| ATOM | 2248 | CE1 | PHE | A | 160 | 67.639 | 62.529 | 59.323 | 1.00 | 0.48 |
| ATOM | 2249 | HE1 | PHE | A | 160 | 68.701 | 62.330 | 59.328 | 1.00 | 0.48 |
| ATOM | 2250 | CZ | PHE | A | 160 | 66.896 | 62.363 | 58.141 | 1.00 | 0.48 |
| ATOM | 2251 | HZ | PHE | A | 160 | 67.387 | 62.045 | 57.239 | 1.00 | 0.48 |
| ATOM | 2252 | CE2 | PHE | A | 160 | 65.511 | 62.589 | 58.136 | 1.00 | 0.48 |
| ATOM | 2253 | HE2 | PHE | A | 160 | 64.935 | 62.427 | 57.236 | 1.00 | 0.48 |
| ATOM | 2254 | CD2 | PHE | A | 160 | 64.871 | 62.998 | 59.318 | 1.00 | 0.48 |
| ATOM | 2255 | HD2 | PHE | A | 160 | 63.801 | 63.151 | 59.320 | 1.00 | 0.48 |
| ATOM | 2256 | C | PHE | A | 160 | 63.951 | 63.055 | 64.030 | 1.00 | 0.48 |
| ATOM | 2257 | O | PHE | A | 160 | 62.855 | 63.605 | 63.924 | 1.00 | 0.48 |
| ATOM | 2258 | N | ASP | A | 161 | 64.607 | 62.971 | 65.183 | 1.00 | 0.56 |
| ATOM | 2259 | H | ASP | A | 161 | 65.548 | 62.582 | 65.188 | 1.00 | 0.56 |
| ATOM | 2260 | CA | ASP | A | 161 | 64.145 | 63.616 | 66.412 | 1.00 | 0.56 |
| ATOM | 2261 | HA | ASP | A | 161 | 63.098 | 63.920 | 66.360 | 1.00 | 0.56 |
| ATOM | 2262 | CB | ASP | A | 161 | 64.333 | 62.605 | 67.566 | 1.00 | 0.56 |
| ATOM | 2263 | HB1 | ASP | A | 161 | 65.144 | 61.917 | 67.318 | 1.00 | 0.56 |
| ATOM | 2264 | HB2 | ASP | A | 161 | 63.425 | 62.005 | 67.652 | 1.00 | 0.56 |
| ATOM | 2265 | CG | ASP | A | 161 | 64.651 | 63.241 | 68.924 | 1.00 | 0.56 |
| ATOM | 2266 | OD1 | ASP | A | 161 | 63.722 | 63.839 | 69.514 | 1.00 | 0.56 |
| ATOM | 2267 | OD2 | ASP | A | 161 | 65.816 | 63.110 | 69.372 | 1.00 | 0.56 |
| ATOM | 2268 | C | ASP | A | 161 | 65.035 | 64.847 | 66.561 | 1.00 | 0.56 |
| ATOM | 2269 | O | ASP | A | 161 | 66.241 | 64.766 | 66.331 | 1.00 | 0.56 |
| ATOM | 2270 | N | ILE | A | 162 | 64.465 | 66.010 | 66.856 | 1.00 | 0.77 |
| ATOM | 2271 | H | ILE | A | 162 | 63.461 | 66.051 | 67.010 | 1.00 | 0.77 |
| ATOM | 2272 | CA | ILE | A | 162 | 65.263 | 67.239 | 66.876 | 1.00 | 0.77 |
| ATOM | 2273 | HA | ILE | A | 162 | 66.325 | 66.997 | 66.924 | 1.00 | 0.77 |
| ATOM | 2274 | CB | ILE | A | 162 | 65.045 | 68.095 | 65.603 | 1.00 | 0.77 |
| ATOM | 2275 | HB | ILE | A | 162 | 64.055 | 68.547 | 65.653 | 1.00 | 0.77 |
| ATOM | 2276 | CG2 | ILE | A | 162 | 66.084 | 69.232 | 65.563 | 1.00 | 0.77 |
| ATOM | 2277 | 1HG2 | ILE | A | 162 | 65.859 | 69.919 | 64.750 | 1.00 | 0.77 |
| ATOM | 2278 | 2HG2 | ILE | A | 162 | 66.061 | 69.806 | 66.491 | 1.00 | 0.77 |
| ATOM | 2279 | 3HG2 | ILE | A | 162 | 67.088 | 68.826 | 65.429 | 1.00 | 0.77 |
| ATOM | 2280 | CG1 | ILE | A | 162 | 65.103 | 67.242 | 64.313 | 1.00 | 0.77 |
| ATOM | 2281 | 1HG1 | ILE | A | 162 | 64.268 | 66.539 | 64.320 | 1.00 | 0.77 |
| ATOM | 2282 | 2HG1 | ILE | A | 162 | 66.028 | 66.667 | 64.305 | 1.00 | 0.77 |
| ATOM | 2283 | CD1 | ILE | A | 162 | 65.008 | 68.028 | 63.001 | 1.00 | 0.77 |
| ATOM | 2284 | HD1 | ILE | A | 162 | 64.818 | 67.328 | 62.190 | 1.00 | 0.77 |

| | | | | | | | | | | |
|---|---|---|---|---|---|---|---|---|---|---|
| ATOM | 2285 | HD2 | ILE | A | 162 | 64.187 | 68.738 | 63.044 | 1.00 | 0.77 |
| ATOM | 2286 | HD3 | ILE | A | 162 | 65.942 | 68.556 | 62.807 | 1.00 | 0.77 |
| ATOM | 2287 | C | ILE | A | 162 | 64.912 | 68.010 | 68.136 | 1.00 | 0.77 |
| ATOM | 2288 | O | ILE | A | 162 | 63.760 | 68.382 | 68.377 | 1.00 | 0.77 |
| ATOM | 2289 | N | GLY | A | 163 | 65.925 | 68.221 | 68.957 | 1.00 | 0.88 |
| ATOM | 2290 | H | GLY | A | 163 | 66.845 | 67.870 | 68.699 | 1.00 | 0.88 |
| ATOM | 2291 | CA | GLY | A | 163 | 65.843 | 68.978 | 70.185 | 1.00 | 0.88 |
| ATOM | 2292 | HA1 | GLY | A | 163 | 65.777 | 68.291 | 71.028 | 1.00 | 0.88 |
| ATOM | 2293 | HA2 | GLY | A | 163 | 64.962 | 69.613 | 70.185 | 1.00 | 0.88 |
| ATOM | 2294 | C | GLY | A | 163 | 67.075 | 69.839 | 70.366 | 1.00 | 0.88 |
| ATOM | 2295 | O | GLY | A | 163 | 68.096 | 69.612 | 69.718 | 1.00 | 0.88 |
| ATOM | 2296 | N | GLU | A | 164 | 66.952 | 70.826 | 71.252 | 1.00 | 1.02 |
| ATOM | 2297 | H | GLU | A | 164 | 66.085 | 70.874 | 71.769 | 1.00 | 1.02 |
| ATOM | 2298 | CA | GLU | A | 164 | 68.045 | 71.650 | 71.782 | 1.00 | 1.02 |
| ATOM | 2299 | HA | GLU | A | 164 | 67.614 | 72.599 | 72.109 | 1.00 | 1.02 |
| ATOM | 2300 | CB | GLU | A | 164 | 68.654 | 70.961 | 73.031 | 1.00 | 1.02 |
| ATOM | 2301 | HB1 | GLU | A | 164 | 69.263 | 71.703 | 73.550 | 1.00 | 1.02 |
| ATOM | 2302 | HB2 | GLU | A | 164 | 69.323 | 70.161 | 72.710 | 1.00 | 1.02 |
| ATOM | 2303 | CG | GLU | A | 164 | 67.669 | 70.372 | 74.055 | 1.00 | 1.02 |
| ATOM | 2304 | HG1 | GLU | A | 164 | 66.871 | 71.094 | 74.243 | 1.00 | 1.02 |
| ATOM | 2305 | HG2 | GLU | A | 164 | 68.205 | 70.216 | 74.994 | 1.00 | 1.02 |
| ATOM | 2306 | CD | GLU | A | 164 | 67.082 | 69.028 | 73.603 | 1.00 | 1.02 |
| ATOM | 2307 | OE1 | GLU | A | 164 | 67.846 | 68.078 | 73.329 | 1.00 | 1.02 |
| ATOM | 2308 | OE2 | GLU | A | 164 | 65.839 | 68.927 | 73.485 | 1.00 | 1.02 |
| ATOM | 2309 | C | GLU | A | 164 | 69.153 | 71.990 | 70.770 | 1.00 | 1.02 |
| ATOM | 2310 | O | GLU | A | 164 | 70.340 | 71.870 | 71.070 | 1.00 | 1.02 |
| ATOM | 2311 | N | SER | A | 165 | 68.780 | 72.306 | 69.531 | 1.00 | 0.93 |
| ATOM | 2312 | H | SER | A | 165 | 67.795 | 72.342 | 69.318 | 1.00 | 0.93 |
| ATOM | 2313 | CA | SER | A | 165 | 69.725 | 72.465 | 68.430 | 1.00 | 0.93 |
| ATOM | 2314 | HA | SER | A | 165 | 70.691 | 72.732 | 68.857 | 1.00 | 0.93 |
| ATOM | 2315 | CB | SER | A | 165 | 69.955 | 71.165 | 67.642 | 1.00 | 0.93 |
| ATOM | 2316 | HB1 | SER | A | 165 | 70.565 | 70.500 | 68.253 | 1.00 | 0.93 |
| ATOM | 2317 | HB2 | SER | A | 165 | 70.508 | 71.386 | 66.729 | 1.00 | 0.93 |
| ATOM | 2318 | OG | SER | A | 165 | 68.769 | 70.475 | 67.319 | 1.00 | 0.93 |
| ATOM | 2319 | HG | SER | A | 165 | 68.448 | 70.079 | 68.157 | 1.00 | 0.93 |
| ATOM | 2320 | C | SER | A | 165 | 69.351 | 73.638 | 67.546 | 1.00 | 0.93 |
| ATOM | 2321 | O | SER | A | 165 | 68.187 | 74.038 | 67.460 | 1.00 | 0.93 |
| ATOM | 2322 | N | SER | A | 166 | 70.370 | 74.194 | 66.908 | 1.00 | 0.82 |
| ATOM | 2323 | H | SER | A | 166 | 71.299 | 73.814 | 67.057 | 1.00 | 0.82 |
| ATOM | 2324 | CA | SER | A | 166 | 70.267 | 75.379 | 66.077 | 1.00 | 0.82 |
| ATOM | 2325 | HA | SER | A | 166 | 69.224 | 75.657 | 65.978 | 1.00 | 0.82 |
| ATOM | 2326 | CB | SER | A | 166 | 71.011 | 76.596 | 66.663 | 1.00 | 0.82 |
| ATOM | 2327 | HB1 | SER | A | 166 | 70.423 | 77.495 | 66.485 | 1.00 | 0.82 |
| ATOM | 2328 | HB2 | SER | A | 166 | 71.939 | 76.730 | 66.117 | 1.00 | 0.82 |

| | | | | | | | | | | |
|---|---|---|---|---|---|---|---|---|---|---|
| ATOM | 2329 | OG | SER | A | 166 | 71.324 | 76.506 | 68.041 | 1.00 | 0.82 |
| ATOM | 2330 | HG | SER | A | 166 | 72.312 | 76.584 | 68.087 | 1.00 | 0.82 |
| ATOM | 2331 | C | SER | A | 166 | 70.836 | 75.084 | 64.699 | 1.00 | 0.82 |
| ATOM | 2332 | O | SER | A | 166 | 71.808 | 74.334 | 64.559 | 1.00 | 0.82 |
| ATOM | 2333 | N | ASN | A | 167 | 70.275 | 75.724 | 63.676 | 1.00 | 0.41 |
| ATOM | 2334 | H | ASN | A | 167 | 69.400 | 76.212 | 63.836 | 1.00 | 0.41 |
| ATOM | 2335 | CA | ASN | A | 167 | 70.827 | 75.670 | 62.325 | 1.00 | 0.41 |
| ATOM | 2336 | HA | ASN | A | 167 | 70.124 | 76.165 | 61.654 | 1.00 | 0.41 |
| ATOM | 2337 | CB | ASN | A | 167 | 72.150 | 76.476 | 62.285 | 1.00 | 0.41 |
| ATOM | 2338 | HB1 | ASN | A | 167 | 72.509 | 76.522 | 61.259 | 1.00 | 0.41 |
| ATOM | 2339 | HB2 | ASN | A | 167 | 72.907 | 75.970 | 62.877 | 1.00 | 0.41 |
| ATOM | 2340 | CG | ASN | A | 167 | 72.025 | 77.898 | 62.796 | 1.00 | 0.41 |
| ATOM | 2341 | OD1 | ASN | A | 167 | 71.277 | 78.702 | 62.257 | 1.00 | 0.41 |
| ATOM | 2342 | ND2 | ASN | A | 167 | 72.759 | 78.273 | 63.822 | 1.00 | 0.41 |
| ATOM | 2343 | 1HD2 | ASN | A | 167 | 73.359 | 77.616 | 64.342 | 1.00 | 0.41 |
| ATOM | 2344 | 2HD2 | ASN | A | 167 | 72.675 | 79.219 | 64.130 | 1.00 | 0.41 |
| ATOM | 2345 | C | ASN | A | 167 | 70.983 | 74.220 | 61.823 | 1.00 | 0.41 |
| ATOM | 2346 | O | ASN | A | 167 | 72.082 | 73.764 | 61.493 | 1.00 | 0.41 |
| ATOM | 2347 | N | VAL | A | 168 | 69.881 | 73.465 | 61.813 | 1.00 | 0.41 |
| ATOM | 2348 | H | VAL | A | 168 | 69.001 | 73.920 | 62.023 | 1.00 | 0.41 |
| ATOM | 2349 | CA | VAL | A | 168 | 69.884 | 72.034 | 61.430 | 1.00 | 0.41 |
| ATOM | 2350 | HA | VAL | A | 168 | 70.907 | 71.696 | 61.285 | 1.00 | 0.41 |
| ATOM | 2351 | CB | VAL | A | 168 | 69.247 | 71.153 | 62.528 | 1.00 | 0.41 |
| ATOM | 2352 | HB | VAL | A | 168 | 68.197 | 71.419 | 62.620 | 1.00 | 0.41 |
| ATOM | 2353 | CG1 | VAL | A | 168 | 69.323 | 69.655 | 62.188 | 1.00 | 0.41 |
| ATOM | 2354 | 1HG1 | VAL | A | 168 | 68.866 | 69.077 | 62.992 | 1.00 | 0.41 |
| ATOM | 2355 | 2HG1 | VAL | A | 168 | 68.770 | 69.442 | 61.274 | 1.00 | 0.41 |
| ATOM | 2356 | 3HG1 | VAL | A | 168 | 70.363 | 69.350 | 62.071 | 1.00 | 0.41 |
| ATOM | 2357 | CG2 | VAL | A | 168 | 69.919 | 71.343 | 63.894 | 1.00 | 0.41 |
| ATOM | 2358 | 1HG2 | VAL | A | 168 | 69.480 | 70.663 | 64.625 | 1.00 | 0.41 |
| ATOM | 2359 | 2HG2 | VAL | A | 168 | 70.991 | 71.159 | 63.819 | 1.00 | 0.41 |
| ATOM | 2360 | 3HG2 | VAL | A | 168 | 69.762 | 72.361 | 64.250 | 1.00 | 0.41 |
| ATOM | 2361 | C | VAL | A | 168 | 69.127 | 71.867 | 60.115 | 1.00 | 0.41 |
| ATOM | 2362 | O | VAL | A | 168 | 68.097 | 72.503 | 59.917 | 1.00 | 0.41 |
| ATOM | 2363 | N | VAL | A | 169 | 69.635 | 71.074 | 59.166 | 1.00 | 0.42 |
| ATOM | 2364 | H | VAL | A | 169 | 70.275 | 70.320 | 59.409 | 1.00 | 0.42 |
| ATOM | 2365 | CA | VAL | A | 169 | 69.462 | 71.372 | 57.734 | 1.00 | 0.42 |
| ATOM | 2366 | HA | VAL | A | 169 | 68.514 | 71.885 | 57.574 | 1.00 | 0.42 |
| ATOM | 2367 | CB | VAL | A | 169 | 70.601 | 72.271 | 57.200 | 1.00 | 0.42 |
| ATOM | 2368 | HB | VAL | A | 169 | 71.542 | 71.735 | 57.299 | 1.00 | 0.42 |
| ATOM | 2369 | CG1 | VAL | A | 169 | 70.383 | 72.628 | 55.723 | 1.00 | 0.42 |
| ATOM | 2370 | 1HG1 | VAL | A | 169 | 71.200 | 73.258 | 55.371 | 1.00 | 0.42 |
| ATOM | 2371 | 2HG1 | VAL | A | 169 | 70.360 | 71.728 | 55.111 | 1.00 | 0.42 |
| ATOM | 2372 | 3HG1 | VAL | A | 169 | 69.443 | 73.168 | 55.605 | 1.00 | 0.42 |

| | | | | | | | | | | |
|---|---|---|---|---|---|---|---|---|---|---|
| ATOM | 2373 | CG2 | VAL | A | 169 | 70.745 | 73.590 | 57.971 | 1.00 | 0.42 |
| ATOM | 2374 | 1HG2 | VAL | A | 169 | 71.484 | 74.230 | 57.489 | 1.00 | 0.42 |
| ATOM | 2375 | 2HG2 | VAL | A | 169 | 69.791 | 74.114 | 58.013 | 1.00 | 0.42 |
| ATOM | 2376 | 3HG2 | VAL | A | 169 | 71.093 | 73.395 | 58.984 | 1.00 | 0.42 |
| ATOM | 2377 | C | VAL | A | 169 | 69.399 | 70.020 | 57.024 | 1.00 | 0.42 |
| ATOM | 2378 | O | VAL | A | 169 | 70.391 | 69.436 | 56.561 | 1.00 | 0.42 |
| ATOM | 2379 | N | ILE | A | 170 | 68.200 | 69.456 | 57.060 | 1.00 | 0.47 |
| ATOM | 2380 | H | ILE | A | 170 | 67.437 | 70.004 | 57.451 | 1.00 | 0.47 |
| ATOM | 2381 | CA | ILE | A | 170 | 67.870 | 68.152 | 56.512 | 1.00 | 0.47 |
| ATOM | 2382 | HA | ILE | A | 170 | 68.768 | 67.538 | 56.473 | 1.00 | 0.47 |
| ATOM | 2383 | CB | ILE | A | 170 | 66.831 | 67.445 | 57.420 | 1.00 | 0.47 |
| ATOM | 2384 | HB | ILE | A | 170 | 65.956 | 68.092 | 57.497 | 1.00 | 0.47 |
| ATOM | 2385 | CG2 | ILE | A | 170 | 66.389 | 66.099 | 56.807 | 1.00 | 0.47 |
| ATOM | 2386 | 1HG2 | ILE | A | 170 | 65.784 | 65.531 | 57.512 | 1.00 | 0.47 |
| ATOM | 2387 | 2HG2 | ILE | A | 170 | 65.780 | 66.268 | 55.919 | 1.00 | 0.47 |
| ATOM | 2388 | 3HG2 | ILE | A | 170 | 67.250 | 65.496 | 56.535 | 1.00 | 0.47 |
| ATOM | 2389 | CG1 | ILE | A | 170 | 67.386 | 67.207 | 58.845 | 1.00 | 0.47 |
| ATOM | 2390 | 1HG1 | ILE | A | 170 | 67.956 | 68.072 | 59.183 | 1.00 | 0.47 |
| ATOM | 2391 | 2HG1 | ILE | A | 170 | 68.053 | 66.349 | 58.839 | 1.00 | 0.47 |
| ATOM | 2392 | CD1 | ILE | A | 170 | 66.295 | 66.961 | 59.890 | 1.00 | 0.47 |
| ATOM | 2393 | HD1 | ILE | A | 170 | 65.621 | 67.817 | 59.925 | 1.00 | 0.47 |
| ATOM | 2394 | HD2 | ILE | A | 170 | 65.727 | 66.061 | 59.660 | 1.00 | 0.47 |
| ATOM | 2395 | HD3 | ILE | A | 170 | 66.766 | 66.834 | 60.865 | 1.00 | 0.47 |
| ATOM | 2396 | C | ILE | A | 170 | 67.343 | 68.310 | 55.085 | 1.00 | 0.47 |
| ATOM | 2397 | O | ILE | A | 170 | 66.748 | 69.319 | 54.712 | 1.00 | 0.47 |
| ATOM | 2398 | N | THR | A | 171 | 67.641 | 67.318 | 54.255 | 1.00 | 0.44 |
| ATOM | 2399 | H | THR | A | 171 | 67.917 | 66.410 | 54.624 | 1.00 | 0.44 |
| ATOM | 2400 | CA | THR | A | 171 | 67.864 | 67.515 | 52.824 | 1.00 | 0.44 |
| ATOM | 2401 | HA | THR | A | 171 | 67.070 | 68.118 | 52.397 | 1.00 | 0.44 |
| ATOM | 2402 | CB | THR | A | 171 | 69.215 | 68.212 | 52.545 | 1.00 | 0.44 |
| ATOM | 2403 | HB | THR | A | 171 | 69.656 | 67.778 | 51.650 | 1.00 | 0.44 |
| ATOM | 2404 | CG2 | THR | A | 171 | 69.057 | 69.712 | 52.310 | 1.00 | 0.44 |
| ATOM | 2405 | 1HG2 | THR | A | 171 | 70.021 | 70.139 | 52.033 | 1.00 | 0.44 |
| ATOM | 2406 | 2HG2 | THR | A | 171 | 68.356 | 69.886 | 51.493 | 1.00 | 0.44 |
| ATOM | 2407 | 3HG2 | THR | A | 171 | 68.689 | 70.212 | 53.205 | 1.00 | 0.44 |
| ATOM | 2408 | OG1 | THR | A | 171 | 70.113 | 68.098 | 53.631 | 1.00 | 0.44 |
| ATOM | 2409 | HG1 | THR | A | 171 | 69.977 | 68.872 | 54.187 | 1.00 | 0.44 |
| ATOM | 2410 | C | THR | A | 171 | 67.844 | 66.099 | 52.208 | 1.00 | 0.44 |
| ATOM | 2411 | O | THR | A | 171 | 68.452 | 65.122 | 52.703 | 1.00 | 0.44 |
| ATOM | 2412 | N | GLY | A | 172 | 67.065 | 65.973 | 51.136 | 1.00 | 0.66 |
| ATOM | 2413 | H | GLY | A | 172 | 66.486 | 66.766 | 50.879 | 1.00 | 0.66 |
| ATOM | 2414 | CA | GLY | A | 172 | 66.924 | 64.740 | 50.365 | 1.00 | 0.66 |
| ATOM | 2415 | HA1 | GLY | A | 172 | 67.910 | 64.332 | 50.141 | 1.00 | 0.66 |
| ATOM | 2416 | HA2 | GLY | A | 172 | 66.434 | 64.967 | 49.418 | 1.00 | 0.66 |

| | | | | | | | | | | |
|---|---|---|---|---|---|---|---|---|---|---|
| ATOM | 2417 | C | GLY | A | 172 | 66.095 | 63.655 | 51.084 | 1.00 | 0.66 |
| ATOM | 2418 | O | GLY | A | 172 | 64.928 | 63.482 | 50.759 | 1.00 | 0.66 |
| ATOM | 2419 | N | ALA | A | 173 | 66.645 | 62.960 | 52.087 | 1.00 | 0.87 |
| ATOM | 2420 | H | ALA | A | 173 | 67.643 | 63.019 | 52.192 | 1.00 | 0.87 |
| ATOM | 2421 | CA | ALA | A | 173 | 65.959 | 62.418 | 53.273 | 1.00 | 0.87 |
| ATOM | 2422 | HA | ALA | A | 173 | 66.681 | 62.498 | 54.086 | 1.00 | 0.87 |
| ATOM | 2423 | CB | ALA | A | 173 | 64.748 | 63.257 | 53.710 | 1.00 | 0.87 |
| ATOM | 2424 | HB1 | ALA | A | 173 | 64.486 | 63.005 | 54.737 | 1.00 | 0.87 |
| ATOM | 2425 | HB2 | ALA | A | 173 | 64.974 | 64.322 | 53.651 | 1.00 | 0.87 |
| ATOM | 2426 | HB3 | ALA | A | 173 | 63.897 | 63.009 | 53.080 | 1.00 | 0.87 |
| ATOM | 2427 | C | ALA | A | 173 | 65.597 | 60.907 | 53.215 | 1.00 | 0.87 |
| ATOM | 2428 | O | ALA | A | 173 | 65.959 | 60.123 | 54.110 | 1.00 | 0.87 |
| ATOM | 2429 | N | LYS | A | 174 | 64.889 | 60.536 | 52.138 | 1.00 | 0.42 |
| ATOM | 2430 | H | LYS | A | 174 | 64.764 | 61.265 | 51.443 | 1.00 | 0.42 |
| ATOM | 2431 | CA | LYS | A | 174 | 63.986 | 59.355 | 52.025 | 1.00 | 0.42 |
| ATOM | 2432 | HA | LYS | A | 174 | 63.394 | 59.560 | 51.142 | 1.00 | 0.42 |
| ATOM | 2433 | CB | LYS | A | 174 | 64.695 | 58.033 | 51.656 | 1.00 | 0.42 |
| ATOM | 2434 | HB1 | LYS | A | 174 | 63.953 | 57.390 | 51.180 | 1.00 | 0.42 |
| ATOM | 2435 | HB2 | LYS | A | 174 | 65.007 | 57.533 | 52.571 | 1.00 | 0.42 |
| ATOM | 2436 | CG | LYS | A | 174 | 65.928 | 58.068 | 50.746 | 1.00 | 0.42 |
| ATOM | 2437 | HG1 | LYS | A | 174 | 66.152 | 57.040 | 50.464 | 1.00 | 0.42 |
| ATOM | 2438 | HG2 | LYS | A | 174 | 66.744 | 58.417 | 51.358 | 1.00 | 0.42 |
| ATOM | 2439 | CD | LYS | A | 174 | 65.908 | 58.909 | 49.460 | 1.00 | 0.42 |
| ATOM | 2440 | HD1 | LYS | A | 174 | 65.760 | 59.964 | 49.689 | 1.00 | 0.42 |
| ATOM | 2441 | HD2 | LYS | A | 174 | 65.091 | 58.568 | 48.823 | 1.00 | 0.42 |
| ATOM | 2442 | CE | LYS | A | 174 | 67.249 | 58.721 | 48.724 | 1.00 | 0.42 |
| ATOM | 2443 | HE1 | LYS | A | 174 | 67.201 | 59.206 | 47.745 | 1.00 | 0.42 |
| ATOM | 2444 | HE2 | LYS | A | 174 | 67.403 | 57.650 | 48.565 | 1.00 | 0.42 |
| ATOM | 2445 | NZ | LYS | A | 174 | 68.397 | 59.272 | 49.486 | 1.00 | 0.42 |
| ATOM | 2446 | HZ1 | LYS | A | 174 | 68.508 | 60.279 | 49.345 | 1.00 | 0.42 |
| ATOM | 2447 | HZ2 | LYS | A | 174 | 68.340 | 59.072 | 50.481 | 1.00 | 0.42 |
| ATOM | 2448 | HZ3 | LYS | A | 174 | 69.297 | 58.930 | 49.158 | 1.00 | 0.42 |
| ATOM | 2449 | C | LYS | A | 174 | 62.898 | 59.210 | 53.120 | 1.00 | 0.42 |
| ATOM | 2450 | O | LYS | A | 174 | 62.111 | 60.155 | 53.281 | 1.00 | 0.42 |
| ATOM | 2451 | N | VAL | A | 175 | 62.824 | 58.041 | 53.799 | 1.00 | 0.43 |
| ATOM | 2452 | H | VAL | A | 175 | 63.460 | 57.316 | 53.498 | 1.00 | 0.43 |
| ATOM | 2453 | CA | VAL | A | 175 | 62.287 | 57.792 | 55.156 | 1.00 | 0.43 |
| ATOM | 2454 | HA | VAL | A | 175 | 63.218 | 57.721 | 55.715 | 1.00 | 0.43 |
| ATOM | 2455 | CB | VAL | A | 175 | 61.594 | 58.965 | 55.891 | 1.00 | 0.43 |
| ATOM | 2456 | HB | VAL | A | 175 | 60.898 | 59.460 | 55.227 | 1.00 | 0.43 |
| ATOM | 2457 | CG1 | VAL | A | 175 | 60.791 | 58.523 | 57.119 | 1.00 | 0.43 |
| ATOM | 2458 | 1HG1 | VAL | A | 175 | 60.406 | 59.388 | 57.651 | 1.00 | 0.43 |
| ATOM | 2459 | 2HG1 | VAL | A | 175 | 59.938 | 57.919 | 56.810 | 1.00 | 0.43 |
| ATOM | 2460 | 3HG1 | VAL | A | 175 | 61.416 | 57.945 | 57.800 | 1.00 | 0.43 |

| | | | | | | | | | | |
|---|---|---|---|---|---|---|---|---|---|---|
| ATOM | 2461 | CG2 | VAL | A | 175 | 62.646 | 59.970 | 56.399 | 1.00 | 0.43 |
| ATOM | 2462 | 1HG2 | VAL | A | 175 | 62.158 | 60.812 | 56.885 | 1.00 | 0.43 |
| ATOM | 2463 | 2HG2 | VAL | A | 175 | 63.305 | 59.489 | 57.123 | 1.00 | 0.43 |
| ATOM | 2464 | 3HG2 | VAL | A | 175 | 63.252 | 60.346 | 55.578 | 1.00 | 0.43 |
| ATOM | 2465 | C | VAL | A | 175 | 61.725 | 56.396 | 55.488 | 1.00 | 0.43 |
| ATOM | 2466 | O | VAL | A | 175 | 62.322 | 55.692 | 56.309 | 1.00 | 0.43 |
| ATOM | 2467 | N | TYR | A | 176 | 60.606 | 55.993 | 54.884 | 1.00 | 0.50 |
| ATOM | 2468 | H | TYR | A | 176 | 60.169 | 56.626 | 54.223 | 1.00 | 0.50 |
| ATOM | 2469 | CA | TYR | A | 176 | 59.941 | 54.699 | 55.144 | 1.00 | 0.50 |
| ATOM | 2470 | HA | TYR | A | 176 | 58.894 | 54.840 | 54.884 | 1.00 | 0.50 |
| ATOM | 2471 | CB | TYR | A | 176 | 60.463 | 53.626 | 54.171 | 1.00 | 0.50 |
| ATOM | 2472 | HB1 | TYR | A | 176 | 59.733 | 52.817 | 54.118 | 1.00 | 0.50 |
| ATOM | 2473 | HB2 | TYR | A | 176 | 61.373 | 53.200 | 54.579 | 1.00 | 0.50 |
| ATOM | 2474 | CG | TYR | A | 176 | 60.761 | 54.119 | 52.762 | 1.00 | 0.50 |
| ATOM | 2475 | CD1 | TYR | A | 176 | 59.719 | 54.310 | 51.833 | 1.00 | 0.50 |
| ATOM | 2476 | HD1 | TYR | A | 176 | 58.703 | 54.067 | 52.109 | 1.00 | 0.50 |
| ATOM | 2477 | CE1 | TYR | A | 176 | 60.004 | 54.808 | 50.545 | 1.00 | 0.50 |
| ATOM | 2478 | HE1 | TYR | A | 176 | 59.213 | 54.967 | 49.827 | 1.00 | 0.50 |
| ATOM | 2479 | CZ | TYR | A | 176 | 61.339 | 55.076 | 50.174 | 1.00 | 0.50 |
| ATOM | 2480 | OH | TYR | A | 176 | 61.626 | 55.510 | 48.920 | 1.00 | 0.50 |
| ATOM | 2481 | HH | TYR | A | 176 | 62.571 | 55.542 | 48.774 | 1.00 | 0.50 |
| ATOM | 2482 | CE2 | TYR | A | 176 | 62.384 | 54.872 | 51.098 | 1.00 | 0.50 |
| ATOM | 2483 | HE2 | TYR | A | 176 | 63.411 | 55.047 | 50.818 | 1.00 | 0.50 |
| ATOM | 2484 | CD2 | TYR | A | 176 | 62.089 | 54.406 | 52.392 | 1.00 | 0.50 |
| ATOM | 2485 | HD2 | TYR | A | 176 | 62.890 | 54.250 | 53.099 | 1.00 | 0.50 |
| ATOM | 2486 | C | TYR | A | 176 | 59.946 | 54.227 | 56.610 | 1.00 | 0.50 |
| ATOM | 2487 | O | TYR | A | 176 | 60.825 | 53.487 | 57.047 | 1.00 | 0.50 |
| ATOM | 2488 | N | ASN | A | 177 | 58.991 | 54.668 | 57.418 | 1.00 | 0.75 |
| ATOM | 2489 | H | ASN | A | 177 | 58.249 | 55.250 | 57.048 | 1.00 | 0.75 |
| ATOM | 2490 | CA | ASN | A | 177 | 58.957 | 54.306 | 58.837 | 1.00 | 0.75 |
| ATOM | 2491 | HA | ASN | A | 177 | 59.349 | 53.299 | 58.965 | 1.00 | 0.75 |
| ATOM | 2492 | CB | ASN | A | 177 | 59.867 | 55.289 | 59.605 | 1.00 | 0.75 |
| ATOM | 2493 | HB1 | ASN | A | 177 | 60.806 | 55.439 | 59.075 | 1.00 | 0.75 |
| ATOM | 2494 | HB2 | ASN | A | 177 | 60.108 | 54.836 | 60.563 | 1.00 | 0.75 |
| ATOM | 2495 | CG | ASN | A | 177 | 59.233 | 56.649 | 59.850 | 1.00 | 0.75 |
| ATOM | 2496 | OD1 | ASN | A | 177 | 58.569 | 57.230 | 59.006 | 1.00 | 0.75 |
| ATOM | 2497 | ND2 | ASN | A | 177 | 59.378 | 57.173 | 61.039 | 1.00 | 0.75 |
| ATOM | 2498 | 1HD2 | ASN | A | 177 | 60.008 | 56.754 | 61.714 | 1.00 | 0.75 |
| ATOM | 2499 | 2HD2 | ASN | A | 177 | 58.829 | 58.004 | 61.234 | 1.00 | 0.75 |
| ATOM | 2500 | C | ASN | A | 177 | 57.526 | 54.264 | 59.370 | 1.00 | 0.75 |
| ATOM | 2501 | O | ASN | A | 177 | 56.577 | 54.415 | 58.609 | 1.00 | 0.75 |
| ATOM | 2502 | N | GLN | A | 178 | 57.377 | 54.079 | 60.682 | 1.00 | 0.85 |
| ATOM | 2503 | H | GLN | A | 178 | 58.206 | 53.955 | 61.248 | 1.00 | 0.85 |
| ATOM | 2504 | CA | GLN | A | 178 | 56.096 | 53.805 | 61.332 | 1.00 | 0.85 |

| | | | | | | | | | | |
|---|---|---|---|---|---|---|---|---|---|---|
| ATOM | 2505 | HA | GLN | A | 178 | 55.300 | 53.823 | 60.584 | 1.00 | 0.85 |
| ATOM | 2506 | CB | GLN | A | 178 | 56.116 | 52.392 | 61.950 | 1.00 | 0.85 |
| ATOM | 2507 | HB1 | GLN | A | 178 | 55.157 | 52.225 | 62.444 | 1.00 | 0.85 |
| ATOM | 2508 | HB2 | GLN | A | 178 | 56.890 | 52.321 | 62.714 | 1.00 | 0.85 |
| ATOM | 2509 | CG | GLN | A | 178 | 56.294 | 51.268 | 60.914 | 1.00 | 0.85 |
| ATOM | 2510 | HG1 | GLN | A | 178 | 55.589 | 51.442 | 60.105 | 1.00 | 0.85 |
| ATOM | 2511 | HG2 | GLN | A | 178 | 56.030 | 50.315 | 61.371 | 1.00 | 0.85 |
| ATOM | 2512 | CD | GLN | A | 178 | 57.702 | 51.129 | 60.337 | 1.00 | 0.85 |
| ATOM | 2513 | OE1 | GLN | A | 178 | 58.713 | 51.178 | 61.034 | 1.00 | 0.85 |
| ATOM | 2514 | NE2 | GLN | A | 178 | 57.848 | 50.974 | 59.047 | 1.00 | 0.85 |
| ATOM | 2515 | 1HE2 | GLN | A | 178 | 57.047 | 50.975 | 58.401 | 1.00 | 0.85 |
| ATOM | 2516 | 2HE2 | GLN | A | 178 | 58.744 | 50.691 | 58.686 | 1.00 | 0.85 |
| ATOM | 2517 | C | GLN | A | 178 | 55.723 | 54.874 | 62.368 | 1.00 | 0.85 |
| ATOM | 2518 | O | GLN | A | 178 | 54.675 | 54.794 | 63.003 | 1.00 | 0.85 |
| ATOM | 2519 | N | ASP | A | 179 | 56.543 | 55.919 | 62.504 | 1.00 | 0.87 |
| ATOM | 2520 | H | ASP | A | 179 | 57.315 | 55.992 | 61.860 | 1.00 | 0.87 |
| ATOM | 2521 | CA | ASP | A | 179 | 56.287 | 57.081 | 63.356 | 1.00 | 0.87 |
| ATOM | 2522 | HA | ASP | A | 179 | 55.213 | 57.157 | 63.485 | 1.00 | 0.87 |
| ATOM | 2523 | CB | ASP | A | 179 | 56.875 | 56.855 | 64.764 | 1.00 | 0.87 |
| ATOM | 2524 | HB1 | ASP | A | 179 | 57.875 | 57.284 | 64.820 | 1.00 | 0.87 |
| ATOM | 2525 | HB2 | ASP | A | 179 | 56.962 | 55.780 | 64.938 | 1.00 | 0.87 |
| ATOM | 2526 | CG | ASP | A | 179 | 55.974 | 57.442 | 65.864 | 1.00 | 0.87 |
| ATOM | 2527 | OD1 | ASP | A | 179 | 55.350 | 58.494 | 65.593 | 1.00 | 0.87 |
| ATOM | 2528 | OD2 | ASP | A | 179 | 55.865 | 56.793 | 66.935 | 1.00 | 0.87 |
| ATOM | 2529 | C | ASP | A | 179 | 56.716 | 58.390 | 62.685 | 1.00 | 0.87 |
| ATOM | 2530 | O | ASP | A | 179 | 56.873 | 58.408 | 61.467 | 1.00 | 0.87 |
| ATOM | 2531 | N | ASP | A | 180 | 56.831 | 59.515 | 63.388 | 1.00 | 0.96 |
| ATOM | 2532 | H | ASP | A | 180 | 56.631 | 59.492 | 64.391 | 1.00 | 0.96 |
| ATOM | 2533 | CA | ASP | A | 180 | 57.188 | 60.786 | 62.754 | 1.00 | 0.96 |
| ATOM | 2534 | HA | ASP | A | 180 | 56.361 | 61.037 | 62.090 | 1.00 | 0.96 |
| ATOM | 2535 | CB | ASP | A | 180 | 57.283 | 61.928 | 63.784 | 1.00 | 0.96 |
| ATOM | 2536 | HB1 | ASP | A | 180 | 58.011 | 62.653 | 63.424 | 1.00 | 0.96 |
| ATOM | 2537 | HB2 | ASP | A | 180 | 57.655 | 61.536 | 64.731 | 1.00 | 0.96 |
| ATOM | 2538 | CG | ASP | A | 180 | 55.963 | 62.684 | 64.013 | 1.00 | 0.96 |
| ATOM | 2539 | OD1 | ASP | A | 180 | 55.217 | 62.981 | 63.050 | 1.00 | 0.96 |
| ATOM | 2540 | OD2 | ASP | A | 180 | 55.654 | 63.059 | 65.169 | 1.00 | 0.96 |
| ATOM | 2541 | C | ASP | A | 180 | 58.432 | 60.719 | 61.851 | 1.00 | 0.96 |
| ATOM | 2542 | O | ASP | A | 180 | 59.494 | 60.196 | 62.192 | 1.00 | 0.96 |
| ATOM | 2543 | N | CYS | A | 181 | 58.262 | 61.255 | 60.641 | 1.00 | 0.82 |
| ATOM | 2544 | H | CYS | A | 181 | 57.387 | 61.733 | 60.462 | 1.00 | 0.82 |
| ATOM | 2545 | CA | CYS | A | 181 | 59.334 | 61.461 | 59.675 | 1.00 | 0.82 |
| ATOM | 2546 | HA | CYS | A | 181 | 59.826 | 60.509 | 59.480 | 1.00 | 0.82 |
| ATOM | 2547 | CB | CYS | A | 181 | 58.650 | 61.967 | 58.386 | 1.00 | 0.82 |
| ATOM | 2548 | HB1 | CYS | A | 181 | 58.214 | 62.939 | 58.595 | 1.00 | 0.82 |

| | | | | | | | | | | |
|---|---|---|---|---|---|---|---|---|---|---|
| ATOM | 2549 | HB2 | CYS | A | 181 | 57.835 | 61.280 | 58.157 | 1.00 | 0.82 |
| ATOM | 2550 | SG | CYS | A | 181 | 59.600 | 62.164 | 56.860 | 1.00 | 0.82 |
| ATOM | 2551 | C | CYS | A | 181 | 60.366 | 62.439 | 60.260 | 1.00 | 0.82 |
| ATOM | 2552 | O | CYS | A | 181 | 61.582 | 62.201 | 60.262 | 1.00 | 0.82 |
| ATOM | 2553 | N | VAL | A | 182 | 59.822 | 63.511 | 60.846 | 1.00 | 0.74 |
| ATOM | 2554 | H | VAL | A | 182 | 58.812 | 63.555 | 60.860 | 1.00 | 0.74 |
| ATOM | 2555 | CA | VAL | A | 182 | 60.524 | 64.546 | 61.595 | 1.00 | 0.74 |
| ATOM | 2556 | HA | VAL | A | 182 | 61.484 | 64.157 | 61.935 | 1.00 | 0.74 |
| ATOM | 2557 | CB | VAL | A | 182 | 60.763 | 65.798 | 60.729 | 1.00 | 0.74 |
| ATOM | 2558 | HB | VAL | A | 182 | 59.798 | 66.217 | 60.437 | 1.00 | 0.74 |
| ATOM | 2559 | CG1 | VAL | A | 182 | 61.561 | 66.875 | 61.483 | 1.00 | 0.74 |
| ATOM | 2560 | 1HG1 | VAL | A | 182 | 61.800 | 67.710 | 60.824 | 1.00 | 0.74 |
| ATOM | 2561 | 2HG1 | VAL | A | 182 | 60.979 | 67.261 | 62.319 | 1.00 | 0.74 |
| ATOM | 2562 | 3HG1 | VAL | A | 182 | 62.488 | 66.445 | 61.864 | 1.00 | 0.74 |
| ATOM | 2563 | CG2 | VAL | A | 182 | 61.548 | 65.488 | 59.456 | 1.00 | 0.74 |
| ATOM | 2564 | 1HG2 | VAL | A | 182 | 61.633 | 66.403 | 58.877 | 1.00 | 0.74 |
| ATOM | 2565 | 2HG2 | VAL | A | 182 | 62.538 | 65.111 | 59.707 | 1.00 | 0.74 |
| ATOM | 2566 | 3HG2 | VAL | A | 182 | 61.028 | 64.755 | 58.842 | 1.00 | 0.74 |
| ATOM | 2567 | C | VAL | A | 182 | 59.691 | 64.947 | 62.806 | 1.00 | 0.74 |
| ATOM | 2568 | O | VAL | A | 182 | 58.480 | 65.128 | 62.682 | 1.00 | 0.74 |
| ATOM | 2569 | N | ALA | A | 183 | 60.347 | 65.157 | 63.944 | 1.00 | 0.79 |
| ATOM | 2570 | H | ALA | A | 183 | 61.327 | 64.890 | 63.979 | 1.00 | 0.79 |
| ATOM | 2571 | CA | ALA | A | 183 | 59.717 | 65.542 | 65.193 | 1.00 | 0.79 |
| ATOM | 2572 | HA | ALA | A | 183 | 58.732 | 65.964 | 64.997 | 1.00 | 0.79 |
| ATOM | 2573 | CB | ALA | A | 183 | 59.506 | 64.283 | 66.047 | 1.00 | 0.79 |
| ATOM | 2574 | HB1 | ALA | A | 183 | 59.116 | 64.561 | 67.027 | 1.00 | 0.79 |
| ATOM | 2575 | HB2 | ALA | A | 183 | 58.794 | 63.618 | 65.562 | 1.00 | 0.79 |
| ATOM | 2576 | HB3 | ALA | A | 183 | 60.450 | 63.752 | 66.181 | 1.00 | 0.79 |
| ATOM | 2577 | C | ALA | A | 183 | 60.548 | 66.604 | 65.931 | 1.00 | 0.79 |
| ATOM | 2578 | O | ALA | A | 183 | 61.480 | 66.314 | 66.682 | 1.00 | 0.79 |
| ATOM | 2579 | N | VAL | A | 184 | 60.227 | 67.873 | 65.677 | 1.00 | 0.70 |
| ATOM | 2580 | H | VAL | A | 184 | 59.487 | 68.061 | 65.007 | 1.00 | 0.70 |
| ATOM | 2581 | CA | VAL | A | 184 | 60.946 | 68.994 | 66.303 | 1.00 | 0.70 |
| ATOM | 2582 | HA | VAL | A | 184 | 61.985 | 68.701 | 66.448 | 1.00 | 0.70 |
| ATOM | 2583 | CB | VAL | A | 184 | 60.953 | 70.253 | 65.407 | 1.00 | 0.70 |
| ATOM | 2584 | HB | VAL | A | 184 | 59.957 | 70.695 | 65.383 | 1.00 | 0.70 |
| ATOM | 2585 | CG1 | VAL | A | 184 | 61.946 | 71.294 | 65.942 | 1.00 | 0.70 |
| ATOM | 2586 | 1HG1 | VAL | A | 184 | 61.949 | 72.171 | 65.296 | 1.00 | 0.70 |
| ATOM | 2587 | 2HG1 | VAL | A | 184 | 61.652 | 71.618 | 66.941 | 1.00 | 0.70 |
| ATOM | 2588 | 3HG1 | VAL | A | 184 | 62.952 | 70.874 | 65.987 | 1.00 | 0.70 |
| ATOM | 2589 | CG2 | VAL | A | 184 | 61.339 | 69.921 | 63.961 | 1.00 | 0.70 |
| ATOM | 2590 | 1HG2 | VAL | A | 184 | 61.483 | 70.837 | 63.389 | 1.00 | 0.70 |
| ATOM | 2591 | 2HG2 | VAL | A | 184 | 62.258 | 69.339 | 63.954 | 1.00 | 0.70 |
| ATOM | 2592 | 3HG2 | VAL | A | 184 | 60.545 | 69.345 | 63.490 | 1.00 | 0.70 |

| | | | | | | | | | | |
|---|---|---|---|---|---|---|---|---|---|---|
| ATOM | 2593 | C | VAL | A | 184 | 60.353 | 69.309 | 67.675 | 1.00 | 0.70 |
| ATOM | 2594 | O | VAL | A | 184 | 59.433 | 70.116 | 67.802 | 1.00 | 0.70 |
| ATOM | 2595 | N | ASN | A | 185 | 60.870 | 68.642 | 68.706 | 1.00 | 0.83 |
| ATOM | 2596 | H | ASN | A | 185 | 61.600 | 67.966 | 68.503 | 1.00 | 0.83 |
| ATOM | 2597 | CA | ASN | A | 185 | 60.627 | 68.977 | 70.111 | 1.00 | 0.83 |
| ATOM | 2598 | HA | ASN | A | 185 | 59.609 | 68.687 | 70.370 | 1.00 | 0.83 |
| ATOM | 2599 | CB | ASN | A | 185 | 61.603 | 68.208 | 71.011 | 1.00 | 0.83 |
| ATOM | 2600 | HB1 | ASN | A | 185 | 61.349 | 68.424 | 72.047 | 1.00 | 0.83 |
| ATOM | 2601 | HB2 | ASN | A | 185 | 62.617 | 68.568 | 70.844 | 1.00 | 0.83 |
| ATOM | 2602 | CG | ASN | A | 185 | 61.594 | 66.704 | 70.816 | 1.00 | 0.83 |
| ATOM | 2603 | OD1 | ASN | A | 185 | 60.867 | 65.986 | 71.483 | 1.00 | 0.83 |
| ATOM | 2604 | ND2 | ASN | A | 185 | 62.483 | 66.226 | 69.982 | 1.00 | 0.83 |
| ATOM | 2605 | 1HD2 | ASN | A | 185 | 63.099 | 66.850 | 69.481 | 1.00 | 0.83 |
| ATOM | 2606 | 2HD2 | ASN | A | 185 | 62.636 | 65.213 | 69.895 | 1.00 | 0.83 |
| ATOM | 2607 | C | ASN | A | 185 | 60.763 | 70.477 | 70.376 | 1.00 | 0.83 |
| ATOM | 2608 | O | ASN | A | 185 | 59.916 | 71.065 | 71.046 | 1.00 | 0.83 |
| ATOM | 2609 | N | SER | A | 186 | 61.851 | 71.029 | 69.836 | 1.00 | 0.83 |
| ATOM | 2610 | H | SER | A | 186 | 62.402 | 70.429 | 69.232 | 1.00 | 0.83 |
| ATOM | 2611 | CA | SER | A | 186 | 62.600 | 72.167 | 70.354 | 1.00 | 0.83 |
| ATOM | 2612 | HA | SER | A | 186 | 61.968 | 73.038 | 70.539 | 1.00 | 0.83 |
| ATOM | 2613 | CB | SER | A | 186 | 63.276 | 71.673 | 71.636 | 1.00 | 0.83 |
| ATOM | 2614 | HB1 | SER | A | 186 | 64.204 | 72.219 | 71.788 | 1.00 | 0.83 |
| ATOM | 2615 | HB2 | SER | A | 186 | 63.531 | 70.617 | 71.554 | 1.00 | 0.83 |
| ATOM | 2616 | OG | SER | A | 186 | 62.416 | 71.863 | 72.733 | 1.00 | 0.83 |
| ATOM | 2617 | HG | SER | A | 186 | 62.625 | 72.792 | 72.993 | 1.00 | 0.83 |
| ATOM | 2618 | C | SER | A | 186 | 63.714 | 72.490 | 69.367 | 1.00 | 0.83 |
| ATOM | 2619 | O | SER | A | 186 | 64.306 | 71.562 | 68.801 | 1.00 | 0.83 |
| ATOM | 2620 | N | GLY | A | 187 | 64.034 | 73.765 | 69.178 | 1.00 | 0.88 |
| ATOM | 2621 | H | GLY | A | 187 | 63.561 | 74.494 | 69.711 | 1.00 | 0.88 |
| ATOM | 2622 | CA | GLY | A | 187 | 65.143 | 74.145 | 68.306 | 1.00 | 0.88 |
| ATOM | 2623 | HA1 | GLY | A | 187 | 65.415 | 73.334 | 67.632 | 1.00 | 0.88 |
| ATOM | 2624 | HA2 | GLY | A | 187 | 66.009 | 74.362 | 68.933 | 1.00 | 0.88 |
| ATOM | 2625 | C | GLY | A | 187 | 64.858 | 75.367 | 67.457 | 1.00 | 0.88 |
| ATOM | 2626 | O | GLY | A | 187 | 63.711 | 75.769 | 67.269 | 1.00 | 0.88 |
| ATOM | 2627 | N | THR | A | 188 | 65.932 | 75.912 | 66.899 | 1.00 | 0.83 |
| ATOM | 2628 | H | THR | A | 188 | 66.823 | 75.440 | 67.022 | 1.00 | 0.83 |
| ATOM | 2629 | CA | THR | A | 188 | 65.919 | 77.149 | 66.121 | 1.00 | 0.83 |
| ATOM | 2630 | HA | THR | A | 188 | 64.915 | 77.465 | 65.971 | 1.00 | 0.83 |
| ATOM | 2631 | CB | THR | A | 188 | 66.695 | 78.276 | 66.835 | 1.00 | 0.83 |
| ATOM | 2632 | HB | THR | A | 188 | 66.709 | 79.161 | 66.199 | 1.00 | 0.83 |
| ATOM | 2633 | CG2 | THR | A | 188 | 66.152 | 78.675 | 68.206 | 1.00 | 0.83 |
| ATOM | 2634 | 1HG2 | THR | A | 188 | 66.829 | 79.392 | 68.671 | 1.00 | 0.83 |
| ATOM | 2635 | 2HG2 | THR | A | 188 | 65.183 | 79.154 | 68.094 | 1.00 | 0.83 |
| ATOM | 2636 | 3HG2 | THR | A | 188 | 66.048 | 77.805 | 68.854 | 1.00 | 0.83 |

| | | | | | | | | | | |
|---|---|---|---|---|---|---|---|---|---|---|
| ATOM | 2637 | OG1 | THR | A | 188 | 68.025 | 77.886 | 67.054 | 1.00 | 0.83 |
| ATOM | 2638 | HG1 | THR | A | 188 | 68.030 | 77.273 | 67.794 | 1.00 | 0.83 |
| ATOM | 2639 | C | THR | A | 188 | 66.525 | 76.930 | 64.742 | 1.00 | 0.83 |
| ATOM | 2640 | O | THR | A | 188 | 67.422 | 76.101 | 64.567 | 1.00 | 0.83 |
| ATOM | 2641 | N | SER | A | 189 | 66.079 | 77.700 | 63.749 | 1.00 | 0.42 |
| ATOM | 2642 | H | SER | A | 189 | 65.396 | 78.422 | 63.978 | 1.00 | 0.42 |
| ATOM | 2643 | CA | SER | A | 189 | 66.630 | 77.651 | 62.391 | 1.00 | 0.42 |
| ATOM | 2644 | HA | SER | A | 189 | 65.982 | 78.225 | 61.731 | 1.00 | 0.42 |
| ATOM | 2645 | CB | SER | A | 189 | 68.003 | 78.364 | 62.447 | 1.00 | 0.42 |
| ATOM | 2646 | HB1 | SER | A | 189 | 68.645 | 77.838 | 63.152 | 1.00 | 0.42 |
| ATOM | 2647 | HB2 | SER | A | 189 | 67.850 | 79.370 | 62.840 | 1.00 | 0.42 |
| ATOM | 2648 | OG | SER | A | 189 | 68.709 | 78.476 | 61.225 | 1.00 | 0.42 |
| ATOM | 2649 | HG | SER | A | 189 | 69.628 | 78.714 | 61.456 | 1.00 | 0.42 |
| ATOM | 2650 | C | SER | A | 189 | 66.689 | 76.215 | 61.845 | 1.00 | 0.42 |
| ATOM | 2651 | O | SER | A | 189 | 67.747 | 75.664 | 61.505 | 1.00 | 0.42 |
| ATOM | 2652 | N | ILE | A | 190 | 65.525 | 75.553 | 61.847 | 1.00 | 0.36 |
| ATOM | 2653 | H | ILE | A | 190 | 64.685 | 76.017 | 62.174 | 1.00 | 0.36 |
| ATOM | 2654 | CA | ILE | A | 190 | 65.474 | 74.158 | 61.365 | 1.00 | 0.36 |
| ATOM | 2655 | HA | ILE | A | 190 | 66.474 | 73.733 | 61.316 | 1.00 | 0.36 |
| ATOM | 2656 | CB | ILE | A | 190 | 64.647 | 73.284 | 62.338 | 1.00 | 0.36 |
| ATOM | 2657 | HB | ILE | A | 190 | 63.605 | 73.578 | 62.239 | 1.00 | 0.36 |
| ATOM | 2658 | CG2 | ILE | A | 190 | 64.748 | 71.797 | 61.947 | 1.00 | 0.36 |
| ATOM | 2659 | 1HG2 | ILE | A | 190 | 64.231 | 71.184 | 62.684 | 1.00 | 0.36 |
| ATOM | 2660 | 2HG2 | ILE | A | 190 | 64.278 | 71.632 | 60.977 | 1.00 | 0.36 |
| ATOM | 2661 | 3HG2 | ILE | A | 190 | 65.792 | 71.488 | 61.889 | 1.00 | 0.36 |
| ATOM | 2662 | CG1 | ILE | A | 190 | 65.010 | 73.461 | 63.835 | 1.00 | 0.36 |
| ATOM | 2663 | 1HG1 | ILE | A | 190 | 64.327 | 72.857 | 64.433 | 1.00 | 0.36 |
| ATOM | 2664 | 2HG1 | ILE | A | 190 | 64.840 | 74.498 | 64.126 | 1.00 | 0.36 |
| ATOM | 2665 | CD1 | ILE | A | 190 | 66.444 | 73.079 | 64.222 | 1.00 | 0.36 |
| ATOM | 2666 | HD1 | ILE | A | 190 | 66.616 | 73.341 | 65.266 | 1.00 | 0.36 |
| ATOM | 2667 | HD2 | ILE | A | 190 | 66.589 | 72.008 | 64.110 | 1.00 | 0.36 |
| ATOM | 2668 | HD3 | ILE | A | 190 | 67.165 | 73.611 | 63.602 | 1.00 | 0.36 |
| ATOM | 2669 | C | ILE | A | 190 | 64.881 | 74.170 | 59.957 | 1.00 | 0.36 |
| ATOM | 2670 | O | ILE | A | 190 | 63.873 | 74.814 | 59.690 | 1.00 | 0.36 |
| ATOM | 2671 | N | THR | A | 191 | 65.470 | 73.419 | 59.045 | 1.00 | 0.42 |
| ATOM | 2672 | H | THR | A | 191 | 66.304 | 72.907 | 59.320 | 1.00 | 0.42 |
| ATOM | 2673 | CA | THR | A | 191 | 65.013 | 73.253 | 57.666 | 1.00 | 0.42 |
| ATOM | 2674 | HA | THR | A | 191 | 64.008 | 73.659 | 57.568 | 1.00 | 0.42 |
| ATOM | 2675 | CB | THR | A | 191 | 65.914 | 73.989 | 56.656 | 1.00 | 0.42 |
| ATOM | 2676 | HB | THR | A | 191 | 65.819 | 73.519 | 55.676 | 1.00 | 0.42 |
| ATOM | 2677 | CG2 | THR | A | 191 | 65.542 | 75.460 | 56.517 | 1.00 | 0.42 |
| ATOM | 2678 | 1HG2 | THR | A | 191 | 66.274 | 75.965 | 55.886 | 1.00 | 0.42 |
| ATOM | 2679 | 2HG2 | THR | A | 191 | 64.568 | 75.537 | 56.042 | 1.00 | 0.42 |
| ATOM | 2680 | 3HG2 | THR | A | 191 | 65.516 | 75.941 | 57.496 | 1.00 | 0.42 |

| | | | | | | | | | | |
|---|---|---|---|---|---|---|---|---|---|---|
| ATOM | 2681 | OG1 | THR | A | 191 | 67.265 | 73.974 | 57.043 | 1.00 | 0.42 |
| ATOM | 2682 | HG1 | THR | A | 191 | 67.315 | 74.390 | 57.911 | 1.00 | 0.42 |
| ATOM | 2683 | C | THR | A | 191 | 64.931 | 71.767 | 57.345 | 1.00 | 0.42 |
| ATOM | 2684 | O | THR | A | 191 | 65.743 | 70.957 | 57.799 | 1.00 | 0.42 |
| ATOM | 2685 | N | PHE | A | 192 | 63.919 | 71.405 | 56.567 | 1.00 | 0.55 |
| ATOM | 2686 | H | PHE | A | 192 | 63.253 | 72.118 | 56.275 | 1.00 | 0.55 |
| ATOM | 2687 | CA | PHE | A | 192 | 63.731 | 70.078 | 56.019 | 1.00 | 0.55 |
| ATOM | 2688 | HA | PHE | A | 192 | 64.677 | 69.541 | 56.000 | 1.00 | 0.55 |
| ATOM | 2689 | CB | PHE | A | 192 | 62.732 | 69.276 | 56.868 | 1.00 | 0.55 |
| ATOM | 2690 | HB1 | PHE | A | 192 | 61.804 | 69.841 | 56.946 | 1.00 | 0.55 |
| ATOM | 2691 | HB2 | PHE | A | 192 | 63.136 | 69.175 | 57.877 | 1.00 | 0.55 |
| ATOM | 2692 | CG | PHE | A | 192 | 62.418 | 67.891 | 56.318 | 1.00 | 0.55 |
| ATOM | 2693 | CD1 | PHE | A | 192 | 63.108 | 66.767 | 56.807 | 1.00 | 0.55 |
| ATOM | 2694 | HD1 | PHE | A | 192 | 63.838 | 66.886 | 57.594 | 1.00 | 0.55 |
| ATOM | 2695 | CE1 | PHE | A | 192 | 62.813 | 65.480 | 56.320 | 1.00 | 0.55 |
| ATOM | 2696 | HE1 | PHE | A | 192 | 63.326 | 64.618 | 56.725 | 1.00 | 0.55 |
| ATOM | 2697 | CZ | PHE | A | 192 | 61.833 | 65.310 | 55.328 | 1.00 | 0.55 |
| ATOM | 2698 | HZ | PHE | A | 192 | 61.589 | 64.319 | 54.970 | 1.00 | 0.55 |
| ATOM | 2699 | CE2 | PHE | A | 192 | 61.149 | 66.429 | 54.823 | 1.00 | 0.55 |
| ATOM | 2700 | HE2 | PHE | A | 192 | 60.390 | 66.303 | 54.063 | 1.00 | 0.55 |
| ATOM | 2701 | CD2 | PHE | A | 192 | 61.432 | 67.715 | 55.324 | 1.00 | 0.55 |
| ATOM | 2702 | HD2 | PHE | A | 192 | 60.882 | 68.564 | 54.943 | 1.00 | 0.55 |
| ATOM | 2703 | C | PHE | A | 192 | 63.231 | 70.253 | 54.593 | 1.00 | 0.55 |
| ATOM | 2704 | O | PHE | A | 192 | 62.146 | 70.798 | 54.386 | 1.00 | 0.55 |
| ATOM | 2705 | N | SER | A | 193 | 64.022 | 69.831 | 53.610 | 1.00 | 0.50 |
| ATOM | 2706 | H | SER | A | 193 | 64.924 | 69.430 | 53.845 | 1.00 | 0.50 |
| ATOM | 2707 | CA | SER | A | 193 | 63.724 | 70.072 | 52.201 | 1.00 | 0.50 |
| ATOM | 2708 | HA | SER | A | 193 | 62.678 | 70.312 | 52.113 | 1.00 | 0.50 |
| ATOM | 2709 | CB | SER | A | 193 | 64.505 | 71.302 | 51.711 | 1.00 | 0.50 |
| ATOM | 2710 | HB1 | SER | A | 193 | 65.537 | 71.215 | 52.054 | 1.00 | 0.50 |
| ATOM | 2711 | HB2 | SER | A | 193 | 64.072 | 72.199 | 52.155 | 1.00 | 0.50 |
| ATOM | 2712 | OG | SER | A | 193 | 64.530 | 71.436 | 50.299 | 1.00 | 0.50 |
| ATOM | 2713 | HG | SER | A | 193 | 63.636 | 71.577 | 49.924 | 1.00 | 0.50 |
| ATOM | 2714 | C | SER | A | 193 | 63.999 | 68.859 | 51.316 | 1.00 | 0.50 |
| ATOM | 2715 | O | SER | A | 193 | 64.902 | 68.046 | 51.557 | 1.00 | 0.50 |
| ATOM | 2716 | N | GLY | A | 194 | 63.195 | 68.735 | 50.263 | 1.00 | 0.45 |
| ATOM | 2717 | H | GLY | A | 194 | 62.444 | 69.406 | 50.143 | 1.00 | 0.45 |
| ATOM | 2718 | CA | GLY | A | 194 | 63.336 | 67.633 | 49.309 | 1.00 | 0.45 |
| ATOM | 2719 | HA1 | GLY | A | 194 | 64.374 | 67.309 | 49.229 | 1.00 | 0.45 |
| ATOM | 2720 | HA2 | GLY | A | 194 | 62.991 | 67.948 | 48.324 | 1.00 | 0.45 |
| ATOM | 2721 | C | GLY | A | 194 | 62.469 | 66.477 | 49.788 | 1.00 | 0.45 |
| ATOM | 2722 | O | GLY | A | 194 | 61.332 | 66.350 | 49.345 | 1.00 | 0.45 |
| ATOM | 2723 | N | GLY | A | 195 | 62.954 | 65.746 | 50.793 | 1.00 | 0.76 |
| ATOM | 2724 | H | GLY | A | 195 | 63.919 | 65.860 | 51.054 | 1.00 | 0.76 |

| | | | | | | | | | | |
|---|---|---|---|---|---|---|---|---|---|---|
| ATOM | 2725 | CA | GLY | A | 195 | 62.130 | 64.849 | 51.596 | 1.00 | 0.76 |
| ATOM | 2726 | HA1 | GLY | A | 195 | 61.242 | 65.398 | 51.909 | 1.00 | 0.76 |
| ATOM | 2727 | HA2 | GLY | A | 195 | 62.664 | 64.572 | 52.497 | 1.00 | 0.76 |
| ATOM | 2728 | C | GLY | A | 195 | 61.648 | 63.591 | 50.877 | 1.00 | 0.76 |
| ATOM | 2729 | O | GLY | A | 195 | 62.056 | 63.312 | 49.761 | 1.00 | 0.76 |
| ATOM | 2730 | N | THR | A | 196 | 60.770 | 62.820 | 51.523 | 1.00 | 0.64 |
| ATOM | 2731 | H | THR | A | 196 | 60.423 | 63.194 | 52.391 | 1.00 | 0.64 |
| ATOM | 2732 | CA | THR | A | 196 | 59.902 | 61.805 | 50.882 | 1.00 | 0.64 |
| ATOM | 2733 | HA | THR | A | 196 | 59.472 | 62.264 | 49.998 | 1.00 | 0.64 |
| ATOM | 2734 | CB | THR | A | 196 | 60.599 | 60.492 | 50.446 | 1.00 | 0.64 |
| ATOM | 2735 | HB | THR | A | 196 | 60.750 | 59.853 | 51.312 | 1.00 | 0.64 |
| ATOM | 2736 | CG2 | THR | A | 196 | 59.743 | 59.716 | 49.448 | 1.00 | 0.64 |
| ATOM | 2737 | 1HG2 | THR | A | 196 | 58.774 | 59.477 | 49.885 | 1.00 | 0.64 |
| ATOM | 2738 | 2HG2 | THR | A | 196 | 59.589 | 60.310 | 48.546 | 1.00 | 0.64 |
| ATOM | 2739 | 3HG2 | THR | A | 196 | 60.244 | 58.784 | 49.183 | 1.00 | 0.64 |
| ATOM | 2740 | OG1 | THR | A | 196 | 61.846 | 60.678 | 49.816 | 1.00 | 0.64 |
| ATOM | 2741 | HG1 | THR | A | 196 | 61.909 | 61.633 | 49.607 | 1.00 | 0.64 |
| ATOM | 2742 | C | THR | A | 196 | 58.749 | 61.425 | 51.845 | 1.00 | 0.64 |
| ATOM | 2743 | O | THR | A | 196 | 57.544 | 61.459 | 51.554 | 1.00 | 0.64 |
| ATOM | 2744 | N | CYS | A | 197 | 59.172 | 61.084 | 53.061 | 1.00 | 0.46 |
| ATOM | 2745 | H | CYS | A | 197 | 60.174 | 61.036 | 53.199 | 1.00 | 0.46 |
| ATOM | 2746 | CA | CYS | A | 197 | 58.342 | 60.493 | 54.103 | 1.00 | 0.46 |
| ATOM | 2747 | HA | CYS | A | 197 | 59.054 | 60.377 | 54.913 | 1.00 | 0.46 |
| ATOM | 2748 | CB | CYS | A | 197 | 57.366 | 61.472 | 54.754 | 1.00 | 0.46 |
| ATOM | 2749 | HB1 | CYS | A | 197 | 56.792 | 60.959 | 55.525 | 1.00 | 0.46 |
| ATOM | 2750 | HB2 | CYS | A | 197 | 56.693 | 61.851 | 53.990 | 1.00 | 0.46 |
| ATOM | 2751 | SG | CYS | A | 197 | 58.218 | 62.895 | 55.521 | 1.00 | 0.46 |
| ATOM | 2752 | C | CYS | A | 197 | 58.023 | 59.006 | 53.888 | 1.00 | 0.46 |
| ATOM | 2753 | O | CYS | A | 197 | 58.959 | 58.232 | 53.666 | 1.00 | 0.46 |
| ATOM | 2754 | N | SER | A | 198 | 56.761 | 58.595 | 53.991 | 1.00 | 0.62 |
| ATOM | 2755 | H | SER | A | 198 | 56.071 | 59.282 | 54.245 | 1.00 | 0.62 |
| ATOM | 2756 | CA | SER | A | 198 | 56.239 | 57.251 | 53.677 | 1.00 | 0.62 |
| ATOM | 2757 | HA | SER | A | 198 | 55.294 | 57.393 | 53.174 | 1.00 | 0.62 |
| ATOM | 2758 | CB | SER | A | 198 | 57.053 | 56.402 | 52.692 | 1.00 | 0.62 |
| ATOM | 2759 | HB1 | SER | A | 198 | 56.458 | 55.536 | 52.396 | 1.00 | 0.62 |
| ATOM | 2760 | HB2 | SER | A | 198 | 57.963 | 56.041 | 53.169 | 1.00 | 0.62 |
| ATOM | 2761 | OG | SER | A | 198 | 57.388 | 57.144 | 51.542 | 1.00 | 0.62 |
| ATOM | 2762 | HG | SER | A | 198 | 58.110 | 57.721 | 51.818 | 1.00 | 0.62 |
| ATOM | 2763 | C | SER | A | 198 | 56.002 | 56.402 | 54.924 | 1.00 | 0.62 |
| ATOM | 2764 | O | SER | A | 198 | 56.938 | 56.107 | 55.677 | 1.00 | 0.62 |
| ATOM | 2765 | N | GLY | A | 199 | 54.743 | 56.003 | 55.124 | 1.00 | 0.84 |
| ATOM | 2766 | H | GLY | A | 199 | 53.994 | 56.328 | 54.523 | 1.00 | 0.84 |
| ATOM | 2767 | CA | GLY | A | 199 | 54.390 | 55.041 | 56.179 | 1.00 | 0.84 |
| ATOM | 2768 | HA1 | GLY | A | 199 | 55.222 | 54.355 | 56.343 | 1.00 | 0.84 |

| | | | | | | | | | | |
|---|---|---|---|---|---|---|---|---|---|---|
| ATOM | 2769 | HA2 | GLY | A | 199 | 53.544 | 54.427 | 55.872 | 1.00 | 0.84 |
| ATOM | 2770 | C | GLY | A | 199 | 54.062 | 55.687 | 57.525 | 1.00 | 0.84 |
| ATOM | 2771 | O | GLY | A | 199 | 53.100 | 55.328 | 58.201 | 1.00 | 0.84 |
| ATOM | 2772 | N | GLY | A | 200 | 54.877 | 56.674 | 57.894 | 1.00 | 0.95 |
| ATOM | 2773 | H | GLY | A | 200 | 55.682 | 56.816 | 57.305 | 1.00 | 0.95 |
| ATOM | 2774 | CA | GLY | A | 200 | 54.933 | 57.221 | 59.239 | 1.00 | 0.95 |
| ATOM | 2775 | HA1 | GLY | A | 200 | 55.894 | 57.721 | 59.341 | 1.00 | 0.95 |
| ATOM | 2776 | HA2 | GLY | A | 200 | 54.922 | 56.400 | 59.957 | 1.00 | 0.95 |
| ATOM | 2777 | C | GLY | A | 200 | 53.843 | 58.196 | 59.632 | 1.00 | 0.95 |
| ATOM | 2778 | O | GLY | A | 200 | 52.789 | 58.308 | 59.014 | 1.00 | 0.95 |
| ATOM | 2779 | N | HIS | A | 201 | 54.089 | 58.932 | 60.710 | 1.00 | 0.77 |
| ATOM | 2780 | H | HIS | A | 201 | 54.976 | 58.807 | 61.191 | 1.00 | 0.77 |
| ATOM | 2781 | CA | HIS | A | 201 | 53.119 | 59.915 | 61.166 | 1.00 | 0.77 |
| ATOM | 2782 | HA | HIS | A | 201 | 52.132 | 59.477 | 61.055 | 1.00 | 0.77 |
| ATOM | 2783 | CB | HIS | A | 201 | 53.287 | 60.266 | 62.646 | 1.00 | 0.77 |
| ATOM | 2784 | HB1 | HIS | A | 201 | 52.748 | 61.192 | 62.830 | 1.00 | 0.77 |
| ATOM | 2785 | HB2 | HIS | A | 201 | 54.334 | 60.447 | 62.864 | 1.00 | 0.77 |
| ATOM | 2786 | CG | HIS | A | 201 | 52.764 | 59.231 | 63.609 | 1.00 | 0.77 |
| ATOM | 2787 | ND1 | HIS | A | 201 | 52.324 | 59.500 | 64.910 | 1.00 | 0.77 |
| ATOM | 2788 | CE1 | HIS | A | 201 | 52.222 | 58.293 | 65.500 | 1.00 | 0.77 |
| ATOM | 2789 | HE1 | HIS | A | 201 | 52.022 | 58.137 | 66.552 | 1.00 | 0.77 |
| ATOM | 2790 | NE2 | HIS | A | 201 | 52.480 | 57.307 | 64.623 | 1.00 | 0.77 |
| ATOM | 2791 | HE2 | HIS | A | 201 | 52.618 | 56.328 | 64.850 | 1.00 | 0.77 |
| ATOM | 2792 | CD2 | HIS | A | 201 | 52.804 | 57.881 | 63.420 | 1.00 | 0.77 |
| ATOM | 2793 | HD2 | HIS | A | 201 | 53.159 | 57.355 | 62.544 | 1.00 | 0.77 |
| ATOM | 2794 | C | HIS | A | 201 | 53.108 | 61.162 | 60.305 | 1.00 | 0.77 |
| ATOM | 2795 | O | HIS | A | 201 | 52.032 | 61.715 | 60.179 | 1.00 | 0.77 |
| ATOM | 2796 | N | GLY | A | 202 | 54.216 | 61.627 | 59.734 | 1.00 | 0.85 |
| ATOM | 2797 | H | GLY | A | 202 | 55.087 | 61.140 | 59.888 | 1.00 | 0.85 |
| ATOM | 2798 | CA | GLY | A | 202 | 54.219 | 62.899 | 59.004 | 1.00 | 0.85 |
| ATOM | 2799 | HA1 | GLY | A | 202 | 53.288 | 63.445 | 59.161 | 1.00 | 0.85 |
| ATOM | 2800 | HA2 | GLY | A | 202 | 54.289 | 62.694 | 57.935 | 1.00 | 0.85 |
| ATOM | 2801 | C | GLY | A | 202 | 55.364 | 63.827 | 59.380 | 1.00 | 0.85 |
| ATOM | 2802 | O | GLY | A | 202 | 56.356 | 63.406 | 59.972 | 1.00 | 0.85 |
| ATOM | 2803 | N | LEU | A | 203 | 55.225 | 65.099 | 59.029 | 1.00 | 0.77 |
| ATOM | 2804 | H | LEU | A | 203 | 54.362 | 65.386 | 58.576 | 1.00 | 0.77 |
| ATOM | 2805 | CA | LEU | A | 203 | 56.178 | 66.136 | 59.414 | 1.00 | 0.77 |
| ATOM | 2806 | HA | LEU | A | 203 | 57.121 | 65.700 | 59.743 | 1.00 | 0.77 |
| ATOM | 2807 | CB | LEU | A | 203 | 56.439 | 67.034 | 58.191 | 1.00 | 0.77 |
| ATOM | 2808 | HB1 | LEU | A | 203 | 57.034 | 67.891 | 58.503 | 1.00 | 0.77 |
| ATOM | 2809 | HB2 | LEU | A | 203 | 55.482 | 67.409 | 57.822 | 1.00 | 0.77 |
| ATOM | 2810 | CG | LEU | A | 203 | 57.165 | 66.321 | 57.035 | 1.00 | 0.77 |
| ATOM | 2811 | HG | LEU | A | 203 | 56.620 | 65.415 | 56.769 | 1.00 | 0.77 |
| ATOM | 2812 | CD1 | LEU | A | 203 | 57.206 | 67.224 | 55.805 | 1.00 | 0.77 |

| | | | | | | | | | | |
|---|---|---|---|---|---|---|---|---|---|---|
| ATOM | 2813 | 1HD1 | LEU | A | 203 | 57.727 | 66.716 | 54.996 | 1.00 | 0.77 |
| ATOM | 2814 | 2HD1 | LEU | A | 203 | 56.191 | 67.438 | 55.469 | 1.00 | 0.77 |
| ATOM | 2815 | 3HD1 | LEU | A | 203 | 57.715 | 68.159 | 56.032 | 1.00 | 0.77 |
| ATOM | 2816 | CD2 | LEU | A | 203 | 58.595 | 65.940 | 57.425 | 1.00 | 0.77 |
| ATOM | 2817 | 1HD2 | LEU | A | 203 | 59.086 | 65.474 | 56.574 | 1.00 | 0.77 |
| ATOM | 2818 | 2HD2 | LEU | A | 203 | 59.160 | 66.824 | 57.711 | 1.00 | 0.77 |
| ATOM | 2819 | 3HD2 | LEU | A | 203 | 58.597 | 65.230 | 58.247 | 1.00 | 0.77 |
| ATOM | 2820 | C | LEU | A | 203 | 55.590 | 66.936 | 60.574 | 1.00 | 0.77 |
| ATOM | 2821 | O | LEU | A | 203 | 54.698 | 67.784 | 60.420 | 1.00 | 0.77 |
| ATOM | 2822 | N | SER | A | 204 | 56.129 | 66.636 | 61.752 | 1.00 | 0.80 |
| ATOM | 2823 | H | SER | A | 204 | 56.867 | 65.943 | 61.784 | 1.00 | 0.80 |
| ATOM | 2824 | CA | SER | A | 204 | 55.678 | 67.178 | 63.016 | 1.00 | 0.80 |
| ATOM | 2825 | HA | SER | A | 204 | 54.703 | 67.627 | 62.884 | 1.00 | 0.80 |
| ATOM | 2826 | CB | SER | A | 204 | 55.516 | 66.084 | 64.068 | 1.00 | 0.80 |
| ATOM | 2827 | HB1 | SER | A | 204 | 55.372 | 66.549 | 65.041 | 1.00 | 0.80 |
| ATOM | 2828 | HB2 | SER | A | 204 | 56.404 | 65.457 | 64.115 | 1.00 | 0.80 |
| ATOM | 2829 | OG | SER | A | 204 | 54.374 | 65.295 | 63.798 | 1.00 | 0.80 |
| ATOM | 2830 | HG | SER | A | 204 | 54.706 | 64.465 | 63.358 | 1.00 | 0.80 |
| ATOM | 2831 | C | SER | A | 204 | 56.655 | 68.211 | 63.583 | 1.00 | 0.80 |
| ATOM | 2832 | O | SER | A | 204 | 57.846 | 67.977 | 63.799 | 1.00 | 0.80 |
| ATOM | 2833 | N | ILE | A | 205 | 56.082 | 69.354 | 63.955 | 1.00 | 0.95 |
| ATOM | 2834 | H | ILE | A | 205 | 55.105 | 69.497 | 63.689 | 1.00 | 0.95 |
| ATOM | 2835 | CA | ILE | A | 205 | 56.524 | 70.073 | 65.140 | 1.00 | 0.95 |
| ATOM | 2836 | HA | ILE | A | 205 | 57.597 | 69.913 | 65.262 | 1.00 | 0.95 |
| ATOM | 2837 | CB | ILE | A | 205 | 56.270 | 71.567 | 64.991 | 1.00 | 0.95 |
| ATOM | 2838 | HB | ILE | A | 205 | 55.321 | 71.727 | 64.478 | 1.00 | 0.95 |
| ATOM | 2839 | CG2 | ILE | A | 205 | 56.218 | 72.315 | 66.341 | 1.00 | 0.95 |
| ATOM | 2840 | 1HG2 | ILE | A | 205 | 55.366 | 71.976 | 66.931 | 1.00 | 0.95 |
| ATOM | 2841 | 2HG2 | ILE | A | 205 | 57.142 | 72.144 | 66.893 | 1.00 | 0.95 |
| ATOM | 2842 | 3HG2 | ILE | A | 205 | 56.103 | 73.380 | 66.167 | 1.00 | 0.95 |
| ATOM | 2843 | CG1 | ILE | A | 205 | 57.428 | 72.109 | 64.121 | 1.00 | 0.95 |
| ATOM | 2844 | 1HG1 | ILE | A | 205 | 57.638 | 71.406 | 63.319 | 1.00 | 0.95 |
| ATOM | 2845 | 2HG1 | ILE | A | 205 | 58.328 | 72.210 | 64.724 | 1.00 | 0.95 |
| ATOM | 2846 | CD1 | ILE | A | 205 | 57.093 | 73.440 | 63.490 | 1.00 | 0.95 |
| ATOM | 2847 | HD1 | ILE | A | 205 | 57.909 | 73.745 | 62.841 | 1.00 | 0.95 |
| ATOM | 2848 | HD2 | ILE | A | 205 | 56.191 | 73.319 | 62.893 | 1.00 | 0.95 |
| ATOM | 2849 | HD3 | ILE | A | 205 | 56.945 | 74.197 | 64.252 | 1.00 | 0.95 |
| ATOM | 2850 | C | ILE | A | 205 | 55.858 | 69.434 | 66.347 | 1.00 | 0.95 |
| ATOM | 2851 | O | ILE | A | 205 | 54.670 | 69.095 | 66.379 | 1.00 | 0.95 |
| ATOM | 2852 | N | GLY | A | 206 | 56.689 | 69.294 | 67.357 | 1.00 | 1.13 |
| ATOM | 2853 | H | GLY | A | 206 | 57.624 | 69.603 | 67.242 | 1.00 | 1.13 |
| ATOM | 2854 | CA | GLY | A | 206 | 56.272 | 69.002 | 68.705 | 1.00 | 1.13 |
| ATOM | 2855 | HA1 | GLY | A | 206 | 55.355 | 69.537 | 68.886 | 1.00 | 1.13 |
| ATOM | 2856 | HA2 | GLY | A | 206 | 57.034 | 69.387 | 69.366 | 1.00 | 1.13 |

| | | | | | | | | | | |
|---|---|---|---|---|---|---|---|---|---|---|
| ATOM | 2857 | C | GLY | A | 206 | 56.032 | 67.484 | 69.045 | 1.00 | 1.13 |
| ATOM | 2858 | O | GLY | A | 206 | 56.259 | 66.659 | 68.163 | 1.00 | 1.13 |
| ATOM | 2859 | N | SER | A | 207 | 55.591 | 66.930 | 70.222 | 1.00 | 0.92 |
| ATOM | 2860 | H | SER | A | 207 | 55.646 | 65.939 | 70.181 | 1.00 | 0.92 |
| ATOM | 2861 | CA | SER | A | 207 | 55.014 | 67.305 | 71.580 | 1.00 | 0.92 |
| ATOM | 2862 | HA | SER | A | 207 | 54.018 | 67.689 | 71.405 | 1.00 | 0.92 |
| ATOM | 2863 | CB | SER | A | 207 | 54.903 | 66.073 | 72.474 | 1.00 | 0.92 |
| ATOM | 2864 | HB1 | SER | A | 207 | 54.376 | 66.311 | 73.393 | 1.00 | 0.92 |
| ATOM | 2865 | HB2 | SER | A | 207 | 55.882 | 65.666 | 72.713 | 1.00 | 0.92 |
| ATOM | 2866 | OG | SER | A | 207 | 54.169 | 65.061 | 71.784 | 1.00 | 0.92 |
| ATOM | 2867 | HG | SER | A | 207 | 53.868 | 64.402 | 72.421 | 1.00 | 0.92 |
| ATOM | 2868 | C | SER | A | 207 | 55.818 | 68.438 | 72.259 | 1.00 | 0.92 |
| ATOM | 2869 | O | SER | A | 207 | 56.649 | 68.167 | 73.114 | 1.00 | 0.92 |
| ATOM | 2870 | N | VAL | A | 208 | 55.609 | 69.700 | 71.856 | 1.00 | 0.78 |
| ATOM | 2871 | H | VAL | A | 208 | 54.903 | 69.882 | 71.157 | 1.00 | 0.78 |
| ATOM | 2872 | CA | VAL | A | 208 | 56.216 | 70.868 | 72.536 | 1.00 | 0.78 |
| ATOM | 2873 | HA | VAL | A | 208 | 57.284 | 70.679 | 72.665 | 1.00 | 0.78 |
| ATOM | 2874 | CB | VAL | A | 208 | 56.074 | 72.182 | 71.718 | 1.00 | 0.78 |
| ATOM | 2875 | HB | VAL | A | 208 | 55.025 | 72.480 | 71.718 | 1.00 | 0.78 |
| ATOM | 2876 | CG1 | VAL | A | 208 | 56.904 | 73.326 | 72.315 | 1.00 | 0.78 |
| ATOM | 2877 | 1HG1 | VAL | A | 208 | 56.786 | 74.229 | 71.714 | 1.00 | 0.78 |
| ATOM | 2878 | 2HG1 | VAL | A | 208 | 56.570 | 73.572 | 73.320 | 1.00 | 0.78 |
| ATOM | 2879 | 3HG1 | VAL | A | 208 | 57.961 | 73.054 | 72.331 | 1.00 | 0.78 |
| ATOM | 2880 | CG2 | VAL | A | 208 | 56.506 | 72.026 | 70.254 | 1.00 | 0.78 |
| ATOM | 2881 | 1HG2 | VAL | A | 208 | 55.752 | 71.464 | 69.715 | 1.00 | 0.78 |
| ATOM | 2882 | 2HG2 | VAL | A | 208 | 56.586 | 73.007 | 69.789 | 1.00 | 0.78 |
| ATOM | 2883 | 3HG2 | VAL | A | 208 | 57.475 | 71.536 | 70.189 | 1.00 | 0.78 |
| ATOM | 2884 | C | VAL | A | 208 | 55.574 | 71.063 | 73.908 | 1.00 | 0.78 |
| ATOM | 2885 | O | VAL | A | 208 | 54.352 | 70.989 | 74.043 | 1.00 | 0.78 |
| ATOM | 2886 | N | GLY | A | 209 | 56.430 | 71.334 | 74.891 | 1.00 | 0.87 |
| ATOM | 2887 | H | GLY | A | 209 | 57.410 | 71.314 | 74.667 | 1.00 | 0.87 |
| ATOM | 2888 | CA | GLY | A | 209 | 56.117 | 71.536 | 76.305 | 1.00 | 0.87 |
| ATOM | 2889 | HA1 | GLY | A | 209 | 55.041 | 71.500 | 76.461 | 1.00 | 0.87 |
| ATOM | 2890 | HA2 | GLY | A | 209 | 56.467 | 72.517 | 76.623 | 1.00 | 0.87 |
| ATOM | 2891 | C | GLY | A | 209 | 56.748 | 70.461 | 77.193 | 1.00 | 0.87 |
| ATOM | 2892 | O | GLY | A | 209 | 57.286 | 69.469 | 76.693 | 1.00 | 0.87 |
| ATOM | 2893 | N | GLY | A | 210 | 56.640 | 70.623 | 78.510 | 1.00 | 1.20 |
| ATOM | 2894 | H | GLY | A | 210 | 56.180 | 71.446 | 78.877 | 1.00 | 1.20 |
| ATOM | 2895 | CA | GLY | A | 210 | 57.087 | 69.638 | 79.498 | 1.00 | 1.20 |
| ATOM | 2896 | HA1 | GLY | A | 210 | 56.484 | 68.737 | 79.404 | 1.00 | 1.20 |
| ATOM | 2897 | HA2 | GLY | A | 210 | 56.954 | 70.039 | 80.500 | 1.00 | 1.20 |
| ATOM | 2898 | C | GLY | A | 210 | 58.550 | 69.235 | 79.388 | 1.00 | 1.20 |
| ATOM | 2899 | O | GLY | A | 210 | 58.905 | 68.067 | 79.553 | 1.00 | 1.20 |
| ATOM | 2900 | N | ARG | A | 211 | 59.390 | 70.221 | 79.086 | 1.00 | 1.24 |

| | | | | | | | | | | |
|---|---|---|---|---|---|---|---|---|---|---|
| ATOM | 2901 | H | ARG | A | 211 | 58.980 | 71.141 | 78.956 | 1.00 | 1.24 |
| ATOM | 2902 | CA | ARG | A | 211 | 60.837 | 70.110 | 78.918 | 1.00 | 1.24 |
| ATOM | 2903 | HA | ARG | A | 211 | 61.182 | 69.160 | 79.328 | 1.00 | 1.24 |
| ATOM | 2904 | CB | ARG | A | 211 | 61.132 | 70.155 | 77.390 | 1.00 | 1.24 |
| ATOM | 2905 | HB1 | ARG | A | 211 | 61.327 | 71.188 | 77.096 | 1.00 | 1.24 |
| ATOM | 2906 | HB2 | ARG | A | 211 | 60.231 | 69.843 | 76.857 | 1.00 | 1.24 |
| ATOM | 2907 | CG | ARG | A | 211 | 62.276 | 69.253 | 76.875 | 1.00 | 1.24 |
| ATOM | 2908 | HG1 | ARG | A | 211 | 62.090 | 68.225 | 77.186 | 1.00 | 1.24 |
| ATOM | 2909 | HG2 | ARG | A | 211 | 63.225 | 69.584 | 77.292 | 1.00 | 1.24 |
| ATOM | 2910 | CD | ARG | A | 211 | 62.343 | 69.314 | 75.334 | 1.00 | 1.24 |
| ATOM | 2911 | HD1 | ARG | A | 211 | 62.562 | 70.342 | 75.041 | 1.00 | 1.24 |
| ATOM | 2912 | HD2 | ARG | A | 211 | 61.360 | 69.075 | 74.925 | 1.00 | 1.24 |
| ATOM | 2913 | NE | ARG | A | 211 | 63.373 | 68.435 | 74.722 | 1.00 | 1.24 |
| ATOM | 2914 | HE | ARG | A | 211 | 64.306 | 68.833 | 74.587 | 1.00 | 1.24 |
| ATOM | 2915 | CZ | ARG | A | 211 | 63.189 | 67.318 | 74.036 | 1.00 | 1.24 |
| ATOM | 2916 | NH1 | ARG | A | 211 | 62.044 | 66.696 | 74.052 | 1.00 | 1.24 |
| ATOM | 2917 | 1HH1 | ARG | A | 211 | 61.304 | 67.099 | 74.593 | 1.00 | 1.24 |
| ATOM | 2918 | 2HH1 | ARG | A | 211 | 61.851 | 65.964 | 73.378 | 1.00 | 1.24 |
| ATOM | 2919 | NH2 | ARG | A | 211 | 64.150 | 66.829 | 73.304 | 1.00 | 1.24 |
| ATOM | 2920 | 1HH2 | ARG | A | 211 | 65.020 | 67.377 | 73.293 | 1.00 | 1.24 |
| ATOM | 2921 | 2HH2 | ARG | A | 211 | 64.035 | 66.032 | 72.707 | 1.00 | 1.24 |
| ATOM | 2922 | C | ARG | A | 211 | 61.509 | 71.238 | 79.704 | 1.00 | 1.24 |
| ATOM | 2923 | O | ARG | A | 211 | 60.829 | 72.059 | 80.321 | 1.00 | 1.24 |
| ATOM | 2924 | N | ASP | A | 212 | 62.826 | 71.331 | 79.661 | 1.00 | 1.44 |
| ATOM | 2925 | H | ASP | A | 212 | 63.373 | 70.626 | 79.174 | 1.00 | 1.44 |
| ATOM | 2926 | CA | ASP | A | 212 | 63.521 | 72.596 | 79.899 | 1.00 | 1.44 |
| ATOM | 2927 | HA | ASP | A | 212 | 63.049 | 73.113 | 80.736 | 1.00 | 1.44 |
| ATOM | 2928 | CB | ASP | A | 212 | 64.983 | 72.308 | 80.305 | 1.00 | 1.44 |
| ATOM | 2929 | HB1 | ASP | A | 212 | 64.970 | 71.823 | 81.283 | 1.00 | 1.44 |
| ATOM | 2930 | HB2 | ASP | A | 212 | 65.500 | 73.261 | 80.423 | 1.00 | 1.44 |
| ATOM | 2931 | CG | ASP | A | 212 | 65.792 | 71.414 | 79.347 | 1.00 | 1.44 |
| ATOM | 2932 | OD1 | ASP | A | 212 | 67.030 | 71.389 | 79.499 | 1.00 | 1.44 |
| ATOM | 2933 | OD2 | ASP | A | 212 | 65.175 | 70.726 | 78.496 | 1.00 | 1.44 |
| ATOM | 2934 | C | ASP | A | 212 | 63.435 | 73.547 | 78.684 | 1.00 | 1.44 |
| ATOM | 2935 | O | ASP | A | 212 | 63.116 | 74.721 | 78.862 | 1.00 | 1.44 |
| ATOM | 2936 | N | ASP | A | 213 | 63.650 | 73.049 | 77.457 | 1.00 | 1.38 |
| ATOM | 2937 | H | ASP | A | 213 | 64.034 | 72.106 | 77.415 | 1.00 | 1.38 |
| ATOM | 2938 | CA | ASP | A | 213 | 63.739 | 73.883 | 76.241 | 1.00 | 1.38 |
| ATOM | 2939 | HA | ASP | A | 213 | 64.431 | 74.698 | 76.465 | 1.00 | 1.38 |
| ATOM | 2940 | CB | ASP | A | 213 | 64.402 | 73.061 | 75.121 | 1.00 | 1.38 |
| ATOM | 2941 | HB1 | ASP | A | 213 | 63.837 | 72.141 | 74.982 | 1.00 | 1.38 |
| ATOM | 2942 | HB2 | ASP | A | 213 | 65.404 | 72.780 | 75.449 | 1.00 | 1.38 |
| ATOM | 2943 | CG | ASP | A | 213 | 64.501 | 73.781 | 73.767 | 1.00 | 1.38 |
| ATOM | 2944 | OD1 | ASP | A | 213 | 63.422 | 74.171 | 73.252 | 1.00 | 1.38 |

| | | | | | | | | | | |
|---|---|---|---|---|---|---|---|---|---|---|
| ATOM | 2945 | OD2 | ASP | A | 213 | 65.594 | 73.766 | 73.158 | 1.00 | 1.38 |
| ATOM | 2946 | C | ASP | A | 213 | 62.424 | 74.584 | 75.800 | 1.00 | 1.38 |
| ATOM | 2947 | O | ASP | A | 213 | 62.356 | 75.815 | 75.757 | 1.00 | 1.38 |
| ATOM | 2948 | N | ASN | A | 214 | 61.376 | 73.811 | 75.490 | 1.00 | 0.93 |
| ATOM | 2949 | H | ASN | A | 214 | 61.538 | 72.819 | 75.538 | 1.00 | 0.93 |
| ATOM | 2950 | CA | ASN | A | 214 | 60.020 | 74.259 | 75.104 | 1.00 | 0.93 |
| ATOM | 2951 | HA | ASN | A | 214 | 59.499 | 73.378 | 74.729 | 1.00 | 0.93 |
| ATOM | 2952 | CB | ASN | A | 214 | 59.274 | 74.724 | 76.372 | 1.00 | 0.93 |
| ATOM | 2953 | HB1 | ASN | A | 214 | 58.218 | 74.855 | 76.143 | 1.00 | 0.93 |
| ATOM | 2954 | HB2 | ASN | A | 214 | 59.668 | 75.690 | 76.687 | 1.00 | 0.93 |
| ATOM | 2955 | CG | ASN | A | 214 | 59.363 | 73.752 | 77.520 | 1.00 | 0.93 |
| ATOM | 2956 | OD1 | ASN | A | 214 | 58.747 | 72.698 | 77.521 | 1.00 | 0.93 |
| ATOM | 2957 | ND2 | ASN | A | 214 | 60.177 | 74.053 | 78.496 | 1.00 | 0.93 |
| ATOM | 2958 | 1HD2 | ASN | A | 214 | 60.797 | 74.845 | 78.425 | 1.00 | 0.93 |
| ATOM | 2959 | 2HD2 | ASN | A | 214 | 60.251 | 73.407 | 79.270 | 1.00 | 0.93 |
| ATOM | 2960 | C | ASN | A | 214 | 59.854 | 75.326 | 73.994 | 1.00 | 0.93 |
| ATOM | 2961 | O | ASN | A | 214 | 58.776 | 75.935 | 73.880 | 1.00 | 0.93 |
| ATOM | 2962 | N | THR | A | 215 | 60.897 | 75.559 | 73.195 | 1.00 | 0.99 |
| ATOM | 2963 | H | THR | A | 215 | 61.755 | 75.029 | 73.346 | 1.00 | 0.99 |
| ATOM | 2964 | CA | THR | A | 215 | 61.003 | 76.729 | 72.313 | 1.00 | 0.99 |
| ATOM | 2965 | HA | THR | A | 215 | 60.048 | 77.254 | 72.283 | 1.00 | 0.99 |
| ATOM | 2966 | CB | THR | A | 215 | 62.045 | 77.725 | 72.851 | 1.00 | 0.99 |
| ATOM | 2967 | HB | THR | A | 215 | 63.043 | 77.289 | 72.781 | 1.00 | 0.99 |
| ATOM | 2968 | CG2 | THR | A | 215 | 62.009 | 79.052 | 72.095 | 1.00 | 0.99 |
| ATOM | 2969 | 1HG2 | THR | A | 215 | 62.741 | 79.733 | 72.526 | 1.00 | 0.99 |
| ATOM | 2970 | 2HG2 | THR | A | 215 | 62.254 | 78.893 | 71.044 | 1.00 | 0.99 |
| ATOM | 2971 | 3HG2 | THR | A | 215 | 61.015 | 79.495 | 72.167 | 1.00 | 0.99 |
| ATOM | 2972 | OG1 | THR | A | 215 | 61.781 | 78.040 | 74.203 | 1.00 | 0.99 |
| ATOM | 2973 | HG1 | THR | A | 215 | 61.984 | 77.254 | 74.743 | 1.00 | 0.99 |
| ATOM | 2974 | C | THR | A | 215 | 61.342 | 76.304 | 70.887 | 1.00 | 0.99 |
| ATOM | 2975 | O | THR | A | 215 | 62.490 | 76.025 | 70.523 | 1.00 | 0.99 |
| ATOM | 2976 | N | VAL | A | 216 | 60.299 | 76.192 | 70.075 | 1.00 | 0.43 |
| ATOM | 2977 | H | VAL | A | 216 | 59.383 | 76.422 | 70.445 | 1.00 | 0.43 |
| ATOM | 2978 | CA | VAL | A | 216 | 60.441 | 76.156 | 68.617 | 1.00 | 0.43 |
| ATOM | 2979 | HA | VAL | A | 216 | 61.417 | 75.747 | 68.356 | 1.00 | 0.43 |
| ATOM | 2980 | CB | VAL | A | 216 | 59.376 | 75.202 | 68.019 | 1.00 | 0.43 |
| ATOM | 2981 | HB | VAL | A | 216 | 58.381 | 75.590 | 68.202 | 1.00 | 0.43 |
| ATOM | 2982 | CG1 | VAL | A | 216 | 59.542 | 74.959 | 66.516 | 1.00 | 0.43 |
| ATOM | 2983 | 1HG1 | VAL | A | 216 | 58.882 | 74.157 | 66.189 | 1.00 | 0.43 |
| ATOM | 2984 | 2HG1 | VAL | A | 216 | 59.261 | 75.851 | 65.958 | 1.00 | 0.43 |
| ATOM | 2985 | 3HG1 | VAL | A | 216 | 60.577 | 74.691 | 66.299 | 1.00 | 0.43 |
| ATOM | 2986 | CG2 | VAL | A | 216 | 59.462 | 73.809 | 68.667 | 1.00 | 0.43 |
| ATOM | 2987 | 1HG2 | VAL | A | 216 | 58.747 | 73.126 | 68.214 | 1.00 | 0.43 |
| ATOM | 2988 | 2HG2 | VAL | A | 216 | 60.466 | 73.406 | 68.542 | 1.00 | 0.43 |

| | | | | | | | | | | |
|---|---|---|---|---|---|---|---|---|---|---|
| ATOM | 2989 | 3HG2 | VAL | A | 216 | 59.236 | 73.867 | 69.732 | 1.00 | 0.43 |
| ATOM | 2990 | C | VAL | A | 216 | 60.422 | 77.613 | 68.105 | 1.00 | 0.43 |
| ATOM | 2991 | O | VAL | A | 216 | 60.239 | 78.565 | 68.866 | 1.00 | 0.43 |
| ATOM | 2992 | N | ASP | A | 217 | 60.733 | 77.790 | 66.829 | 1.00 | 0.38 |
| ATOM | 2993 | H | ASP | A | 217 | 60.656 | 76.988 | 66.227 | 1.00 | 0.38 |
| ATOM | 2994 | CA | ASP | A | 217 | 61.373 | 78.975 | 66.244 | 1.00 | 0.38 |
| ATOM | 2995 | HA | ASP | A | 217 | 61.120 | 79.878 | 66.797 | 1.00 | 0.38 |
| ATOM | 2996 | CB | ASP | A | 217 | 62.880 | 78.767 | 66.322 | 1.00 | 0.38 |
| ATOM | 2997 | HB1 | ASP | A | 217 | 63.058 | 77.897 | 65.694 | 1.00 | 0.38 |
| ATOM | 2998 | HB2 | ASP | A | 217 | 63.165 | 78.548 | 67.351 | 1.00 | 0.38 |
| ATOM | 2999 | CG | ASP | A | 217 | 63.710 | 79.951 | 65.801 | 1.00 | 0.38 |
| ATOM | 3000 | OD1 | ASP | A | 217 | 63.314 | 81.106 | 66.050 | 1.00 | 0.38 |
| ATOM | 3001 | OD2 | ASP | A | 217 | 64.611 | 79.737 | 64.960 | 1.00 | 0.38 |
| ATOM | 3002 | C | ASP | A | 217 | 60.922 | 79.103 | 64.768 | 1.00 | 0.38 |
| ATOM | 3003 | O | ASP | A | 217 | 59.979 | 78.458 | 64.319 | 1.00 | 0.38 |
| ATOM | 3004 | N | THR | A | 218 | 61.633 | 79.875 | 63.949 | 1.00 | 0.43 |
| ATOM | 3005 | H | THR | A | 218 | 62.271 | 80.540 | 64.377 | 1.00 | 0.43 |
| ATOM | 3006 | CA | THR | A | 218 | 61.998 | 79.475 | 62.576 | 1.00 | 0.43 |
| ATOM | 3007 | HA | THR | A | 218 | 61.246 | 79.882 | 61.907 | 1.00 | 0.43 |
| ATOM | 3008 | CB | THR | A | 218 | 63.361 | 80.080 | 62.166 | 1.00 | 0.43 |
| ATOM | 3009 | HB | THR | A | 218 | 64.169 | 79.416 | 62.463 | 1.00 | 0.43 |
| ATOM | 3010 | CG2 | THR | A | 218 | 63.454 | 80.286 | 60.656 | 1.00 | 0.43 |
| ATOM | 3011 | 1HG2 | THR | A | 218 | 64.432 | 80.696 | 60.408 | 1.00 | 0.43 |
| ATOM | 3012 | 2HG2 | THR | A | 218 | 63.324 | 79.335 | 60.140 | 1.00 | 0.43 |
| ATOM | 3013 | 3HG2 | THR | A | 218 | 62.682 | 80.984 | 60.331 | 1.00 | 0.43 |
| ATOM | 3014 | OG1 | THR | A | 218 | 63.612 | 81.322 | 62.781 | 1.00 | 0.43 |
| ATOM | 3015 | HG1 | THR | A | 218 | 64.023 | 81.117 | 63.643 | 1.00 | 0.43 |
| ATOM | 3016 | C | THR | A | 218 | 62.083 | 77.960 | 62.362 | 1.00 | 0.43 |
| ATOM | 3017 | O | THR | A | 218 | 63.030 | 77.305 | 62.798 | 1.00 | 0.43 |
| ATOM | 3018 | N | VAL | A | 219 | 61.142 | 77.406 | 61.610 | 1.00 | 0.37 |
| ATOM | 3019 | H | VAL | A | 219 | 60.374 | 78.000 | 61.307 | 1.00 | 0.37 |
| ATOM | 3020 | CA | VAL | A | 219 | 61.211 | 76.038 | 61.071 | 1.00 | 0.37 |
| ATOM | 3021 | HA | VAL | A | 219 | 62.249 | 75.738 | 61.017 | 1.00 | 0.37 |
| ATOM | 3022 | CB | VAL | A | 219 | 60.452 | 75.022 | 61.958 | 1.00 | 0.37 |
| ATOM | 3023 | HB | VAL | A | 219 | 59.402 | 75.304 | 61.979 | 1.00 | 0.37 |
| ATOM | 3024 | CG1 | VAL | A | 219 | 60.535 | 73.591 | 61.401 | 1.00 | 0.37 |
| ATOM | 3025 | 1HG1 | VAL | A | 219 | 60.068 | 72.889 | 62.091 | 1.00 | 0.37 |
| ATOM | 3026 | 2HG1 | VAL | A | 219 | 60.003 | 73.525 | 60.453 | 1.00 | 0.37 |
| ATOM | 3027 | 3HG1 | VAL | A | 219 | 61.574 | 73.303 | 61.251 | 1.00 | 0.37 |
| ATOM | 3028 | CG2 | VAL | A | 219 | 60.977 | 74.983 | 63.396 | 1.00 | 0.37 |
| ATOM | 3029 | 1HG2 | VAL | A | 219 | 60.476 | 74.195 | 63.955 | 1.00 | 0.37 |
| ATOM | 3030 | 2HG2 | VAL | A | 219 | 62.052 | 74.812 | 63.411 | 1.00 | 0.37 |
| ATOM | 3031 | 3HG2 | VAL | A | 219 | 60.769 | 75.932 | 63.888 | 1.00 | 0.37 |
| ATOM | 3032 | C | VAL | A | 219 | 60.608 | 76.089 | 59.672 | 1.00 | 0.37 |

| | | | | | | | | | | |
|---|---|---|---|---|---|---|---|---|---|---|
| ATOM | 3033 | O | VAL | A | 219 | 59.693 | 76.858 | 59.397 | 1.00 | 0.37 |
| ATOM | 3034 | N | THR | A | 220 | 61.142 | 75.307 | 58.745 | 1.00 | 0.36 |
| ATOM | 3035 | H | THR | A | 220 | 61.959 | 74.768 | 59.010 | 1.00 | 0.36 |
| ATOM | 3036 | CA | THR | A | 220 | 60.817 | 75.404 | 57.323 | 1.00 | 0.36 |
| ATOM | 3037 | HA | THR | A | 220 | 59.794 | 75.758 | 57.214 | 1.00 | 0.36 |
| ATOM | 3038 | CB | THR | A | 220 | 61.726 | 76.444 | 56.650 | 1.00 | 0.36 |
| ATOM | 3039 | HB | THR | A | 220 | 62.753 | 76.271 | 56.965 | 1.00 | 0.36 |
| ATOM | 3040 | CG2 | THR | A | 220 | 61.674 | 76.477 | 55.122 | 1.00 | 0.36 |
| ATOM | 3041 | 1HG2 | THR | A | 220 | 62.157 | 77.385 | 54.762 | 1.00 | 0.36 |
| ATOM | 3042 | 2HG2 | THR | A | 220 | 62.213 | 75.621 | 54.719 | 1.00 | 0.36 |
| ATOM | 3043 | 3HG2 | THR | A | 220 | 60.645 | 76.451 | 54.771 | 1.00 | 0.36 |
| ATOM | 3044 | OG1 | THR | A | 220 | 61.338 | 77.724 | 57.091 | 1.00 | 0.36 |
| ATOM | 3045 | HG1 | THR | A | 220 | 60.861 | 77.631 | 57.930 | 1.00 | 0.36 |
| ATOM | 3046 | C | THR | A | 220 | 60.872 | 74.029 | 56.668 | 1.00 | 0.36 |
| ATOM | 3047 | O | THR | A | 220 | 61.920 | 73.552 | 56.231 | 1.00 | 0.36 |
| ATOM | 3048 | N | PHE | A | 221 | 59.707 | 73.383 | 56.639 | 1.00 | 0.38 |
| ATOM | 3049 | H | PHE | A | 221 | 58.889 | 73.866 | 57.002 | 1.00 | 0.38 |
| ATOM | 3050 | CA | PHE | A | 221 | 59.452 | 72.207 | 55.820 | 1.00 | 0.38 |
| ATOM | 3051 | HA | PHE | A | 221 | 60.341 | 71.582 | 55.785 | 1.00 | 0.38 |
| ATOM | 3052 | CB | PHE | A | 221 | 58.298 | 71.401 | 56.426 | 1.00 | 0.38 |
| ATOM | 3053 | HB1 | PHE | A | 221 | 58.132 | 70.517 | 55.810 | 1.00 | 0.38 |
| ATOM | 3054 | HB2 | PHE | A | 221 | 57.387 | 71.999 | 56.380 | 1.00 | 0.38 |
| ATOM | 3055 | CG | PHE | A | 221 | 58.512 | 70.964 | 57.862 | 1.00 | 0.38 |
| ATOM | 3056 | CD1 | PHE | A | 221 | 59.190 | 69.765 | 58.151 | 1.00 | 0.38 |
| ATOM | 3057 | HD1 | PHE | A | 221 | 59.578 | 69.155 | 57.348 | 1.00 | 0.38 |
| ATOM | 3058 | CE1 | PHE | A | 221 | 59.359 | 69.357 | 59.486 | 1.00 | 0.38 |
| ATOM | 3059 | HE1 | PHE | A | 221 | 59.887 | 68.444 | 59.705 | 1.00 | 0.38 |
| ATOM | 3060 | CZ | PHE | A | 221 | 58.841 | 70.135 | 60.538 | 1.00 | 0.38 |
| ATOM | 3061 | HZ | PHE | A | 221 | 58.967 | 69.818 | 61.564 | 1.00 | 0.38 |
| ATOM | 3062 | CE2 | PHE | A | 221 | 58.172 | 71.335 | 60.246 | 1.00 | 0.38 |
| ATOM | 3063 | HE2 | PHE | A | 221 | 57.769 | 71.946 | 61.041 | 1.00 | 0.38 |
| ATOM | 3064 | CD2 | PHE | A | 221 | 58.021 | 71.755 | 58.916 | 1.00 | 0.38 |
| ATOM | 3065 | HD2 | PHE | A | 221 | 57.501 | 72.680 | 58.700 | 1.00 | 0.38 |
| ATOM | 3066 | C | PHE | A | 221 | 59.067 | 72.645 | 54.397 | 1.00 | 0.38 |
| ATOM | 3067 | O | PHE | A | 221 | 58.306 | 73.601 | 54.220 | 1.00 | 0.38 |
| ATOM | 3068 | N | LYS | A | 222 | 59.601 | 71.959 | 53.382 | 1.00 | 0.41 |
| ATOM | 3069 | H | LYS | A | 222 | 60.211 | 71.180 | 53.607 | 1.00 | 0.41 |
| ATOM | 3070 | CA | LYS | A | 222 | 59.658 | 72.452 | 52.002 | 1.00 | 0.41 |
| ATOM | 3071 | HA | LYS | A | 222 | 58.748 | 72.992 | 51.773 | 1.00 | 0.41 |
| ATOM | 3072 | CB | LYS | A | 222 | 60.883 | 73.405 | 51.909 | 1.00 | 0.41 |
| ATOM | 3073 | HB1 | LYS | A | 222 | 61.449 | 73.225 | 50.998 | 1.00 | 0.41 |
| ATOM | 3074 | HB2 | LYS | A | 222 | 61.576 | 73.171 | 52.721 | 1.00 | 0.41 |
| ATOM | 3075 | CG | LYS | A | 222 | 60.547 | 74.905 | 51.997 | 1.00 | 0.41 |
| ATOM | 3076 | HG1 | LYS | A | 222 | 61.370 | 75.395 | 52.515 | 1.00 | 0.41 |

| | | | | | | | | | | |
|---|---|---|---|---|---|---|---|---|---|---|
| ATOM | 3077 | HG2 | LYS | A | 222 | 59.667 | 75.039 | 52.622 | 1.00 | 0.41 |
| ATOM | 3078 | CD | LYS | A | 222 | 60.347 | 75.672 | 50.674 | 1.00 | 0.41 |
| ATOM | 3079 | HD1 | LYS | A | 222 | 61.309 | 76.080 | 50.360 | 1.00 | 0.41 |
| ATOM | 3080 | HD2 | LYS | A | 222 | 59.702 | 76.526 | 50.888 | 1.00 | 0.41 |
| ATOM | 3081 | CE | LYS | A | 222 | 59.727 | 74.891 | 49.506 | 1.00 | 0.41 |
| ATOM | 3082 | HE1 | LYS | A | 222 | 59.123 | 75.588 | 48.917 | 1.00 | 0.41 |
| ATOM | 3083 | HE2 | LYS | A | 222 | 59.049 | 74.130 | 49.901 | 1.00 | 0.41 |
| ATOM | 3084 | NZ | LYS | A | 222 | 60.771 | 74.289 | 48.636 | 1.00 | 0.41 |
| ATOM | 3085 | HZ1 | LYS | A | 222 | 61.346 | 73.585 | 49.101 | 1.00 | 0.41 |
| ATOM | 3086 | HZ2 | LYS | A | 222 | 60.394 | 73.732 | 47.863 | 1.00 | 0.41 |
| ATOM | 3087 | HZ3 | LYS | A | 222 | 61.391 | 74.967 | 48.227 | 1.00 | 0.41 |
| ATOM | 3088 | C | LYS | A | 222 | 59.873 | 71.322 | 50.975 | 1.00 | 0.41 |
| ATOM | 3089 | O | LYS | A | 222 | 60.675 | 70.399 | 51.157 | 1.00 | 0.41 |
| ATOM | 3090 | N | ASP | A | 223 | 59.216 | 71.467 | 49.828 | 1.00 | 0.67 |
| ATOM | 3091 | H | ASP | A | 223 | 58.479 | 72.161 | 49.810 | 1.00 | 0.67 |
| ATOM | 3092 | CA | ASP | A | 223 | 59.227 | 70.450 | 48.758 | 1.00 | 0.67 |
| ATOM | 3093 | HA | ASP | A | 223 | 58.666 | 70.873 | 47.923 | 1.00 | 0.67 |
| ATOM | 3094 | CB | ASP | A | 223 | 60.631 | 70.156 | 48.191 | 1.00 | 0.67 |
| ATOM | 3095 | HB1 | ASP | A | 223 | 60.528 | 69.631 | 47.241 | 1.00 | 0.67 |
| ATOM | 3096 | HB2 | ASP | A | 223 | 61.179 | 69.509 | 48.866 | 1.00 | 0.67 |
| ATOM | 3097 | CG | ASP | A | 223 | 61.397 | 71.462 | 47.975 | 1.00 | 0.67 |
| ATOM | 3098 | OD1 | ASP | A | 223 | 61.028 | 72.194 | 47.030 | 1.00 | 0.67 |
| ATOM | 3099 | OD2 | ASP | A | 223 | 62.171 | 71.881 | 48.876 | 1.00 | 0.67 |
| ATOM | 3100 | C | ASP | A | 223 | 58.395 | 69.241 | 49.236 | 1.00 | 0.67 |
| ATOM | 3101 | O | ASP | A | 223 | 57.226 | 69.508 | 49.492 | 1.00 | 0.67 |
| ATOM | 3102 | N | SER | A | 224 | 58.900 | 68.014 | 49.486 | 1.00 | 0.78 |
| ATOM | 3103 | H | SER | A | 224 | 59.825 | 67.785 | 49.130 | 1.00 | 0.78 |
| ATOM | 3104 | CA | SER | A | 224 | 58.518 | 67.205 | 50.673 | 1.00 | 0.78 |
| ATOM | 3105 | HA | SER | A | 224 | 59.457 | 67.074 | 51.210 | 1.00 | 0.78 |
| ATOM | 3106 | CB | SER | A | 224 | 57.568 | 67.823 | 51.728 | 1.00 | 0.78 |
| ATOM | 3107 | HB1 | SER | A | 224 | 57.580 | 67.187 | 52.614 | 1.00 | 0.78 |
| ATOM | 3108 | HB2 | SER | A | 224 | 56.551 | 67.809 | 51.340 | 1.00 | 0.78 |
| ATOM | 3109 | OG | SER | A | 224 | 57.897 | 69.139 | 52.126 | 1.00 | 0.78 |
| ATOM | 3110 | HG | SER | A | 224 | 57.642 | 69.677 | 51.363 | 1.00 | 0.78 |
| ATOM | 3111 | C | SER | A | 224 | 58.002 | 65.769 | 50.477 | 1.00 | 0.78 |
| ATOM | 3112 | O | SER | A | 224 | 58.521 | 64.836 | 51.102 | 1.00 | 0.78 |
| ATOM | 3113 | N | THR | A | 225 | 56.909 | 65.650 | 49.723 | 1.00 | 0.57 |
| ATOM | 3114 | H | THR | A | 225 | 56.567 | 66.514 | 49.313 | 1.00 | 0.57 |
| ATOM | 3115 | CA | THR | A | 225 | 55.894 | 64.575 | 49.753 | 1.00 | 0.57 |
| ATOM | 3116 | HA | THR | A | 225 | 55.099 | 65.128 | 49.262 | 1.00 | 0.57 |
| ATOM | 3117 | CB | THR | A | 225 | 56.003 | 63.489 | 48.640 | 1.00 | 0.57 |
| ATOM | 3118 | HB | THR | A | 225 | 55.732 | 63.979 | 47.703 | 1.00 | 0.57 |
| ATOM | 3119 | CG2 | THR | A | 225 | 57.391 | 62.906 | 48.415 | 1.00 | 0.57 |
| ATOM | 3120 | 1HG2 | THR | A | 225 | 57.581 | 62.161 | 49.176 | 1.00 | 0.57 |

| | | | | | | | | | | |
|---|---|---|---|---|---|---|---|---|---|---|
| ATOM | 3121 | 2HG2 | THR | A | 225 | 57.410 | 62.412 | 47.444 | 1.00 | 0.57 |
| ATOM | 3122 | 3HG2 | THR | A | 225 | 58.153 | 63.685 | 48.430 | 1.00 | 0.57 |
| ATOM | 3123 | OG1 | THR | A | 225 | 55.144 | 62.379 | 48.787 | 1.00 | 0.57 |
| ATOM | 3124 | HG1 | THR | A | 225 | 54.307 | 62.551 | 48.293 | 1.00 | 0.57 |
| ATOM | 3125 | C | THR | A | 225 | 55.062 | 64.380 | 51.038 | 1.00 | 0.57 |
| ATOM | 3126 | O | THR | A | 225 | 54.507 | 65.350 | 51.578 | 1.00 | 0.57 |
| ATOM | 3127 | N | VAL | A | 226 | 54.896 | 63.127 | 51.440 | 1.00 | 0.42 |
| ATOM | 3128 | H | VAL | A | 226 | 55.294 | 62.466 | 50.783 | 1.00 | 0.42 |
| ATOM | 3129 | CA | VAL | A | 226 | 54.186 | 62.548 | 52.600 | 1.00 | 0.42 |
| ATOM | 3130 | HA | VAL | A | 226 | 54.903 | 61.872 | 53.049 | 1.00 | 0.42 |
| ATOM | 3131 | CB | VAL | A | 226 | 53.758 | 63.470 | 53.752 | 1.00 | 0.42 |
| ATOM | 3132 | HB | VAL | A | 226 | 53.013 | 64.145 | 53.367 | 1.00 | 0.42 |
| ATOM | 3133 | CG1 | VAL | A | 226 | 53.124 | 62.645 | 54.886 | 1.00 | 0.42 |
| ATOM | 3134 | 1HG1 | VAL | A | 226 | 52.953 | 63.275 | 55.757 | 1.00 | 0.42 |
| ATOM | 3135 | 2HG1 | VAL | A | 226 | 52.158 | 62.246 | 54.576 | 1.00 | 0.42 |
| ATOM | 3136 | 3HG1 | VAL | A | 226 | 53.778 | 61.824 | 55.176 | 1.00 | 0.42 |
| ATOM | 3137 | CG2 | VAL | A | 226 | 54.909 | 64.240 | 54.425 | 1.00 | 0.42 |
| ATOM | 3138 | 1HG2 | VAL | A | 226 | 55.481 | 63.578 | 55.066 | 1.00 | 0.42 |
| ATOM | 3139 | 2HG2 | VAL | A | 226 | 55.584 | 64.676 | 53.692 | 1.00 | 0.42 |
| ATOM | 3140 | 3HG2 | VAL | A | 226 | 54.496 | 65.046 | 55.033 | 1.00 | 0.42 |
| ATOM | 3141 | C | VAL | A | 226 | 53.039 | 61.668 | 52.109 | 1.00 | 0.42 |
| ATOM | 3142 | O | VAL | A | 226 | 51.888 | 62.078 | 51.912 | 1.00 | 0.42 |
| ATOM | 3143 | N | SER | A | 227 | 53.417 | 60.399 | 51.964 | 1.00 | 0.77 |
| ATOM | 3144 | H | SER | A | 227 | 54.394 | 60.191 | 52.081 | 1.00 | 0.77 |
| ATOM | 3145 | CA | SER | A | 227 | 52.561 | 59.348 | 51.453 | 1.00 | 0.77 |
| ATOM | 3146 | HA | SER | A | 227 | 51.613 | 59.791 | 51.163 | 1.00 | 0.77 |
| ATOM | 3147 | CB | SER | A | 227 | 53.203 | 58.775 | 50.178 | 1.00 | 0.77 |
| ATOM | 3148 | HB1 | SER | A | 227 | 53.782 | 57.883 | 50.419 | 1.00 | 0.77 |
| ATOM | 3149 | HB2 | SER | A | 227 | 53.877 | 59.515 | 49.739 | 1.00 | 0.77 |
| ATOM | 3150 | OG | SER | A | 227 | 52.219 | 58.470 | 49.210 | 1.00 | 0.77 |
| ATOM | 3151 | HG | SER | A | 227 | 52.241 | 59.232 | 48.571 | 1.00 | 0.77 |
| ATOM | 3152 | C | SER | A | 227 | 52.271 | 58.275 | 52.493 | 1.00 | 0.77 |
| ATOM | 3153 | O | SER | A | 227 | 53.114 | 57.938 | 53.337 | 1.00 | 0.77 |
| ATOM | 3154 | N | ASN | A | 228 | 51.067 | 57.710 | 52.408 | 1.00 | 0.95 |
| ATOM | 3155 | H | ASN | A | 228 | 50.396 | 58.131 | 51.772 | 1.00 | 0.95 |
| ATOM | 3156 | CA | ASN | A | 228 | 50.681 | 56.508 | 53.151 | 1.00 | 0.95 |
| ATOM | 3157 | HA | ASN | A | 228 | 49.596 | 56.406 | 53.092 | 1.00 | 0.95 |
| ATOM | 3158 | CB | ASN | A | 228 | 51.313 | 55.290 | 52.443 | 1.00 | 0.95 |
| ATOM | 3159 | HB1 | ASN | A | 228 | 51.121 | 54.401 | 53.044 | 1.00 | 0.95 |
| ATOM | 3160 | HB2 | ASN | A | 228 | 52.392 | 55.413 | 52.374 | 1.00 | 0.95 |
| ATOM | 3161 | CG | ASN | A | 228 | 50.753 | 55.032 | 51.048 | 1.00 | 0.95 |
| ATOM | 3162 | OD1 | ASN | A | 228 | 50.105 | 54.030 | 50.810 | 1.00 | 0.95 |
| ATOM | 3163 | ND2 | ASN | A | 228 | 50.966 | 55.877 | 50.060 | 1.00 | 0.95 |
| ATOM | 3164 | 1HD2 | ASN | A | 228 | 51.479 | 56.742 | 50.160 | 1.00 | 0.95 |

| | | | | | | | | | | |
|---|---|---|---|---|---|---|---|---|---|---|
| ATOM | 3165 | 2HD2 | ASN | A | 228 | 50.536 | 55.646 | 49.186 | 1.00 | 0.95 |
| ATOM | 3166 | C | ASN | A | 228 | 51.012 | 56.650 | 54.641 | 1.00 | 0.95 |
| ATOM | 3167 | O | ASN | A | 228 | 51.734 | 55.846 | 55.209 | 1.00 | 0.95 |
| ATOM | 3168 | N | SER | A | 229 | 50.595 | 57.772 | 55.221 | 1.00 | 0.86 |
| ATOM | 3169 | H | SER | A | 229 | 49.963 | 58.358 | 54.697 | 1.00 | 0.86 |
| ATOM | 3170 | CA | SER | A | 229 | 50.997 | 58.249 | 56.545 | 1.00 | 0.86 |
| ATOM | 3171 | HA | SER | A | 229 | 51.570 | 57.477 | 57.061 | 1.00 | 0.86 |
| ATOM | 3172 | CB | SER | A | 229 | 51.863 | 59.511 | 56.395 | 1.00 | 0.86 |
| ATOM | 3173 | HB1 | SER | A | 229 | 51.996 | 59.994 | 57.364 | 1.00 | 0.86 |
| ATOM | 3174 | HB2 | SER | A | 229 | 51.358 | 60.218 | 55.734 | 1.00 | 0.86 |
| ATOM | 3175 | OG | SER | A | 229 | 53.138 | 59.195 | 55.860 | 1.00 | 0.86 |
| ATOM | 3176 | HG | SER | A | 229 | 53.003 | 58.586 | 55.118 | 1.00 | 0.86 |
| ATOM | 3177 | C | SER | A | 229 | 49.758 | 58.565 | 57.372 | 1.00 | 0.86 |
| ATOM | 3178 | O | SER | A | 229 | 48.709 | 58.886 | 56.834 | 1.00 | 0.86 |
| ATOM | 3179 | N | VAL | A | 230 | 49.866 | 58.517 | 58.688 | 1.00 | 0.95 |
| ATOM | 3180 | H | VAL | A | 230 | 50.769 | 58.226 | 59.052 | 1.00 | 0.95 |
| ATOM | 3181 | CA | VAL | A | 230 | 48.777 | 58.813 | 59.636 | 1.00 | 0.95 |
| ATOM | 3182 | HA | VAL | A | 230 | 47.882 | 58.245 | 59.381 | 1.00 | 0.95 |
| ATOM | 3183 | CB | VAL | A | 230 | 49.277 | 58.354 | 61.033 | 1.00 | 0.95 |
| ATOM | 3184 | HB | VAL | A | 230 | 50.150 | 58.950 | 61.277 | 1.00 | 0.95 |
| ATOM | 3185 | CG1 | VAL | A | 230 | 48.299 | 58.531 | 62.195 | 1.00 | 0.95 |
| ATOM | 3186 | 1HG1 | VAL | A | 230 | 48.809 | 58.359 | 63.141 | 1.00 | 0.95 |
| ATOM | 3187 | 2HG1 | VAL | A | 230 | 47.874 | 59.534 | 62.202 | 1.00 | 0.95 |
| ATOM | 3188 | 3HG1 | VAL | A | 230 | 47.495 | 57.802 | 62.109 | 1.00 | 0.95 |
| ATOM | 3189 | CG2 | VAL | A | 230 | 49.728 | 56.883 | 61.049 | 1.00 | 0.95 |
| ATOM | 3190 | 1HG2 | VAL | A | 230 | 50.016 | 56.594 | 62.059 | 1.00 | 0.95 |
| ATOM | 3191 | 2HG2 | VAL | A | 230 | 48.904 | 56.245 | 60.724 | 1.00 | 0.95 |
| ATOM | 3192 | 3HG2 | VAL | A | 230 | 50.588 | 56.720 | 60.399 | 1.00 | 0.95 |
| ATOM | 3193 | C | VAL | A | 230 | 48.419 | 60.303 | 59.648 | 1.00 | 0.95 |
| ATOM | 3194 | O | VAL | A | 230 | 47.265 | 60.688 | 59.785 | 1.00 | 0.95 |
| ATOM | 3195 | N | ASN | A | 231 | 49.392 | 61.180 | 59.419 | 1.00 | 0.46 |
| ATOM | 3196 | H | ASN | A | 231 | 50.323 | 60.856 | 59.191 | 1.00 | 0.46 |
| ATOM | 3197 | CA | ASN | A | 231 | 49.196 | 62.623 | 59.396 | 1.00 | 0.46 |
| ATOM | 3198 | HA | ASN | A | 231 | 48.144 | 62.799 | 59.196 | 1.00 | 0.46 |
| ATOM | 3199 | CB | ASN | A | 231 | 49.529 | 63.305 | 60.758 | 1.00 | 0.46 |
| ATOM | 3200 | HB1 | ASN | A | 231 | 48.913 | 64.199 | 60.849 | 1.00 | 0.46 |
| ATOM | 3201 | HB2 | ASN | A | 231 | 50.565 | 63.638 | 60.747 | 1.00 | 0.46 |
| ATOM | 3202 | CG | ASN | A | 231 | 49.336 | 62.475 | 62.029 | 1.00 | 0.46 |
| ATOM | 3203 | OD1 | ASN | A | 231 | 48.618 | 62.817 | 62.950 | 1.00 | 0.46 |
| ATOM | 3204 | ND2 | ASN | A | 231 | 50.082 | 61.416 | 62.213 | 1.00 | 0.46 |
| ATOM | 3205 | 1HD2 | ASN | A | 231 | 50.722 | 61.169 | 61.470 | 1.00 | 0.46 |
| ATOM | 3206 | 2HD2 | ASN | A | 231 | 49.877 | 60.828 | 62.999 | 1.00 | 0.46 |
| ATOM | 3207 | C | ASN | A | 231 | 49.984 | 63.225 | 58.215 | 1.00 | 0.46 |
| ATOM | 3208 | O | ASN | A | 231 | 50.838 | 62.544 | 57.634 | 1.00 | 0.46 |

| | | | | | | | | | | |
|---|---|---|---|---|---|---|---|---|---|---|
| ATOM | 3209 | N | GLY | A | 232 | 49.734 | 64.492 | 57.860 | 1.00 | 0.42 |
| ATOM | 3210 | H | GLY | A | 232 | 48.941 | 64.984 | 58.260 | 1.00 | 0.42 |
| ATOM | 3211 | CA | GLY | A | 232 | 50.584 | 65.162 | 56.877 | 1.00 | 0.42 |
| ATOM | 3212 | HA1 | GLY | A | 232 | 49.943 | 65.651 | 56.144 | 1.00 | 0.42 |
| ATOM | 3213 | HA2 | GLY | A | 232 | 51.207 | 64.458 | 56.331 | 1.00 | 0.42 |
| ATOM | 3214 | C | GLY | A | 232 | 51.479 | 66.220 | 57.486 | 1.00 | 0.42 |
| ATOM | 3215 | O | GLY | A | 232 | 52.659 | 65.997 | 57.769 | 1.00 | 0.42 |
| ATOM | 3216 | N | ILE | A | 233 | 50.865 | 67.365 | 57.742 | 1.00 | 0.35 |
| ATOM | 3217 | H | ILE | A | 233 | 49.874 | 67.406 | 57.522 | 1.00 | 0.35 |
| ATOM | 3218 | CA | ILE | A | 233 | 51.419 | 68.421 | 58.592 | 1.00 | 0.35 |
| ATOM | 3219 | HA | ILE | A | 233 | 52.511 | 68.410 | 58.597 | 1.00 | 0.35 |
| ATOM | 3220 | CB | ILE | A | 233 | 50.888 | 69.790 | 58.117 | 1.00 | 0.35 |
| ATOM | 3221 | HB | ILE | A | 233 | 49.806 | 69.695 | 58.030 | 1.00 | 0.35 |
| ATOM | 3222 | CG2 | ILE | A | 233 | 51.176 | 70.927 | 59.124 | 1.00 | 0.35 |
| ATOM | 3223 | 1HG2 | ILE | A | 233 | 50.815 | 71.876 | 58.739 | 1.00 | 0.35 |
| ATOM | 3224 | 2HG2 | ILE | A | 233 | 50.667 | 70.751 | 60.072 | 1.00 | 0.35 |
| ATOM | 3225 | 3HG2 | ILE | A | 233 | 52.245 | 71.006 | 59.300 | 1.00 | 0.35 |
| ATOM | 3226 | CG1 | ILE | A | 233 | 51.434 | 70.116 | 56.717 | 1.00 | 0.35 |
| ATOM | 3227 | 1HG1 | ILE | A | 233 | 51.307 | 69.254 | 56.065 | 1.00 | 0.35 |
| ATOM | 3228 | 2HG1 | ILE | A | 233 | 52.499 | 70.338 | 56.772 | 1.00 | 0.35 |
| ATOM | 3229 | CD1 | ILE | A | 233 | 50.694 | 71.285 | 56.065 | 1.00 | 0.35 |
| ATOM | 3230 | HD1 | ILE | A | 233 | 51.035 | 72.226 | 56.490 | 1.00 | 0.35 |
| ATOM | 3231 | HD2 | ILE | A | 233 | 50.901 | 71.272 | 55.002 | 1.00 | 0.35 |
| ATOM | 3232 | HD3 | ILE | A | 233 | 49.620 | 71.184 | 56.203 | 1.00 | 0.35 |
| ATOM | 3233 | C | ILE | A | 233 | 50.883 | 68.177 | 59.993 | 1.00 | 0.35 |
| ATOM | 3234 | O | ILE | A | 233 | 49.676 | 67.957 | 60.166 | 1.00 | 0.35 |
| ATOM | 3235 | N | ARG | A | 234 | 51.742 | 68.290 | 61.006 | 1.00 | 0.27 |
| ATOM | 3236 | H | ARG | A | 234 | 52.732 | 68.426 | 60.825 | 1.00 | 0.27 |
| ATOM | 3237 | CA | ARG | A | 234 | 51.274 | 68.213 | 62.388 | 1.00 | 0.27 |
| ATOM | 3238 | HA | ARG | A | 234 | 50.236 | 68.506 | 62.419 | 1.00 | 0.27 |
| ATOM | 3239 | CB | ARG | A | 234 | 51.331 | 66.735 | 62.809 | 1.00 | 0.27 |
| ATOM | 3240 | HB1 | ARG | A | 234 | 52.342 | 66.357 | 62.669 | 1.00 | 0.27 |
| ATOM | 3241 | HB2 | ARG | A | 234 | 50.666 | 66.166 | 62.155 | 1.00 | 0.27 |
| ATOM | 3242 | CG | ARG | A | 234 | 50.895 | 66.505 | 64.260 | 1.00 | 0.27 |
| ATOM | 3243 | HG1 | ARG | A | 234 | 49.991 | 67.083 | 64.460 | 1.00 | 0.27 |
| ATOM | 3244 | HG2 | ARG | A | 234 | 51.691 | 66.860 | 64.909 | 1.00 | 0.27 |
| ATOM | 3245 | CD | ARG | A | 234 | 50.596 | 65.034 | 64.562 | 1.00 | 0.27 |
| ATOM | 3246 | HD1 | ARG | A | 234 | 49.884 | 64.671 | 63.823 | 1.00 | 0.27 |
| ATOM | 3247 | HD2 | ARG | A | 234 | 50.115 | 64.984 | 65.543 | 1.00 | 0.27 |
| ATOM | 3248 | NE | ARG | A | 234 | 51.812 | 64.191 | 64.567 | 1.00 | 0.27 |
| ATOM | 3249 | HE | ARG | A | 234 | 52.688 | 64.626 | 64.296 | 1.00 | 0.27 |
| ATOM | 3250 | CZ | ARG | A | 234 | 51.872 | 62.922 | 64.937 | 1.00 | 0.27 |
| ATOM | 3251 | NH1 | ARG | A | 234 | 50.821 | 62.180 | 65.120 | 1.00 | 0.27 |
| ATOM | 3252 | 1HH1 | ARG | A | 234 | 49.930 | 62.541 | 64.805 | 1.00 | 0.27 |

| | | | | | | | | | | |
|---|---|---|---|---|---|---|---|---|---|---|
| ATOM | 3253 | 2HH1 | ARG | A | 234 | 50.963 | 61.193 | 65.287 | 1.00 | 0.27 |
| ATOM | 3254 | NH2 | ARG | A | 234 | 53.013 | 62.352 | 65.168 | 1.00 | 0.27 |
| ATOM | 3255 | 1HH2 | ARG | A | 234 | 53.897 | 62.799 | 64.908 | 1.00 | 0.27 |
| ATOM | 3256 | 2HH2 | ARG | A | 234 | 53.037 | 61.342 | 65.264 | 1.00 | 0.27 |
| ATOM | 3257 | C | ARG | A | 234 | 52.034 | 69.152 | 63.319 | 1.00 | 0.27 |
| ATOM | 3258 | O | ARG | A | 234 | 53.231 | 69.362 | 63.154 | 1.00 | 0.27 |
| ATOM | 3259 | N | ILE | A | 235 | 51.338 | 69.721 | 64.301 | 1.00 | 0.35 |
| ATOM | 3260 | H | ILE | A | 235 | 50.346 | 69.511 | 64.352 | 1.00 | 0.35 |
| ATOM | 3261 | CA | ILE | A | 235 | 51.871 | 70.742 | 65.211 | 1.00 | 0.35 |
| ATOM | 3262 | HA | ILE | A | 235 | 52.947 | 70.600 | 65.312 | 1.00 | 0.35 |
| ATOM | 3263 | CB | ILE | A | 235 | 51.661 | 72.182 | 64.661 | 1.00 | 0.35 |
| ATOM | 3264 | HB | ILE | A | 235 | 50.595 | 72.403 | 64.690 | 1.00 | 0.35 |
| ATOM | 3265 | CG2 | ILE | A | 235 | 52.391 | 73.198 | 65.564 | 1.00 | 0.35 |
| ATOM | 3266 | 1HG2 | ILE | A | 235 | 52.137 | 74.215 | 65.269 | 1.00 | 0.35 |
| ATOM | 3267 | 2HG2 | ILE | A | 235 | 52.086 | 73.091 | 66.606 | 1.00 | 0.35 |
| ATOM | 3268 | 3HG2 | ILE | A | 235 | 53.470 | 73.072 | 65.489 | 1.00 | 0.35 |
| ATOM | 3269 | CG1 | ILE | A | 235 | 52.130 | 72.337 | 63.193 | 1.00 | 0.35 |
| ATOM | 3270 | 1HG1 | ILE | A | 235 | 51.562 | 71.656 | 62.561 | 1.00 | 0.35 |
| ATOM | 3271 | 2HG1 | ILE | A | 235 | 53.187 | 72.081 | 63.119 | 1.00 | 0.35 |
| ATOM | 3272 | CD1 | ILE | A | 235 | 51.923 | 73.730 | 62.599 | 1.00 | 0.35 |
| ATOM | 3273 | HD1 | ILE | A | 235 | 52.069 | 73.686 | 61.521 | 1.00 | 0.35 |
| ATOM | 3274 | HD2 | ILE | A | 235 | 50.907 | 74.061 | 62.798 | 1.00 | 0.35 |
| ATOM | 3275 | HD3 | ILE | A | 235 | 52.636 | 74.436 | 63.024 | 1.00 | 0.35 |
| ATOM | 3276 | C | ILE | A | 235 | 51.246 | 70.580 | 66.607 | 1.00 | 0.35 |
| ATOM | 3277 | O | ILE | A | 235 | 50.229 | 71.195 | 66.974 | 1.00 | 0.35 |
| ATOM | 3278 | N | LYS | A | 236 | 51.906 | 69.713 | 67.374 | 1.00 | 0.35 |
| ATOM | 3279 | H | LYS | A | 236 | 52.751 | 69.317 | 66.977 | 1.00 | 0.35 |
| ATOM | 3280 | CA | LYS | A | 236 | 51.498 | 69.270 | 68.708 | 1.00 | 0.35 |
| ATOM | 3281 | HA | LYS | A | 236 | 50.425 | 69.239 | 68.791 | 1.00 | 0.35 |
| ATOM | 3282 | CB | LYS | A | 236 | 52.107 | 67.868 | 68.947 | 1.00 | 0.35 |
| ATOM | 3283 | HB1 | LYS | A | 236 | 51.910 | 67.563 | 69.976 | 1.00 | 0.35 |
| ATOM | 3284 | HB2 | LYS | A | 236 | 53.188 | 67.934 | 68.822 | 1.00 | 0.35 |
| ATOM | 3285 | CG | LYS | A | 236 | 51.610 | 66.752 | 68.015 | 1.00 | 0.35 |
| ATOM | 3286 | HG1 | LYS | A | 236 | 51.559 | 67.124 | 66.999 | 1.00 | 0.35 |
| ATOM | 3287 | HG2 | LYS | A | 236 | 50.603 | 66.479 | 68.312 | 1.00 | 0.35 |
| ATOM | 3288 | CD | LYS | A | 236 | 52.507 | 65.501 | 68.068 | 1.00 | 0.35 |
| ATOM | 3289 | HD1 | LYS | A | 236 | 51.891 | 64.627 | 67.850 | 1.00 | 0.35 |
| ATOM | 3290 | HD2 | LYS | A | 236 | 52.916 | 65.386 | 69.073 | 1.00 | 0.35 |
| ATOM | 3291 | CE | LYS | A | 236 | 53.640 | 65.572 | 67.031 | 1.00 | 0.35 |
| ATOM | 3292 | HE1 | LYS | A | 236 | 54.118 | 66.556 | 67.079 | 1.00 | 0.35 |
| ATOM | 3293 | HE2 | LYS | A | 236 | 53.204 | 65.480 | 66.036 | 1.00 | 0.35 |
| ATOM | 3294 | NZ | LYS | A | 236 | 54.667 | 64.516 | 67.233 | 1.00 | 0.35 |
| ATOM | 3295 | HZ1 | LYS | A | 236 | 55.425 | 64.883 | 67.801 | 1.00 | 0.35 |
| ATOM | 3296 | HZ2 | LYS | A | 236 | 54.284 | 63.683 | 67.653 | 1.00 | 0.35 |

| | | | | | | | | | | |
|---|---|---|---|---|---|---|---|---|---|---|
| ATOM | 3297 | HZ3 | LYS | A | 236 | 55.099 | 64.214 | 66.351 | 1.00 | 0.35 |
| ATOM | 3298 | C | LYS | A | 236 | 52.078 | 70.215 | 69.754 | 1.00 | 0.35 |
| ATOM | 3299 | O | LYS | A | 236 | 53.287 | 70.174 | 70.005 | 1.00 | 0.35 |
| ATOM | 3300 | N | ALA | A | 237 | 51.222 | 70.968 | 70.442 | 1.00 | 0.44 |
| ATOM | 3301 | H | ALA | A | 237 | 50.248 | 70.985 | 70.169 | 1.00 | 0.44 |
| ATOM | 3302 | CA | ALA | A | 237 | 51.570 | 71.301 | 71.832 | 1.00 | 0.44 |
| ATOM | 3303 | HA | ALA | A | 237 | 52.633 | 71.151 | 72.028 | 1.00 | 0.44 |
| ATOM | 3304 | CB | ALA | A | 237 | 51.273 | 72.790 | 72.051 | 1.00 | 0.44 |
| ATOM | 3305 | HB1 | ALA | A | 237 | 50.296 | 73.052 | 71.653 | 1.00 | 0.44 |
| ATOM | 3306 | HB2 | ALA | A | 237 | 51.290 | 73.021 | 73.115 | 1.00 | 0.44 |
| ATOM | 3307 | HB3 | ALA | A | 237 | 52.023 | 73.390 | 71.538 | 1.00 | 0.44 |
| ATOM | 3308 | C | ALA | A | 237 | 50.791 | 70.428 | 72.767 | 1.00 | 0.44 |
| ATOM | 3309 | O | ALA | A | 237 | 49.674 | 70.021 | 72.393 | 1.00 | 0.44 |
| ATOM | 3310 | N | LYS | A | 238 | 51.465 | 70.209 | 73.919 | 1.00 | 0.54 |
| ATOM | 3311 | H | LYS | A | 238 | 52.396 | 70.620 | 73.965 | 1.00 | 0.54 |
| ATOM | 3312 | CA | LYS | A | 238 | 51.023 | 69.552 | 75.157 | 1.00 | 0.54 |
| ATOM | 3313 | HA | LYS | A | 238 | 50.884 | 68.518 | 74.881 | 1.00 | 0.54 |
| ATOM | 3314 | CB | LYS | A | 238 | 52.138 | 69.633 | 76.214 | 1.00 | 0.54 |
| ATOM | 3315 | HB1 | LYS | A | 238 | 51.686 | 69.879 | 77.170 | 1.00 | 0.54 |
| ATOM | 3316 | HB2 | LYS | A | 238 | 52.817 | 70.458 | 76.002 | 1.00 | 0.54 |
| ATOM | 3317 | CG | LYS | A | 238 | 52.942 | 68.315 | 76.332 | 1.00 | 0.54 |
| ATOM | 3318 | HG1 | LYS | A | 238 | 53.852 | 68.367 | 75.733 | 1.00 | 0.54 |
| ATOM | 3319 | HG2 | LYS | A | 238 | 52.345 | 67.461 | 76.011 | 1.00 | 0.54 |
| ATOM | 3320 | CD | LYS | A | 238 | 53.273 | 68.132 | 77.809 | 1.00 | 0.54 |
| ATOM | 3321 | HD1 | LYS | A | 238 | 52.323 | 68.220 | 78.332 | 1.00 | 0.54 |
| ATOM | 3322 | HD2 | LYS | A | 238 | 53.946 | 68.932 | 78.114 | 1.00 | 0.54 |
| ATOM | 3323 | CE | LYS | A | 238 | 53.848 | 66.807 | 78.283 | 1.00 | 0.54 |
| ATOM | 3324 | HE1 | LYS | A | 238 | 54.929 | 66.797 | 78.116 | 1.00 | 0.54 |
| ATOM | 3325 | HE2 | LYS | A | 238 | 53.371 | 65.992 | 77.731 | 1.00 | 0.54 |
| ATOM | 3326 | NZ | LYS | A | 238 | 53.522 | 66.690 | 79.724 | 1.00 | 0.54 |
| ATOM | 3327 | HZ1 | LYS | A | 238 | 53.886 | 65.855 | 80.144 | 1.00 | 0.54 |
| ATOM | 3328 | HZ2 | LYS | A | 238 | 53.851 | 67.524 | 80.228 | 1.00 | 0.54 |
| ATOM | 3329 | HZ3 | LYS | A | 238 | 52.500 | 66.660 | 79.809 | 1.00 | 0.54 |
| ATOM | 3330 | C | LYS | A | 238 | 49.622 | 70.054 | 75.597 | 1.00 | 0.54 |
| ATOM | 3331 | O | LYS | A | 238 | 48.913 | 70.675 | 74.786 | 1.00 | 0.54 |
| ATOM | 3332 | N | SER | A | 239 | 49.238 | 69.784 | 76.854 | 1.00 | 0.93 |
| ATOM | 3333 | H | SER | A | 239 | 49.736 | 69.075 | 77.393 | 1.00 | 0.93 |
| ATOM | 3334 | CA | SER | A | 239 | 48.134 | 70.493 | 77.510 | 1.00 | 0.93 |
| ATOM | 3335 | HA | SER | A | 239 | 47.587 | 71.040 | 76.741 | 1.00 | 0.93 |
| ATOM | 3336 | CB | SER | A | 239 | 47.081 | 69.513 | 78.079 | 1.00 | 0.93 |
| ATOM | 3337 | HB1 | SER | A | 239 | 46.293 | 70.086 | 78.571 | 1.00 | 0.93 |
| ATOM | 3338 | HB2 | SER | A | 239 | 47.538 | 68.853 | 78.815 | 1.00 | 0.93 |
| ATOM | 3339 | OG | SER | A | 239 | 46.490 | 68.746 | 77.036 | 1.00 | 0.93 |
| ATOM | 3340 | HG | SER | A | 239 | 45.708 | 68.268 | 77.380 | 1.00 | 0.93 |

| | | | | | | | | | | |
|---|---|---|---|---|---|---|---|---|---|---|
| ATOM | 3341 | C | SER | A | 239 | 48.562 | 71.580 | 78.507 | 1.00 | 0.93 |
| ATOM | 3342 | O | SER | A | 239 | 48.534 | 72.764 | 78.165 | 1.00 | 0.93 |
| ATOM | 3343 | N | GLY | A | 240 | 48.963 | 71.241 | 79.734 | 1.00 | 0.95 |
| ATOM | 3344 | H | GLY | A | 240 | 49.075 | 70.254 | 79.939 | 1.00 | 0.95 |
| ATOM | 3345 | CA | GLY | A | 240 | 49.067 | 72.215 | 80.847 | 1.00 | 0.95 |
| ATOM | 3346 | HA1 | GLY | A | 240 | 48.801 | 71.713 | 81.777 | 1.00 | 0.95 |
| ATOM | 3347 | HA2 | GLY | A | 240 | 48.352 | 73.024 | 80.693 | 1.00 | 0.95 |
| ATOM | 3348 | C | GLY | A | 240 | 50.435 | 72.842 | 81.039 | 1.00 | 0.95 |
| ATOM | 3349 | O | GLY | A | 240 | 50.940 | 72.919 | 82.154 | 1.00 | 0.95 |
| ATOM | 3350 | N | GLU | A | 241 | 51.068 | 73.187 | 79.927 | 1.00 | 1.09 |
| ATOM | 3351 | H | GLU | A | 241 | 50.514 | 73.214 | 79.081 | 1.00 | 1.09 |
| ATOM | 3352 | CA | GLU | A | 241 | 52.521 | 73.300 | 79.756 | 1.00 | 1.09 |
| ATOM | 3353 | HA | GLU | A | 241 | 53.019 | 73.309 | 80.728 | 1.00 | 1.09 |
| ATOM | 3354 | CB | GLU | A | 241 | 52.966 | 72.056 | 78.994 | 1.00 | 1.09 |
| ATOM | 3355 | HB1 | GLU | A | 241 | 53.993 | 72.165 | 78.643 | 1.00 | 1.09 |
| ATOM | 3356 | HB2 | GLU | A | 241 | 52.328 | 71.935 | 78.118 | 1.00 | 1.09 |
| ATOM | 3357 | CG | GLU | A | 241 | 52.814 | 70.826 | 79.918 | 1.00 | 1.09 |
| ATOM | 3358 | HG1 | GLU | A | 241 | 52.374 | 70.026 | 79.345 | 1.00 | 1.09 |
| ATOM | 3359 | HG2 | GLU | A | 241 | 52.117 | 70.991 | 80.733 | 1.00 | 1.09 |
| ATOM | 3360 | CD | GLU | A | 241 | 54.131 | 70.386 | 80.538 | 1.00 | 1.09 |
| ATOM | 3361 | OE1 | GLU | A | 241 | 54.974 | 71.281 | 80.771 | 1.00 | 1.09 |
| ATOM | 3362 | OE2 | GLU | A | 241 | 54.318 | 69.150 | 80.687 | 1.00 | 1.09 |
| ATOM | 3363 | C | GLU | A | 241 | 52.808 | 74.629 | 79.072 | 1.00 | 1.09 |
| ATOM | 3364 | O | GLU | A | 241 | 51.898 | 75.249 | 78.513 | 1.00 | 1.09 |
| ATOM | 3365 | N | THR | A | 242 | 54.045 | 75.118 | 79.148 | 1.00 | 0.92 |
| ATOM | 3366 | H | THR | A | 242 | 54.788 | 74.577 | 79.573 | 1.00 | 0.92 |
| ATOM | 3367 | CA | THR | A | 242 | 54.344 | 76.459 | 78.639 | 1.00 | 0.92 |
| ATOM | 3368 | HA | THR | A | 242 | 53.549 | 76.711 | 77.962 | 1.00 | 0.92 |
| ATOM | 3369 | CB | THR | A | 242 | 54.307 | 77.509 | 79.763 | 1.00 | 0.92 |
| ATOM | 3370 | HB | THR | A | 242 | 53.390 | 77.355 | 80.332 | 1.00 | 0.92 |
| ATOM | 3371 | CG2 | THR | A | 242 | 55.486 | 77.437 | 80.733 | 1.00 | 0.92 |
| ATOM | 3372 | 1HG2 | THR | A | 242 | 55.354 | 78.182 | 81.517 | 1.00 | 0.92 |
| ATOM | 3373 | 2HG2 | THR | A | 242 | 55.534 | 76.447 | 81.186 | 1.00 | 0.92 |
| ATOM | 3374 | 3HG2 | THR | A | 242 | 56.420 | 77.636 | 80.206 | 1.00 | 0.92 |
| ATOM | 3375 | OG1 | THR | A | 242 | 54.257 | 78.817 | 79.234 | 1.00 | 0.92 |
| ATOM | 3376 | HG1 | THR | A | 242 | 53.740 | 78.805 | 78.413 | 1.00 | 0.92 |
| ATOM | 3377 | C | THR | A | 242 | 55.605 | 76.501 | 77.796 | 1.00 | 0.92 |
| ATOM | 3378 | O | THR | A | 242 | 56.539 | 75.734 | 78.012 | 1.00 | 0.92 |
| ATOM | 3379 | N | GLY | A | 243 | 55.568 | 77.338 | 76.766 | 1.00 | 0.66 |
| ATOM | 3380 | H | GLY | A | 243 | 54.774 | 77.967 | 76.666 | 1.00 | 0.66 |
| ATOM | 3381 | CA | GLY | A | 243 | 56.470 | 77.245 | 75.628 | 1.00 | 0.66 |
| ATOM | 3382 | HA1 | GLY | A | 243 | 56.491 | 76.222 | 75.254 | 1.00 | 0.66 |
| ATOM | 3383 | HA2 | GLY | A | 243 | 57.481 | 77.534 | 75.918 | 1.00 | 0.66 |
| ATOM | 3384 | C | GLY | A | 243 | 56.001 | 78.164 | 74.521 | 1.00 | 0.66 |

| | | | | | | | | | | |
|---|---|---|---|---|---|---|---|---|---|---|
| ATOM | 3385 | O | GLY | A | 243 | 55.172 | 79.044 | 74.744 | 1.00 | 0.66 |
| ATOM | 3386 | N | GLU | A | 244 | 56.518 | 77.957 | 73.321 | 1.00 | 0.43 |
| ATOM | 3387 | H | GLU | A | 244 | 57.211 | 77.222 | 73.198 | 1.00 | 0.43 |
| ATOM | 3388 | CA | GLU | A | 244 | 56.184 | 78.778 | 72.145 | 1.00 | 0.43 |
| ATOM | 3389 | HA | GLU | A | 244 | 55.128 | 79.052 | 72.149 | 1.00 | 0.43 |
| ATOM | 3390 | CB | GLU | A | 244 | 57.067 | 80.048 | 72.186 | 1.00 | 0.43 |
| ATOM | 3391 | HB1 | GLU | A | 244 | 57.628 | 80.162 | 71.256 | 1.00 | 0.43 |
| ATOM | 3392 | HB2 | GLU | A | 244 | 57.816 | 79.947 | 72.973 | 1.00 | 0.43 |
| ATOM | 3393 | CG | GLU | A | 244 | 56.266 | 81.331 | 72.466 | 1.00 | 0.43 |
| ATOM | 3394 | HG1 | GLU | A | 244 | 56.956 | 82.065 | 72.889 | 1.00 | 0.43 |
| ATOM | 3395 | HG2 | GLU | A | 244 | 55.494 | 81.141 | 73.212 | 1.00 | 0.43 |
| ATOM | 3396 | CD | GLU | A | 244 | 55.635 | 81.942 | 71.212 | 1.00 | 0.43 |
| ATOM | 3397 | OE1 | GLU | A | 244 | 55.248 | 83.133 | 71.258 | 1.00 | 0.43 |
| ATOM | 3398 | OE2 | GLU | A | 244 | 55.650 | 81.311 | 70.129 | 1.00 | 0.43 |
| ATOM | 3399 | C | GLU | A | 244 | 56.459 | 77.971 | 70.868 | 1.00 | 0.43 |
| ATOM | 3400 | O | GLU | A | 244 | 57.126 | 76.930 | 70.936 | 1.00 | 0.43 |
| ATOM | 3401 | N | ILE | A | 245 | 55.954 | 78.418 | 69.708 | 1.00 | 0.34 |
| ATOM | 3402 | H | ILE | A | 245 | 55.450 | 79.304 | 69.715 | 1.00 | 0.34 |
| ATOM | 3403 | CA | ILE | A | 245 | 56.160 | 77.684 | 68.448 | 1.00 | 0.34 |
| ATOM | 3404 | HA | ILE | A | 245 | 56.961 | 76.986 | 68.653 | 1.00 | 0.34 |
| ATOM | 3405 | CB | ILE | A | 245 | 54.987 | 76.760 | 68.053 | 1.00 | 0.34 |
| ATOM | 3406 | HB | ILE | A | 245 | 54.132 | 77.379 | 67.888 | 1.00 | 0.34 |
| ATOM | 3407 | CG2 | ILE | A | 245 | 55.251 | 76.063 | 66.712 | 1.00 | 0.34 |
| ATOM | 3408 | 1HG2 | ILE | A | 245 | 54.474 | 75.342 | 66.475 | 1.00 | 0.34 |
| ATOM | 3409 | 2HG2 | ILE | A | 245 | 55.230 | 76.818 | 65.927 | 1.00 | 0.34 |
| ATOM | 3410 | 3HG2 | ILE | A | 245 | 56.229 | 75.580 | 66.735 | 1.00 | 0.34 |
| ATOM | 3411 | CG1 | ILE | A | 245 | 54.687 | 75.743 | 69.182 | 1.00 | 0.34 |
| ATOM | 3412 | 1HG1 | ILE | A | 245 | 54.361 | 76.298 | 70.060 | 1.00 | 0.34 |
| ATOM | 3413 | 2HG1 | ILE | A | 245 | 55.606 | 75.216 | 69.440 | 1.00 | 0.34 |
| ATOM | 3414 | CD1 | ILE | A | 245 | 53.610 | 74.688 | 68.899 | 1.00 | 0.34 |
| ATOM | 3415 | HD1 | ILE | A | 245 | 53.960 | 73.974 | 68.154 | 1.00 | 0.34 |
| ATOM | 3416 | HD2 | ILE | A | 245 | 53.401 | 74.140 | 69.817 | 1.00 | 0.34 |
| ATOM | 3417 | HD3 | ILE | A | 245 | 52.695 | 75.168 | 68.555 | 1.00 | 0.34 |
| ATOM | 3418 | C | ILE | A | 245 | 56.789 | 78.466 | 67.278 | 1.00 | 0.34 |
| ATOM | 3419 | O | ILE | A | 245 | 57.668 | 77.917 | 66.607 | 1.00 | 0.34 |
| ATOM | 3420 | N | LYS | A | 246 | 56.437 | 79.736 | 67.075 | 1.00 | 0.36 |
| ATOM | 3421 | H | LYS | A | 246 | 55.634 | 80.075 | 67.588 | 1.00 | 0.36 |
| ATOM | 3422 | CA | LYS | A | 246 | 57.078 | 80.671 | 66.131 | 1.00 | 0.36 |
| ATOM | 3423 | HA | LYS | A | 246 | 56.532 | 81.595 | 66.269 | 1.00 | 0.36 |
| ATOM | 3424 | CB | LYS | A | 246 | 58.526 | 80.982 | 66.590 | 1.00 | 0.36 |
| ATOM | 3425 | HB1 | LYS | A | 246 | 59.110 | 81.460 | 65.804 | 1.00 | 0.36 |
| ATOM | 3426 | HB2 | LYS | A | 246 | 59.008 | 80.047 | 66.851 | 1.00 | 0.36 |
| ATOM | 3427 | CG | LYS | A | 246 | 58.570 | 81.899 | 67.817 | 1.00 | 0.36 |
| ATOM | 3428 | HG1 | LYS | A | 246 | 59.564 | 81.847 | 68.264 | 1.00 | 0.36 |

| | | | | | | | | | | |
|---|---|---|---|---|---|---|---|---|---|---|
| ATOM | 3429 | HG2 | LYS | A | 246 | 57.859 | 81.534 | 68.557 | 1.00 | 0.36 |
| ATOM | 3430 | CD | LYS | A | 246 | 58.281 | 83.366 | 67.436 | 1.00 | 0.36 |
| ATOM | 3431 | HD1 | LYS | A | 246 | 57.629 | 83.425 | 66.564 | 1.00 | 0.36 |
| ATOM | 3432 | HD2 | LYS | A | 246 | 59.225 | 83.844 | 67.170 | 1.00 | 0.36 |
| ATOM | 3433 | CE | LYS | A | 246 | 57.629 | 84.135 | 68.586 | 1.00 | 0.36 |
| ATOM | 3434 | HE1 | LYS | A | 246 | 57.573 | 85.192 | 68.314 | 1.00 | 0.36 |
| ATOM | 3435 | HE2 | LYS | A | 246 | 58.249 | 84.026 | 69.480 | 1.00 | 0.36 |
| ATOM | 3436 | NZ | LYS | A | 246 | 56.266 | 83.625 | 68.845 | 1.00 | 0.36 |
| ATOM | 3437 | HZ1 | LYS | A | 246 | 55.847 | 83.957 | 69.715 | 1.00 | 0.36 |
| ATOM | 3438 | HZ2 | LYS | A | 246 | 55.632 | 83.735 | 68.061 | 1.00 | 0.36 |
| ATOM | 3439 | HZ3 | LYS | A | 246 | 56.264 | 82.617 | 69.028 | 1.00 | 0.36 |
| ATOM | 3440 | C | LYS | A | 246 | 56.846 | 80.475 | 64.623 | 1.00 | 0.36 |
| ATOM | 3441 | O | LYS | A | 246 | 55.692 | 80.429 | 64.183 | 1.00 | 0.36 |
| ATOM | 3442 | N | GLY | A | 247 | 57.909 | 80.559 | 63.807 | 1.00 | 0.37 |
| ATOM | 3443 | H | GLY | A | 247 | 58.816 | 80.302 | 64.174 | 1.00 | 0.37 |
| ATOM | 3444 | CA | GLY | A | 247 | 57.823 | 81.085 | 62.437 | 1.00 | 0.37 |
| ATOM | 3445 | HA1 | GLY | A | 247 | 58.639 | 81.783 | 62.255 | 1.00 | 0.37 |
| ATOM | 3446 | HA2 | GLY | A | 247 | 56.878 | 81.580 | 62.245 | 1.00 | 0.37 |
| ATOM | 3447 | C | GLY | A | 247 | 57.962 | 79.906 | 61.493 | 1.00 | 0.37 |
| ATOM | 3448 | O | GLY | A | 247 | 59.053 | 79.516 | 61.069 | 1.00 | 0.37 |
| ATOM | 3449 | N | VAL | A | 248 | 56.831 | 79.250 | 61.305 | 1.00 | 0.34 |
| ATOM | 3450 | H | VAL | A | 248 | 55.976 | 79.719 | 61.586 | 1.00 | 0.34 |
| ATOM | 3451 | CA | VAL | A | 248 | 56.779 | 77.807 | 61.111 | 1.00 | 0.34 |
| ATOM | 3452 | HA | VAL | A | 248 | 57.781 | 77.388 | 61.025 | 1.00 | 0.34 |
| ATOM | 3453 | CB | VAL | A | 248 | 56.087 | 77.170 | 62.332 | 1.00 | 0.34 |
| ATOM | 3454 | HB | VAL | A | 248 | 55.262 | 77.802 | 62.667 | 1.00 | 0.34 |
| ATOM | 3455 | CG1 | VAL | A | 248 | 55.518 | 75.796 | 62.017 | 1.00 | 0.34 |
| ATOM | 3456 | 1HG1 | VAL | A | 248 | 55.137 | 75.344 | 62.931 | 1.00 | 0.34 |
| ATOM | 3457 | 2HG1 | VAL | A | 248 | 54.679 | 75.893 | 61.335 | 1.00 | 0.34 |
| ATOM | 3458 | 3HG1 | VAL | A | 248 | 56.286 | 75.174 | 61.557 | 1.00 | 0.34 |
| ATOM | 3459 | CG2 | VAL | A | 248 | 57.094 | 77.013 | 63.477 | 1.00 | 0.34 |
| ATOM | 3460 | 1HG2 | VAL | A | 248 | 56.638 | 76.515 | 64.323 | 1.00 | 0.34 |
| ATOM | 3461 | 2HG2 | VAL | A | 248 | 57.961 | 76.445 | 63.158 | 1.00 | 0.34 |
| ATOM | 3462 | 3HG2 | VAL | A | 248 | 57.433 | 77.986 | 63.813 | 1.00 | 0.34 |
| ATOM | 3463 | C | VAL | A | 248 | 56.050 | 77.609 | 59.790 | 1.00 | 0.34 |
| ATOM | 3464 | O | VAL | A | 248 | 54.823 | 77.712 | 59.678 | 1.00 | 0.34 |
| ATOM | 3465 | N | THR | A | 249 | 56.869 | 77.395 | 58.768 | 1.00 | 0.43 |
| ATOM | 3466 | H | THR | A | 249 | 57.860 | 77.340 | 58.970 | 1.00 | 0.43 |
| ATOM | 3467 | CA | THR | A | 249 | 56.480 | 77.397 | 57.365 | 1.00 | 0.43 |
| ATOM | 3468 | HA | THR | A | 249 | 55.457 | 77.743 | 57.324 | 1.00 | 0.43 |
| ATOM | 3469 | CB | THR | A | 249 | 57.308 | 78.437 | 56.579 | 1.00 | 0.43 |
| ATOM | 3470 | HB | THR | A | 249 | 57.986 | 78.936 | 57.274 | 1.00 | 0.43 |
| ATOM | 3471 | CG2 | THR | A | 249 | 58.158 | 77.909 | 55.426 | 1.00 | 0.43 |
| ATOM | 3472 | 1HG2 | THR | A | 249 | 58.738 | 78.730 | 55.005 | 1.00 | 0.43 |

| | | | | | | | | | | |
|---|---|---|---|---|---|---|---|---|---|---|
| ATOM | 3473 | 2HG2 | THR | A | 249 | 58.834 | 77.153 | 55.805 | 1.00 | 0.43 |
| ATOM | 3474 | 3HG2 | THR | A | 249 | 57.528 | 77.481 | 54.647 | 1.00 | 0.43 |
| ATOM | 3475 | OG1 | THR | A | 249 | 56.467 | 79.429 | 56.044 | 1.00 | 0.43 |
| ATOM | 3476 | HG1 | THR | A | 249 | 56.000 | 79.036 | 55.267 | 1.00 | 0.43 |
| ATOM | 3477 | C | THR | A | 249 | 56.506 | 75.987 | 56.773 | 1.00 | 0.43 |
| ATOM | 3478 | O | THR | A | 249 | 57.339 | 75.143 | 57.119 | 1.00 | 0.43 |
| ATOM | 3479 | N | TYR | A | 250 | 55.554 | 75.729 | 55.891 | 1.00 | 0.41 |
| ATOM | 3480 | H | TYR | A | 250 | 54.943 | 76.502 | 55.640 | 1.00 | 0.41 |
| ATOM | 3481 | CA | TYR | A | 250 | 55.156 | 74.404 | 55.451 | 1.00 | 0.41 |
| ATOM | 3482 | HA | TYR | A | 250 | 55.990 | 73.707 | 55.509 | 1.00 | 0.41 |
| ATOM | 3483 | CB | TYR | A | 250 | 54.001 | 73.908 | 56.352 | 1.00 | 0.41 |
| ATOM | 3484 | HB1 | TYR | A | 250 | 53.376 | 73.221 | 55.787 | 1.00 | 0.41 |
| ATOM | 3485 | HB2 | TYR | A | 250 | 53.365 | 74.760 | 56.582 | 1.00 | 0.41 |
| ATOM | 3486 | CG | TYR | A | 250 | 54.370 | 73.211 | 57.653 | 1.00 | 0.41 |
| ATOM | 3487 | CD1 | TYR | A | 250 | 54.253 | 73.895 | 58.878 | 1.00 | 0.41 |
| ATOM | 3488 | HD1 | TYR | A | 250 | 53.980 | 74.944 | 58.883 | 1.00 | 0.41 |
| ATOM | 3489 | CE1 | TYR | A | 250 | 54.472 | 73.209 | 60.089 | 1.00 | 0.41 |
| ATOM | 3490 | HE1 | TYR | A | 250 | 54.372 | 73.704 | 61.037 | 1.00 | 0.41 |
| ATOM | 3491 | CZ | TYR | A | 250 | 54.816 | 71.846 | 60.091 | 1.00 | 0.41 |
| ATOM | 3492 | OH | TYR | A | 250 | 55.021 | 71.200 | 61.267 | 1.00 | 0.41 |
| ATOM | 3493 | HH | TYR | A | 250 | 55.264 | 70.284 | 61.115 | 1.00 | 0.41 |
| ATOM | 3494 | CE2 | TYR | A | 250 | 54.941 | 71.162 | 58.866 | 1.00 | 0.41 |
| ATOM | 3495 | HE2 | TYR | A | 250 | 55.214 | 70.118 | 58.857 | 1.00 | 0.41 |
| ATOM | 3496 | CD2 | TYR | A | 250 | 54.722 | 71.844 | 57.655 | 1.00 | 0.41 |
| ATOM | 3497 | HD2 | TYR | A | 250 | 54.822 | 71.306 | 56.729 | 1.00 | 0.41 |
| ATOM | 3498 | C | TYR | A | 250 | 54.703 | 74.530 | 53.985 | 1.00 | 0.41 |
| ATOM | 3499 | O | TYR | A | 250 | 53.552 | 74.863 | 53.700 | 1.00 | 0.41 |
| ATOM | 3500 | N | SER | A | 251 | 55.632 | 74.302 | 53.057 | 1.00 | 0.33 |
| ATOM | 3501 | H | SER | A | 251 | 56.536 | 73.981 | 53.389 | 1.00 | 0.33 |
| ATOM | 3502 | CA | SER | A | 251 | 55.604 | 74.943 | 51.737 | 1.00 | 0.33 |
| ATOM | 3503 | HA | SER | A | 251 | 54.657 | 75.453 | 51.587 | 1.00 | 0.33 |
| ATOM | 3504 | CB | SER | A | 251 | 56.726 | 75.985 | 51.755 | 1.00 | 0.33 |
| ATOM | 3505 | HB1 | SER | A | 251 | 57.682 | 75.469 | 51.775 | 1.00 | 0.33 |
| ATOM | 3506 | HB2 | SER | A | 251 | 56.650 | 76.575 | 52.668 | 1.00 | 0.33 |
| ATOM | 3507 | OG | SER | A | 251 | 56.698 | 76.860 | 50.651 | 1.00 | 0.33 |
| ATOM | 3508 | HG | SER | A | 251 | 56.384 | 77.724 | 51.028 | 1.00 | 0.33 |
| ATOM | 3509 | C | SER | A | 251 | 55.844 | 73.946 | 50.584 | 1.00 | 0.33 |
| ATOM | 3510 | O | SER | A | 251 | 56.859 | 73.239 | 50.511 | 1.00 | 0.33 |
| ATOM | 3511 | N | GLY | A | 252 | 54.887 | 73.879 | 49.664 | 1.00 | 0.49 |
| ATOM | 3512 | H | GLY | A | 252 | 54.068 | 74.469 | 49.812 | 1.00 | 0.49 |
| ATOM | 3513 | CA | GLY | A | 252 | 54.795 | 72.875 | 48.615 | 1.00 | 0.49 |
| ATOM | 3514 | HA1 | GLY | A | 252 | 55.783 | 72.514 | 48.329 | 1.00 | 0.49 |
| ATOM | 3515 | HA2 | GLY | A | 252 | 54.316 | 73.316 | 47.741 | 1.00 | 0.49 |
| ATOM | 3516 | C | GLY | A | 252 | 53.928 | 71.700 | 49.089 | 1.00 | 0.49 |

| | | | | | | | | | | |
|---|---|---|---|---|---|---|---|---|---|---|
| ATOM | 3517 | O | GLY | A | 252 | 52.729 | 71.616 | 48.837 | 1.00 | 0.49 |
| ATOM | 3518 | N | ILE | A | 253 | 54.594 | 70.852 | 49.856 | 1.00 | 0.42 |
| ATOM | 3519 | H | ILE | A | 253 | 55.585 | 71.033 | 49.922 | 1.00 | 0.42 |
| ATOM | 3520 | CA | ILE | A | 253 | 54.178 | 69.634 | 50.549 | 1.00 | 0.42 |
| ATOM | 3521 | HA | ILE | A | 253 | 55.171 | 69.320 | 50.860 | 1.00 | 0.42 |
| ATOM | 3522 | CB | ILE | A | 253 | 53.572 | 69.823 | 51.947 | 1.00 | 0.42 |
| ATOM | 3523 | HB | ILE | A | 253 | 52.499 | 70.005 | 51.885 | 1.00 | 0.42 |
| ATOM | 3524 | CG2 | ILE | A | 253 | 53.857 | 68.540 | 52.743 | 1.00 | 0.42 |
| ATOM | 3525 | 1HG2 | ILE | A | 253 | 53.278 | 68.532 | 53.662 | 1.00 | 0.42 |
| ATOM | 3526 | 2HG2 | ILE | A | 253 | 53.597 | 67.663 | 52.158 | 1.00 | 0.42 |
| ATOM | 3527 | 3HG2 | ILE | A | 253 | 54.916 | 68.464 | 52.991 | 1.00 | 0.42 |
| ATOM | 3528 | CG1 | ILE | A | 253 | 54.300 | 70.993 | 52.653 | 1.00 | 0.42 |
| ATOM | 3529 | 1HG1 | ILE | A | 253 | 53.986 | 71.934 | 52.204 | 1.00 | 0.42 |
| ATOM | 3530 | 2HG1 | ILE | A | 253 | 55.375 | 70.894 | 52.495 | 1.00 | 0.42 |
| ATOM | 3531 | CD1 | ILE | A | 253 | 54.089 | 71.094 | 54.160 | 1.00 | 0.42 |
| ATOM | 3532 | HD1 | ILE | A | 253 | 54.402 | 70.187 | 54.674 | 1.00 | 0.42 |
| ATOM | 3533 | HD2 | ILE | A | 253 | 54.718 | 71.896 | 54.526 | 1.00 | 0.42 |
| ATOM | 3534 | HD3 | ILE | A | 253 | 53.045 | 71.306 | 54.371 | 1.00 | 0.42 |
| ATOM | 3535 | C | ILE | A | 253 | 53.860 | 68.403 | 49.688 | 1.00 | 0.42 |
| ATOM | 3536 | O | ILE | A | 253 | 54.815 | 67.731 | 49.296 | 1.00 | 0.42 |
| ATOM | 3537 | N | SER | A | 254 | 52.595 | 68.085 | 49.402 | 1.00 | 0.46 |
| ATOM | 3538 | H | SER | A | 254 | 51.868 | 68.711 | 49.731 | 1.00 | 0.46 |
| ATOM | 3539 | CA | SER | A | 254 | 52.167 | 66.866 | 48.663 | 1.00 | 0.46 |
| ATOM | 3540 | HA | SER | A | 254 | 51.207 | 67.130 | 48.219 | 1.00 | 0.46 |
| ATOM | 3541 | CB | SER | A | 254 | 53.035 | 66.460 | 47.457 | 1.00 | 0.46 |
| ATOM | 3542 | HB1 | SER | A | 254 | 54.002 | 66.080 | 47.776 | 1.00 | 0.46 |
| ATOM | 3543 | HB2 | SER | A | 254 | 53.190 | 67.333 | 46.823 | 1.00 | 0.46 |
| ATOM | 3544 | OG | SER | A | 254 | 52.389 | 65.450 | 46.698 | 1.00 | 0.46 |
| ATOM | 3545 | HG | SER | A | 254 | 52.623 | 64.557 | 47.052 | 1.00 | 0.46 |
| ATOM | 3546 | C | SER | A | 254 | 51.907 | 65.626 | 49.548 | 1.00 | 0.46 |
| ATOM | 3547 | O | SER | A | 254 | 52.690 | 64.689 | 49.720 | 1.00 | 0.46 |
| ATOM | 3548 | N | LEU | A | 255 | 50.707 | 65.679 | 50.095 | 1.00 | 0.41 |
| ATOM | 3549 | H | LEU | A | 255 | 50.203 | 66.521 | 49.879 | 1.00 | 0.41 |
| ATOM | 3550 | CA | LEU | A | 255 | 50.140 | 64.933 | 51.202 | 1.00 | 0.41 |
| ATOM | 3551 | HA | LEU | A | 255 | 50.895 | 64.292 | 51.656 | 1.00 | 0.41 |
| ATOM | 3552 | CB | LEU | A | 255 | 49.762 | 66.082 | 52.208 | 1.00 | 0.41 |
| ATOM | 3553 | HB1 | LEU | A | 255 | 49.205 | 65.669 | 53.042 | 1.00 | 0.41 |
| ATOM | 3554 | HB2 | LEU | A | 255 | 49.068 | 66.743 | 51.683 | 1.00 | 0.41 |
| ATOM | 3555 | CG | LEU | A | 255 | 50.806 | 67.031 | 52.799 | 1.00 | 0.41 |
| ATOM | 3556 | HG | LEU | A | 255 | 51.421 | 67.434 | 52.000 | 1.00 | 0.41 |
| ATOM | 3557 | CD1 | LEU | A | 255 | 50.093 | 68.207 | 53.475 | 1.00 | 0.41 |
| ATOM | 3558 | 1HD1 | LEU | A | 255 | 50.825 | 68.936 | 53.817 | 1.00 | 0.41 |
| ATOM | 3559 | 2HD1 | LEU | A | 255 | 49.437 | 68.690 | 52.754 | 1.00 | 0.41 |
| ATOM | 3560 | 3HD1 | LEU | A | 255 | 49.492 | 67.857 | 54.314 | 1.00 | 0.41 |

| | | | | | | | | | | |
|---|---|---|---|---|---|---|---|---|---|---|
| ATOM | 3561 | CD2 | LEU | A | 255 | 51.677 | 66.349 | 53.852 | 1.00 | 0.41 |
| ATOM | 3562 | 1HD2 | LEU | A | 255 | 52.641 | 66.151 | 53.398 | 1.00 | 0.41 |
| ATOM | 3563 | 2HD2 | LEU | A | 255 | 51.837 | 66.988 | 54.719 | 1.00 | 0.41 |
| ATOM | 3564 | 3HD2 | LEU | A | 255 | 51.229 | 65.409 | 54.164 | 1.00 | 0.41 |
| ATOM | 3565 | C | LEU | A | 255 | 48.883 | 64.141 | 50.908 | 1.00 | 0.41 |
| ATOM | 3566 | O | LEU | A | 255 | 47.837 | 64.821 | 51.095 | 1.00 | 0.41 |
| ATOM | 3567 | N | GLU | A | 256 | 49.177 | 62.825 | 50.602 | 1.00 | 0.52 |
| ATOM | 3568 | H | GLU | A | 256 | 50.159 | 62.679 | 50.818 | 1.00 | 0.52 |
| ATOM | 3569 | CA | GLU | A | 256 | 48.733 | 61.617 | 49.783 | 1.00 | 0.52 |
| ATOM | 3570 | HA | GLU | A | 256 | 47.888 | 61.837 | 49.148 | 1.00 | 0.52 |
| ATOM | 3571 | CB | GLU | A | 256 | 49.925 | 61.212 | 48.895 | 1.00 | 0.52 |
| ATOM | 3572 | HB1 | GLU | A | 256 | 49.638 | 60.346 | 48.297 | 1.00 | 0.52 |
| ATOM | 3573 | HB2 | GLU | A | 256 | 50.707 | 60.894 | 49.580 | 1.00 | 0.52 |
| ATOM | 3574 | CG | GLU | A | 256 | 50.551 | 62.266 | 47.965 | 1.00 | 0.52 |
| ATOM | 3575 | HG1 | GLU | A | 256 | 50.510 | 63.253 | 48.425 | 1.00 | 0.52 |
| ATOM | 3576 | HG2 | GLU | A | 256 | 49.978 | 62.307 | 47.035 | 1.00 | 0.52 |
| ATOM | 3577 | CD | GLU | A | 256 | 52.024 | 61.919 | 47.664 | 1.00 | 0.52 |
| ATOM | 3578 | OE1 | GLU | A | 256 | 52.363 | 60.708 | 47.705 | 1.00 | 0.52 |
| ATOM | 3579 | OE2 | GLU | A | 256 | 52.826 | 62.856 | 47.417 | 1.00 | 0.52 |
| ATOM | 3580 | C | GLU | A | 256 | 48.390 | 60.362 | 50.626 | 1.00 | 0.52 |
| ATOM | 3581 | O | GLU | A | 256 | 49.214 | 59.829 | 51.379 | 1.00 | 0.52 |
| ATOM | 3582 | N | SER | A | 257 | 47.188 | 59.805 | 50.436 | 1.00 | 0.98 |
| ATOM | 3583 | H | SER | A | 257 | 46.531 | 60.300 | 49.843 | 1.00 | 0.98 |
| ATOM | 3584 | CA | SER | A | 257 | 46.700 | 58.617 | 51.166 | 1.00 | 0.98 |
| ATOM | 3585 | HA | SER | A | 257 | 45.629 | 58.560 | 50.972 | 1.00 | 0.98 |
| ATOM | 3586 | CB | SER | A | 257 | 47.321 | 57.338 | 50.591 | 1.00 | 0.98 |
| ATOM | 3587 | HB1 | SER | A | 257 | 48.369 | 57.267 | 50.885 | 1.00 | 0.98 |
| ATOM | 3588 | HB2 | SER | A | 257 | 47.261 | 57.365 | 49.502 | 1.00 | 0.98 |
| ATOM | 3589 | OG | SER | A | 257 | 46.612 | 56.209 | 51.059 | 1.00 | 0.98 |
| ATOM | 3590 | HG | SER | A | 257 | 46.711 | 56.186 | 52.023 | 1.00 | 0.98 |
| ATOM | 3591 | C | SER | A | 257 | 46.837 | 58.729 | 52.688 | 1.00 | 0.98 |
| ATOM | 3592 | O | SER | A | 257 | 47.227 | 57.759 | 53.347 | 1.00 | 0.98 |
| ATOM | 3593 | N | ILE | A | 258 | 46.562 | 59.914 | 53.245 | 1.00 | 0.82 |
| ATOM | 3594 | H | ILE | A | 258 | 46.306 | 60.690 | 52.642 | 1.00 | 0.82 |
| ATOM | 3595 | CA | ILE | A | 258 | 46.721 | 60.144 | 54.687 | 1.00 | 0.82 |
| ATOM | 3596 | HA | ILE | A | 258 | 47.593 | 59.584 | 54.997 | 1.00 | 0.82 |
| ATOM | 3597 | CB | ILE | A | 258 | 47.003 | 61.632 | 54.968 | 1.00 | 0.82 |
| ATOM | 3598 | HB | ILE | A | 258 | 46.279 | 62.237 | 54.419 | 1.00 | 0.82 |
| ATOM | 3599 | CG2 | ILE | A | 258 | 46.890 | 61.975 | 56.467 | 1.00 | 0.82 |
| ATOM | 3600 | 1HG2 | ILE | A | 258 | 47.154 | 63.015 | 56.641 | 1.00 | 0.82 |
| ATOM | 3601 | 2HG2 | ILE | A | 258 | 45.868 | 61.833 | 56.819 | 1.00 | 0.82 |
| ATOM | 3602 | 3HG2 | ILE | A | 258 | 47.560 | 61.340 | 57.044 | 1.00 | 0.82 |
| ATOM | 3603 | CG1 | ILE | A | 258 | 48.428 | 61.944 | 54.459 | 1.00 | 0.82 |
| ATOM | 3604 | 1HG1 | ILE | A | 258 | 48.569 | 61.534 | 53.459 | 1.00 | 0.82 |

| | | | | | | | | | | |
|---|---|---|---|---|---|---|---|---|---|---|
| ATOM | 3605 | 2HG1 | ILE | A | 258 | 49.166 | 61.483 | 55.117 | 1.00 | 0.82 |
| ATOM | 3606 | CD1 | ILE | A | 258 | 48.706 | 63.438 | 54.362 | 1.00 | 0.82 |
| ATOM | 3607 | HD1 | ILE | A | 258 | 49.700 | 63.579 | 53.939 | 1.00 | 0.82 |
| ATOM | 3608 | HD2 | ILE | A | 258 | 47.958 | 63.901 | 53.718 | 1.00 | 0.82 |
| ATOM | 3609 | HD3 | ILE | A | 258 | 48.669 | 63.902 | 55.342 | 1.00 | 0.82 |
| ATOM | 3610 | C | ILE | A | 258 | 45.521 | 59.614 | 55.477 | 1.00 | 0.82 |
| ATOM | 3611 | O | ILE | A | 258 | 44.379 | 60.000 | 55.228 | 1.00 | 0.82 |
| ATOM | 3612 | N | SER | A | 259 | 45.791 | 58.736 | 56.437 | 1.00 | 0.94 |
| ATOM | 3613 | H | SER | A | 259 | 46.762 | 58.491 | 56.586 | 1.00 | 0.94 |
| ATOM | 3614 | CA | SER | A | 259 | 44.782 | 57.973 | 57.167 | 1.00 | 0.94 |
| ATOM | 3615 | HA | SER | A | 259 | 44.034 | 57.631 | 56.453 | 1.00 | 0.94 |
| ATOM | 3616 | CB | SER | A | 259 | 45.401 | 56.726 | 57.809 | 1.00 | 0.94 |
| ATOM | 3617 | HB1 | SER | A | 259 | 44.718 | 56.320 | 58.558 | 1.00 | 0.94 |
| ATOM | 3618 | HB2 | SER | A | 259 | 46.342 | 56.984 | 58.295 | 1.00 | 0.94 |
| ATOM | 3619 | OG | SER | A | 259 | 45.618 | 55.741 | 56.810 | 1.00 | 0.94 |
| ATOM | 3620 | HG | SER | A | 259 | 44.762 | 55.344 | 56.618 | 1.00 | 0.94 |
| ATOM | 3621 | C | SER | A | 259 | 44.013 | 58.743 | 58.232 | 1.00 | 0.94 |
| ATOM | 3622 | O | SER | A | 259 | 42.823 | 58.484 | 58.360 | 1.00 | 0.94 |
| ATOM | 3623 | N | ASP | A | 260 | 44.624 | 59.696 | 58.946 | 1.00 | 0.96 |
| ATOM | 3624 | H | ASP | A | 260 | 45.626 | 59.797 | 58.860 | 1.00 | 0.96 |
| ATOM | 3625 | CA | ASP | A | 260 | 44.000 | 60.256 | 60.153 | 1.00 | 0.96 |
| ATOM | 3626 | HA | ASP | A | 260 | 42.993 | 59.847 | 60.244 | 1.00 | 0.96 |
| ATOM | 3627 | CB | ASP | A | 260 | 44.702 | 59.840 | 61.463 | 1.00 | 0.96 |
| ATOM | 3628 | HB1 | ASP | A | 260 | 44.084 | 60.217 | 62.278 | 1.00 | 0.96 |
| ATOM | 3629 | HB2 | ASP | A | 260 | 45.663 | 60.337 | 61.550 | 1.00 | 0.96 |
| ATOM | 3630 | CG | ASP | A | 260 | 44.910 | 58.337 | 61.709 | 1.00 | 0.96 |
| ATOM | 3631 | OD1 | ASP | A | 260 | 45.368 | 57.633 | 60.777 | 1.00 | 0.96 |
| ATOM | 3632 | OD2 | ASP | A | 260 | 44.727 | 57.931 | 62.880 | 1.00 | 0.96 |
| ATOM | 3633 | C | ASP | A | 260 | 43.803 | 61.765 | 60.096 | 1.00 | 0.96 |
| ATOM | 3634 | O | ASP | A | 260 | 42.692 | 62.235 | 60.321 | 1.00 | 0.96 |
| ATOM | 3635 | N | TYR | A | 261 | 44.828 | 62.537 | 59.737 | 1.00 | 0.86 |
| ATOM | 3636 | H | TYR | A | 261 | 45.719 | 62.086 | 59.574 | 1.00 | 0.86 |
| ATOM | 3637 | CA | TYR | A | 261 | 44.733 | 64.005 | 59.744 | 1.00 | 0.86 |
| ATOM | 3638 | HA | TYR | A | 261 | 43.705 | 64.309 | 59.623 | 1.00 | 0.86 |
| ATOM | 3639 | CB | TYR | A | 261 | 45.202 | 64.549 | 61.110 | 1.00 | 0.86 |
| ATOM | 3640 | HB1 | TYR | A | 261 | 45.018 | 65.624 | 61.115 | 1.00 | 0.86 |
| ATOM | 3641 | HB2 | TYR | A | 261 | 46.279 | 64.399 | 61.203 | 1.00 | 0.86 |
| ATOM | 3642 | CG | TYR | A | 261 | 44.551 | 63.947 | 62.342 | 1.00 | 0.86 |
| ATOM | 3643 | CD1 | TYR | A | 261 | 45.140 | 62.833 | 62.970 | 1.00 | 0.86 |
| ATOM | 3644 | HD1 | TYR | A | 261 | 46.053 | 62.410 | 62.578 | 1.00 | 0.86 |
| ATOM | 3645 | CE1 | TYR | A | 261 | 44.525 | 62.242 | 64.088 | 1.00 | 0.86 |
| ATOM | 3646 | HE1 | TYR | A | 261 | 44.948 | 61.361 | 64.543 | 1.00 | 0.86 |
| ATOM | 3647 | CZ | TYR | A | 261 | 43.323 | 62.773 | 64.593 | 1.00 | 0.86 |
| ATOM | 3648 | OH | TYR | A | 261 | 42.723 | 62.185 | 65.662 | 1.00 | 0.86 |

| | | | | | | | | | | |
|---|---|---|---|---|---|---|---|---|---|---|
| ATOM | 3649 | HH | TYR | A | 261 | 41.930 | 62.648 | 65.930 | 1.00 | 0.86 |
| ATOM | 3650 | CE2 | TYR | A | 261 | 42.747 | 63.913 | 63.987 | 1.00 | 0.86 |
| ATOM | 3651 | HE2 | TYR | A | 261 | 41.833 | 64.325 | 64.378 | 1.00 | 0.86 |
| ATOM | 3652 | CD2 | TYR | A | 261 | 43.367 | 64.499 | 62.866 | 1.00 | 0.86 |
| ATOM | 3653 | HD2 | TYR | A | 261 | 42.934 | 65.368 | 62.401 | 1.00 | 0.86 |
| ATOM | 3654 | C | TYR | A | 261 | 45.600 | 64.629 | 58.648 | 1.00 | 0.86 |
| ATOM | 3655 | O | TYR | A | 261 | 46.830 | 64.590 | 58.717 | 1.00 | 0.86 |
| ATOM | 3656 | N | GLY | A | 262 | 44.981 | 65.245 | 57.639 | 1.00 | 0.80 |
| ATOM | 3657 | H | GLY | A | 262 | 43.968 | 65.238 | 57.614 | 1.00 | 0.80 |
| ATOM | 3658 | CA | GLY | A | 262 | 45.752 | 65.920 | 56.575 | 1.00 | 0.80 |
| ATOM | 3659 | HA1 | GLY | A | 262 | 45.064 | 66.370 | 55.864 | 1.00 | 0.80 |
| ATOM | 3660 | HA2 | GLY | A | 262 | 46.368 | 65.192 | 56.047 | 1.00 | 0.80 |
| ATOM | 3661 | C | GLY | A | 262 | 46.667 | 67.023 | 57.121 | 1.00 | 0.80 |
| ATOM | 3662 | O | GLY | A | 262 | 47.904 | 66.902 | 57.115 | 1.00 | 0.80 |
| ATOM | 3663 | N | ILE | A | 263 | 46.020 | 68.010 | 57.742 | 1.00 | 0.39 |
| ATOM | 3664 | H | ILE | A | 263 | 45.010 | 67.990 | 57.703 | 1.00 | 0.39 |
| ATOM | 3665 | CA | ILE | A | 263 | 46.650 | 68.977 | 58.647 | 1.00 | 0.39 |
| ATOM | 3666 | HA | ILE | A | 263 | 47.722 | 68.790 | 58.684 | 1.00 | 0.39 |
| ATOM | 3667 | CB | ILE | A | 263 | 46.427 | 70.444 | 58.197 | 1.00 | 0.39 |
| ATOM | 3668 | HB | ILE | A | 263 | 45.437 | 70.731 | 58.544 | 1.00 | 0.39 |
| ATOM | 3669 | CG2 | ILE | A | 263 | 47.451 | 71.375 | 58.871 | 1.00 | 0.39 |
| ATOM | 3670 | 1HG2 | ILE | A | 263 | 47.077 | 72.399 | 58.874 | 1.00 | 0.39 |
| ATOM | 3671 | 2HG2 | ILE | A | 263 | 47.647 | 71.078 | 59.899 | 1.00 | 0.39 |
| ATOM | 3672 | 3HG2 | ILE | A | 263 | 48.389 | 71.364 | 58.321 | 1.00 | 0.39 |
| ATOM | 3673 | CG1 | ILE | A | 263 | 46.454 | 70.670 | 56.670 | 1.00 | 0.39 |
| ATOM | 3674 | 1HG1 | ILE | A | 263 | 45.668 | 70.075 | 56.214 | 1.00 | 0.39 |
| ATOM | 3675 | 2HG1 | ILE | A | 263 | 47.409 | 70.339 | 56.263 | 1.00 | 0.39 |
| ATOM | 3676 | CD1 | ILE | A | 263 | 46.204 | 72.133 | 56.280 | 1.00 | 0.39 |
| ATOM | 3677 | HD1 | ILE | A | 263 | 45.941 | 72.202 | 55.227 | 1.00 | 0.39 |
| ATOM | 3678 | HD2 | ILE | A | 263 | 45.375 | 72.534 | 56.860 | 1.00 | 0.39 |
| ATOM | 3679 | HD3 | ILE | A | 263 | 47.096 | 72.734 | 56.459 | 1.00 | 0.39 |
| ATOM | 3680 | C | ILE | A | 263 | 46.075 | 68.782 | 60.048 | 1.00 | 0.39 |
| ATOM | 3681 | O | ILE | A | 263 | 44.859 | 68.643 | 60.213 | 1.00 | 0.39 |
| ATOM | 3682 | N | LEU | A | 264 | 46.924 | 68.823 | 61.071 | 1.00 | 0.39 |
| ATOM | 3683 | H | LEU | A | 264 | 47.925 | 68.895 | 60.937 | 1.00 | 0.39 |
| ATOM | 3684 | CA | LEU | A | 264 | 46.528 | 68.740 | 62.459 | 1.00 | 0.39 |
| ATOM | 3685 | HA | LEU | A | 264 | 45.463 | 68.926 | 62.582 | 1.00 | 0.39 |
| ATOM | 3686 | CB | LEU | A | 264 | 46.898 | 67.320 | 62.950 | 1.00 | 0.39 |
| ATOM | 3687 | HB1 | LEU | A | 264 | 47.960 | 67.309 | 63.197 | 1.00 | 0.39 |
| ATOM | 3688 | HB2 | LEU | A | 264 | 46.776 | 66.617 | 62.130 | 1.00 | 0.39 |
| ATOM | 3689 | CG | LEU | A | 264 | 46.109 | 66.764 | 64.147 | 1.00 | 0.39 |
| ATOM | 3690 | HG | LEU | A | 264 | 45.122 | 66.469 | 63.807 | 1.00 | 0.39 |
| ATOM | 3691 | CD1 | LEU | A | 264 | 46.814 | 65.515 | 64.674 | 1.00 | 0.39 |
| ATOM | 3692 | 1HD1 | LEU | A | 264 | 46.211 | 65.059 | 65.458 | 1.00 | 0.39 |

| | | | | | | | | | | |
|---|---|---|---|---|---|---|---|---|---|---|
| ATOM | 3693 | 2HD1 | LEU | A | 264 | 46.961 | 64.794 | 63.871 | 1.00 | 0.39 |
| ATOM | 3694 | 3HD1 | LEU | A | 264 | 47.776 | 65.794 | 65.101 | 1.00 | 0.39 |
| ATOM | 3695 | CD2 | LEU | A | 264 | 45.945 | 67.764 | 65.287 | 1.00 | 0.39 |
| ATOM | 3696 | 1HD2 | LEU | A | 264 | 45.550 | 67.264 | 66.172 | 1.00 | 0.39 |
| ATOM | 3697 | 2HD2 | LEU | A | 264 | 46.915 | 68.189 | 65.531 | 1.00 | 0.39 |
| ATOM | 3698 | 3HD2 | LEU | A | 264 | 45.271 | 68.567 | 65.004 | 1.00 | 0.39 |
| ATOM | 3699 | C | LEU | A | 264 | 47.361 | 69.790 | 63.203 | 1.00 | 0.39 |
| ATOM | 3700 | O | LEU | A | 264 | 48.586 | 69.827 | 63.104 | 1.00 | 0.39 |
| ATOM | 3701 | N | ILE | A | 265 | 46.707 | 70.668 | 63.935 | 1.00 | 0.48 |
| ATOM | 3702 | H | ILE | A | 265 | 45.691 | 70.630 | 63.947 | 1.00 | 0.48 |
| ATOM | 3703 | CA | ILE | A | 265 | 47.351 | 71.551 | 64.893 | 1.00 | 0.48 |
| ATOM | 3704 | HA | ILE | A | 265 | 48.382 | 71.250 | 65.081 | 1.00 | 0.48 |
| ATOM | 3705 | CB | ILE | A | 265 | 47.313 | 73.020 | 64.430 | 1.00 | 0.48 |
| ATOM | 3706 | HB | ILE | A | 265 | 46.278 | 73.322 | 64.265 | 1.00 | 0.48 |
| ATOM | 3707 | CG2 | ILE | A | 265 | 47.886 | 73.904 | 65.544 | 1.00 | 0.48 |
| ATOM | 3708 | 1HG2 | ILE | A | 265 | 47.969 | 74.933 | 65.205 | 1.00 | 0.48 |
| ATOM | 3709 | 2HG2 | ILE | A | 265 | 47.232 | 73.892 | 66.420 | 1.00 | 0.48 |
| ATOM | 3710 | 3HG2 | ILE | A | 265 | 48.871 | 73.544 | 65.836 | 1.00 | 0.48 |
| ATOM | 3711 | CG1 | ILE | A | 265 | 48.117 | 73.248 | 63.132 | 1.00 | 0.48 |
| ATOM | 3712 | 1HG1 | ILE | A | 265 | 48.935 | 72.532 | 63.068 | 1.00 | 0.48 |
| ATOM | 3713 | 2HG1 | ILE | A | 265 | 48.548 | 74.245 | 63.143 | 1.00 | 0.48 |
| ATOM | 3714 | CD1 | ILE | A | 265 | 47.265 | 73.151 | 61.865 | 1.00 | 0.48 |
| ATOM | 3715 | HD1 | ILE | A | 265 | 47.909 | 73.311 | 61.001 | 1.00 | 0.48 |
| ATOM | 3716 | HD2 | ILE | A | 265 | 46.792 | 72.175 | 61.785 | 1.00 | 0.48 |
| ATOM | 3717 | HD3 | ILE | A | 265 | 46.495 | 73.919 | 61.883 | 1.00 | 0.48 |
| ATOM | 3718 | C | ILE | A | 265 | 46.560 | 71.340 | 66.157 | 1.00 | 0.48 |
| ATOM | 3719 | O | ILE | A | 265 | 45.352 | 71.554 | 66.074 | 1.00 | 0.48 |
| ATOM | 3720 | N | GLU | A | 266 | 47.153 | 70.894 | 67.271 | 1.00 | 0.58 |
| ATOM | 3721 | H | GLU | A | 266 | 48.152 | 70.708 | 67.315 | 1.00 | 0.58 |
| ATOM | 3722 | CA | GLU | A | 266 | 46.320 | 70.648 | 68.432 | 1.00 | 0.58 |
| ATOM | 3723 | HA | GLU | A | 266 | 45.271 | 70.670 | 68.142 | 1.00 | 0.58 |
| ATOM | 3724 | CB | GLU | A | 266 | 46.451 | 69.210 | 68.986 | 1.00 | 0.58 |
| ATOM | 3725 | HB1 | GLU | A | 266 | 45.848 | 68.548 | 68.363 | 1.00 | 0.58 |
| ATOM | 3726 | HB2 | GLU | A | 266 | 46.008 | 69.179 | 69.982 | 1.00 | 0.58 |
| ATOM | 3727 | CG | GLU | A | 266 | 47.867 | 68.640 | 69.055 | 1.00 | 0.58 |
| ATOM | 3728 | HG1 | GLU | A | 266 | 47.892 | 67.905 | 69.859 | 1.00 | 0.58 |
| ATOM | 3729 | HG2 | GLU | A | 266 | 48.530 | 69.452 | 69.351 | 1.00 | 0.58 |
| ATOM | 3730 | CD | GLU | A | 266 | 48.318 | 67.957 | 67.740 | 1.00 | 0.58 |
| ATOM | 3731 | OE1 | GLU | A | 266 | 47.992 | 66.770 | 67.541 | 1.00 | 0.58 |
| ATOM | 3732 | OE2 | GLU | A | 266 | 49.043 | 68.584 | 66.937 | 1.00 | 0.58 |
| ATOM | 3733 | C | GLU | A | 266 | 46.398 | 71.832 | 69.341 | 1.00 | 0.58 |
| ATOM | 3734 | O | GLU | A | 266 | 46.013 | 72.887 | 68.828 | 1.00 | 0.58 |
| ATOM | 3735 | N | GLN | A | 267 | 46.897 | 71.581 | 70.564 | 1.00 | 0.54 |
| ATOM | 3736 | H | GLN | A | 267 | 47.014 | 70.597 | 70.726 | 1.00 | 0.54 |

| | | | | | | | | | | |
|---|---|---|---|---|---|---|---|---|---|---|
| ATOM | 3737 | CA | GLN | A | 267 | 47.245 | 72.415 | 71.726 | 1.00 | 0.54 |
| ATOM | 3738 | HA | GLN | A | 267 | 48.312 | 72.319 | 71.890 | 1.00 | 0.54 |
| ATOM | 3739 | CB | GLN | A | 267 | 46.942 | 73.894 | 71.408 | 1.00 | 0.54 |
| ATOM | 3740 | HB1 | GLN | A | 267 | 45.862 | 74.036 | 71.356 | 1.00 | 0.54 |
| ATOM | 3741 | HB2 | GLN | A | 267 | 47.367 | 74.104 | 70.427 | 1.00 | 0.54 |
| ATOM | 3742 | CG | GLN | A | 267 | 47.538 | 74.974 | 72.311 | 1.00 | 0.54 |
| ATOM | 3743 | HG1 | GLN | A | 267 | 48.587 | 74.757 | 72.507 | 1.00 | 0.54 |
| ATOM | 3744 | HG2 | GLN | A | 267 | 46.993 | 75.007 | 73.251 | 1.00 | 0.54 |
| ATOM | 3745 | CD | GLN | A | 267 | 47.416 | 76.325 | 71.605 | 1.00 | 0.54 |
| ATOM | 3746 | OE1 | GLN | A | 267 | 46.368 | 76.748 | 71.161 | 1.00 | 0.54 |
| ATOM | 3747 | NE2 | GLN | A | 267 | 48.462 | 77.079 | 71.404 | 1.00 | 0.54 |
| ATOM | 3748 | 1HE2 | GLN | A | 267 | 49.389 | 76.841 | 71.744 | 1.00 | 0.54 |
| ATOM | 3749 | 2HE2 | GLN | A | 267 | 48.285 | 77.964 | 70.943 | 1.00 | 0.54 |
| ATOM | 3750 | C | GLN | A | 267 | 46.572 | 71.834 | 72.975 | 1.00 | 0.54 |
| ATOM | 3751 | O | GLN | A | 267 | 46.019 | 72.539 | 73.815 | 1.00 | 0.54 |
| ATOM | 3752 | N | ASN | A | 268 | 46.574 | 70.500 | 72.974 | 1.00 | 0.63 |
| ATOM | 3753 | H | ASN | A | 268 | 47.280 | 70.078 | 72.389 | 1.00 | 0.63 |
| ATOM | 3754 | CA | ASN | A | 268 | 45.817 | 69.555 | 73.770 | 1.00 | 0.63 |
| ATOM | 3755 | HA | ASN | A | 268 | 45.951 | 69.780 | 74.827 | 1.00 | 0.63 |
| ATOM | 3756 | CB | ASN | A | 268 | 44.328 | 69.650 | 73.380 | 1.00 | 0.63 |
| ATOM | 3757 | HB1 | ASN | A | 268 | 44.186 | 69.314 | 72.353 | 1.00 | 0.63 |
| ATOM | 3758 | HB2 | ASN | A | 268 | 43.982 | 70.670 | 73.432 | 1.00 | 0.63 |
| ATOM | 3759 | CG | ASN | A | 268 | 43.453 | 68.813 | 74.284 | 1.00 | 0.63 |
| ATOM | 3760 | OD1 | ASN | A | 268 | 42.740 | 67.938 | 73.827 | 1.00 | 0.63 |
| ATOM | 3761 | ND2 | ASN | A | 268 | 43.512 | 69.039 | 75.573 | 1.00 | 0.63 |
| ATOM | 3762 | 1HD2 | ASN | A | 268 | 43.936 | 69.867 | 75.954 | 1.00 | 0.63 |
| ATOM | 3763 | 2HD2 | ASN | A | 268 | 42.872 | 68.552 | 76.204 | 1.00 | 0.63 |
| ATOM | 3764 | C | ASN | A | 268 | 46.410 | 68.182 | 73.448 | 1.00 | 0.63 |
| ATOM | 3765 | O | ASN | A | 268 | 45.903 | 67.453 | 72.598 | 1.00 | 0.63 |
| ATOM | 3766 | N | TYR | A | 269 | 47.572 | 67.909 | 74.032 | 1.00 | 0.88 |
| ATOM | 3767 | H | TYR | A | 269 | 47.940 | 68.604 | 74.668 | 1.00 | 0.88 |
| ATOM | 3768 | CA | TYR | A | 269 | 48.125 | 66.565 | 74.088 | 1.00 | 0.88 |
| ATOM | 3769 | HA | TYR | A | 269 | 47.291 | 65.876 | 73.972 | 1.00 | 0.88 |
| ATOM | 3770 | CB | TYR | A | 269 | 49.175 | 66.278 | 72.994 | 1.00 | 0.88 |
| ATOM | 3771 | HB1 | TYR | A | 269 | 50.062 | 65.845 | 73.457 | 1.00 | 0.88 |
| ATOM | 3772 | HB2 | TYR | A | 269 | 49.481 | 67.222 | 72.539 | 1.00 | 0.88 |
| ATOM | 3773 | CG | TYR | A | 269 | 48.734 | 65.310 | 71.903 | 1.00 | 0.88 |
| ATOM | 3774 | CD1 | TYR | A | 269 | 49.117 | 65.539 | 70.573 | 1.00 | 0.88 |
| ATOM | 3775 | HD1 | TYR | A | 269 | 49.706 | 66.407 | 70.332 | 1.00 | 0.88 |
| ATOM | 3776 | CE1 | TYR | A | 269 | 48.663 | 64.688 | 69.548 | 1.00 | 0.88 |
| ATOM | 3777 | HE1 | TYR | A | 269 | 48.927 | 64.884 | 68.524 | 1.00 | 0.88 |
| ATOM | 3778 | CZ | TYR | A | 269 | 47.788 | 63.625 | 69.833 | 1.00 | 0.88 |
| ATOM | 3779 | OH | TYR | A | 269 | 47.254 | 62.905 | 68.812 | 1.00 | 0.88 |
| ATOM | 3780 | HH | TYR | A | 269 | 47.435 | 63.333 | 67.972 | 1.00 | 0.88 |

| | | | | | | | | | | |
|---|---|---|---|---|---|---|---|---|---|---|
| ATOM | 3781 | CE2 | TYR | A | 269 | 47.467 | 63.342 | 71.172 | 1.00 | 0.88 |
| ATOM | 3782 | HE2 | TYR | A | 269 | 46.819 | 62.509 | 71.397 | 1.00 | 0.88 |
| ATOM | 3783 | CD2 | TYR | A | 269 | 47.955 | 64.172 | 72.201 | 1.00 | 0.88 |
| ATOM | 3784 | HD2 | TYR | A | 269 | 47.685 | 63.958 | 73.220 | 1.00 | 0.88 |
| ATOM | 3785 | C | TYR | A | 269 | 48.676 | 66.350 | 75.491 | 1.00 | 0.88 |
| ATOM | 3786 | O | TYR | A | 269 | 49.693 | 66.941 | 75.856 | 1.00 | 0.88 |
| ATOM | 3787 | N | ASP | A | 270 | 48.047 | 65.491 | 76.288 | 1.00 | 1.19 |
| ATOM | 3788 | H | ASP | A | 270 | 47.204 | 65.009 | 75.970 | 1.00 | 1.19 |
| ATOM | 3789 | CA | ASP | A | 270 | 48.724 | 64.990 | 77.474 | 1.00 | 1.19 |
| ATOM | 3790 | HA | ASP | A | 270 | 49.788 | 65.070 | 77.242 | 1.00 | 1.19 |
| ATOM | 3791 | CB | ASP | A | 270 | 48.548 | 65.884 | 78.723 | 1.00 | 1.19 |
| ATOM | 3792 | HB1 | ASP | A | 270 | 48.275 | 65.261 | 79.576 | 1.00 | 1.19 |
| ATOM | 3793 | HB2 | ASP | A | 270 | 47.735 | 66.591 | 78.560 | 1.00 | 1.19 |
| ATOM | 3794 | CG | ASP | A | 270 | 49.852 | 66.646 | 79.072 | 1.00 | 1.19 |
| ATOM | 3795 | OD1 | ASP | A | 270 | 50.871 | 65.973 | 79.387 | 1.00 | 1.19 |
| ATOM | 3796 | OD2 | ASP | A | 270 | 49.863 | 67.900 | 79.039 | 1.00 | 1.19 |
| ATOM | 3797 | C | ASP | A | 270 | 48.598 | 63.492 | 77.707 | 1.00 | 1.19 |
| ATOM | 3798 | O | ASP | A | 270 | 47.667 | 62.845 | 77.249 | 1.00 | 1.19 |
| ATOM | 3799 | N | GLY | A | 271 | 49.657 | 62.918 | 78.284 | 1.00 | 1.90 |
| ATOM | 3800 | H | GLY | A | 271 | 50.379 | 63.526 | 78.648 | 1.00 | 1.90 |
| ATOM | 3801 | CA | GLY | A | 271 | 49.994 | 61.521 | 78.019 | 1.00 | 1.90 |
| ATOM | 3802 | HA1 | GLY | A | 271 | 49.120 | 60.893 | 78.210 | 1.00 | 1.90 |
| ATOM | 3803 | HA2 | GLY | A | 271 | 50.794 | 61.202 | 78.685 | 1.00 | 1.90 |
| ATOM | 3804 | C | GLY | A | 271 | 50.451 | 61.299 | 76.573 | 1.00 | 1.90 |
| ATOM | 3805 | O | GLY | A | 271 | 51.601 | 61.574 | 76.222 | 1.00 | 1.90 |
| ATOM | 3806 | N | GLY | A | 272 | 49.517 | 60.867 | 75.733 | 1.00 | 1.96 |
| ATOM | 3807 | H | GLY | A | 272 | 48.629 | 60.557 | 76.145 | 1.00 | 1.96 |
| ATOM | 3808 | CA | GLY | A | 272 | 49.530 | 61.000 | 74.273 | 1.00 | 1.96 |
| ATOM | 3809 | HA1 | GLY | A | 272 | 50.037 | 60.147 | 73.826 | 1.00 | 1.96 |
| ATOM | 3810 | HA2 | GLY | A | 272 | 50.039 | 61.922 | 73.994 | 1.00 | 1.96 |
| ATOM | 3811 | C | GLY | A | 272 | 48.099 | 61.063 | 73.744 | 1.00 | 1.96 |
| ATOM | 3812 | O | GLY | A | 272 | 47.782 | 60.504 | 72.694 | 1.00 | 1.96 |
| ATOM | 3813 | N | ASP | A | 273 | 47.246 | 61.741 | 74.509 | 1.00 | 1.85 |
| ATOM | 3814 | H | ASP | A | 273 | 47.598 | 62.114 | 75.387 | 1.00 | 1.85 |
| ATOM | 3815 | CA | ASP | A | 273 | 45.796 | 61.727 | 74.434 | 1.00 | 1.85 |
| ATOM | 3816 | HA | ASP | A | 273 | 45.475 | 61.137 | 73.576 | 1.00 | 1.85 |
| ATOM | 3817 | CB | ASP | A | 273 | 45.239 | 61.076 | 75.721 | 1.00 | 1.85 |
| ATOM | 3818 | HB1 | ASP | A | 273 | 44.164 | 60.933 | 75.591 | 1.00 | 1.85 |
| ATOM | 3819 | HB2 | ASP | A | 273 | 45.367 | 61.766 | 76.556 | 1.00 | 1.85 |
| ATOM | 3820 | CG | ASP | A | 273 | 45.891 | 59.734 | 76.099 | 1.00 | 1.85 |
| ATOM | 3821 | OD1 | ASP | A | 273 | 47.041 | 59.740 | 76.607 | 1.00 | 1.85 |
| ATOM | 3822 | OD2 | ASP | A | 273 | 45.229 | 58.693 | 75.882 | 1.00 | 1.85 |
| ATOM | 3823 | C | ASP | A | 273 | 45.284 | 63.166 | 74.302 | 1.00 | 1.85 |
| ATOM | 3824 | O | ASP | A | 273 | 45.781 | 64.079 | 74.965 | 1.00 | 1.85 |

| | | | | | | | | | | |
|---|---|---|---|---|---|---|---|---|---|---|
| ATOM | 3825 | N | LEU | A | 274 | 44.289 | 63.388 | 73.441 | 1.00 | 1.60 |
| ATOM | 3826 | H | LEU | A | 274 | 43.899 | 62.612 | 72.932 | 1.00 | 1.60 |
| ATOM | 3827 | CA | LEU | A | 274 | 43.687 | 64.707 | 73.256 | 1.00 | 1.60 |
| ATOM | 3828 | HA | LEU | A | 274 | 44.341 | 65.457 | 73.707 | 1.00 | 1.60 |
| ATOM | 3829 | CB | LEU | A | 274 | 43.614 | 65.131 | 71.770 | 1.00 | 1.60 |
| ATOM | 3830 | HB1 | LEU | A | 274 | 44.632 | 65.306 | 71.418 | 1.00 | 1.60 |
| ATOM | 3831 | HB2 | LEU | A | 274 | 43.117 | 66.102 | 71.756 | 1.00 | 1.60 |
| ATOM | 3832 | CG | LEU | A | 274 | 42.905 | 64.201 | 70.763 | 1.00 | 1.60 |
| ATOM | 3833 | HG | LEU | A | 274 | 42.078 | 63.679 | 71.240 | 1.00 | 1.60 |
| ATOM | 3834 | CD1 | LEU | A | 274 | 42.344 | 65.024 | 69.596 | 1.00 | 1.60 |
| ATOM | 3835 | 1HD1 | LEU | A | 274 | 41.838 | 64.371 | 68.887 | 1.00 | 1.60 |
| ATOM | 3836 | 2HD1 | LEU | A | 274 | 41.626 | 65.749 | 69.981 | 1.00 | 1.60 |
| ATOM | 3837 | 3HD1 | LEU | A | 274 | 43.154 | 65.556 | 69.094 | 1.00 | 1.60 |
| ATOM | 3838 | CD2 | LEU | A | 274 | 43.852 | 63.165 | 70.147 | 1.00 | 1.60 |
| ATOM | 3839 | 1HD2 | LEU | A | 274 | 43.317 | 62.554 | 69.420 | 1.00 | 1.60 |
| ATOM | 3840 | 2HD2 | LEU | A | 274 | 44.681 | 63.671 | 69.651 | 1.00 | 1.60 |
| ATOM | 3841 | 3HD2 | LEU | A | 274 | 44.252 | 62.512 | 70.918 | 1.00 | 1.60 |
| ATOM | 3842 | C | LEU | A | 274 | 42.377 | 64.811 | 74.031 | 1.00 | 1.60 |
| ATOM | 3843 | O | LEU | A | 274 | 41.301 | 64.498 | 73.521 | 1.00 | 1.60 |
| ATOM | 3844 | N | ASP | A | 275 | 42.513 | 65.173 | 75.307 | 1.00 | 1.80 |
| ATOM | 3845 | H | ASP | A | 275 | 43.448 | 65.397 | 75.627 | 1.00 | 1.80 |
| ATOM | 3846 | CA | ASP | A | 275 | 41.432 | 65.230 | 76.294 | 1.00 | 1.80 |
| ATOM | 3847 | HA | ASP | A | 275 | 41.051 | 64.214 | 76.385 | 1.00 | 1.80 |
| ATOM | 3848 | CB | ASP | A | 275 | 42.014 | 65.592 | 77.675 | 1.00 | 1.80 |
| ATOM | 3849 | HB1 | ASP | A | 275 | 42.789 | 64.867 | 77.930 | 1.00 | 1.80 |
| ATOM | 3850 | HB2 | ASP | A | 275 | 41.224 | 65.507 | 78.423 | 1.00 | 1.80 |
| ATOM | 3851 | CG | ASP | A | 275 | 42.598 | 67.004 | 77.733 | 1.00 | 1.80 |
| ATOM | 3852 | OD1 | ASP | A | 275 | 41.808 | 67.945 | 77.510 | 1.00 | 1.80 |
| ATOM | 3853 | OD2 | ASP | A | 275 | 43.825 | 67.149 | 77.949 | 1.00 | 1.80 |
| ATOM | 3854 | C | ASP | A | 275 | 40.199 | 66.070 | 75.904 | 1.00 | 1.80 |
| ATOM | 3855 | O | ASP | A | 275 | 39.108 | 65.823 | 76.424 | 1.00 | 1.80 |
| ATOM | 3856 | N | GLY | A | 276 | 40.356 | 66.991 | 74.953 | 1.00 | 2.69 |
| ATOM | 3857 | H | GLY | A | 276 | 41.298 | 67.121 | 74.599 | 1.00 | 2.69 |
| ATOM | 3858 | CA | GLY | A | 276 | 39.325 | 67.862 | 74.401 | 1.00 | 2.69 |
| ATOM | 3859 | HA1 | GLY | A | 276 | 38.351 | 67.583 | 74.805 | 1.00 | 2.69 |
| ATOM | 3860 | HA2 | GLY | A | 276 | 39.303 | 67.755 | 73.318 | 1.00 | 2.69 |
| ATOM | 3861 | C | GLY | A | 276 | 39.567 | 69.334 | 74.739 | 1.00 | 2.69 |
| ATOM | 3862 | O | GLY | A | 276 | 39.299 | 70.208 | 73.919 | 1.00 | 2.69 |
| ATOM | 3863 | N | GLU | A | 277 | 40.124 | 69.613 | 75.915 | 1.00 | 1.44 |
| ATOM | 3864 | H | GLU | A | 277 | 40.443 | 68.846 | 76.508 | 1.00 | 1.44 |
| ATOM | 3865 | CA | GLU | A | 277 | 40.298 | 70.961 | 76.451 | 1.00 | 1.44 |
| ATOM | 3866 | HA | GLU | A | 277 | 39.631 | 71.655 | 75.936 | 1.00 | 1.44 |
| ATOM | 3867 | CB | GLU | A | 277 | 39.916 | 70.984 | 77.946 | 1.00 | 1.44 |
| ATOM | 3868 | HB1 | GLU | A | 277 | 40.245 | 71.924 | 78.391 | 1.00 | 1.44 |

| | | | | | | | | | | |
|---|---|---|---|---|---|---|---|---|---|---|
| ATOM | 3869 | HB2 | GLU | A | 277 | 40.450 | 70.179 | 78.453 | 1.00 | 1.44 |
| ATOM | 3870 | CG | GLU | A | 277 | 38.411 | 70.815 | 78.226 | 1.00 | 1.44 |
| ATOM | 3871 | HG1 | GLU | A | 277 | 38.308 | 70.413 | 79.238 | 1.00 | 1.44 |
| ATOM | 3872 | HG2 | GLU | A | 277 | 37.979 | 70.080 | 77.544 | 1.00 | 1.44 |
| ATOM | 3873 | CD | GLU | A | 277 | 37.628 | 72.136 | 78.138 | 1.00 | 1.44 |
| ATOM | 3874 | OE1 | GLU | A | 277 | 37.767 | 72.844 | 77.115 | 1.00 | 1.44 |
| ATOM | 3875 | OE2 | GLU | A | 277 | 36.877 | 72.429 | 79.100 | 1.00 | 1.44 |
| ATOM | 3876 | C | GLU | A | 277 | 41.726 | 71.454 | 76.255 | 1.00 | 1.44 |
| ATOM | 3877 | O | GLU | A | 277 | 42.643 | 71.285 | 77.064 | 1.00 | 1.44 |
| ATOM | 3878 | N | VAL | A | 278 | 41.891 | 72.072 | 75.098 | 1.00 | 1.15 |
| ATOM | 3879 | H | VAL | A | 278 | 41.080 | 72.052 | 74.495 | 1.00 | 1.15 |
| ATOM | 3880 | CA | VAL | A | 278 | 42.963 | 72.992 | 74.720 | 1.00 | 1.15 |
| ATOM | 3881 | HA | VAL | A | 278 | 43.804 | 72.432 | 74.355 | 1.00 | 1.15 |
| ATOM | 3882 | CB | VAL | A | 278 | 42.403 | 73.887 | 73.596 | 1.00 | 1.15 |
| ATOM | 3883 | HB | VAL | A | 278 | 43.219 | 74.491 | 73.207 | 1.00 | 1.15 |
| ATOM | 3884 | CG1 | VAL | A | 278 | 41.835 | 73.033 | 72.461 | 1.00 | 1.15 |
| ATOM | 3885 | 1HG1 | VAL | A | 278 | 41.587 | 73.683 | 71.624 | 1.00 | 1.15 |
| ATOM | 3886 | 2HG1 | VAL | A | 278 | 42.568 | 72.287 | 72.157 | 1.00 | 1.15 |
| ATOM | 3887 | 3HG1 | VAL | A | 278 | 40.918 | 72.525 | 72.762 | 1.00 | 1.15 |
| ATOM | 3888 | CG2 | VAL | A | 278 | 41.247 | 74.805 | 74.045 | 1.00 | 1.15 |
| ATOM | 3889 | 1HG2 | VAL | A | 278 | 40.859 | 75.361 | 73.192 | 1.00 | 1.15 |
| ATOM | 3890 | 2HG2 | VAL | A | 278 | 40.432 | 74.222 | 74.475 | 1.00 | 1.15 |
| ATOM | 3891 | 3HG2 | VAL | A | 278 | 41.587 | 75.517 | 74.796 | 1.00 | 1.15 |
| ATOM | 3892 | C | VAL | A | 278 | 43.427 | 73.870 | 75.879 | 1.00 | 1.15 |
| ATOM | 3893 | O | VAL | A | 278 | 42.640 | 74.277 | 76.735 | 1.00 | 1.15 |
| ATOM | 3894 | N | THR | A | 279 | 44.706 | 74.242 | 75.900 | 1.00 | 0.98 |
| ATOM | 3895 | H | THR | A | 279 | 45.353 | 73.871 | 75.213 | 1.00 | 0.98 |
| ATOM | 3896 | CA | THR | A | 279 | 45.191 | 75.187 | 76.914 | 1.00 | 0.98 |
| ATOM | 3897 | HA | THR | A | 279 | 44.384 | 75.898 | 77.101 | 1.00 | 0.98 |
| ATOM | 3898 | CB | THR | A | 279 | 45.483 | 74.518 | 78.286 | 1.00 | 0.98 |
| ATOM | 3899 | HB | THR | A | 279 | 44.758 | 74.919 | 78.995 | 1.00 | 0.98 |
| ATOM | 3900 | CG2 | THR | A | 279 | 45.384 | 72.992 | 78.361 | 1.00 | 0.98 |
| ATOM | 3901 | 1HG2 | THR | A | 279 | 45.769 | 72.634 | 79.315 | 1.00 | 0.98 |
| ATOM | 3902 | 2HG2 | THR | A | 279 | 44.337 | 72.701 | 78.295 | 1.00 | 0.98 |
| ATOM | 3903 | 3HG2 | THR | A | 279 | 45.933 | 72.534 | 77.541 | 1.00 | 0.98 |
| ATOM | 3904 | OG1 | THR | A | 279 | 46.762 | 74.830 | 78.796 | 1.00 | 0.98 |
| ATOM | 3905 | HG1 | THR | A | 279 | 47.393 | 74.204 | 78.395 | 1.00 | 0.98 |
| ATOM | 3906 | C | THR | A | 279 | 46.373 | 76.028 | 76.431 | 1.00 | 0.98 |
| ATOM | 3907 | O | THR | A | 279 | 47.251 | 75.578 | 75.692 | 1.00 | 0.98 |
| ATOM | 3908 | N | SER | A | 280 | 46.380 | 77.290 | 76.859 | 1.00 | 0.89 |
| ATOM | 3909 | H | SER | A | 280 | 45.665 | 77.581 | 77.516 | 1.00 | 0.89 |
| ATOM | 3910 | CA | SER | A | 280 | 46.991 | 78.391 | 76.121 | 1.00 | 0.89 |
| ATOM | 3911 | HA | SER | A | 280 | 46.942 | 78.125 | 75.065 | 1.00 | 0.89 |
| ATOM | 3912 | CB | SER | A | 280 | 46.113 | 79.647 | 76.268 | 1.00 | 0.89 |

| | | | | | | | | | | |
|---|---|---|---|---|---|---|---|---|---|---|
| ATOM | 3913 | HB1 | SER | A | 280 | 45.260 | 79.535 | 75.599 | 1.00 | 0.89 |
| ATOM | 3914 | HB2 | SER | A | 280 | 46.659 | 80.541 | 75.965 | 1.00 | 0.89 |
| ATOM | 3915 | OG | SER | A | 280 | 45.612 | 79.811 | 77.579 | 1.00 | 0.89 |
| ATOM | 3916 | HG | SER | A | 280 | 44.764 | 80.312 | 77.479 | 1.00 | 0.89 |
| ATOM | 3917 | C | SER | A | 280 | 48.467 | 78.692 | 76.367 | 1.00 | 0.89 |
| ATOM | 3918 | O | SER | A | 280 | 49.031 | 79.572 | 75.717 | 1.00 | 0.89 |
| ATOM | 3919 | N | GLY | A | 281 | 49.141 | 77.940 | 77.236 | 1.00 | 0.94 |
| ATOM | 3920 | H | GLY | A | 281 | 48.637 | 77.189 | 77.689 | 1.00 | 0.94 |
| ATOM | 3921 | CA | GLY | A | 281 | 50.516 | 78.240 | 77.648 | 1.00 | 0.94 |
| ATOM | 3922 | HA1 | GLY | A | 281 | 50.767 | 77.598 | 78.492 | 1.00 | 0.94 |
| ATOM | 3923 | HA2 | GLY | A | 281 | 50.561 | 79.277 | 77.983 | 1.00 | 0.94 |
| ATOM | 3924 | C | GLY | A | 281 | 51.604 | 78.053 | 76.587 | 1.00 | 0.94 |
| ATOM | 3925 | O | GLY | A | 281 | 52.750 | 78.446 | 76.827 | 1.00 | 0.94 |
| ATOM | 3926 | N | ILE | A | 282 | 51.266 | 77.486 | 75.422 | 1.00 | 0.62 |
| ATOM | 3927 | H | ILE | A | 282 | 50.293 | 77.245 | 75.288 | 1.00 | 0.62 |
| ATOM | 3928 | CA | ILE | A | 282 | 52.196 | 77.265 | 74.306 | 1.00 | 0.62 |
| ATOM | 3929 | HA | ILE | A | 282 | 53.135 | 77.761 | 74.535 | 1.00 | 0.62 |
| ATOM | 3930 | CB | ILE | A | 282 | 52.526 | 75.759 | 74.109 | 1.00 | 0.62 |
| ATOM | 3931 | HB | ILE | A | 282 | 51.647 | 75.259 | 73.702 | 1.00 | 0.62 |
| ATOM | 3932 | CG2 | ILE | A | 282 | 53.686 | 75.594 | 73.109 | 1.00 | 0.62 |
| ATOM | 3933 | 1HG2 | ILE | A | 282 | 53.806 | 74.548 | 72.833 | 1.00 | 0.62 |
| ATOM | 3934 | 2HG2 | ILE | A | 282 | 53.494 | 76.155 | 72.197 | 1.00 | 0.62 |
| ATOM | 3935 | 3HG2 | ILE | A | 282 | 54.617 | 75.948 | 73.548 | 1.00 | 0.62 |
| ATOM | 3936 | CG1 | ILE | A | 282 | 52.859 | 75.079 | 75.452 | 1.00 | 0.62 |
| ATOM | 3937 | 1HG1 | ILE | A | 282 | 51.942 | 75.030 | 76.034 | 1.00 | 0.62 |
| ATOM | 3938 | 2HG1 | ILE | A | 282 | 53.578 | 75.695 | 75.982 | 1.00 | 0.62 |
| ATOM | 3939 | CD1 | ILE | A | 282 | 53.424 | 73.661 | 75.389 | 1.00 | 0.62 |
| ATOM | 3940 | HD1 | ILE | A | 282 | 54.403 | 73.663 | 74.911 | 1.00 | 0.62 |
| ATOM | 3941 | HD2 | ILE | A | 282 | 53.540 | 73.284 | 76.404 | 1.00 | 0.62 |
| ATOM | 3942 | HD3 | ILE | A | 282 | 52.742 | 73.008 | 74.849 | 1.00 | 0.62 |
| ATOM | 3943 | C | ILE | A | 282 | 51.662 | 77.923 | 73.026 | 1.00 | 0.62 |
| ATOM | 3944 | O | ILE | A | 282 | 51.087 | 77.220 | 72.187 | 1.00 | 0.62 |
| ATOM | 3945 | N | PRO | A | 283 | 51.760 | 79.262 | 72.893 | 1.00 | 0.44 |
| ATOM | 3946 | CD | PRO | A | 283 | 52.227 | 80.221 | 73.888 | 1.00 | 0.44 |
| ATOM | 3947 | HD1 | PRO | A | 283 | 53.313 | 80.267 | 73.865 | 1.00 | 0.44 |
| ATOM | 3948 | HD2 | PRO | A | 283 | 51.873 | 79.992 | 74.889 | 1.00 | 0.44 |
| ATOM | 3949 | CG | PRO | A | 283 | 51.669 | 81.574 | 73.463 | 1.00 | 0.44 |
| ATOM | 3950 | HG1 | PRO | A | 283 | 52.291 | 82.398 | 73.812 | 1.00 | 0.44 |
| ATOM | 3951 | HG2 | PRO | A | 283 | 50.643 | 81.678 | 73.823 | 1.00 | 0.44 |
| ATOM | 3952 | CB | PRO | A | 283 | 51.677 | 81.459 | 71.945 | 1.00 | 0.44 |
| ATOM | 3953 | HB1 | PRO | A | 283 | 52.681 | 81.681 | 71.578 | 1.00 | 0.44 |
| ATOM | 3954 | HB2 | PRO | A | 283 | 50.946 | 82.125 | 71.486 | 1.00 | 0.44 |
| ATOM | 3955 | CA | PRO | A | 283 | 51.334 | 79.985 | 71.703 | 1.00 | 0.44 |
| ATOM | 3956 | HA | PRO | A | 283 | 50.254 | 79.889 | 71.643 | 1.00 | 0.44 |

| | | | | | | | | | | |
|---|---|---|---|---|---|---|---|---|---|---|
| ATOM | 3957 | C | PRO | A | 283 | 51.953 | 79.503 | 70.383 | 1.00 | 0.44 |
| ATOM | 3958 | O | PRO | A | 283 | 53.157 | 79.253 | 70.358 | 1.00 | 0.44 |
| ATOM | 3959 | N | ILE | A | 284 | 51.181 | 79.390 | 69.277 | 1.00 | 0.38 |
| ATOM | 3960 | H | ILE | A | 284 | 50.168 | 79.343 | 69.317 | 1.00 | 0.38 |
| ATOM | 3961 | CA | ILE | A | 284 | 51.731 | 78.825 | 68.049 | 1.00 | 0.38 |
| ATOM | 3962 | HA | ILE | A | 284 | 52.486 | 78.139 | 68.381 | 1.00 | 0.38 |
| ATOM | 3963 | CB | ILE | A | 284 | 50.673 | 77.933 | 67.349 | 1.00 | 0.38 |
| ATOM | 3964 | HB | ILE | A | 284 | 49.897 | 78.569 | 66.919 | 1.00 | 0.38 |
| ATOM | 3965 | CG2 | ILE | A | 284 | 51.328 | 77.129 | 66.211 | 1.00 | 0.38 |
| ATOM | 3966 | 1HG2 | ILE | A | 284 | 50.583 | 76.526 | 65.694 | 1.00 | 0.38 |
| ATOM | 3967 | 2HG2 | ILE | A | 284 | 51.777 | 77.799 | 65.478 | 1.00 | 0.38 |
| ATOM | 3968 | 3HG2 | ILE | A | 284 | 52.104 | 76.480 | 66.613 | 1.00 | 0.38 |
| ATOM | 3969 | CG1 | ILE | A | 284 | 50.014 | 76.990 | 68.388 | 1.00 | 0.38 |
| ATOM | 3970 | 1HG1 | ILE | A | 284 | 49.376 | 77.594 | 69.026 | 1.00 | 0.38 |
| ATOM | 3971 | 2HG1 | ILE | A | 284 | 50.786 | 76.536 | 69.012 | 1.00 | 0.38 |
| ATOM | 3972 | CD1 | ILE | A | 284 | 49.130 | 75.872 | 67.840 | 1.00 | 0.38 |
| ATOM | 3973 | HD1 | ILE | A | 284 | 49.743 | 75.128 | 67.335 | 1.00 | 0.38 |
| ATOM | 3974 | HD2 | ILE | A | 284 | 48.609 | 75.386 | 68.665 | 1.00 | 0.38 |
| ATOM | 3975 | HD3 | ILE | A | 284 | 48.394 | 76.290 | 67.155 | 1.00 | 0.38 |
| ATOM | 3976 | C | ILE | A | 284 | 52.409 | 79.824 | 67.074 | 1.00 | 0.38 |
| ATOM | 3977 | O | ILE | A | 284 | 53.516 | 79.583 | 66.620 | 1.00 | 0.38 |
| ATOM | 3978 | N | THR | A | 285 | 51.735 | 80.929 | 66.769 | 1.00 | 0.48 |
| ATOM | 3979 | H | THR | A | 285 | 50.764 | 80.920 | 67.048 | 1.00 | 0.48 |
| ATOM | 3980 | CA | THR | A | 285 | 52.214 | 82.259 | 66.322 | 1.00 | 0.48 |
| ATOM | 3981 | HA | THR | A | 285 | 51.317 | 82.800 | 66.586 | 1.00 | 0.48 |
| ATOM | 3982 | CB | THR | A | 285 | 53.204 | 83.005 | 67.220 | 1.00 | 0.48 |
| ATOM | 3983 | HB | THR | A | 285 | 53.053 | 84.073 | 67.059 | 1.00 | 0.48 |
| ATOM | 3984 | CG2 | THR | A | 285 | 52.975 | 82.731 | 68.706 | 1.00 | 0.48 |
| ATOM | 3985 | 1HG2 | THR | A | 285 | 53.521 | 83.460 | 69.301 | 1.00 | 0.48 |
| ATOM | 3986 | 2HG2 | THR | A | 285 | 51.917 | 82.821 | 68.946 | 1.00 | 0.48 |
| ATOM | 3987 | 3HG2 | THR | A | 285 | 53.327 | 81.732 | 68.960 | 1.00 | 0.48 |
| ATOM | 3988 | OG1 | THR | A | 285 | 54.537 | 82.723 | 66.910 | 1.00 | 0.48 |
| ATOM | 3989 | HG1 | THR | A | 285 | 54.794 | 83.284 | 66.132 | 1.00 | 0.48 |
| ATOM | 3990 | C | THR | A | 285 | 52.332 | 82.681 | 64.867 | 1.00 | 0.48 |
| ATOM | 3991 | O | THR | A | 285 | 51.680 | 83.675 | 64.572 | 1.00 | 0.48 |
| ATOM | 3992 | N | ASP | A | 286 | 53.087 | 82.064 | 63.955 | 1.00 | 0.50 |
| ATOM | 3993 | H | ASP | A | 286 | 53.422 | 81.102 | 64.070 | 1.00 | 0.50 |
| ATOM | 3994 | CA | ASP | A | 286 | 53.493 | 82.785 | 62.721 | 1.00 | 0.50 |
| ATOM | 3995 | HA | ASP | A | 286 | 52.772 | 83.540 | 62.440 | 1.00 | 0.50 |
| ATOM | 3996 | CB | ASP | A | 286 | 54.827 | 83.531 | 62.942 | 1.00 | 0.50 |
| ATOM | 3997 | HB1 | ASP | A | 286 | 55.119 | 84.018 | 62.010 | 1.00 | 0.50 |
| ATOM | 3998 | HB2 | ASP | A | 286 | 55.594 | 82.813 | 63.204 | 1.00 | 0.50 |
| ATOM | 3999 | CG | ASP | A | 286 | 54.782 | 84.580 | 64.057 | 1.00 | 0.50 |
| ATOM | 4000 | OD1 | ASP | A | 286 | 55.424 | 84.332 | 65.110 | 1.00 | 0.50 |

| | | | | | | | | | | |
|---|---|---|---|---|---|---|---|---|---|---|
| ATOM | 4001 | OD2 | ASP | A | 286 | 54.092 | 85.613 | 63.895 | 1.00 | 0.50 |
| ATOM | 4002 | C | ASP | A | 286 | 53.562 | 81.719 | 61.622 | 1.00 | 0.50 |
| ATOM | 4003 | O | ASP | A | 286 | 54.613 | 81.343 | 61.084 | 1.00 | 0.50 |
| ATOM | 4004 | N | LEU | A | 287 | 52.409 | 81.076 | 61.454 | 1.00 | 0.41 |
| ATOM | 4005 | H | LEU | A | 287 | 51.593 | 81.510 | 61.862 | 1.00 | 0.41 |
| ATOM | 4006 | CA | LEU | A | 287 | 52.316 | 79.689 | 61.006 | 1.00 | 0.41 |
| ATOM | 4007 | HA | LEU | A | 287 | 53.320 | 79.275 | 60.902 | 1.00 | 0.41 |
| ATOM | 4008 | CB | LEU | A | 287 | 51.588 | 78.841 | 62.069 | 1.00 | 0.41 |
| ATOM | 4009 | HB1 | LEU | A | 287 | 50.901 | 79.491 | 62.610 | 1.00 | 0.41 |
| ATOM | 4010 | HB2 | LEU | A | 287 | 52.338 | 78.504 | 62.785 | 1.00 | 0.41 |
| ATOM | 4011 | CG | LEU | A | 287 | 50.768 | 77.622 | 61.590 | 1.00 | 0.41 |
| ATOM | 4012 | HG | LEU | A | 287 | 49.888 | 77.975 | 61.052 | 1.00 | 0.41 |
| ATOM | 4013 | CD1 | LEU | A | 287 | 51.496 | 76.605 | 60.707 | 1.00 | 0.41 |
| ATOM | 4014 | 1HD1 | LEU | A | 287 | 50.884 | 75.711 | 60.588 | 1.00 | 0.41 |
| ATOM | 4015 | 2HD1 | LEU | A | 287 | 51.689 | 77.001 | 59.716 | 1.00 | 0.41 |
| ATOM | 4016 | 3HD1 | LEU | A | 287 | 52.444 | 76.336 | 61.168 | 1.00 | 0.41 |
| ATOM | 4017 | CD2 | LEU | A | 287 | 50.295 | 76.858 | 62.822 | 1.00 | 0.41 |
| ATOM | 4018 | 1HD2 | LEU | A | 287 | 49.535 | 76.135 | 62.535 | 1.00 | 0.41 |
| ATOM | 4019 | 2HD2 | LEU | A | 287 | 51.139 | 76.358 | 63.293 | 1.00 | 0.41 |
| ATOM | 4020 | 3HD2 | LEU | A | 287 | 49.872 | 77.547 | 63.544 | 1.00 | 0.41 |
| ATOM | 4021 | C | LEU | A | 287 | 51.666 | 79.678 | 59.627 | 1.00 | 0.41 |
| ATOM | 4022 | O | LEU | A | 287 | 50.529 | 80.106 | 59.415 | 1.00 | 0.41 |
| ATOM | 4023 | N | THR | A | 288 | 52.426 | 79.201 | 58.659 | 1.00 | 0.37 |
| ATOM | 4024 | H | THR | A | 288 | 53.090 | 78.448 | 58.845 | 1.00 | 0.37 |
| ATOM | 4025 | CA | THR | A | 288 | 52.425 | 79.707 | 57.300 | 1.00 | 0.37 |
| ATOM | 4026 | HA | THR | A | 288 | 51.506 | 80.251 | 57.106 | 1.00 | 0.37 |
| ATOM | 4027 | CB | THR | A | 288 | 53.599 | 80.665 | 57.079 | 1.00 | 0.37 |
| ATOM | 4028 | HB | THR | A | 288 | 54.534 | 80.161 | 57.307 | 1.00 | 0.37 |
| ATOM | 4029 | CG2 | THR | A | 288 | 53.619 | 81.237 | 55.667 | 1.00 | 0.37 |
| ATOM | 4030 | 1HG2 | THR | A | 288 | 52.667 | 81.712 | 55.437 | 1.00 | 0.37 |
| ATOM | 4031 | 2HG2 | THR | A | 288 | 54.423 | 81.968 | 55.585 | 1.00 | 0.37 |
| ATOM | 4032 | 3HG2 | THR | A | 288 | 53.807 | 80.439 | 54.955 | 1.00 | 0.37 |
| ATOM | 4033 | OG1 | THR | A | 288 | 53.454 | 81.773 | 57.936 | 1.00 | 0.37 |
| ATOM | 4034 | HG1 | THR | A | 288 | 53.903 | 81.560 | 58.764 | 1.00 | 0.37 |
| ATOM | 4035 | C | THR | A | 288 | 52.475 | 78.454 | 56.459 | 1.00 | 0.37 |
| ATOM | 4036 | O | THR | A | 288 | 53.307 | 77.572 | 56.665 | 1.00 | 0.37 |
| ATOM | 4037 | N | ILE | A | 289 | 51.441 | 78.276 | 55.661 | 1.00 | 0.39 |
| ATOM | 4038 | H | ILE | A | 289 | 50.831 | 79.063 | 55.465 | 1.00 | 0.39 |
| ATOM | 4039 | CA | ILE | A | 289 | 51.114 | 76.996 | 55.053 | 1.00 | 0.39 |
| ATOM | 4040 | HA | ILE | A | 289 | 51.989 | 76.348 | 55.027 | 1.00 | 0.39 |
| ATOM | 4041 | CB | ILE | A | 289 | 49.971 | 76.312 | 55.852 | 1.00 | 0.39 |
| ATOM | 4042 | HB | ILE | A | 289 | 49.473 | 77.072 | 56.449 | 1.00 | 0.39 |
| ATOM | 4043 | CG2 | ILE | A | 289 | 48.884 | 75.662 | 54.978 | 1.00 | 0.39 |
| ATOM | 4044 | 1HG2 | ILE | A | 289 | 49.335 | 74.939 | 54.304 | 1.00 | 0.39 |

| | | | | | | | | | | |
|---|---|---|---|---|---|---|---|---|---|---|
| ATOM | 4045 | 2HG2 | ILE | A | 289 | 48.131 | 75.175 | 55.595 | 1.00 | 0.39 |
| ATOM | 4046 | 3HG2 | ILE | A | 289 | 48.377 | 76.424 | 54.388 | 1.00 | 0.39 |
| ATOM | 4047 | CG1 | ILE | A | 289 | 50.617 | 75.300 | 56.820 | 1.00 | 0.39 |
| ATOM | 4048 | 1HG1 | ILE | A | 289 | 51.420 | 75.800 | 57.360 | 1.00 | 0.39 |
| ATOM | 4049 | 2HG1 | ILE | A | 289 | 51.053 | 74.492 | 56.235 | 1.00 | 0.39 |
| ATOM | 4050 | CD1 | ILE | A | 289 | 49.689 | 74.693 | 57.879 | 1.00 | 0.39 |
| ATOM | 4051 | HD1 | ILE | A | 289 | 50.275 | 74.053 | 58.536 | 1.00 | 0.39 |
| ATOM | 4052 | HD2 | ILE | A | 289 | 49.254 | 75.484 | 58.483 | 1.00 | 0.39 |
| ATOM | 4053 | HD3 | ILE | A | 289 | 48.904 | 74.098 | 57.416 | 1.00 | 0.39 |
| ATOM | 4054 | C | ILE | A | 289 | 50.770 | 77.358 | 53.616 | 1.00 | 0.39 |
| ATOM | 4055 | O | ILE | A | 289 | 49.928 | 78.221 | 53.380 | 1.00 | 0.39 |
| ATOM | 4056 | N | GLU | A | 290 | 51.513 | 76.810 | 52.665 | 1.00 | 0.41 |
| ATOM | 4057 | H | GLU | A | 290 | 52.023 | 75.944 | 52.853 | 1.00 | 0.41 |
| ATOM | 4058 | CA | GLU | A | 290 | 51.736 | 77.462 | 51.372 | 1.00 | 0.41 |
| ATOM | 4059 | HA | GLU | A | 290 | 50.937 | 78.170 | 51.148 | 1.00 | 0.41 |
| ATOM | 4060 | CB | GLU | A | 290 | 53.098 | 78.175 | 51.302 | 1.00 | 0.41 |
| ATOM | 4061 | HB1 | GLU | A | 290 | 53.134 | 78.750 | 50.375 | 1.00 | 0.41 |
| ATOM | 4062 | HB2 | GLU | A | 290 | 53.863 | 77.407 | 51.211 | 1.00 | 0.41 |
| ATOM | 4063 | CG | GLU | A | 290 | 53.501 | 79.102 | 52.461 | 1.00 | 0.41 |
| ATOM | 4064 | HG1 | GLU | A | 290 | 52.841 | 78.974 | 53.316 | 1.00 | 0.41 |
| ATOM | 4065 | HG2 | GLU | A | 290 | 53.415 | 80.141 | 52.136 | 1.00 | 0.41 |
| ATOM | 4066 | CD | GLU | A | 290 | 54.946 | 78.799 | 52.875 | 1.00 | 0.41 |
| ATOM | 4067 | OE1 | GLU | A | 290 | 55.841 | 78.929 | 52.004 | 1.00 | 0.41 |
| ATOM | 4068 | OE2 | GLU | A | 290 | 55.169 | 78.362 | 54.031 | 1.00 | 0.41 |
| ATOM | 4069 | C | GLU | A | 290 | 51.742 | 76.350 | 50.323 | 1.00 | 0.41 |
| ATOM | 4070 | O | GLU | A | 290 | 52.504 | 75.386 | 50.420 | 1.00 | 0.41 |
| ATOM | 4071 | N | ASN | A | 291 | 50.847 | 76.411 | 49.348 | 1.00 | 0.52 |
| ATOM | 4072 | H | ASN | A | 291 | 50.226 | 77.212 | 49.311 | 1.00 | 0.52 |
| ATOM | 4073 | CA | ASN | A | 291 | 50.300 | 75.170 | 48.825 | 1.00 | 0.52 |
| ATOM | 4074 | HA | ASN | A | 291 | 49.340 | 75.488 | 48.419 | 1.00 | 0.52 |
| ATOM | 4075 | CB | ASN | A | 291 | 51.064 | 74.599 | 47.617 | 1.00 | 0.52 |
| ATOM | 4076 | HB1 | ASN | A | 291 | 51.912 | 74.003 | 47.947 | 1.00 | 0.52 |
| ATOM | 4077 | HB2 | ASN | A | 291 | 51.420 | 75.421 | 46.996 | 1.00 | 0.52 |
| ATOM | 4078 | CG | ASN | A | 291 | 50.102 | 73.756 | 46.801 | 1.00 | 0.52 |
| ATOM | 4079 | OD1 | ASN | A | 291 | 50.085 | 72.535 | 46.885 | 1.00 | 0.52 |
| ATOM | 4080 | ND2 | ASN | A | 291 | 49.194 | 74.380 | 46.090 | 1.00 | 0.52 |
| ATOM | 4081 | 1HD2 | ASN | A | 291 | 49.202 | 75.408 | 46.090 | 1.00 | 0.52 |
| ATOM | 4082 | 2HD2 | ASN | A | 291 | 48.474 | 73.845 | 45.649 | 1.00 | 0.52 |
| ATOM | 4083 | C | ASN | A | 291 | 49.843 | 74.201 | 49.941 | 1.00 | 0.52 |
| ATOM | 4084 | O | ASN | A | 291 | 49.183 | 74.641 | 50.877 | 1.00 | 0.52 |
| ATOM | 4085 | N | ILE | A | 292 | 50.122 | 72.909 | 49.802 | 1.00 | 0.48 |
| ATOM | 4086 | H | ILE | A | 292 | 50.547 | 72.610 | 48.931 | 1.00 | 0.48 |
| ATOM | 4087 | CA | ILE | A | 292 | 50.106 | 71.819 | 50.781 | 1.00 | 0.48 |
| ATOM | 4088 | HA | ILE | A | 292 | 51.142 | 71.502 | 50.692 | 1.00 | 0.48 |

| | | | | | | | | | | |
|---|---|---|---|---|---|---|---|---|---|---|
| ATOM | 4089 | CB | ILE | A | 292 | 50.048 | 72.084 | 52.308 | 1.00 | 0.48 |
| ATOM | 4090 | HB | ILE | A | 292 | 50.416 | 71.162 | 52.759 | 1.00 | 0.48 |
| ATOM | 4091 | CG2 | ILE | A | 292 | 51.080 | 73.148 | 52.755 | 1.00 | 0.48 |
| ATOM | 4092 | 1HG2 | ILE | A | 292 | 51.301 | 73.052 | 53.815 | 1.00 | 0.48 |
| ATOM | 4093 | 2HG2 | ILE | A | 292 | 51.997 | 73.038 | 52.180 | 1.00 | 0.48 |
| ATOM | 4094 | 3HG2 | ILE | A | 292 | 50.697 | 74.149 | 52.594 | 1.00 | 0.48 |
| ATOM | 4095 | CG1 | ILE | A | 292 | 48.678 | 72.327 | 52.972 | 1.00 | 0.48 |
| ATOM | 4096 | 1HG1 | ILE | A | 292 | 48.090 | 72.988 | 52.352 | 1.00 | 0.48 |
| ATOM | 4097 | 2HG1 | ILE | A | 292 | 48.819 | 72.806 | 53.939 | 1.00 | 0.48 |
| ATOM | 4098 | CD1 | ILE | A | 292 | 47.898 | 71.037 | 53.230 | 1.00 | 0.48 |
| ATOM | 4099 | HD1 | ILE | A | 292 | 48.353 | 70.482 | 54.048 | 1.00 | 0.48 |
| ATOM | 4100 | HD2 | ILE | A | 292 | 46.869 | 71.275 | 53.481 | 1.00 | 0.48 |
| ATOM | 4101 | HD3 | ILE | A | 292 | 47.888 | 70.419 | 52.343 | 1.00 | 0.48 |
| ATOM | 4102 | C | ILE | A | 292 | 49.431 | 70.560 | 50.239 | 1.00 | 0.48 |
| ATOM | 4103 | O | ILE | A | 292 | 50.024 | 69.469 | 50.316 | 1.00 | 0.48 |
| ATOM | 4104 | N | SER | A | 293 | 48.334 | 70.754 | 49.524 | 1.00 | 0.65 |
| ATOM | 4105 | H | SER | A | 293 | 47.861 | 71.631 | 49.622 | 1.00 | 0.65 |
| ATOM | 4106 | CA | SER | A | 293 | 47.615 | 69.736 | 48.778 | 1.00 | 0.65 |
| ATOM | 4107 | HA | SER | A | 293 | 46.754 | 70.228 | 48.270 | 1.00 | 0.65 |
| ATOM | 4108 | CB | SER | A | 293 | 48.447 | 69.118 | 47.649 | 1.00 | 0.65 |
| ATOM | 4109 | HB1 | SER | A | 293 | 47.925 | 68.269 | 47.224 | 1.00 | 0.65 |
| ATOM | 4110 | HB2 | SER | A | 293 | 49.404 | 68.776 | 48.022 | 1.00 | 0.65 |
| ATOM | 4111 | OG | SER | A | 293 | 48.643 | 70.044 | 46.594 | 1.00 | 0.65 |
| ATOM | 4112 | HG | SER | A | 293 | 49.094 | 70.837 | 46.932 | 1.00 | 0.65 |
| ATOM | 4113 | C | SER | A | 293 | 46.929 | 68.608 | 49.764 | 1.00 | 0.65 |
| ATOM | 4114 | O | SER | A | 293 | 46.563 | 69.007 | 50.847 | 1.00 | 0.65 |
| ATOM | 4115 | N | GLY | A | 294 | 46.613 | 67.279 | 49.549 | 1.00 | 0.75 |
| ATOM | 4116 | H | GLY | A | 294 | 46.181 | 66.982 | 50.426 | 1.00 | 0.75 |
| ATOM | 4117 | CA | GLY | A | 294 | 46.792 | 66.157 | 48.540 | 1.00 | 0.75 |
| ATOM | 4118 | HA1 | GLY | A | 294 | 47.867 | 66.082 | 48.308 | 1.00 | 0.75 |
| ATOM | 4119 | HA2 | GLY | A | 294 | 46.291 | 66.475 | 47.610 | 1.00 | 0.75 |
| ATOM | 4120 | C | GLY | A | 294 | 46.343 | 64.730 | 48.831 | 1.00 | 0.75 |
| ATOM | 4121 | O | GLY | A | 294 | 46.996 | 63.796 | 48.469 | 1.00 | 0.75 |
| ATOM | 4122 | N | SER | A | 295 | 45.204 | 64.641 | 49.552 | 1.00 | 0.78 |
| ATOM | 4123 | H | SER | A | 295 | 44.784 | 65.502 | 49.879 | 1.00 | 0.78 |
| ATOM | 4124 | CA | SER | A | 295 | 44.308 | 63.479 | 49.705 | 1.00 | 0.78 |
| ATOM | 4125 | HA | SER | A | 295 | 43.315 | 63.931 | 49.686 | 1.00 | 0.78 |
| ATOM | 4126 | CB | SER | A | 295 | 44.281 | 62.473 | 48.538 | 1.00 | 0.78 |
| ATOM | 4127 | HB1 | SER | A | 295 | 44.152 | 63.019 | 47.601 | 1.00 | 0.78 |
| ATOM | 4128 | HB2 | SER | A | 295 | 43.412 | 61.825 | 48.663 | 1.00 | 0.78 |
| ATOM | 4129 | OG | SER | A | 295 | 45.428 | 61.660 | 48.441 | 1.00 | 0.78 |
| ATOM | 4130 | HG | SER | A | 295 | 46.176 | 62.284 | 48.387 | 1.00 | 0.78 |
| ATOM | 4131 | C | SER | A | 295 | 44.429 | 62.779 | 51.067 | 1.00 | 0.78 |
| ATOM | 4132 | O | SER | A | 295 | 45.373 | 62.042 | 51.386 | 1.00 | 0.78 |

| | | | | | | | | | | |
|---|---|---|---|---|---|---|---|---|---|---|
| ATOM | 4133 | N | GLY | A | 296 | 43.378 | 62.944 | 51.868 | 1.00 | 0.96 |
| ATOM | 4134 | H | GLY | A | 296 | 42.660 | 63.608 | 51.591 | 1.00 | 0.96 |
| ATOM | 4135 | CA | GLY | A | 296 | 43.009 | 61.975 | 52.895 | 1.00 | 0.96 |
| ATOM | 4136 | HA1 | GLY | A | 296 | 42.262 | 62.416 | 53.553 | 1.00 | 0.96 |
| ATOM | 4137 | HA2 | GLY | A | 296 | 43.888 | 61.736 | 53.493 | 1.00 | 0.96 |
| ATOM | 4138 | C | GLY | A | 296 | 42.435 | 60.676 | 52.328 | 1.00 | 0.96 |
| ATOM | 4139 | O | GLY | A | 296 | 42.176 | 60.549 | 51.129 | 1.00 | 0.96 |
| ATOM | 4140 | N | ALA | A | 297 | 42.247 | 59.698 | 53.208 | 1.00 | 1.10 |
| ATOM | 4141 | H | ALA | A | 297 | 42.542 | 59.840 | 54.167 | 1.00 | 1.10 |
| ATOM | 4142 | CA | ALA | A | 297 | 41.683 | 58.403 | 52.865 | 1.00 | 1.10 |
| ATOM | 4143 | HA | ALA | A | 297 | 42.284 | 57.981 | 52.058 | 1.00 | 1.10 |
| ATOM | 4144 | CB | ALA | A | 297 | 41.834 | 57.482 | 54.077 | 1.00 | 1.10 |
| ATOM | 4145 | HB1 | ALA | A | 297 | 41.422 | 56.502 | 53.844 | 1.00 | 1.10 |
| ATOM | 4146 | HB2 | ALA | A | 297 | 42.888 | 57.369 | 54.330 | 1.00 | 1.10 |
| ATOM | 4147 | HB3 | ALA | A | 297 | 41.307 | 57.901 | 54.936 | 1.00 | 1.10 |
| ATOM | 4148 | C | ALA | A | 297 | 40.240 | 58.500 | 52.365 | 1.00 | 1.10 |
| ATOM | 4149 | O | ALA | A | 297 | 39.405 | 59.159 | 52.975 | 1.00 | 1.10 |
| ATOM | 4150 | N | VAL | A | 298 | 39.959 | 57.793 | 51.261 | 1.00 | 2.38 |
| ATOM | 4151 | H | VAL | A | 298 | 40.708 | 57.286 | 50.819 | 1.00 | 2.38 |
| ATOM | 4152 | CA | VAL | A | 298 | 38.582 | 57.533 | 50.806 | 1.00 | 2.38 |
| ATOM | 4153 | HA | VAL | A | 298 | 38.067 | 58.493 | 50.733 | 1.00 | 2.38 |
| ATOM | 4154 | CB | VAL | A | 298 | 38.533 | 56.855 | 49.421 | 1.00 | 2.38 |
| ATOM | 4155 | HB | VAL | A | 298 | 37.493 | 56.622 | 49.190 | 1.00 | 2.38 |
| ATOM | 4156 | CG1 | VAL | A | 298 | 39.043 | 57.802 | 48.326 | 1.00 | 2.38 |
| ATOM | 4157 | 1HG1 | VAL | A | 298 | 38.941 | 57.328 | 47.350 | 1.00 | 2.38 |
| ATOM | 4158 | 2HG1 | VAL | A | 298 | 38.454 | 58.720 | 48.341 | 1.00 | 2.38 |
| ATOM | 4159 | 3HG1 | VAL | A | 298 | 40.090 | 58.052 | 48.500 | 1.00 | 2.38 |
| ATOM | 4160 | CG2 | VAL | A | 298 | 39.342 | 55.553 | 49.335 | 1.00 | 2.38 |
| ATOM | 4161 | 1HG2 | VAL | A | 298 | 39.222 | 55.109 | 48.348 | 1.00 | 2.38 |
| ATOM | 4162 | 2HG2 | VAL | A | 298 | 40.402 | 55.735 | 49.511 | 1.00 | 2.38 |
| ATOM | 4163 | 3HG2 | VAL | A | 298 | 38.983 | 54.835 | 50.073 | 1.00 | 2.38 |
| ATOM | 4164 | C | VAL | A | 298 | 37.803 | 56.712 | 51.831 | 1.00 | 2.38 |
| ATOM | 4165 | O | VAL | A | 298 | 36.604 | 56.931 | 51.996 | 1.00 | 2.38 |
| ATOM | 4166 | N | ASP | A | 299 | 38.502 | 55.851 | 52.575 | 1.00 | 2.51 |
| ATOM | 4167 | H | ASP | A | 299 | 39.427 | 55.587 | 52.261 | 1.00 | 2.51 |
| ATOM | 4168 | CA | ASP | A | 299 | 38.180 | 55.426 | 53.936 | 1.00 | 2.51 |
| ATOM | 4169 | HA | ASP | A | 299 | 37.214 | 54.918 | 53.909 | 1.00 | 2.51 |
| ATOM | 4170 | CB | ASP | A | 299 | 39.205 | 54.383 | 54.426 | 1.00 | 2.51 |
| ATOM | 4171 | HB1 | ASP | A | 299 | 38.833 | 53.934 | 55.349 | 1.00 | 2.51 |
| ATOM | 4172 | HB2 | ASP | A | 299 | 40.149 | 54.874 | 54.658 | 1.00 | 2.51 |
| ATOM | 4173 | CG | ASP | A | 299 | 39.470 | 53.280 | 53.386 | 1.00 | 2.51 |
| ATOM | 4174 | OD1 | ASP | A | 299 | 40.246 | 53.565 | 52.440 | 1.00 | 2.51 |
| ATOM | 4175 | OD2 | ASP | A | 299 | 38.894 | 52.175 | 53.515 | 1.00 | 2.51 |
| ATOM | 4176 | C | ASP | A | 299 | 38.026 | 56.662 | 54.840 | 1.00 | 2.51 |

| | | | | | | | | | | |
|---|---|---|---|---|---|---|---|---|---|---|
| ATOM | 4177 | O | ASP | A | 299 | 38.960 | 57.123 | 55.502 | 1.00 | 2.51 |
| ATOM | 4178 | N | SER | A | 300 | 36.847 | 57.284 | 54.729 | 1.00 | 2.92 |
| ATOM | 4179 | H | SER | A | 300 | 36.165 | 56.843 | 54.129 | 1.00 | 2.92 |
| ATOM | 4180 | CA | SER | A | 300 | 36.572 | 58.670 | 55.140 | 1.00 | 2.92 |
| ATOM | 4181 | HA | SER | A | 300 | 37.301 | 59.321 | 54.657 | 1.00 | 2.92 |
| ATOM | 4182 | CB | SER | A | 300 | 35.173 | 59.087 | 54.673 | 1.00 | 2.92 |
| ATOM | 4183 | HB1 | SER | A | 300 | 35.015 | 60.141 | 54.909 | 1.00 | 2.92 |
| ATOM | 4184 | HB2 | SER | A | 300 | 34.430 | 58.498 | 55.214 | 1.00 | 2.92 |
| ATOM | 4185 | OG | SER | A | 300 | 34.984 | 58.892 | 53.278 | 1.00 | 2.92 |
| ATOM | 4186 | HG | SER | A | 300 | 35.682 | 58.308 | 52.934 | 1.00 | 2.92 |
| ATOM | 4187 | C | SER | A | 300 | 36.691 | 58.908 | 56.648 | 1.00 | 2.92 |
| ATOM | 4188 | O | SER | A | 300 | 36.591 | 60.042 | 57.105 | 1.00 | 2.92 |
| ATOM | 4189 | N | ASP | A | 301 | 36.958 | 57.839 | 57.399 | 1.00 | 2.18 |
| ATOM | 4190 | H | ASP | A | 301 | 36.921 | 56.933 | 56.944 | 1.00 | 2.18 |
| ATOM | 4191 | CA | ASP | A | 301 | 37.585 | 57.821 | 58.716 | 1.00 | 2.18 |
| ATOM | 4192 | HA | ASP | A | 301 | 36.824 | 58.087 | 59.450 | 1.00 | 2.18 |
| ATOM | 4193 | CB | ASP | A | 301 | 38.041 | 56.385 | 59.018 | 1.00 | 2.18 |
| ATOM | 4194 | HB1 | ASP | A | 301 | 38.428 | 56.342 | 60.038 | 1.00 | 2.18 |
| ATOM | 4195 | HB2 | ASP | A | 301 | 38.851 | 56.114 | 58.338 | 1.00 | 2.18 |
| ATOM | 4196 | CG | ASP | A | 301 | 36.894 | 55.377 | 58.860 | 1.00 | 2.18 |
| ATOM | 4197 | OD1 | ASP | A | 301 | 36.595 | 55.017 | 57.695 | 1.00 | 2.18 |
| ATOM | 4198 | OD2 | ASP | A | 301 | 36.300 | 54.993 | 59.895 | 1.00 | 2.18 |
| ATOM | 4199 | C | ASP | A | 301 | 38.719 | 58.842 | 58.868 | 1.00 | 2.18 |
| ATOM | 4200 | O | ASP | A | 301 | 38.884 | 59.443 | 59.934 | 1.00 | 2.18 |
| ATOM | 4201 | N | GLY | A | 302 | 39.451 | 59.101 | 57.781 | 1.00 | 1.43 |
| ATOM | 4202 | H | GLY | A | 302 | 39.282 | 58.551 | 56.944 | 1.00 | 1.43 |
| ATOM | 4203 | CA | GLY | A | 302 | 40.435 | 60.175 | 57.756 | 1.00 | 1.43 |
| ATOM | 4204 | HA1 | GLY | A | 302 | 41.124 | 59.998 | 56.932 | 1.00 | 1.43 |
| ATOM | 4205 | HA2 | GLY | A | 302 | 41.008 | 60.129 | 58.682 | 1.00 | 1.43 |
| ATOM | 4206 | C | GLY | A | 302 | 39.876 | 61.576 | 57.609 | 1.00 | 1.43 |
| ATOM | 4207 | O | GLY | A | 302 | 38.999 | 61.875 | 56.802 | 1.00 | 1.43 |
| ATOM | 4208 | N | TYR | A | 303 | 40.491 | 62.484 | 58.345 | 1.00 | 0.82 |
| ATOM | 4209 | H | TYR | A | 303 | 41.245 | 62.161 | 58.943 | 1.00 | 0.82 |
| ATOM | 4210 | CA | TYR | A | 303 | 39.883 | 63.727 | 58.751 | 1.00 | 0.82 |
| ATOM | 4211 | HA | TYR | A | 303 | 38.896 | 63.810 | 58.292 | 1.00 | 0.82 |
| ATOM | 4212 | CB | TYR | A | 303 | 39.692 | 63.531 | 60.275 | 1.00 | 0.82 |
| ATOM | 4213 | HB1 | TYR | A | 303 | 40.634 | 63.331 | 60.776 | 1.00 | 0.82 |
| ATOM | 4214 | HB2 | TYR | A | 303 | 39.014 | 62.696 | 60.464 | 1.00 | 0.82 |
| ATOM | 4215 | CG | TYR | A | 303 | 39.112 | 64.771 | 60.809 | 1.00 | 0.82 |
| ATOM | 4216 | CD1 | TYR | A | 303 | 37.851 | 65.071 | 60.300 | 1.00 | 0.82 |
| ATOM | 4217 | HD1 | TYR | A | 303 | 37.139 | 64.280 | 60.117 | 1.00 | 0.82 |
| ATOM | 4218 | CE1 | TYR | A | 303 | 37.661 | 66.326 | 59.726 | 1.00 | 0.82 |
| ATOM | 4219 | HE1 | TYR | A | 303 | 36.745 | 66.514 | 59.219 | 1.00 | 0.82 |
| ATOM | 4220 | CZ | TYR | A | 303 | 38.680 | 67.296 | 59.759 | 1.00 | 0.82 |

| | | | | | | | | | | |
|---|---|---|---|---|---|---|---|---|---|---|
| ATOM | 4221 | OH | TYR | A | 303 | 38.517 | 68.494 | 59.138 | 1.00 | 0.82 |
| ATOM | 4222 | HH | TYR | A | 303 | 39.100 | 69.209 | 59.455 | 1.00 | 0.82 |
| ATOM | 4223 | CE2 | TYR | A | 303 | 39.844 | 67.017 | 60.494 | 1.00 | 0.82 |
| ATOM | 4224 | HE2 | TYR | A | 303 | 40.654 | 67.688 | 60.526 | 1.00 | 0.82 |
| ATOM | 4225 | CD2 | TYR | A | 303 | 40.053 | 65.764 | 61.057 | 1.00 | 0.82 |
| ATOM | 4226 | HD2 | TYR | A | 303 | 41.039 | 65.497 | 61.400 | 1.00 | 0.82 |
| ATOM | 4227 | C | TYR | A | 303 | 40.758 | 64.897 | 58.205 | 1.00 | 0.82 |
| ATOM | 4228 | O | TYR | A | 303 | 41.988 | 64.924 | 58.321 | 1.00 | 0.82 |
| ATOM | 4229 | N | ASN | A | 304 | 40.177 | 65.828 | 57.437 | 1.00 | 0.57 |
| ATOM | 4230 | H | ASN | A | 304 | 39.159 | 65.833 | 57.359 | 1.00 | 0.57 |
| ATOM | 4231 | CA | ASN | A | 304 | 40.946 | 66.634 | 56.466 | 1.00 | 0.57 |
| ATOM | 4232 | HA | ASN | A | 304 | 41.693 | 65.983 | 56.012 | 1.00 | 0.57 |
| ATOM | 4233 | CB | ASN | A | 304 | 39.999 | 67.134 | 55.350 | 1.00 | 0.57 |
| ATOM | 4234 | HB1 | ASN | A | 304 | 40.397 | 68.064 | 54.944 | 1.00 | 0.57 |
| ATOM | 4235 | HB2 | ASN | A | 304 | 39.014 | 67.368 | 55.747 | 1.00 | 0.57 |
| ATOM | 4236 | CG | ASN | A | 304 | 39.852 | 66.204 | 54.154 | 1.00 | 0.57 |
| ATOM | 4237 | OD1 | ASN | A | 304 | 39.327 | 66.576 | 53.123 | 1.00 | 0.57 |
| ATOM | 4238 | ND2 | ASN | A | 304 | 40.383 | 65.002 | 54.184 | 1.00 | 0.57 |
| ATOM | 4239 | 1HD2 | ASN | A | 304 | 40.637 | 64.579 | 55.055 | 1.00 | 0.57 |
| ATOM | 4240 | 2HD2 | ASN | A | 304 | 40.409 | 64.521 | 53.297 | 1.00 | 0.57 |
| ATOM | 4241 | C | ASN | A | 304 | 41.788 | 67.802 | 56.987 | 1.00 | 0.57 |
| ATOM | 4242 | O | ASN | A | 304 | 42.848 | 68.067 | 56.409 | 1.00 | 0.57 |
| ATOM | 4243 | N | ILE | A | 305 | 41.331 | 68.508 | 58.023 | 1.00 | 0.69 |
| ATOM | 4244 | H | ILE | A | 305 | 40.402 | 68.277 | 58.349 | 1.00 | 0.69 |
| ATOM | 4245 | CA | ILE | A | 305 | 42.036 | 69.604 | 58.717 | 1.00 | 0.69 |
| ATOM | 4246 | HA | ILE | A | 305 | 43.090 | 69.358 | 58.802 | 1.00 | 0.69 |
| ATOM | 4247 | CB | ILE | A | 305 | 41.906 | 70.951 | 57.930 | 1.00 | 0.69 |
| ATOM | 4248 | HB | ILE | A | 305 | 40.851 | 71.182 | 57.875 | 1.00 | 0.69 |
| ATOM | 4249 | CG2 | ILE | A | 305 | 42.587 | 72.107 | 58.693 | 1.00 | 0.69 |
| ATOM | 4250 | 1HG2 | ILE | A | 305 | 42.303 | 73.054 | 58.242 | 1.00 | 0.69 |
| ATOM | 4251 | 2HG2 | ILE | A | 305 | 42.258 | 72.160 | 59.727 | 1.00 | 0.69 |
| ATOM | 4252 | 3HG2 | ILE | A | 305 | 43.666 | 71.996 | 58.656 | 1.00 | 0.69 |
| ATOM | 4253 | CG1 | ILE | A | 305 | 42.393 | 70.933 | 56.461 | 1.00 | 0.69 |
| ATOM | 4254 | 1HG1 | ILE | A | 305 | 41.746 | 70.264 | 55.896 | 1.00 | 0.69 |
| ATOM | 4255 | 2HG1 | ILE | A | 305 | 43.413 | 70.557 | 56.424 | 1.00 | 0.69 |
| ATOM | 4256 | CD1 | ILE | A | 305 | 42.354 | 72.272 | 55.712 | 1.00 | 0.69 |
| ATOM | 4257 | HD1 | ILE | A | 305 | 42.443 | 72.090 | 54.641 | 1.00 | 0.69 |
| ATOM | 4258 | HD2 | ILE | A | 305 | 41.418 | 72.790 | 55.914 | 1.00 | 0.69 |
| ATOM | 4259 | HD3 | ILE | A | 305 | 43.188 | 72.898 | 56.019 | 1.00 | 0.69 |
| ATOM | 4260 | C | ILE | A | 305 | 41.441 | 69.830 | 60.104 | 1.00 | 0.69 |
| ATOM | 4261 | O | ILE | A | 305 | 40.272 | 70.187 | 60.206 | 1.00 | 0.69 |
| ATOM | 4262 | N | VAL | A | 306 | 42.183 | 69.583 | 61.188 | 1.00 | 0.81 |
| ATOM | 4263 | H | VAL | A | 306 | 43.174 | 69.371 | 61.104 | 1.00 | 0.81 |
| ATOM | 4264 | CA | VAL | A | 306 | 41.679 | 69.792 | 62.550 | 1.00 | 0.81 |

| | | | | | | | | | | |
|---|---|---|---|---|---|---|---|---|---|---|
| ATOM | 4265 | HA | VAL | A | 306 | 40.710 | 70.287 | 62.489 | 1.00 | 0.81 |
| ATOM | 4266 | CB | VAL | A | 306 | 41.442 | 68.479 | 63.323 | 1.00 | 0.81 |
| ATOM | 4267 | HB | VAL | A | 306 | 41.082 | 67.728 | 62.634 | 1.00 | 0.81 |
| ATOM | 4268 | CG1 | VAL | A | 306 | 42.685 | 67.899 | 63.980 | 1.00 | 0.81 |
| ATOM | 4269 | 1HG1 | VAL | A | 306 | 42.473 | 66.910 | 64.384 | 1.00 | 0.81 |
| ATOM | 4270 | 2HG1 | VAL | A | 306 | 43.473 | 67.827 | 63.233 | 1.00 | 0.81 |
| ATOM | 4271 | 3HG1 | VAL | A | 306 | 43.008 | 68.545 | 64.796 | 1.00 | 0.81 |
| ATOM | 4272 | CG2 | VAL | A | 306 | 40.346 | 68.663 | 64.369 | 1.00 | 0.81 |
| ATOM | 4273 | 1HG2 | VAL | A | 306 | 40.172 | 67.725 | 64.897 | 1.00 | 0.81 |
| ATOM | 4274 | 2HG2 | VAL | A | 306 | 40.629 | 69.427 | 65.088 | 1.00 | 0.81 |
| ATOM | 4275 | 3HG2 | VAL | A | 306 | 39.416 | 68.963 | 63.883 | 1.00 | 0.81 |
| ATOM | 4276 | C | VAL | A | 306 | 42.623 | 70.765 | 63.228 | 1.00 | 0.81 |
| ATOM | 4277 | O | VAL | A | 306 | 43.843 | 70.653 | 63.129 | 1.00 | 0.81 |
| ATOM | 4278 | N | ILE | A | 307 | 42.041 | 71.781 | 63.843 | 1.00 | 0.63 |
| ATOM | 4279 | H | ILE | A | 307 | 41.036 | 71.776 | 63.945 | 1.00 | 0.63 |
| ATOM | 4280 | CA | ILE | A | 307 | 42.753 | 72.886 | 64.476 | 1.00 | 0.63 |
| ATOM | 4281 | HA | ILE | A | 307 | 43.818 | 72.673 | 64.550 | 1.00 | 0.63 |
| ATOM | 4282 | CB | ILE | A | 307 | 42.556 | 74.202 | 63.698 | 1.00 | 0.63 |
| ATOM | 4283 | HB | ILE | A | 307 | 41.482 | 74.356 | 63.593 | 1.00 | 0.63 |
| ATOM | 4284 | CG2 | ILE | A | 307 | 43.146 | 75.372 | 64.516 | 1.00 | 0.63 |
| ATOM | 4285 | 1HG2 | ILE | A | 307 | 43.230 | 76.274 | 63.914 | 1.00 | 0.63 |
| ATOM | 4286 | 2HG2 | ILE | A | 307 | 42.490 | 75.602 | 65.358 | 1.00 | 0.63 |
| ATOM | 4287 | 3HG2 | ILE | A | 307 | 44.129 | 75.108 | 64.904 | 1.00 | 0.63 |
| ATOM | 4288 | CG1 | ILE | A | 307 | 43.166 | 74.138 | 62.281 | 1.00 | 0.63 |
| ATOM | 4289 | 1HG1 | ILE | A | 307 | 42.912 | 73.188 | 61.814 | 1.00 | 0.63 |
| ATOM | 4290 | 2HG1 | ILE | A | 307 | 44.252 | 74.196 | 62.350 | 1.00 | 0.63 |
| ATOM | 4291 | CD1 | ILE | A | 307 | 42.646 | 75.244 | 61.352 | 1.00 | 0.63 |
| ATOM | 4292 | HD1 | ILE | A | 307 | 43.032 | 75.088 | 60.347 | 1.00 | 0.63 |
| ATOM | 4293 | HD2 | ILE | A | 307 | 41.558 | 75.209 | 61.308 | 1.00 | 0.63 |
| ATOM | 4294 | HD3 | ILE | A | 307 | 42.962 | 76.227 | 61.698 | 1.00 | 0.63 |
| ATOM | 4295 | C | ILE | A | 307 | 42.180 | 72.999 | 65.874 | 1.00 | 0.63 |
| ATOM | 4296 | O | ILE | A | 307 | 40.959 | 73.107 | 65.938 | 1.00 | 0.63 |
| ATOM | 4297 | N | VAL | A | 308 | 42.960 | 72.927 | 66.968 | 1.00 | 0.64 |
| ATOM | 4298 | H | VAL | A | 308 | 43.963 | 72.769 | 66.906 | 1.00 | 0.64 |
| ATOM | 4299 | CA | VAL | A | 308 | 42.277 | 72.766 | 68.251 | 1.00 | 0.64 |
| ATOM | 4300 | HA | VAL | A | 308 | 41.260 | 73.153 | 68.201 | 1.00 | 0.64 |
| ATOM | 4301 | CB | VAL | A | 308 | 42.192 | 71.284 | 68.719 | 1.00 | 0.64 |
| ATOM | 4302 | HB | VAL | A | 308 | 43.066 | 71.018 | 69.312 | 1.00 | 0.64 |
| ATOM | 4303 | CG1 | VAL | A | 308 | 40.920 | 71.074 | 69.554 | 1.00 | 0.64 |
| ATOM | 4304 | 1HG1 | VAL | A | 308 | 40.931 | 70.081 | 70.005 | 1.00 | 0.64 |
| ATOM | 4305 | 2HG1 | VAL | A | 308 | 40.867 | 71.808 | 70.353 | 1.00 | 0.64 |
| ATOM | 4306 | 3HG1 | VAL | A | 308 | 40.027 | 71.173 | 68.935 | 1.00 | 0.64 |
| ATOM | 4307 | CG2 | VAL | A | 308 | 42.095 | 70.261 | 67.577 | 1.00 | 0.64 |
| ATOM | 4308 | 1HG2 | VAL | A | 308 | 42.000 | 69.257 | 67.990 | 1.00 | 0.64 |

| | | | | | | | | | | |
|---|---|---|---|---|---|---|---|---|---|---|
| ATOM | 4309 | 2HG2 | VAL | A | 308 | 41.223 | 70.481 | 66.963 | 1.00 | 0.64 |
| ATOM | 4310 | 3HG2 | VAL | A | 308 | 42.997 | 70.293 | 66.964 | 1.00 | 0.64 |
| ATOM | 4311 | C | VAL | A | 308 | 42.998 | 73.691 | 69.205 | 1.00 | 0.64 |
| ATOM | 4312 | O | VAL | A | 308 | 43.639 | 73.254 | 70.147 | 1.00 | 0.64 |
| ATOM | 4313 | N | CYS | A | 309 | 42.985 | 74.978 | 68.858 | 1.00 | 0.88 |
| ATOM | 4314 | H | CYS | A | 309 | 42.352 | 75.276 | 68.128 | 1.00 | 0.88 |
| ATOM | 4315 | CA | CYS | A | 309 | 43.699 | 76.003 | 69.588 | 1.00 | 0.88 |
| ATOM | 4316 | HA | CYS | A | 309 | 44.676 | 75.603 | 69.857 | 1.00 | 0.88 |
| ATOM | 4317 | CB | CYS | A | 309 | 43.906 | 77.241 | 68.694 | 1.00 | 0.88 |
| ATOM | 4318 | HB1 | CYS | A | 309 | 43.831 | 78.136 | 69.312 | 1.00 | 0.88 |
| ATOM | 4319 | HB2 | CYS | A | 309 | 43.105 | 77.293 | 67.954 | 1.00 | 0.88 |
| ATOM | 4320 | SG | CYS | A | 309 | 45.519 | 77.307 | 67.859 | 1.00 | 0.88 |
| ATOM | 4321 | C | CYS | A | 309 | 42.970 | 76.390 | 70.876 | 1.00 | 0.88 |
| ATOM | 4322 | O | CYS | A | 309 | 41.741 | 76.446 | 70.933 | 1.00 | 0.88 |
| ATOM | 4323 | N | GLY | A | 310 | 43.749 | 76.763 | 71.885 | 1.00 | 0.87 |
| ATOM | 4324 | H | GLY | A | 310 | 44.747 | 76.691 | 71.745 | 1.00 | 0.87 |
| ATOM | 4325 | CA | GLY | A | 310 | 43.293 | 77.521 | 73.030 | 1.00 | 0.87 |
| ATOM | 4326 | HA1 | GLY | A | 310 | 44.084 | 77.576 | 73.777 | 1.00 | 0.87 |
| ATOM | 4327 | HA2 | GLY | A | 310 | 42.436 | 77.028 | 73.477 | 1.00 | 0.87 |
| ATOM | 4328 | C | GLY | A | 310 | 42.880 | 78.946 | 72.660 | 1.00 | 0.87 |
| ATOM | 4329 | O | GLY | A | 310 | 43.310 | 79.523 | 71.655 | 1.00 | 0.87 |
| ATOM | 4330 | N | ASP | A | 311 | 42.084 | 79.518 | 73.549 | 1.00 | 0.97 |
| ATOM | 4331 | H | ASP | A | 311 | 41.938 | 78.968 | 74.393 | 1.00 | 0.97 |
| ATOM | 4332 | CA | ASP | A | 311 | 41.930 | 80.952 | 73.771 | 1.00 | 0.97 |
| ATOM | 4333 | HA | ASP | A | 311 | 41.085 | 81.321 | 73.191 | 1.00 | 0.97 |
| ATOM | 4334 | CB | ASP | A | 311 | 41.614 | 81.113 | 75.282 | 1.00 | 0.97 |
| ATOM | 4335 | HB1 | ASP | A | 311 | 40.633 | 80.676 | 75.476 | 1.00 | 0.97 |
| ATOM | 4336 | HB2 | ASP | A | 311 | 41.546 | 82.177 | 75.515 | 1.00 | 0.97 |
| ATOM | 4337 | CG | ASP | A | 311 | 42.643 | 80.438 | 76.232 | 1.00 | 0.97 |
| ATOM | 4338 | OD1 | ASP | A | 311 | 42.771 | 79.191 | 76.168 | 1.00 | 0.97 |
| ATOM | 4339 | OD2 | ASP | A | 311 | 43.278 | 81.137 | 77.056 | 1.00 | 0.97 |
| ATOM | 4340 | C | ASP | A | 311 | 43.178 | 81.798 | 73.445 | 1.00 | 0.97 |
| ATOM | 4341 | O | ASP | A | 311 | 44.231 | 81.628 | 74.047 | 1.00 | 0.97 |
| ATOM | 4342 | N | ASP | A | 312 | 43.050 | 82.698 | 72.466 | 1.00 | 1.15 |
| ATOM | 4343 | H | ASP | A | 312 | 42.123 | 82.803 | 72.093 | 1.00 | 1.15 |
| ATOM | 4344 | CA | ASP | A | 312 | 44.096 | 83.534 | 71.834 | 1.00 | 1.15 |
| ATOM | 4345 | HA | ASP | A | 312 | 43.656 | 83.859 | 70.892 | 1.00 | 1.15 |
| ATOM | 4346 | CB | ASP | A | 312 | 44.325 | 84.814 | 72.658 | 1.00 | 1.15 |
| ATOM | 4347 | HB1 | ASP | A | 312 | 45.042 | 84.607 | 73.455 | 1.00 | 1.15 |
| ATOM | 4348 | HB2 | ASP | A | 312 | 43.383 | 85.118 | 73.120 | 1.00 | 1.15 |
| ATOM | 4349 | CG | ASP | A | 312 | 44.813 | 85.979 | 71.785 | 1.00 | 1.15 |
| ATOM | 4350 | OD1 | ASP | A | 312 | 45.763 | 86.675 | 72.208 | 1.00 | 1.15 |
| ATOM | 4351 | OD2 | ASP | A | 312 | 44.195 | 86.182 | 70.711 | 1.00 | 1.15 |
| ATOM | 4352 | C | ASP | A | 312 | 45.421 | 82.852 | 71.415 | 1.00 | 1.15 |

| | | | | | | | | | | |
|---|---|---|---|---|---|---|---|---|---|---|
| ATOM | 4353 | O | ASP | A | 312 | 46.265 | 83.448 | 70.745 | 1.00 | 1.15 |
| ATOM | 4354 | N | ALA | A | 313 | 45.593 | 81.561 | 71.708 | 1.00 | 0.89 |
| ATOM | 4355 | H | ALA | A | 313 | 44.846 | 81.116 | 72.230 | 1.00 | 0.89 |
| ATOM | 4356 | CA | ALA | A | 313 | 46.889 | 80.892 | 71.746 | 1.00 | 0.89 |
| ATOM | 4357 | HA | ALA | A | 313 | 47.601 | 81.584 | 72.199 | 1.00 | 0.89 |
| ATOM | 4358 | CB | ALA | A | 313 | 46.747 | 79.693 | 72.681 | 1.00 | 0.89 |
| ATOM | 4359 | HB1 | ALA | A | 313 | 47.718 | 79.231 | 72.852 | 1.00 | 0.89 |
| ATOM | 4360 | HB2 | ALA | A | 313 | 46.357 | 80.037 | 73.635 | 1.00 | 0.89 |
| ATOM | 4361 | HB3 | ALA | A | 313 | 46.048 | 78.973 | 72.260 | 1.00 | 0.89 |
| ATOM | 4362 | C | ALA | A | 313 | 47.451 | 80.531 | 70.353 | 1.00 | 0.89 |
| ATOM | 4363 | O | ALA | A | 313 | 48.329 | 79.674 | 70.223 | 1.00 | 0.89 |
| ATOM | 4364 | N | CYS | A | 314 | 46.943 | 81.166 | 69.296 | 1.00 | 0.70 |
| ATOM | 4365 | H | CYS | A | 314 | 46.322 | 81.940 | 69.509 | 1.00 | 0.70 |
| ATOM | 4366 | CA | CYS | A | 314 | 47.026 | 80.682 | 67.927 | 1.00 | 0.70 |
| ATOM | 4367 | HA | CYS | A | 314 | 47.576 | 79.740 | 67.914 | 1.00 | 0.70 |
| ATOM | 4368 | CB | CYS | A | 314 | 45.584 | 80.374 | 67.478 | 1.00 | 0.70 |
| ATOM | 4369 | HB1 | CYS | A | 314 | 45.204 | 81.203 | 66.880 | 1.00 | 0.70 |
| ATOM | 4370 | HB2 | CYS | A | 314 | 44.937 | 80.290 | 68.354 | 1.00 | 0.70 |
| ATOM | 4371 | SG | CYS | A | 314 | 45.358 | 78.848 | 66.535 | 1.00 | 0.70 |
| ATOM | 4372 | C | CYS | A | 314 | 47.770 | 81.656 | 66.998 | 1.00 | 0.70 |
| ATOM | 4373 | O | CYS | A | 314 | 48.797 | 81.277 | 66.439 | 1.00 | 0.70 |
| ATOM | 4374 | N | SER | A | 315 | 47.346 | 82.926 | 66.960 | 1.00 | 0.72 |
| ATOM | 4375 | H | SER | A | 315 | 46.514 | 83.148 | 67.485 | 1.00 | 0.72 |
| ATOM | 4376 | CA | SER | A | 315 | 48.023 | 84.067 | 66.316 | 1.00 | 0.72 |
| ATOM | 4377 | HA | SER | A | 315 | 47.351 | 84.906 | 66.497 | 1.00 | 0.72 |
| ATOM | 4378 | CB | SER | A | 315 | 49.309 | 84.433 | 67.083 | 1.00 | 0.72 |
| ATOM | 4379 | HB1 | SER | A | 315 | 50.163 | 84.414 | 66.415 | 1.00 | 0.72 |
| ATOM | 4380 | HB2 | SER | A | 315 | 49.489 | 83.730 | 67.898 | 1.00 | 0.72 |
| ATOM | 4381 | OG | SER | A | 315 | 49.216 | 85.737 | 67.613 | 1.00 | 0.72 |
| ATOM | 4382 | HG | SER | A | 315 | 48.698 | 85.717 | 68.428 | 1.00 | 0.72 |
| ATOM | 4383 | C | SER | A | 315 | 48.132 | 84.078 | 64.788 | 1.00 | 0.72 |
| ATOM | 4384 | O | SER | A | 315 | 47.346 | 83.431 | 64.100 | 1.00 | 0.72 |
| ATOM | 4385 | N | ASN | A | 316 | 49.008 | 84.939 | 64.269 | 1.00 | 0.60 |
| ATOM | 4386 | H | ASN | A | 316 | 49.609 | 85.410 | 64.933 | 1.00 | 0.60 |
| ATOM | 4387 | CA | ASN | A | 316 | 49.295 | 85.257 | 62.868 | 1.00 | 0.60 |
| ATOM | 4388 | HA | ASN | A | 316 | 48.441 | 85.801 | 62.463 | 1.00 | 0.60 |
| ATOM | 4389 | CB | ASN | A | 316 | 50.540 | 86.194 | 62.811 | 1.00 | 0.60 |
| ATOM | 4390 | HB1 | ASN | A | 316 | 50.535 | 86.747 | 61.874 | 1.00 | 0.60 |
| ATOM | 4391 | HB2 | ASN | A | 316 | 51.447 | 85.590 | 62.811 | 1.00 | 0.60 |
| ATOM | 4392 | CG | ASN | A | 316 | 50.703 | 87.183 | 63.953 | 1.00 | 0.60 |
| ATOM | 4393 | OD1 | ASN | A | 316 | 49.769 | 87.859 | 64.355 | 1.00 | 0.60 |
| ATOM | 4394 | ND2 | ASN | A | 316 | 51.879 | 87.278 | 64.528 | 1.00 | 0.60 |
| ATOM | 4395 | 1HD2 | ASN | A | 316 | 52.656 | 86.671 | 64.236 | 1.00 | 0.60 |
| ATOM | 4396 | 2HD2 | ASN | A | 316 | 51.960 | 87.891 | 65.311 | 1.00 | 0.60 |

| | | | | | | | | | | |
|---|---|---|---|---|---|---|---|---|---|---|
| ATOM | 4397 | C | ASN | A | 316 | 49.532 | 84.012 | 61.989 | 1.00 | 0.60 |
| ATOM | 4398 | O | ASN | A | 316 | 50.655 | 83.597 | 61.705 | 1.00 | 0.60 |
| ATOM | 4399 | N | TRP | A | 317 | 48.474 | 83.353 | 61.547 | 1.00 | 0.46 |
| ATOM | 4400 | H | TRP | A | 317 | 47.561 | 83.584 | 61.908 | 1.00 | 0.46 |
| ATOM | 4401 | CA | TRP | A | 317 | 48.621 | 82.331 | 60.517 | 1.00 | 0.46 |
| ATOM | 4402 | HA | TRP | A | 317 | 49.608 | 81.884 | 60.585 | 1.00 | 0.46 |
| ATOM | 4403 | CB | TRP | A | 317 | 47.609 | 81.197 | 60.734 | 1.00 | 0.46 |
| ATOM | 4404 | HB1 | TRP | A | 317 | 47.672 | 80.517 | 59.884 | 1.00 | 0.46 |
| ATOM | 4405 | HB2 | TRP | A | 317 | 46.605 | 81.621 | 60.734 | 1.00 | 0.46 |
| ATOM | 4406 | CG | TRP | A | 317 | 47.781 | 80.365 | 61.982 | 1.00 | 0.46 |
| ATOM | 4407 | CD1 | TRP | A | 317 | 48.441 | 80.718 | 63.112 | 1.00 | 0.46 |
| ATOM | 4408 | HD1 | TRP | A | 317 | 48.961 | 81.651 | 63.280 | 1.00 | 0.46 |
| ATOM | 4409 | NE1 | TRP | A | 317 | 48.282 | 79.754 | 64.080 | 1.00 | 0.46 |
| ATOM | 4410 | HE1 | TRP | A | 317 | 48.615 | 79.892 | 65.029 | 1.00 | 0.46 |
| ATOM | 4411 | CE2 | TRP | A | 317 | 47.547 | 78.691 | 63.616 | 1.00 | 0.46 |
| ATOM | 4412 | CZ2 | TRP | A | 317 | 47.132 | 77.488 | 64.197 | 1.00 | 0.46 |
| ATOM | 4413 | HZ2 | TRP | A | 317 | 47.400 | 77.258 | 65.215 | 1.00 | 0.46 |
| ATOM | 4414 | CH2 | TRP | A | 317 | 46.352 | 76.598 | 63.444 | 1.00 | 0.46 |
| ATOM | 4415 | HH2 | TRP | A | 317 | 46.027 | 75.665 | 63.876 | 1.00 | 0.46 |
| ATOM | 4416 | CZ3 | TRP | A | 317 | 45.996 | 76.926 | 62.128 | 1.00 | 0.46 |
| ATOM | 4417 | HZ3 | TRP | A | 317 | 45.379 | 76.251 | 61.552 | 1.00 | 0.46 |
| ATOM | 4418 | CE3 | TRP | A | 317 | 46.437 | 78.128 | 61.550 | 1.00 | 0.46 |
| ATOM | 4419 | HE3 | TRP | A | 317 | 46.166 | 78.367 | 60.539 | 1.00 | 0.46 |
| ATOM | 4420 | CD2 | TRP | A | 317 | 47.227 | 79.044 | 62.271 | 1.00 | 0.46 |
| ATOM | 4421 | C | TRP | A | 317 | 48.514 | 82.986 | 59.143 | 1.00 | 0.46 |
| ATOM | 4422 | O | TRP | A | 317 | 48.016 | 84.102 | 58.991 | 1.00 | 0.46 |
| ATOM | 4423 | N | THR | A | 318 | 48.996 | 82.296 | 58.123 | 1.00 | 0.46 |
| ATOM | 4424 | H | THR | A | 318 | 49.494 | 81.437 | 58.336 | 1.00 | 0.46 |
| ATOM | 4425 | CA | THR | A | 318 | 48.872 | 82.695 | 56.721 | 1.00 | 0.46 |
| ATOM | 4426 | HA | THR | A | 318 | 47.951 | 83.262 | 56.585 | 1.00 | 0.46 |
| ATOM | 4427 | CB | THR | A | 318 | 50.040 | 83.600 | 56.285 | 1.00 | 0.46 |
| ATOM | 4428 | HB | THR | A | 318 | 50.991 | 83.121 | 56.523 | 1.00 | 0.46 |
| ATOM | 4429 | CG2 | THR | A | 318 | 50.007 | 83.975 | 54.801 | 1.00 | 0.46 |
| ATOM | 4430 | 1HG2 | THR | A | 318 | 50.800 | 84.695 | 54.596 | 1.00 | 0.46 |
| ATOM | 4431 | 2HG2 | THR | A | 318 | 50.177 | 83.094 | 54.183 | 1.00 | 0.46 |
| ATOM | 4432 | 3HG2 | THR | A | 318 | 49.044 | 84.421 | 54.549 | 1.00 | 0.46 |
| ATOM | 4433 | OG1 | THR | A | 318 | 49.941 | 84.823 | 56.981 | 1.00 | 0.46 |
| ATOM | 4434 | HG1 | THR | A | 318 | 49.557 | 84.637 | 57.849 | 1.00 | 0.46 |
| ATOM | 4435 | C | THR | A | 318 | 48.777 | 81.437 | 55.882 | 1.00 | 0.46 |
| ATOM | 4436 | O | THR | A | 318 | 49.782 | 80.804 | 55.557 | 1.00 | 0.46 |
| ATOM | 4437 | N | TRP | A | 319 | 47.545 | 81.010 | 55.615 | 1.00 | 0.55 |
| ATOM | 4438 | H | TRP | A | 319 | 46.751 | 81.507 | 56.001 | 1.00 | 0.55 |
| ATOM | 4439 | CA | TRP | A | 319 | 47.281 | 79.826 | 54.807 | 1.00 | 0.55 |
| ATOM | 4440 | HA | TRP | A | 319 | 48.170 | 79.198 | 54.759 | 1.00 | 0.55 |

| | | | | | | | | | | |
|---|---|---|---|---|---|---|---|---|---|---|
| ATOM | 4441 | CB | TRP | A | 319 | 46.180 | 79.003 | 55.471 | 1.00 | 0.55 |
| ATOM | 4442 | HB1 | TRP | A | 319 | 45.854 | 78.251 | 54.756 | 1.00 | 0.55 |
| ATOM | 4443 | HB2 | TRP | A | 319 | 45.320 | 79.641 | 55.677 | 1.00 | 0.55 |
| ATOM | 4444 | CG | TRP | A | 319 | 46.568 | 78.277 | 56.727 | 1.00 | 0.55 |
| ATOM | 4445 | CD1 | TRP | A | 319 | 47.606 | 78.552 | 57.556 | 1.00 | 0.55 |
| ATOM | 4446 | HD1 | TRP | A | 319 | 48.321 | 79.353 | 57.430 | 1.00 | 0.55 |
| ATOM | 4447 | NE1 | TRP | A | 319 | 47.672 | 77.610 | 58.557 | 1.00 | 0.55 |
| ATOM | 4448 | HE1 | TRP | A | 319 | 48.381 | 77.618 | 59.281 | 1.00 | 0.55 |
| ATOM | 4449 | CE2 | TRP | A | 319 | 46.684 | 76.662 | 58.414 | 1.00 | 0.55 |
| ATOM | 4450 | CZ2 | TRP | A | 319 | 46.358 | 75.508 | 59.134 | 1.00 | 0.55 |
| ATOM | 4451 | HZ2 | TRP | A | 319 | 46.963 | 75.213 | 59.974 | 1.00 | 0.55 |
| ATOM | 4452 | CH2 | TRP | A | 319 | 45.230 | 74.762 | 58.760 | 1.00 | 0.55 |
| ATOM | 4453 | HH2 | TRP | A | 319 | 44.957 | 73.876 | 59.314 | 1.00 | 0.55 |
| ATOM | 4454 | CZ3 | TRP | A | 319 | 44.457 | 75.177 | 57.663 | 1.00 | 0.55 |
| ATOM | 4455 | HZ3 | TRP | A | 319 | 43.587 | 74.611 | 57.370 | 1.00 | 0.55 |
| ATOM | 4456 | CE3 | TRP | A | 319 | 44.813 | 76.317 | 56.921 | 1.00 | 0.55 |
| ATOM | 4457 | HE3 | TRP | A | 319 | 44.230 | 76.599 | 56.056 | 1.00 | 0.55 |
| ATOM | 4458 | CD2 | TRP | A | 319 | 45.936 | 77.088 | 57.280 | 1.00 | 0.55 |
| ATOM | 4459 | C | TRP | A | 319 | 46.919 | 80.245 | 53.383 | 1.00 | 0.55 |
| ATOM | 4460 | O | TRP | A | 319 | 46.123 | 81.161 | 53.168 | 1.00 | 0.55 |
| ATOM | 4461 | N | SER | A | 320 | 47.521 | 79.553 | 52.422 | 1.00 | 0.43 |
| ATOM | 4462 | H | SER | A | 320 | 48.217 | 78.869 | 52.710 | 1.00 | 0.43 |
| ATOM | 4463 | CA | SER | A | 320 | 47.434 | 79.808 | 50.993 | 1.00 | 0.43 |
| ATOM | 4464 | HA | SER | A | 320 | 46.512 | 80.350 | 50.788 | 1.00 | 0.43 |
| ATOM | 4465 | CB | SER | A | 320 | 48.601 | 80.680 | 50.524 | 1.00 | 0.43 |
| ATOM | 4466 | HB1 | SER | A | 320 | 49.542 | 80.136 | 50.622 | 1.00 | 0.43 |
| ATOM | 4467 | HB2 | SER | A | 320 | 48.644 | 81.582 | 51.136 | 1.00 | 0.43 |
| ATOM | 4468 | OG | SER | A | 320 | 48.395 | 81.050 | 49.175 | 1.00 | 0.43 |
| ATOM | 4469 | HG | SER | A | 320 | 48.663 | 80.284 | 48.605 | 1.00 | 0.43 |
| ATOM | 4470 | C | SER | A | 320 | 47.432 | 78.506 | 50.201 | 1.00 | 0.43 |
| ATOM | 4471 | O | SER | A | 320 | 48.295 | 77.648 | 50.383 | 1.00 | 0.43 |
| ATOM | 4472 | N | ASP | A | 321 | 46.455 | 78.391 | 49.299 | 1.00 | 0.60 |
| ATOM | 4473 | H | ASP | A | 321 | 45.827 | 79.175 | 49.233 | 1.00 | 0.60 |
| ATOM | 4474 | CA | ASP | A | 321 | 46.279 | 77.349 | 48.275 | 1.00 | 0.60 |
| ATOM | 4475 | HA | ASP | A | 321 | 45.254 | 77.531 | 47.954 | 1.00 | 0.60 |
| ATOM | 4476 | CB | ASP | A | 321 | 47.051 | 77.662 | 46.980 | 1.00 | 0.60 |
| ATOM | 4477 | HB1 | ASP | A | 321 | 46.621 | 78.569 | 46.549 | 1.00 | 0.60 |
| ATOM | 4478 | HB2 | ASP | A | 321 | 46.866 | 76.858 | 46.265 | 1.00 | 0.60 |
| ATOM | 4479 | CG | ASP | A | 321 | 48.557 | 77.892 | 47.112 | 1.00 | 0.60 |
| ATOM | 4480 | OD1 | ASP | A | 321 | 49.285 | 77.095 | 46.484 | 1.00 | 0.60 |
| ATOM | 4481 | OD2 | ASP | A | 321 | 48.964 | 78.893 | 47.752 | 1.00 | 0.60 |
| ATOM | 4482 | C | ASP | A | 321 | 46.128 | 75.868 | 48.694 | 1.00 | 0.60 |
| ATOM | 4483 | O | ASP | A | 321 | 45.482 | 75.099 | 47.982 | 1.00 | 0.60 |
| ATOM | 4484 | N | VAL | A | 322 | 46.494 | 75.557 | 49.933 | 1.00 | 0.58 |

| | | | | | | | | | | |
|---|---|---|---|---|---|---|---|---|---|---|
| ATOM | 4485 | H | VAL | A | 322 | 47.203 | 76.163 | 50.332 | 1.00 | 0.58 |
| ATOM | 4486 | CA | VAL | A | 322 | 45.925 | 74.591 | 50.893 | 1.00 | 0.58 |
| ATOM | 4487 | HA | VAL | A | 322 | 46.745 | 74.405 | 51.579 | 1.00 | 0.58 |
| ATOM | 4488 | CB | VAL | A | 322 | 44.833 | 75.230 | 51.780 | 1.00 | 0.58 |
| ATOM | 4489 | HB | VAL | A | 322 | 44.446 | 74.455 | 52.443 | 1.00 | 0.58 |
| ATOM | 4490 | CG1 | VAL | A | 322 | 45.431 | 76.329 | 52.674 | 1.00 | 0.58 |
| ATOM | 4491 | 1HG1 | VAL | A | 322 | 45.773 | 77.165 | 52.067 | 1.00 | 0.58 |
| ATOM | 4492 | 2HG1 | VAL | A | 322 | 44.674 | 76.676 | 53.375 | 1.00 | 0.58 |
| ATOM | 4493 | 3HG1 | VAL | A | 322 | 46.273 | 75.926 | 53.236 | 1.00 | 0.58 |
| ATOM | 4494 | CG2 | VAL | A | 322 | 43.647 | 75.838 | 51.017 | 1.00 | 0.58 |
| ATOM | 4495 | 1HG2 | VAL | A | 322 | 43.161 | 75.076 | 50.409 | 1.00 | 0.58 |
| ATOM | 4496 | 2HG2 | VAL | A | 322 | 42.911 | 76.210 | 51.729 | 1.00 | 0.58 |
| ATOM | 4497 | 3HG2 | VAL | A | 322 | 43.978 | 76.657 | 50.380 | 1.00 | 0.58 |
| ATOM | 4498 | C | VAL | A | 322 | 45.534 | 73.162 | 50.455 | 1.00 | 0.58 |
| ATOM | 4499 | O | VAL | A | 322 | 46.108 | 72.211 | 50.963 | 1.00 | 0.58 |
| ATOM | 4500 | N | GLU | A | 323 | 44.555 | 72.978 | 49.576 | 1.00 | 0.56 |
| ATOM | 4501 | H | GLU | A | 323 | 44.334 | 73.783 | 49.004 | 1.00 | 0.56 |
| ATOM | 4502 | CA | GLU | A | 323 | 43.531 | 71.913 | 49.628 | 1.00 | 0.56 |
| ATOM | 4503 | HA | GLU | A | 323 | 42.856 | 72.215 | 50.430 | 1.00 | 0.56 |
| ATOM | 4504 | CB | GLU | A | 323 | 42.679 | 71.987 | 48.345 | 1.00 | 0.56 |
| ATOM | 4505 | HB1 | GLU | A | 323 | 43.356 | 72.033 | 47.490 | 1.00 | 0.56 |
| ATOM | 4506 | HB2 | GLU | A | 323 | 42.121 | 72.925 | 48.364 | 1.00 | 0.56 |
| ATOM | 4507 | CG | GLU | A | 323 | 41.685 | 70.839 | 48.068 | 1.00 | 0.56 |
| ATOM | 4508 | HG1 | GLU | A | 323 | 42.242 | 69.931 | 47.836 | 1.00 | 0.56 |
| ATOM | 4509 | HG2 | GLU | A | 323 | 41.141 | 71.102 | 47.157 | 1.00 | 0.56 |
| ATOM | 4510 | CD | GLU | A | 323 | 40.659 | 70.516 | 49.169 | 1.00 | 0.56 |
| ATOM | 4511 | OE1 | GLU | A | 323 | 40.860 | 70.861 | 50.357 | 1.00 | 0.56 |
| ATOM | 4512 | OE2 | GLU | A | 323 | 39.663 | 69.841 | 48.821 | 1.00 | 0.56 |
| ATOM | 4513 | C | GLU | A | 323 | 43.922 | 70.436 | 49.992 | 1.00 | 0.56 |
| ATOM | 4514 | O | GLU | A | 323 | 44.454 | 69.672 | 49.172 | 1.00 | 0.56 |
| ATOM | 4515 | N | VAL | A | 324 | 43.513 | 69.974 | 51.192 | 1.00 | 0.45 |
| ATOM | 4516 | H | VAL | A | 324 | 43.136 | 70.635 | 51.851 | 1.00 | 0.45 |
| ATOM | 4517 | CA | VAL | A | 324 | 43.431 | 68.562 | 51.536 | 1.00 | 0.45 |
| ATOM | 4518 | HA | VAL | A | 324 | 44.425 | 68.191 | 51.312 | 1.00 | 0.45 |
| ATOM | 4519 | CB | VAL | A | 324 | 43.300 | 68.326 | 53.053 | 1.00 | 0.45 |
| ATOM | 4520 | HB | VAL | A | 324 | 42.328 | 68.673 | 53.405 | 1.00 | 0.45 |
| ATOM | 4521 | CG1 | VAL | A | 324 | 43.463 | 66.830 | 53.384 | 1.00 | 0.45 |
| ATOM | 4522 | 1HG1 | VAL | A | 324 | 43.427 | 66.671 | 54.456 | 1.00 | 0.45 |
| ATOM | 4523 | 2HG1 | VAL | A | 324 | 42.649 | 66.250 | 52.951 | 1.00 | 0.45 |
| ATOM | 4524 | 3HG1 | VAL | A | 324 | 44.413 | 66.459 | 52.999 | 1.00 | 0.45 |
| ATOM | 4525 | CG2 | VAL | A | 324 | 44.417 | 69.088 | 53.787 | 1.00 | 0.45 |
| ATOM | 4526 | 1HG2 | VAL | A | 324 | 44.496 | 68.759 | 54.815 | 1.00 | 0.45 |
| ATOM | 4527 | 2HG2 | VAL | A | 324 | 45.376 | 68.910 | 53.298 | 1.00 | 0.45 |
| ATOM | 4528 | 3HG2 | VAL | A | 324 | 44.213 | 70.158 | 53.776 | 1.00 | 0.45 |

| | | | | | | | | | | |
|---|---|---|---|---|---|---|---|---|---|---|
| ATOM | 4529 | C | VAL | A | 324 | 42.569 | 67.575 | 50.737 | 1.00 | 0.45 |
| ATOM | 4530 | O | VAL | A | 324 | 43.115 | 66.645 | 50.144 | 1.00 | 0.45 |
| ATOM | 4531 | N | THR | A | 325 | 41.246 | 67.676 | 50.794 | 1.00 | 0.66 |
| ATOM | 4532 | H | THR | A | 325 | 40.859 | 68.530 | 51.177 | 1.00 | 0.66 |
| ATOM | 4533 | CA | THR | A | 325 | 40.299 | 66.726 | 50.175 | 1.00 | 0.66 |
| ATOM | 4534 | HA | THR | A | 325 | 39.330 | 67.139 | 50.457 | 1.00 | 0.66 |
| ATOM | 4535 | CB | THR | A | 325 | 40.377 | 66.878 | 48.633 | 1.00 | 0.66 |
| ATOM | 4536 | HB | THR | A | 325 | 41.118 | 67.642 | 48.395 | 1.00 | 0.66 |
| ATOM | 4537 | CG2 | THR | A | 325 | 40.754 | 65.646 | 47.804 | 1.00 | 0.66 |
| ATOM | 4538 | 1HG2 | THR | A | 325 | 40.914 | 65.955 | 46.771 | 1.00 | 0.66 |
| ATOM | 4539 | 2HG2 | THR | A | 325 | 41.678 | 65.217 | 48.189 | 1.00 | 0.66 |
| ATOM | 4540 | 3HG2 | THR | A | 325 | 39.957 | 64.905 | 47.826 | 1.00 | 0.66 |
| ATOM | 4541 | OG1 | THR | A | 325 | 39.140 | 67.364 | 48.176 | 1.00 | 0.66 |
| ATOM | 4542 | HG1 | THR | A | 325 | 39.187 | 68.332 | 48.386 | 1.00 | 0.66 |
| ATOM | 4543 | C | THR | A | 325 | 40.198 | 65.268 | 50.642 | 1.00 | 0.66 |
| ATOM | 4544 | O | THR | A | 325 | 41.087 | 64.699 | 51.274 | 1.00 | 0.66 |
| ATOM | 4545 | N | GLY | A | 326 | 39.068 | 64.635 | 50.309 | 1.00 | 1.01 |
| ATOM | 4546 | H | GLY | A | 326 | 38.352 | 65.197 | 49.869 | 1.00 | 1.01 |
| ATOM | 4547 | CA | GLY | A | 326 | 38.877 | 63.176 | 50.313 | 1.00 | 1.01 |
| ATOM | 4548 | HA1 | GLY | A | 326 | 39.822 | 62.702 | 50.047 | 1.00 | 1.01 |
| ATOM | 4549 | HA2 | GLY | A | 326 | 38.149 | 62.922 | 49.543 | 1.00 | 1.01 |
| ATOM | 4550 | C | GLY | A | 326 | 38.408 | 62.532 | 51.605 | 1.00 | 1.01 |
| ATOM | 4551 | O | GLY | A | 326 | 38.057 | 61.361 | 51.564 | 1.00 | 1.01 |
| ATOM | 4552 | N | GLY | A | 327 | 38.372 | 63.283 | 52.701 | 1.00 | 0.95 |
| ATOM | 4553 | H | GLY | A | 327 | 38.508 | 64.280 | 52.603 | 1.00 | 0.95 |
| ATOM | 4554 | CA | GLY | A | 327 | 38.016 | 62.801 | 54.033 | 1.00 | 0.95 |
| ATOM | 4555 | HA1 | GLY | A | 327 | 38.878 | 62.899 | 54.683 | 1.00 | 0.95 |
| ATOM | 4556 | HA2 | GLY | A | 327 | 37.718 | 61.751 | 54.008 | 1.00 | 0.95 |
| ATOM | 4557 | C | GLY | A | 327 | 36.865 | 63.620 | 54.618 | 1.00 | 0.95 |
| ATOM | 4558 | O | GLY | A | 327 | 36.268 | 64.466 | 53.950 | 1.00 | 0.95 |
| ATOM | 4559 | N | GLU | A | 328 | 36.566 | 63.361 | 55.884 | 1.00 | 1.14 |
| ATOM | 4560 | H | GLU | A | 328 | 37.085 | 62.625 | 56.356 | 1.00 | 1.14 |
| ATOM | 4561 | CA | GLU | A | 328 | 35.640 | 64.166 | 56.688 | 1.00 | 1.14 |
| ATOM | 4562 | HA | GLU | A | 328 | 34.667 | 64.195 | 56.196 | 1.00 | 1.14 |
| ATOM | 4563 | CB | GLU | A | 328 | 35.494 | 63.479 | 58.059 | 1.00 | 1.14 |
| ATOM | 4564 | HB1 | GLU | A | 328 | 35.259 | 64.218 | 58.827 | 1.00 | 1.14 |
| ATOM | 4565 | HB2 | GLU | A | 328 | 36.445 | 63.018 | 58.331 | 1.00 | 1.14 |
| ATOM | 4566 | CG | GLU | A | 328 | 34.398 | 62.405 | 58.081 | 1.00 | 1.14 |
| ATOM | 4567 | HG1 | GLU | A | 328 | 34.643 | 61.691 | 58.871 | 1.00 | 1.14 |
| ATOM | 4568 | HG2 | GLU | A | 328 | 34.380 | 61.867 | 57.131 | 1.00 | 1.14 |
| ATOM | 4569 | CD | GLU | A | 328 | 33.023 | 63.019 | 58.367 | 1.00 | 1.14 |
| ATOM | 4570 | OE1 | GLU | A | 328 | 32.723 | 64.092 | 57.792 | 1.00 | 1.14 |
| ATOM | 4571 | OE2 | GLU | A | 328 | 32.287 | 62.443 | 59.200 | 1.00 | 1.14 |
| ATOM | 4572 | C | GLU | A | 328 | 36.091 | 65.632 | 56.874 | 1.00 | 1.14 |

| | | | | | | | | | | |
|---|---|---|---|---|---|---|---|---|---|---|
| ATOM | 4573 | O | GLU | A | 328 | 37.282 | 65.935 | 56.765 | 1.00 | 1.14 |
| ATOM | 4574 | N | ASP | A | 329 | 35.163 | 66.521 | 57.278 | 1.00 | 1.01 |
| ATOM | 4575 | H | ASP | A | 329 | 34.216 | 66.179 | 57.416 | 1.00 | 1.01 |
| ATOM | 4576 | CA | ASP | A | 329 | 35.416 | 67.958 | 57.551 | 1.00 | 1.01 |
| ATOM | 4577 | HA | ASP | A | 329 | 36.494 | 68.111 | 57.561 | 1.00 | 1.01 |
| ATOM | 4578 | CB | ASP | A | 329 | 34.900 | 68.823 | 56.368 | 1.00 | 1.01 |
| ATOM | 4579 | HB1 | ASP | A | 329 | 33.834 | 69.015 | 56.505 | 1.00 | 1.01 |
| ATOM | 4580 | HB2 | ASP | A | 329 | 35.002 | 68.244 | 55.447 | 1.00 | 1.01 |
| ATOM | 4581 | CG | ASP | A | 329 | 35.643 | 70.163 | 56.146 | 1.00 | 1.01 |
| ATOM | 4582 | OD1 | ASP | A | 329 | 36.612 | 70.213 | 55.346 | 1.00 | 1.01 |
| ATOM | 4583 | OD2 | ASP | A | 329 | 35.231 | 71.186 | 56.738 | 1.00 | 1.01 |
| ATOM | 4584 | C | ASP | A | 329 | 34.888 | 68.394 | 58.952 | 1.00 | 1.01 |
| ATOM | 4585 | O | ASP | A | 329 | 33.696 | 68.270 | 59.238 | 1.00 | 1.01 |
| ATOM | 4586 | N | TYR | A | 330 | 35.787 | 68.727 | 59.908 | 1.00 | 1.16 |
| ATOM | 4587 | H | TYR | A | 330 | 36.745 | 68.874 | 59.625 | 1.00 | 1.16 |
| ATOM | 4588 | CA | TYR | A | 330 | 35.519 | 68.387 | 61.323 | 1.00 | 1.16 |
| ATOM | 4589 | HA | TYR | A | 330 | 34.930 | 67.479 | 61.239 | 1.00 | 1.16 |
| ATOM | 4590 | CB | TYR | A | 330 | 36.695 | 68.063 | 62.280 | 1.00 | 1.16 |
| ATOM | 4591 | HB1 | TYR | A | 330 | 36.891 | 68.941 | 62.897 | 1.00 | 1.16 |
| ATOM | 4592 | HB2 | TYR | A | 330 | 37.619 | 67.899 | 61.771 | 1.00 | 1.16 |
| ATOM | 4593 | CG | TYR | A | 330 | 36.431 | 66.867 | 63.204 | 1.00 | 1.16 |
| ATOM | 4594 | CD1 | TYR | A | 330 | 35.816 | 65.687 | 62.724 | 1.00 | 1.16 |
| ATOM | 4595 | HD1 | TYR | A | 330 | 35.569 | 65.580 | 61.677 | 1.00 | 1.16 |
| ATOM | 4596 | CE1 | TYR | A | 330 | 35.508 | 64.626 | 63.596 | 1.00 | 1.16 |
| ATOM | 4597 | HE1 | TYR | A | 330 | 35.020 | 63.736 | 63.225 | 1.00 | 1.16 |
| ATOM | 4598 | CZ | TYR | A | 330 | 35.821 | 64.724 | 64.965 | 1.00 | 1.16 |
| ATOM | 4599 | OH | TYR | A | 330 | 35.481 | 63.722 | 65.819 | 1.00 | 1.16 |
| ATOM | 4600 | HH | TYR | A | 330 | 35.717 | 63.933 | 66.722 | 1.00 | 1.16 |
| ATOM | 4601 | CE2 | TYR | A | 330 | 36.481 | 65.876 | 65.445 | 1.00 | 1.16 |
| ATOM | 4602 | HE2 | TYR | A | 330 | 36.746 | 65.957 | 66.488 | 1.00 | 1.16 |
| ATOM | 4603 | CD2 | TYR | A | 330 | 36.789 | 66.933 | 64.565 | 1.00 | 1.16 |
| ATOM | 4604 | HD2 | TYR | A | 330 | 37.302 | 67.803 | 64.944 | 1.00 | 1.16 |
| ATOM | 4605 | C | TYR | A | 330 | 34.617 | 69.364 | 62.061 | 1.00 | 1.16 |
| ATOM | 4606 | O | TYR | A | 330 | 34.886 | 70.555 | 62.192 | 1.00 | 1.16 |
| ATOM | 4607 | N | GLY | A | 331 | 33.648 | 68.770 | 62.748 | 1.00 | 1.39 |
| ATOM | 4608 | H | GLY | A | 331 | 33.498 | 67.787 | 62.582 | 1.00 | 1.39 |
| ATOM | 4609 | CA | GLY | A | 331 | 32.774 | 69.459 | 63.683 | 1.00 | 1.39 |
| ATOM | 4610 | HA1 | GLY | A | 331 | 31.832 | 68.916 | 63.740 | 1.00 | 1.39 |
| ATOM | 4611 | HA2 | GLY | A | 331 | 32.559 | 70.452 | 63.286 | 1.00 | 1.39 |
| ATOM | 4612 | C | GLY | A | 331 | 33.297 | 69.644 | 65.109 | 1.00 | 1.39 |
| ATOM | 4613 | O | GLY | A | 331 | 32.516 | 69.716 | 66.058 | 1.00 | 1.39 |
| ATOM | 4614 | N | SER | A | 332 | 34.617 | 69.717 | 65.246 | 1.00 | 0.88 |
| ATOM | 4615 | H | SER | A | 332 | 35.152 | 69.663 | 64.389 | 1.00 | 0.88 |
| ATOM | 4616 | CA | SER | A | 332 | 35.328 | 70.266 | 66.400 | 1.00 | 0.88 |

| | | | | | | | | | | |
|---|---|---|---|---|---|---|---|---|---|---|
| ATOM | 4617 | HA | SER | A | 332 | 34.740 | 71.072 | 66.843 | 1.00 | 0.88 |
| ATOM | 4618 | CB | SER | A | 332 | 35.571 | 69.213 | 67.484 | 1.00 | 0.88 |
| ATOM | 4619 | HB1 | SER | A | 332 | 35.875 | 68.278 | 67.014 | 1.00 | 0.88 |
| ATOM | 4620 | HB2 | SER | A | 332 | 34.640 | 69.041 | 68.025 | 1.00 | 0.88 |
| ATOM | 4621 | OG | SER | A | 332 | 36.583 | 69.591 | 68.399 | 1.00 | 0.88 |
| ATOM | 4622 | HG | SER | A | 332 | 36.503 | 70.544 | 68.660 | 1.00 | 0.88 |
| ATOM | 4623 | C | SER | A | 332 | 36.621 | 70.857 | 65.866 | 1.00 | 0.88 |
| ATOM | 4624 | O | SER | A | 332 | 37.457 | 70.140 | 65.311 | 1.00 | 0.88 |
| ATOM | 4625 | N | CYS | A | 333 | 36.740 | 72.179 | 65.929 | 1.00 | 0.80 |
| ATOM | 4626 | H | CYS | A | 333 | 36.057 | 72.704 | 66.476 | 1.00 | 0.80 |
| ATOM | 4627 | CA | CYS | A | 333 | 37.885 | 72.899 | 65.410 | 1.00 | 0.80 |
| ATOM | 4628 | HA | CYS | A | 333 | 38.752 | 72.305 | 65.689 | 1.00 | 0.80 |
| ATOM | 4629 | CB | CYS | A | 333 | 37.857 | 72.920 | 63.865 | 1.00 | 0.80 |
| ATOM | 4630 | HB1 | CYS | A | 333 | 37.696 | 71.903 | 63.507 | 1.00 | 0.80 |
| ATOM | 4631 | HB2 | CYS | A | 333 | 38.852 | 73.223 | 63.546 | 1.00 | 0.80 |
| ATOM | 4632 | SG | CYS | A | 333 | 36.668 | 73.997 | 63.004 | 1.00 | 0.80 |
| ATOM | 4633 | C | CYS | A | 333 | 38.090 | 74.286 | 66.061 | 1.00 | 0.80 |
| ATOM | 4634 | O | CYS | A | 333 | 37.709 | 75.316 | 65.501 | 1.00 | 0.80 |
| ATOM | 4635 | N | GLU | A | 334 | 38.634 | 74.346 | 67.283 | 1.00 | 0.91 |
| ATOM | 4636 | H | GLU | A | 334 | 39.043 | 73.521 | 67.699 | 1.00 | 0.91 |
| ATOM | 4637 | CA | GLU | A | 334 | 38.319 | 75.501 | 68.123 | 1.00 | 0.91 |
| ATOM | 4638 | HA | GLU | A | 334 | 37.368 | 75.936 | 67.810 | 1.00 | 0.91 |
| ATOM | 4639 | CB | GLU | A | 334 | 38.141 | 75.004 | 69.572 | 1.00 | 0.91 |
| ATOM | 4640 | HB1 | GLU | A | 334 | 38.313 | 75.838 | 70.255 | 1.00 | 0.91 |
| ATOM | 4641 | HB2 | GLU | A | 334 | 38.888 | 74.239 | 69.796 | 1.00 | 0.91 |
| ATOM | 4642 | CG | GLU | A | 334 | 36.732 | 74.460 | 69.868 | 1.00 | 0.91 |
| ATOM | 4643 | HG1 | GLU | A | 334 | 36.028 | 75.294 | 69.815 | 1.00 | 0.91 |
| ATOM | 4644 | HG2 | GLU | A | 334 | 36.711 | 74.087 | 70.895 | 1.00 | 0.91 |
| ATOM | 4645 | CD | GLU | A | 334 | 36.272 | 73.347 | 68.912 | 1.00 | 0.91 |
| ATOM | 4646 | OE1 | GLU | A | 334 | 36.649 | 72.170 | 69.105 | 1.00 | 0.91 |
| ATOM | 4647 | OE2 | GLU | A | 334 | 35.540 | 73.662 | 67.947 | 1.00 | 0.91 |
| ATOM | 4648 | C | GLU | A | 334 | 39.387 | 76.599 | 68.048 | 1.00 | 0.91 |
| ATOM | 4649 | O | GLU | A | 334 | 40.566 | 76.360 | 67.786 | 1.00 | 0.91 |
| ATOM | 4650 | N | ASN | A | 335 | 38.935 | 77.834 | 68.286 | 1.00 | 1.19 |
| ATOM | 4651 | H | ASN | A | 335 | 37.964 | 77.908 | 68.544 | 1.00 | 1.19 |
| ATOM | 4652 | CA | ASN | A | 335 | 39.701 | 79.084 | 68.233 | 1.00 | 1.19 |
| ATOM | 4653 | HA | ASN | A | 335 | 38.963 | 79.874 | 68.078 | 1.00 | 1.19 |
| ATOM | 4654 | CB | ASN | A | 335 | 40.340 | 79.323 | 69.615 | 1.00 | 1.19 |
| ATOM | 4655 | HB1 | ASN | A | 335 | 41.298 | 78.808 | 69.671 | 1.00 | 1.19 |
| ATOM | 4656 | HB2 | ASN | A | 335 | 39.701 | 78.938 | 70.410 | 1.00 | 1.19 |
| ATOM | 4657 | CG | ASN | A | 335 | 40.523 | 80.805 | 69.861 | 1.00 | 1.19 |
| ATOM | 4658 | OD1 | ASN | A | 335 | 39.578 | 81.576 | 69.852 | 1.00 | 1.19 |
| ATOM | 4659 | ND2 | ASN | A | 335 | 41.729 | 81.268 | 70.061 | 1.00 | 1.19 |
| ATOM | 4660 | 1HD2 | ASN | A | 335 | 42.494 | 80.623 | 70.234 | 1.00 | 1.19 |

| | | | | | | | | | | |
|---|---|---|---|---|---|---|---|---|---|---|
| ATOM | 4661 | 2HD2 | ASN | A | 335 | 41.844 | 82.262 | 70.053 | 1.00 | 1.19 |
| ATOM | 4662 | C | ASN | A | 335 | 40.668 | 79.219 | 67.031 | 1.00 | 1.19 |
| ATOM | 4663 | O | ASN | A | 335 | 41.763 | 79.776 | 67.133 | 1.00 | 1.19 |
| ATOM | 4664 | N | VAL | A | 336 | 40.241 | 78.708 | 65.874 | 1.00 | 1.37 |
| ATOM | 4665 | H | VAL | A | 336 | 39.400 | 78.150 | 65.896 | 1.00 | 1.37 |
| ATOM | 4666 | CA | VAL | A | 336 | 40.871 | 78.930 | 64.568 | 1.00 | 1.37 |
| ATOM | 4667 | HA | VAL | A | 336 | 41.796 | 78.359 | 64.555 | 1.00 | 1.37 |
| ATOM | 4668 | CB | VAL | A | 336 | 39.945 | 78.363 | 63.468 | 1.00 | 1.37 |
| ATOM | 4669 | HB | VAL | A | 336 | 38.944 | 78.774 | 63.604 | 1.00 | 1.37 |
| ATOM | 4670 | CG1 | VAL | A | 336 | 40.399 | 78.669 | 62.035 | 1.00 | 1.37 |
| ATOM | 4671 | 1HG1 | VAL | A | 336 | 39.723 | 78.191 | 61.327 | 1.00 | 1.37 |
| ATOM | 4672 | 2HG1 | VAL | A | 336 | 40.364 | 79.742 | 61.847 | 1.00 | 1.37 |
| ATOM | 4673 | 3HG1 | VAL | A | 336 | 41.410 | 78.301 | 61.871 | 1.00 | 1.37 |
| ATOM | 4674 | CG2 | VAL | A | 336 | 39.866 | 76.837 | 63.564 | 1.00 | 1.37 |
| ATOM | 4675 | 1HG2 | VAL | A | 336 | 40.812 | 76.412 | 63.252 | 1.00 | 1.37 |
| ATOM | 4676 | 2HG2 | VAL | A | 336 | 39.667 | 76.520 | 64.586 | 1.00 | 1.37 |
| ATOM | 4677 | 3HG2 | VAL | A | 336 | 39.066 | 76.462 | 62.926 | 1.00 | 1.37 |
| ATOM | 4678 | C | VAL | A | 336 | 41.168 | 80.416 | 64.316 | 1.00 | 1.37 |
| ATOM | 4679 | O | VAL | A | 336 | 40.276 | 81.249 | 64.488 | 1.00 | 1.37 |
| ATOM | 4680 | N | PRO | A | 337 | 42.383 | 80.773 | 63.864 | 1.00 | 1.36 |
| ATOM | 4681 | CD | PRO | A | 337 | 43.524 | 79.905 | 63.642 | 1.00 | 1.36 |
| ATOM | 4682 | HD1 | PRO | A | 337 | 43.314 | 79.169 | 62.866 | 1.00 | 1.36 |
| ATOM | 4683 | HD2 | PRO | A | 337 | 43.774 | 79.398 | 64.566 | 1.00 | 1.36 |
| ATOM | 4684 | CG | PRO | A | 337 | 44.675 | 80.808 | 63.203 | 1.00 | 1.36 |
| ATOM | 4685 | HG1 | PRO | A | 337 | 44.828 | 80.703 | 62.129 | 1.00 | 1.36 |
| ATOM | 4686 | HG2 | PRO | A | 337 | 45.578 | 80.576 | 63.760 | 1.00 | 1.36 |
| ATOM | 4687 | CB | PRO | A | 337 | 44.225 | 82.227 | 63.521 | 1.00 | 1.36 |
| ATOM | 4688 | HB1 | PRO | A | 337 | 44.625 | 82.950 | 62.808 | 1.00 | 1.36 |
| ATOM | 4689 | HB2 | PRO | A | 337 | 44.536 | 82.484 | 64.535 | 1.00 | 1.36 |
| ATOM | 4690 | CA | PRO | A | 337 | 42.703 | 82.135 | 63.471 | 1.00 | 1.36 |
| ATOM | 4691 | HA | PRO | A | 337 | 42.281 | 82.837 | 64.191 | 1.00 | 1.36 |
| ATOM | 4692 | C | PRO | A | 337 | 42.146 | 82.446 | 62.086 | 1.00 | 1.36 |
| ATOM | 4693 | O | PRO | A | 337 | 42.150 | 81.600 | 61.200 | 1.00 | 1.36 |
| ATOM | 4694 | N | SER | A | 338 | 41.682 | 83.674 | 61.870 | 1.00 | 1.52 |
| ATOM | 4695 | H | SER | A | 338 | 41.753 | 84.359 | 62.607 | 1.00 | 1.52 |
| ATOM | 4696 | CA | SER | A | 338 | 40.872 | 84.043 | 60.705 | 1.00 | 1.52 |
| ATOM | 4697 | HA | SER | A | 338 | 40.136 | 83.253 | 60.545 | 1.00 | 1.52 |
| ATOM | 4698 | CB | SER | A | 338 | 40.112 | 85.336 | 61.035 | 1.00 | 1.52 |
| ATOM | 4699 | HB1 | SER | A | 338 | 39.534 | 85.189 | 61.949 | 1.00 | 1.52 |
| ATOM | 4700 | HB2 | SER | A | 338 | 39.422 | 85.585 | 60.226 | 1.00 | 1.52 |
| ATOM | 4701 | OG | SER | A | 338 | 41.028 | 86.396 | 61.228 | 1.00 | 1.52 |
| ATOM | 4702 | HG | SER | A | 338 | 41.379 | 86.642 | 60.364 | 1.00 | 1.52 |
| ATOM | 4703 | C | SER | A | 338 | 41.631 | 84.212 | 59.375 | 1.00 | 1.52 |
| ATOM | 4704 | O | SER | A | 338 | 41.152 | 84.892 | 58.471 | 1.00 | 1.52 |

| | | | | | | | | | | |
|---|---|---|---|---|---|---|---|---|---|---|
| ATOM | 4705 | N | VAL | A | 339 | 42.815 | 83.619 | 59.251 | 1.00 | 1.86 |
| ATOM | 4706 | H | VAL | A | 339 | 43.127 | 83.075 | 60.044 | 1.00 | 1.86 |
| ATOM | 4707 | CA | VAL | A | 339 | 43.530 | 83.416 | 57.975 | 1.00 | 1.86 |
| ATOM | 4708 | HA | VAL | A | 339 | 42.833 | 83.601 | 57.156 | 1.00 | 1.86 |
| ATOM | 4709 | CB | VAL | A | 339 | 44.718 | 84.397 | 57.811 | 1.00 | 1.86 |
| ATOM | 4710 | HB | VAL | A | 339 | 45.538 | 84.052 | 58.436 | 1.00 | 1.86 |
| ATOM | 4711 | CG1 | VAL | A | 339 | 45.192 | 84.489 | 56.351 | 1.00 | 1.86 |
| ATOM | 4712 | 1HG1 | VAL | A | 339 | 46.048 | 85.160 | 56.285 | 1.00 | 1.86 |
| ATOM | 4713 | 2HG1 | VAL | A | 339 | 45.500 | 83.516 | 55.976 | 1.00 | 1.86 |
| ATOM | 4714 | 3HG1 | VAL | A | 339 | 44.391 | 84.870 | 55.719 | 1.00 | 1.86 |
| ATOM | 4715 | CG2 | VAL | A | 339 | 44.374 | 85.835 | 58.233 | 1.00 | 1.86 |
| ATOM | 4716 | 1HG2 | VAL | A | 339 | 45.228 | 86.487 | 58.049 | 1.00 | 1.86 |
| ATOM | 4717 | 2HG2 | VAL | A | 339 | 43.513 | 86.196 | 57.668 | 1.00 | 1.86 |
| ATOM | 4718 | 3HG2 | VAL | A | 339 | 44.156 | 85.873 | 59.299 | 1.00 | 1.86 |
| ATOM | 4719 | C | VAL | A | 339 | 43.951 | 81.944 | 57.879 | 1.00 | 1.86 |
| ATOM | 4720 | O | VAL | A | 339 | 45.026 | 81.569 | 57.413 | 1.00 | 1.86 |
| ATOM | 4721 | N | ALA | A | 340 | 43.051 | 81.122 | 58.401 | 1.00 | 2.05 |
| ATOM | 4722 | H | ALA | A | 340 | 42.237 | 81.556 | 58.820 | 1.00 | 2.05 |
| ATOM | 4723 | CA | ALA | A | 340 | 42.982 | 79.686 | 58.374 | 1.00 | 2.05 |
| ATOM | 4724 | HA | ALA | A | 340 | 43.518 | 79.318 | 57.497 | 1.00 | 2.05 |
| ATOM | 4725 | CB | ALA | A | 340 | 43.639 | 79.114 | 59.630 | 1.00 | 2.05 |
| ATOM | 4726 | HB1 | ALA | A | 340 | 43.755 | 78.033 | 59.533 | 1.00 | 2.05 |
| ATOM | 4727 | HB2 | ALA | A | 340 | 44.615 | 79.579 | 59.750 | 1.00 | 2.05 |
| ATOM | 4728 | HB3 | ALA | A | 340 | 43.034 | 79.331 | 60.507 | 1.00 | 2.05 |
| ATOM | 4729 | C | ALA | A | 340 | 41.502 | 79.327 | 58.243 | 1.00 | 2.05 |
| ATOM | 4730 | O | ALA | A | 340 | 40.604 | 80.125 | 58.521 | 1.00 | 2.05 |
| ATOM | 4731 | N | SER | A | 341 | 41.242 | 78.094 | 57.849 | 1.00 | 2.24 |
| ATOM | 4732 | H | SER | A | 341 | 42.006 | 77.467 | 57.625 | 1.00 | 2.24 |
| ATOM | 4733 | CA | SER | A | 341 | 39.950 | 77.481 | 58.085 | 1.00 | 2.24 |
| ATOM | 4734 | HA | SER | A | 341 | 39.439 | 77.973 | 58.914 | 1.00 | 2.24 |
| ATOM | 4735 | CB | SER | A | 341 | 39.061 | 77.555 | 56.842 | 1.00 | 2.24 |
| ATOM | 4736 | HB1 | SER | A | 341 | 39.504 | 76.972 | 56.032 | 1.00 | 2.24 |
| ATOM | 4737 | HB2 | SER | A | 341 | 38.962 | 78.596 | 56.528 | 1.00 | 2.24 |
| ATOM | 4738 | OG | SER | A | 341 | 37.785 | 77.039 | 57.168 | 1.00 | 2.24 |
| ATOM | 4739 | HG | SER | A | 341 | 37.232 | 77.047 | 56.377 | 1.00 | 2.24 |
| ATOM | 4740 | C | SER | A | 341 | 40.219 | 76.040 | 58.456 | 1.00 | 2.24 |
| ATOM | 4741 | O | SER | A | 341 | 41.197 | 75.451 | 57.990 | 1.00 | 2.24 |
| ATOM | 4742 | N | CYS | A | 342 | 39.352 | 75.472 | 59.288 | 1.00 | 2.60 |
| ATOM | 4743 | H | CYS | A | 342 | 38.537 | 75.996 | 59.569 | 1.00 | 2.60 |
| ATOM | 4744 | CA | CYS | A | 342 | 39.337 | 74.028 | 59.470 | 1.00 | 2.60 |
| ATOM | 4745 | HA | CYS | A | 342 | 40.373 | 73.692 | 59.497 | 1.00 | 2.60 |
| ATOM | 4746 | CB | CYS | A | 342 | 38.751 | 73.673 | 60.839 | 1.00 | 2.60 |
| ATOM | 4747 | HB1 | CYS | A | 342 | 39.146 | 74.374 | 61.573 | 1.00 | 2.60 |
| ATOM | 4748 | HB2 | CYS | A | 342 | 39.122 | 72.682 | 61.101 | 1.00 | 2.60 |

```
ATOM   4749  SG  CYS A 342      36.952  73.608  61.019  1.00  2.60
ATOM   4750  C   CYS A 342      38.698  73.289  58.298  1.00  2.60
ATOM   4751  O   CYS A 342      38.709  72.064  58.280  1.00  2.60
ATOM   4752  N   SER A 343      38.218  74.034  57.302  1.00  3.76
ATOM   4753  H   SER A 343      38.168  75.036  57.416  1.00  3.76
ATOM   4754  CA  SER A 343      37.734  73.463  56.062  1.00  3.76
ATOM   4755  HA  SER A 343      37.536  72.404  56.234  1.00  3.76
ATOM   4756  CB  SER A 343      36.378  74.042  55.652  1.00  3.76
ATOM   4757  HB1 SER A 343      36.053  73.564  54.727  1.00  3.76
ATOM   4758  HB2 SER A 343      36.472  75.115  55.485  1.00  3.76
ATOM   4759  OG  SER A 343      35.415  73.798  56.657  1.00  3.76
ATOM   4760  HG  SER A 343      35.307  72.811  56.727  1.00  3.76
ATOM   4761  C   SER A 343      38.727  73.563  54.922  1.00  3.76
ATOM   4762  O   SER A 343      39.039  74.658  54.446  1.00  3.76
ATOM   4763  N   THR A 344      39.152  72.375  54.491  1.00  4.68
ATOM   4764  H   THR A 344      38.789  71.590  55.021  1.00  4.68
ATOM   4765  CA  THR A 344      39.593  72.028  53.134  1.00  4.68
ATOM   4766  HA  THR A 344      40.492  71.420  53.213  1.00  4.68
ATOM   4767  CB  THR A 344      38.507  71.162  52.450  1.00  4.68
ATOM   4768  HB  THR A 344      38.560  71.290  51.369  1.00  4.68
ATOM   4769  CG2 THR A 344      38.716  69.686  52.772  1.00  4.68
ATOM   4770 1HG2 THR A 344      37.951  69.092  52.272  1.00  4.68
ATOM   4771 2HG2 THR A 344      39.693  69.372  52.412  1.00  4.68
ATOM   4772 3HG2 THR A 344      38.653  69.520  53.846  1.00  4.68
ATOM   4773  OG1 THR A 344      37.193  71.482  52.883  1.00  4.68
ATOM   4774  HG1 THR A 344      36.989  70.947  53.681  1.00  4.68
ATOM   4775  C   THR A 344      39.981  73.181  52.203  1.00  4.68
ATOM   4776  O   THR A 344      39.925  74.352  52.578  1.00  4.68
TER
```
